# Risk of hydroxychloroquine alone and in combination with azithromycin in the treatment of rheumatoid arthritis: a multinational, retrospective study

**DOI:** 10.1016/S2665-9913(20)30276-9

**Published:** 2020-08-21

**Authors:** Jennifer C E Lane, James Weaver, Kristin Kostka, Talita Duarte-Salles, Maria Tereza F Abrahao, Heba Alghoul, Osaid Alser, Thamir M Alshammari, Patricia Biedermann, Juan M Banda, Edward Burn, Paula Casajust, Mitchell M Conover, Aedin C Culhane, Alexander Davydov, Scott L DuVall, Dmitry Dymshyts, Sergio Fernandez-Bertolin, Kristina Fišter, Jill Hardin, Laura Hester, George Hripcsak, Benjamin Skov Kaas-Hansen, Seamus Kent, Sajan Khosla, Spyros Kolovos, Christophe G Lambert, Johan van der Lei, Kristine E Lynch, Rupa Makadia, Andrea V Margulis, Michael E Matheny, Paras Mehta, Daniel R Morales, Henry Morgan-Stewart, Mees Mosseveld, Danielle Newby, Fredrik Nyberg, Anna Ostropolets, Rae Woong Park, Albert Prats-Uribe, Gowtham A Rao, Christian Reich, Jenna Reps, Peter Rijnbeek, Selva Muthu Kumaran Sathappan, Martijn Schuemie, Sarah Seager, Anthony G Sena, Azza Shoaibi, Matthew Spotnitz, Marc A Suchard, Carmen O Torre, David Vizcaya, Haini Wen, Marcel de Wilde, Junqing Xie, Seng Chan You, Lin Zhang, Oleg Zhuk, Patrick Ryan, Daniel Prieto-Alhambra, OHDSI-COVID-19 consortium

**Affiliations:** a Centre for Statistics in Medicine, Nuffield Department of Orthopaedics, Rheumatology, and Musculoskeletal Sciences, University of Oxford, Oxford, UK; b Janssen Research and Development, Titusville, NJ, USA; c Real World Solutions, IQVIA, Cambridge, MA, USA; d Fundació Institut Universitari per a la Recerca a l'Atenció Primària de Salut Jordi Gol i Gurina (IDIAPJGol), Barcelona, Spain; e Faculty of Medicine, University of Sao Paulo, Sao Paulo, Brazil; f Faculty of Medicine, Islamic University of Gaza, Palestine; g Massachusetts General Hospital, Harvard Medical School, Boston, MA, USA; h Medication Safety Research Chair, King Saud University, Riyadh, Saudi Arabia; i Actelion Pharmaceuticals, Allschwil, Switzerland; j Department of Computer Science, Georgia State University, Atlanta, GA, USA; k Real-World Evidence, Trial Form Support, Barcelona, Spain; l Department of Data Sciences, Dana-Farber Cancer Institute, Department of Biostatistics, Harvard T H Chan School of Public Health, Boston, MA, USA; m Medical Ontology Solutions, Odysseus Data Services, Cambridge MA, USA; n Western Institute for Biomedical Research, Department of Veterans Affairs, Salt Lake City, UT, USA; o Department of Internal Medicine, Division of Epidemiology, University of Utah School of Medicine, Salt Lake City, UT, USA; p School of Medicine, Andrija Štampar School of Public Health, University of Zagreb, Zagreb, Croatia; q Department of Biomedical Informatics, Columbia University Irving Medical Center, New York, NY, USA; r New York-Presbyterian Hospital, New York, NY, USA; s Clinical Pharmacology Unit, Zealand University Hospital, Roskilde, Denmark; t NNF Centre for Protein Research, University of Copenhagen, Copenhagen, Denmark; u National Institute for Health and Care Excellence, London, UK; v Real World Science and Digital, AstraZeneca, Cambridge, UK; w Department of Internal Medicine, Center for Global Health and Division of Translational Informatics, Albuquerque, NM, USA; x Department of Medical Informatics, Erasmus University Medical Center, Rotterdam, Netherlands; y RTI Health Solutions, Barcelona, Spain; z Geriatrics Research Education and Clinical Care Center, Tennessee Valley Healthcare System VA, Nashville, TN, USA; aa Department of Biomedical Informatics, Vanderbilt University Medical Center, Nashville, TN, USA; ab College of Medicine, University of Arizona, Tucson, AZ, USA; ac Division of Population Health and Genomics, University of Dundee, UK; ad Department of Psychiatry, University of Oxford, Warneford Hospital, Oxford, UK; ae School of Public Health and Community Medicine, Institute of Medicine, Sahlgrenska Academy, University of Gothenburg, Gothenburg, Sweden; af Department of Biomedical Informatics, Ajou University School of Medicine, Suwon-si Gyeonggi-do, South Korea; ag Saw Swee Hock School of Public Health, National University of Singapore, Singapore; ah Department of Biomathematics and Department of Human Genetics, David Geffen School of Medicine at UCLA, and Department of Biostatistics, UCLA Fielding School of Public Health, University of California, Los Angeles, CA, USA; ai Bayer Pharmaceuticals, Barcelona, Spain; aj Department of Pharmacy, Shanghai Chest Hospital, Shanghai Jiao Tong University, Shanghai, China; ak School of Population Medicine and Public Health, Peking Union Medical College/Chinese Academy of Medical Sciences, Beijing, China; al Melbourne School of Population and Global Health, University of Melbourne, VIC, Australia

## Abstract

**Background:**

Hydroxychloroquine, a drug commonly used in the treatment of rheumatoid arthritis, has received much negative publicity for adverse events associated with its authorisation for emergency use to treat patients with COVID-19 pneumonia. We studied the safety of hydroxychloroquine, alone and in combination with azithromycin, to determine the risk associated with its use in routine care in patients with rheumatoid arthritis.

**Methods:**

In this multinational, retrospective study, new user cohort studies in patients with rheumatoid arthritis aged 18 years or older and initiating hydroxychloroquine were compared with those initiating sulfasalazine and followed up over 30 days, with 16 severe adverse events studied. Self-controlled case series were done to further establish safety in wider populations, and included all users of hydroxychloroquine regardless of rheumatoid arthritis status or indication. Separately, severe adverse events associated with hydroxychloroquine plus azithromycin (compared with hydroxychloroquine plus amoxicillin) were studied. Data comprised 14 sources of claims data or electronic medical records from Germany, Japan, the Netherlands, Spain, the UK, and the USA. Propensity score stratification and calibration using negative control outcomes were used to address confounding. Cox models were fitted to estimate calibrated hazard ratios (HRs) according to drug use. Estimates were pooled where the *I*^2^ value was less than 0·4.

**Findings:**

The study included 956 374 users of hydroxychloroquine, 310 350 users of sulfasalazine, 323 122 users of hydroxychloroquine plus azithromycin, and 351 956 users of hydroxychloroquine plus amoxicillin. No excess risk of severe adverse events was identified when 30-day hydroxychloroquine and sulfasalazine use were compared. Self-controlled case series confirmed these findings. However, long-term use of hydroxychloroquine appeared to be associated with increased cardiovascular mortality (calibrated HR 1·65 [95% CI 1·12–2·44]). Addition of azithromycin appeared to be associated with an increased risk of 30-day cardiovascular mortality (calibrated HR 2·19 [95% CI 1·22–3·95]), chest pain or angina (1·15 [1·05–1·26]), and heart failure (1·22 [1·02–1·45]).

**Interpretation:**

Hydroxychloroquine treatment appears to have no increased risk in the short term among patients with rheumatoid arthritis, but in the long term it appears to be associated with excess cardiovascular mortality. The addition of azithromycin increases the risk of heart failure and cardiovascular mortality even in the short term. We call for careful consideration of the benefit–risk trade-off when counselling those on hydroxychloroquine treatment.

**Funding:**

National Institute for Health Research (NIHR) Oxford Biomedical Research Centre, NIHR Senior Research Fellowship programme, US National Institutes of Health, US Department of Veterans Affairs, Janssen Research and Development, IQVIA, Korea Health Industry Development Institute through the Ministry of Health and Welfare Republic of Korea, Versus Arthritis, UK Medical Research Council Doctoral Training Partnership, Foundation Alfonso Martin Escudero, Innovation Fund Denmark, Novo Nordisk Foundation, Singapore Ministry of Health's National Medical Research Council Open Fund Large Collaborative Grant, VINCI, Innovative Medicines Initiative 2 Joint Undertaking, EU's Horizon 2020 research and innovation programme, and European Federation of Pharmaceutical Industries and Associations.

**Research in context**

**Evidence before this study**

We systematically searched PubMed, Embase, clinical trial registries (ClinicalTrials.gov, the International Clinical Trials Registry Platform Search Portal, and the Chinese Clinical Trial Registry), and preprint servers (*bioRxiv* and *medRxiv*) from inception until March 27, 2020 (appendix pp 126–30) for research articles in English, Chinese, Spanish, and Italian (see appendix p 126 for search terms). No contemporary large-scale evidence was found that investigated the real-world safety of hydroxychloroquine compared with other first-line disease-modifying antirheumatic drugs, especially in combination with macrolide antibiotics such as azithromycin, which have been proposed for use as a treatment for COVID-19. Systematic reviews that have informed European guidelines focused on severe adverse events associated with biological therapies with little high-level evidence focused on hydroxychloroquine. Severe cardiovascular adverse events, mostly lethal arrhythmias and heart failure, have been described in independent retrospective case series and case reports, and reported within the US Food and Drug Administration adverse events database.

**Added value of this study**

This study uses state-of-the-art methods to control for residual confounding and bias and shows comparable results across 14 international health databases. Hydroxychloroquine does not seem to confer increased risk when used in patients with rheumatoid arthritis without contraindications in the short term (up to 30 days) compared with sulfasalazine, but confers an increased risk of cardiovascular mortality when used long term. Short-term treatment with hydroxychloroquine plus azithromycin appears to be associated with elevated risk of cardiovascular mortality, angina, and heart failure compared with hydroxychloroquine plus amoxicillin.

**Implications of all the available evidence**

Short-term use of hydroxychloroquine appears to confer no increased risk in patients with rheumatoid arthritis without contraindications, but hydroxychloroquine in combination with azithromycin appears to be associated with serious cardiovascular adverse events and should therefore be used with caution.

## Introduction

Hydroxychloroquine, which is most commonly used as the first-line treatment in patients with autoimmune diseases such as rheumatoid arthritis and systemic lupus erythematosus (SLE), has gained extensive media coverage as a potential antiviral agent for use against severe acute respiratory syndrome coronavirus 2 (SARS-CoV-2), which causes COVID-19.^1^, ^2^, ^3^, ^4^, ^5^ Unfortunately, the exponential generation of research into hydroxychloroquine has led to confusion in the rheumatological community regarding the safety implications of hydroxychloroquine within its traditional uses.

Early in the COVID-19 pandemic, publicity focused on a study from France^6^ showing faster recovery and reduction in viral load in patients treated with high-dose hydroxychloroquine plus azithromycin, a macrolide antibiotic, compared with patients receiving standard treatment available at the time. This report led to widespread use of high-dose hydroxychloroquine either alone or with azithromycin. Subsequently, serious cardiac adverse events associated with QT segment prolongation that could lead to potentially lethal arrhythmia and cardiovascular-related death were identified in patients taking hydroxychloroquine in several health-care centres in the USA and Brazil.^7^, ^8^, ^9^, ^10^ Because of these reports of increased risk, emergency authorisation of hydroxychloroquine by medicines regulators was retracted, statements cautioning against hydroxychloroquine use were released, and randomised trials were stopped.^10^, ^11^, ^12^, ^13^, ^14^, ^15^

European guidelines for the treatment of patients with rheumatoid arthritis contain little high-level evidence for the safety of hydroxychloroquine, and most systematic reviews of rheumatoid arthritis treatments have focused on biological therapies.^16^, ^17^ Before the COVID-19 pandemic, evidence for hydroxychloroquine safety was largely found in retrospective case series and case reports, or within pharmaceutical adverse events registers.^18^, ^19^, ^20^ Azithromycin and macrolides in general are also known to induce cardiotoxicity and to interact with other drugs that prolong QTc.^21^, ^22^, ^23^

The combination of minimal large-scale hydroxychloroquine safety studies before this pandemic, and the extensive research suggesting risks associated with hydroxychloroquine use that has been produced during 2020 is of great concern to both patients and clinicians. We therefore aimed to assess the safety of hydroxychloroquine alone compared with sulfasalazine and of hydroxychloroquine in combination with azithromycin (compared with hydroxychloroquine in combination with amoxicillin), in part to provide clarity for patients taking hydroxychloroquine for rheumatoid arthritis.

## Methods

### Study design and participants

In this multinational, retrospective study, new user cohort studies were used as recommended by methodological guidelines^24^ for observational drug safety research to estimate the safety of hydroxychloroquine alone or in combination with macrolide antibiotics in patients with rheumatoid arthritis. Sulfasalazine and amoxicillin were chosen as active comparators because they have similar indications as the target treatments (hydroxychloroquine and azithromycin, respectively). Participants were included if they had a history of rheumatoid arthritis (a condition occurrence or observation indicating rheumatoid arthritis any time before or on the same day as therapy initiation), were aged 18 years or older at the index event, and had at least 365 days of continuous observation time before the index event.

As a secondary analysis, a self-controlled case series was used to estimate the safety of hydroxychloroquine in the wider population, including patients without rheumatoid arthritis. For this analysis, all prevalent users of hydroxychloroquine were included, regardless of rheumatoid arthritis status or indication.

All data partners received approval or waiver from their institutional review boards in accordance with their institutional governance guidelines. The full study protocol is available online.

### Data sources

Electronic health records (EHRs) and administrative claims data were mapped to the Observational Medical Outcomes Partnership common data model (version 5.0 or higher) and analysed in a distributed network as part of an international effort with the Observational Health Data Science and Informatics community, including 14 databases: IQVIA (Durham, NC, USA) Disease Analyzer Germany (ambulatory electronic medical record [EMR] from Germany); Japanese Medical Data Center Claims Database (Tokyo, Japan); Integrated Primary Care Information (IPCI; Rotterdam, Netherlands; primary care EMR); Information System for the Development of Research in Primary Care (SIDIAP; Barcelona, Spain; primary care EMR); Clinical Practice Research Datalink (CPRD; London, UK) and IQVIA UK (London, UK) Integrated Medical Record Data (IMRD; primary care EMRs); and IBM MarketScan (Somers, NY, USA) Commercial Claims and Encounters (CCAE), Optum (Eden Prairie, MN, USA) de-identified Clinformatics Data Mart Database (Clinformatics), Optum EHR (Optum de-identified Electronic Health Record dataset), IBM MarketScan Medicare Supplemental Database (MDCR), IBM MarketScan Multi-State Medicaid Database (MDCD), IQVIA Open Claims, US Department of Veterans Affairs (VA; Salt Lake City, UT, USA), and IQVIA US Ambulatory EMR (USA).

Self-controlled case series were done on a subset of these databases as a secondary analysis: CCAE, CPRD, Clinformatics, MDCD, MDCR, and VA. A description of these data sources is available in the appendix (pp 3–4).

### Study period and outcomes

The study period started from Sept 1, 2000, and ended at the latest available date for all data sources in 2020. Follow-up for each of the cohorts started at an index date defined by the first dispensing or prescription of the target or comparator drug as described in the cohort definitions (appendix pp 5–8). Two periods were considered to define time at risk. For a short-term, intention-to-treat analysis, follow-up started 1 day after the index date and continued until the first of: outcome of interest, loss to follow-up, or 30 days after the index date to resemble the duration of COVID-19 treatment regimens.^6^ For a longer-term, on-treatment analysis, follow-up started 1 day after the index date and continued until the earliest of: outcome of interest, loss to follow-up, or discontinuation, with an added washout time of 14 days. Continued use of the same treatment was inferred by allowing up to 90-day gaps between dispensing or prescription records. Additional detail on the exposure cohorts is available in the appendix (pp 5–8).

For self-controlled case series, periods of persistent exposure to hydroxychloroquine were generated allowing up to 90-day gaps between dispensing or prescription records. Patients were followed up for their entire observation time (eg, from enrolment to disenrolment in each database), and rates of each of the outcomes calculated in periods of exposure and non-exposure time.

The proposed code lists for the identification of the study population and for the study exposures were created by clinicians with experience in the management of rheumatoid arthritis using ATLAS, and reviewed by four clinicians and one epidemiologist.^25^

16 severe adverse events were analysed. Hospital-based events, which are not available in primary care records (CPRD, IMRD, and SIDIAP), included gastrointestinal bleeding, acute renal failure, acute pancreatitis, myocardial infarction, stroke, transient ischaemic attack, and cardiovascular events (composite). Additionally, angina or chest pain, heart failure, cardiac arrhythmia, bradycardia, venous thromboembolism, end-stage renal disease, and hepatic failure were analysed from both primary and secondary care data. All-cause mortality outcomes were obtained only from data sources with reliable information on death date (CPRD, IMRD, IPCI, Clinformatics, SIDIAP, and VA) and cardiovascular mortality outcomes from sources with information on cardiovascular events preceding death (CPRD, IMRD, Clinformatics, and VA). All codes for the identification of the 16 proposed study outcomes were based on a previously published paper^26^ and are detailed in the appendix (pp 8–9). Face validity for each of the outcome cohorts was further reviewed and compared with previous clinical knowledge and existing literature.

A list of negative control outcomes was also assessed for which there is no known causal relationship with any of the drugs of interest. These outcomes were identified using a semi-automatic process based on data extracted from the literature, product labels, and spontaneous reports, and confirmed by manual review by three clinicians (JCEL, AP-U, and DP-A).^27^ A full list of the codes that were used to identify negative control outcomes and details on covariate and confounder identification are provided in the appendix (pp 10–11).

### Statistical analysis

We used propensity score stratification (into quintiles) to adjust for observed confounders, using a large-scale regularised logistic regression fitted with a LASSO penalty and with the optimal hyperparameter determined through ten-fold cross-validation.^28^ Baseline patient characteristics were constructed for inclusion as potentially confounding covariates.^29^ Predictor variables included were based on all observed patient characteristics as available in each data source, including conditions, procedures, visits, observations, and measurements. We plotted the propensity score distribution and assessed covariate balance expressed as the standardised difference of the mean for every covariate before and after propensity score stratification. A standardised difference of more than 0·1 indicated a non-negligible imbalance between exposure cohorts.^30^ Cox proportional hazards models conditioned on the propensity score strata were fitted to estimate hazard ratios (HRs) according to treatment status. Negative control outcomes analyses and empirical calibration were used to minimise potential unresolved confounding, with calibrated HRs and 95% CIs estimated.^31^, ^32^

For self-controlled case series, safety of hydroxychloroquine therapy was assessed separately as a secondary analysis, regardless of indication, comparing exposed and unexposed time periods within the same individuals. The method is self-controlled in that it makes within-person comparisons of event rates during periods of hypothesised increased risk with other periods of baseline risk, which eliminates all time-invariant confounding. Because we do not compare between individuals, the self-controlled case series is robust to between-person differences, even including unmeasured differences (such as genetics). However, the method is vulnerable to time-varying confounders. To adjust for this confounding, we included many time-varying covariates in the models, including age, season, and other drug exposures. A conditional Poisson regression was used to fit the outcome model using the Cyclops package (version 2.0), with a hyperparameter selected through ten-fold cross-validation.^33^

Study diagnostics (power, propensity score distribution, covariate balance, and empirical null distribution) were evaluated by clinicians and epidemiologists to determine which database target comparator outcome analysis variants could produce unbiased estimates (appendix pp 104–18). Analyses with zero event outcomes or with confounder imbalances with standardised mean difference of more than 0·1 after stratification were excluded from analysis. All analyses were conducted for each database separately, with estimates combined in random-effects meta-analysis methods where the *I*^2^ value was less than 0·4.^34^ The standard errors of the database-specific estimates were adjusted to incorporate estimate variation across databases, where the across-database variance was estimated by comparing each database-specific result to that of an inverse-variance, fixed-effects meta-analysis. No meta-analysis was done where *I*^2^ for a given drug–outcome pair was 0·4 or more. Of note, when running analysis in a distributed network, it was not possible to link across datasets, and to know the extent of overlap between data.

Small cell counts (n) of less than five (and resulting estimates) are reported as <n to minimise risk of re-identification. For the cohort analysis, the CohortMethod package (version 3.1.0) was used as well as the Cyclops package (version 2.0) for propensity score estimation.^33^ All self-controlled case series were run using the self-controlled case series package.^35^ The full source code for analyses is available online.

This study is registered with the EU Post-Authorisation Studies Register, EUPAS34497.^36^

### Role of the funding source

The funders of the study had no role in study design, data collection, data analysis, data interpretation, writing of the manuscript, or the decision to submit for publication. All authors had full access to aggregated data in the study, and the lead and senior authors (JCEL, JW, PRy, and DP-A) had final responsibility for the decision to submit for publication.

## Results

956 374 hydroxychloroquine and 310 350 sulfasalazine users were identified, and 323 122 and 351 956 contributed to the analyses of combination therapy of hydroxychloroquine plus azithromycin compared with hydroxychloroquine plus amoxicillin, respectively. Participant counts in each data source are provided in the appendix (pp 13–65). Duration of hydroxychloroquine therapy in the long-term analysis varied between databases, and ranged from a median of 43 days (IQR 43–193) in IQVIA US Ambulatory EMR to 338 days (106–1507) in CPRD. Full details can be found in the power tab for each database online.

Compared with sulfasalazine, users of hydroxychloroquine were more likely to be female (eg, 82·0% *vs* 74·3% in CCAE) and less likely to have certain comorbidities such as Crohn's disease (0·6% *vs* 1·8% in CCAE) or psoriasis (3·0% *vs* 8·9% in CCAE; appendix pp 15–16). In CCAE, the mean baseline dose for hydroxychloroquine was 420 mg (SD 463), and 2·8% of patients had an estimated dose of more than 500 mg. All differences were minimised after propensity score stratification, with all reported analyses balanced on all identified confounders. For example, systemic corticosteroid use or a diagnosis of SLE in the year before hydroxychloroquine or sulfasalazine use before propensity score matching was imbalanced but was balanced through propensity score stratification. Full details of all of the variables used within the propensity score are available in the shiny application (population characteristics tab, searching for the variable within the raw setting). Similarly, users of combination hydroxychloroquine plus azithromycin differed from those of hydroxychloroquine plus amoxicillin, with a higher prevalence of acute respiratory disease among azithromycin users (eg, 62·5% *vs* 50·7% in CCAE; appendix p 43). Again, propensity score methods mitigated these differences, and comparison groups became balanced for all observed confounders after stratification. Detailed baseline characteristics for the two pairs of treatment groups after propensity score stratification in CCAE are detailed in table 1 for illustrative purposes, and a complete list of features for each database comparing before and after propensity score stratification are provided in the appendix (pp 13–65). Propensity score distribution plots and negative control outcome analyses can be found in the appendix (pp 104–118) in addition to all elements of the propensity model and Kaplan-Meier analyses.

**Table 1:** Baseline characteristics of users of HCQ versus SSZ, and HCQ plus AZM versus HCQ plus AMX after propensity score stratification in the CCAE database

	**HCQ *vs* SSZ**			**HCQ plus AZM *vs* HCQ plus AMX**		
	HCQ (n=66 604)	SSZ (n=22 370)	Standardised mean difference	HCQ plus AZM (n=32 586)	HCQ plus AMX (n=32 496)	Standard mean difference
**Age, years**						
15–19	0·6%	0·6%	0·00	0·5%	0·5%	<0·00
20–24	1·8%	2·0%	−0·01	1·4%	1·4%	<0·00
25–29	2·5%	2·7%	−0·01	2·2%	2·2%	<0·00
30–34	4·5%	4·4%	<0·00	4·0%	3·9%	0·01
35–39	7·1%	7·1%	0·00	6·8%	6·7%	<0·00
40–44	9·7%	9·5%	0·01	9·3%	9·3%	<0·00
45–49	13·6%	13·4%	<0·00	13·2%	13·3%	<0·00
50–54	18·2%	18·1%	0·01	18·1%	18·0%	<0·00
55–59	20·8%	20·8%	<0·00	21·5%	21·8%	−0·01
60–64	19·4%	19·8%	−0·01	21·1%	21·1%	<0·00
65–69	1·8%	1·6%	0·01	2·0%	2·0%	<0·00
**Sex**						
Female	80·1%	79·7%	0·01	86·3%	86·2%	0·00
Male	19·9%	20·3%	0·01	13·7%	13·8%	0·00
**Medical history: general**						
Chronic obstructive lung disease	4·3%	4·5%	−0·01	5·0%	5·2%	−0·01
Depressive disorder	13·3%	13·5%	<0·00	14·7%	14·8%	<0·00
Diabetes	13·6%	13·8%	−0·01	13·2%	13·1%	<0·00
Hyperlipidaemia	31·2%	31·4%	<0·00	30·4%	30·3%	<0·00
Pneumonia	4·0%	4·0%	<0·00	5·7%	5·5%	0·01
Renal impairment	3·0%	2·8%	0·01	4·2%	4·1%	<0·00
Urinary tract infections	11·6%	11·5%	<0·00	14·0%	13·9%	<0·00
**Medical history: cardiovascular disease**						
Atrial fibrillation	1·4%	1·3%	0·01	1·7%	1·8%	<0·00
Cerebrovascular disease	2·8%	2·9%	−0·01	3·1%	3·2%	−0·01
Coronary arteriosclerosis	4·4%	4·6%	−0·01	5·0%	4·9%	<0·00
Heart disease	15·5%	15·4%	<0·00	17·8%	17·9%	<0·00
Heart failure	1·9%	2·0%	<0·00	2·5%	2·4%	0·01
Ischaemic heart disease	3·0%	3·1%	−0·01	3·3%	3·1%	0·01
**Medication use**						
Agents acting on the renin–angiotensin system	24·5%	24·6%	<0·00	27·1%	26·9%	<0·00
Antidepressants	36·3%	36·5%	<0·00	43·0%	42·8%	<0·00
Drugs for obstructive airway diseases	29·5%	29·5%	<0·00	41·1%	40·7%	0·01
Immunosuppressants	43·4%	43·6%	<0·00	51·1%	51·2%	<0·00
Opioids	39·0%	39·3%	−0·01	41·4%	41·2%	<0·00
Psycholeptics	33·4%	33·3%	<0·00	38·2%	38·1%	<0·00

Percentages might not sum to 100% because of rounding. An example of one dataset is included. AMX=amoxicillin. AZM=azithromycin. CCAE=IBM Commercial Claims and Encounters. HCQ=hydroxychloroquine. SSZ=sulfasalazine.

Database-specific and subtotal (meta-analysis) counts and rates of key outcomes (cardiovascular mortality, all-cause mortality, chest pain or angina, and heart failure) observed in the prespecified 30-day intention-to-treat analysis are shown in Table 2, Table 3. Mortality risk was assessed only using databases with reliable death capture: Clinformatics, CPRD, IMRD, IPCI, SIDIAP, and VA. For the analysis of hydroxychloroquine versus sulfasalazine, four databases (Clinformatics, CPRD, IMRD, and VA) were used to analyse all-cause mortality (no events were seen in SIDIAP and IPCI), and three databases (Clinformatics, CPRD, and VA) were used to analyse cardiovascular mortality. Two databases were used to analyse all-cause mortality and cardiovascular mortality for hydroxychloroquine plus azithromycin versus hydroxychloroquine plus amoxicillin (Clinformatics and VA); no events were seen in the other datasets. Mortality rates ranged from 4·81 per 1000 person-years in hydroxychloroquine users in Clinformatics to 17·13 per 1000 person-years among hydroxychloroquine users in VA, with cardiovascular-specific mortality ranging from 3·43 per 1000 person-years in hydroxychloroquine users in VA to less than 4·25 per 1000 person-years in sulfasalazine users in the same data source. Database-specific counts and incidence rates for severe adverse events stratified by drug use are detailed in full in the appendix (pp 66–71).

**Table 2:** Patient counts, event counts, and incidence rates of key outcomes according to HCQ versus SSZ use

	**30-day follow-up**						**On-treatment follow-up**					
	HCQ users	SSZ users	HCQ events	SSZ events	HCQ incidence rate (per 1000 person-years)	SSZ incidence rate (per 1000 person-years)	HCQ users	SSZ users	HCQ events	SSZ events	HCQ incidence rate (per 1000 person-years)	SSZ incidence rate (per 1000 person-years)
**Cardiovascular-related mortality**												
Clinformatics	51 280	17 389	16	<5	3·85	<3·54	51 280	17 389	234	25	4·39	2·00
CPRD	NA	NA	NA	NA	NA	NA	9127	11 398	7	25	0·39	0·94
VA	32 028	14 349	9	<5	3·43	<4·25	32 028	14 349	315	65	5·69	3·71
Meta-analysis	83 308	31 738	25	<10	3·68	<3·86	92 435	43 136	556	115	4·39	2·03
**All-cause mortality**												
Clinformatics	51 280	17 389	20	10	4·81	7·09	51 280	17 389	527	66	9·88	5·29
CPRD	9127	11 398	6	5	8·03	5·35	9127	11 398	253	386	14·02	14·56
IMRD	8851	8460	<5	6	<6·91	8·66	8851	8460	214	241	12·32	12·72
VA	32 028	14 349	45	17	17·13	14·45	32 028	14 349	1356	327	24·51	18·65
Meta-analysis	101 286	51 596	<76	38	<9·20	9·02	NA	NA	NA	NA	NA	NA
**Chest pain or angina**												
AmbEMR	57 140	15 268	122	31	26·04	24·76	57 140	15 268	451	112	24·44	19·89
CCAE	65 935	22 173	440	143	82·41	79·62	65 935	22 173	3354	810	55·00	58·80
Clinformatics	50 698	17 221	396	166	96·62	119·34	50 698	17 221	3185	829	66·13	72·48
CPRD	9114	11 388	10	17	13·40	18·22	9114	11 388	260	422	14·99	16·78
DAGermany	3884	5045	<5	5	<15·69	12·07	3884	5045	31	36	12·36	10·26
IMRD	8843	8452	9	10	12·45	14·46	8843	8452	235	293	14·00	16·25
MDCD	7982	2177	80	23	123·50	130·43	7982	2177	467	100	87·34	85·81
MDCR	15 690	5150	129	49	101·25	117·43	15 690	5150	1178	279	71·38	75·12
OpenClaims	617 628	182 776	2674	804	52·83	53·68	617 628	182 776	31 161	6198	38·59	38·11
OptumEHR	76 844	21 549	629	143	101·46	82·23	NA	NA	NA	NA	NA	NA
VA	31 824	14 276	130	54	49·89	46·20	31 824	14 276	1822	611	35·88	37·31
Meta-analysis	945 582	305 475	<4624	1445	<59·86	57·90	868 738	283 926	42 144	9690	40·36	37·07
**Heart failure**												
AmbEMR	57 383	15 305	42	10	8·92	7·96	57 383	15 305	182	53	9·76	9·37
CCAE	66 604	22 370	30	5	5·55	2·75	66 604	22 370	305	74	4·64	5·07
Clinformatics	51 204	17 356	84	25	20·23	17·76	51 204	17 356	915	207	17·55	16·90
CPRD	9126	11 397	<5	<5	<6·69	<5·35	9126	11 397	16	36	0·89	1·36
DAGermany	3885	5042	<5	<5	<15·68	<12·08	3885	5042	11	22	4·29	6·22
IMRD	8852	8460	<5	<5	<6·91	<7·22	8852	8460	15	21	0·86	1·11
MDCD	8072	2195	15	<5	22·81	<27·99	8072	2195	118	28	20·55	23·02
MDCR	15 808	5171	39	19	30·30	45·22	15 808	5171	586	141	33·13	36·29
OpenClaims	620 244	183 350	749	214	14·71	14·22	620 244	183 350	12 246	2246	14·36	13·22
OptumEHR	77 813	21 768	237	50	37·64	28·39	NA	NA	NA	NA	NA	NA
VA	31 895	14 307	56	17	21·42	14·49	31 895	14 307	897	296	16·75	17·42
Meta-analysis	950 886	306 721	<1267	<360	<16·28	<14·34	873 073	284 953	15 291	3124	13·85	11·43

AmbEMR=IQVIA Ambulatory EMR. CCAE=IBM Commercial Claims and Encounters. CPRD=Clinical Practice Research Datalink. DAGermany=IQVIA Disease Analyzer Germany. EMR=electronic medical record. HCQ=hydroxychloroquine. IMRD=IQVIA UK Integrated Medical Record Data. MDCD=IBM Multi-state Medicaid. MDCR=IBM Medicare Supplemental Database. NA=non-applicable (not reported because of failed diagnostics or on-treatment follow-up unavailable). OptumEHR=Optum de-identified Electronic Health Record. SSZ=sulfasalazine. VA=US Department of Veterans Affairs.

**Table 3:** Patient counts, event counts, and incidence rates of key outcomes according to HCQ plus AZM versus HCQ plus AMX use

	**30-day follow-up**						**On-treatment follow-up**					
	HCQ plus AZM users	HCQ plus AMX users	HCQ plus AZM events	HCQ plus AMX events	HCQ plus AZM incidence rate (per 1000 person-years)	HCQ plus AMX incidence rate (per 1000 person-years)	HCQ plus AZM users	HCQ plus AMX users	HCQ plus AZM events	HCQ plus AMX events	HCQ plus AZM incidence rate (per 1000 person-years)	HCQ plus AMX incidence rate (per 1000 person-years)
**Cardiovascular-related mortality**												
Clinformatics	23 597	24 521	9	6	4·70	3·02	23 597	24 521	96	82	5·56	5·58
VA	6234	8005	46	18	90·60	27·49	6234	8005	157	115	14·60	10·20
Meta-analysis	29 831	32 526	55	24	22·70	9·08	29 831	32 526	253	197	9·03	7·59
**All-cause mortality**												
Clinformatics	23 597	24 521	17	17	8·88	8·55	23 597	24 521	268	276	15·56	18·85
VA	6234	8005	91	52	179·23	79·42	6234	8005	550	518	51·16	45·97
Meta-analysis	29 831	32 526	108	69	44·58	26·12	29 831	32 526	818	794	29·24	30·64
CCAE	32 610	32 507	13	11	4·92	4·17	32 610	32 507	117	94	4·33	4·33
Clinformatics	23 565	24 484	30	29	15·70	14·62	23 565	24 484	179	147	10·60	10·19
MDCD	3803	3808	<5	6	<16·21	19·40	3803	3808	29	27	11·46	13·46
MDCR	8119	9254	16	9	24·33	11·96	8119	9254	166	140	20·41	17·34
Open Claims	216 028	232 938	182	173	10·26	9·05	216 028	232 938	2065	1732	8·11	7·94
OptumEHR	18 477	16 424	26	20	17·35	15·01	NA	NA	NA	NA	NA	NA
VA	6203	7978	33	19	65·53	29·15	6203	7978	154	127	14·79	11·59
Meta-analysis	308 805	327 393	<305	267	<12·08	9·97	290 328	310 969	2710	2267	8·48	8·24
**Chest pain or angina**												
AmbEMR	13 093	12 028	32	21	29·80	21·29	13 093	12 028	142	119	25·69	25·31
CCAE	32 165	32 229	241	211	92·76	80·98	32 165	32 229	1402	1145	60·46	60·54
Clinformatics	23 206	24 254	244	203	130·28	103·70	23 206	24 254	1019	887	70·33	70·28
MDCD	3712	3764	30	37	99·97	121·56	3712	3764	129	113	60·05	63·39
MDCR	7991	9195	81	85	125·60	114·20	7991	9195	517	498	74·83	71·25
OpenClaims	214 494	231 851	1050	888	59·76	46·74	214 494	231 851	8348	7223	36·24	36·37
OptumEHR	18 039	16 191	218	134	150·01	102·42	NA	NA	NA	NA	NA	NA
VA	6121	7912	58	50	116·96	77·52	6121	7912	340	371	38·48	39·87
Meta-analysis	318 821	337 424	1954	1629	75·13	59·12	300 782	321 233	11 897	10 356	40·82	40·95
**Heart failure**												
AmbEMR	13 152	12 053	16	16	14·83	16·18	13 152	12 053	61	49	10·44	9·96
CCAE	32 586	32 496	30	23	11·36	8·73	32 586	32 496	177	126	6·58	5·82
Clinformatics	23 541	24 468	65	49	34·08	24·73	23 541	24 468	337	317	20·33	22·63
MDCD	3796	3795	16	9	52·08	29·21	3796	3795	65	48	26·26	24·83
MDCR	8085	9239	45	33	68·88	43·97	8085	9239	322	295	41·61	38·34
OpenClaims	215 732	232 725	472	370	26·68	19·38	215 732	232 725	4352	3714	17·50	17·43
OptumEHR	18 054	16 298	99	60	67·77	45·45	NA	NA	NA	NA	NA	NA
VA	6164	7959	79	31	158·53	47·73	6164	7959	280	229	28·17	21·64
Meta-analysis	321 110	339 033	822	591	31·32	21·32	303 056	322 735	5594	4778	17·58	17·44

AmbEMR=IQVIA Ambulatory EMR. AMX=amoxicillin. AZM=azithromycin. CCAE=IBM Commercial Claims and Encounters. CPRD=Clinical Practice Research Datalink. DAGermany=IQVIA Disease Analyzer Germany. EMR=electronic medical record. HCQ=hydroxychloroquine. IMRD=IQVIA UK Integrated Medical Record Data. MDCD=IBM Multi-state Medicaid. MDCR=IBM Medicare Supplemental Database. NA=non-applicable (not reported because of failed diagnostics or on-treatment follow-up unavailable). OptumEHR=Optum de-identified Electronic Health Record. VA=US Department of Veterans Affairs.

Least common outcomes among hydroxychloroquine users included bradycardia (eg, incidence rate 0·92 per 1000 person-years in CCAE) and end-stage renal disease (eg, less than 0·92 per 1000 person-years in CCAE), whereas most common outcomes were chest pain or angina (eg, 82·41 per 1000 person-years in CCAE; table 2) and composite cardiovascular events (eg, 17·96 per 1000 person-years in CCAE).

**Figure 1:**
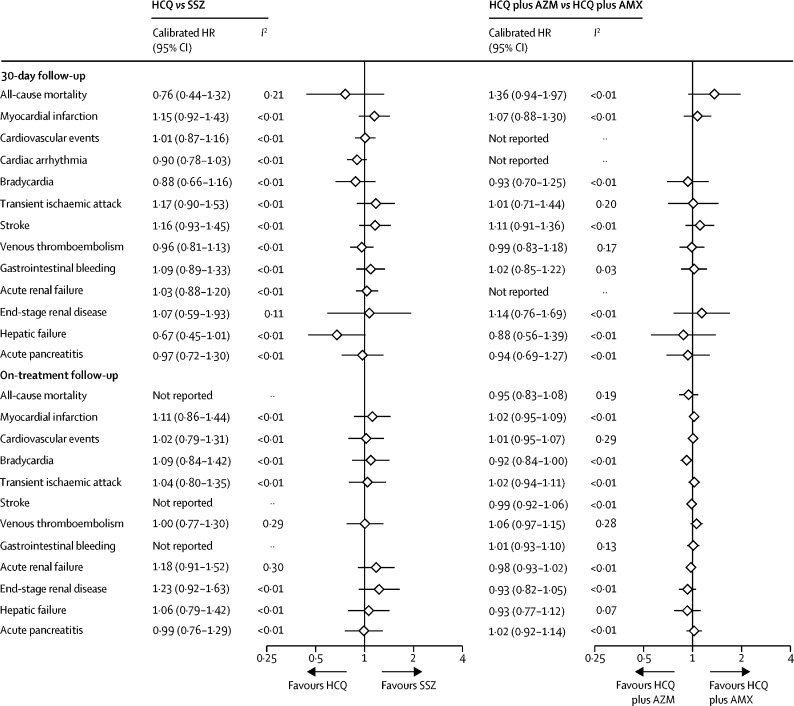
Meta-analytic estimates for HCQ versus SSZ and HCQ plus AZM versus HCQ plus AMX new users during 30-day (intention-to-treat) and long-term (on-treatment) follow-up AMX=amoxicillin. AZM=azithromycin. HCQ=hydroxychloroquine. HR=hazard ratio. SSZ=sulfasalazine.

**Figure 2:**
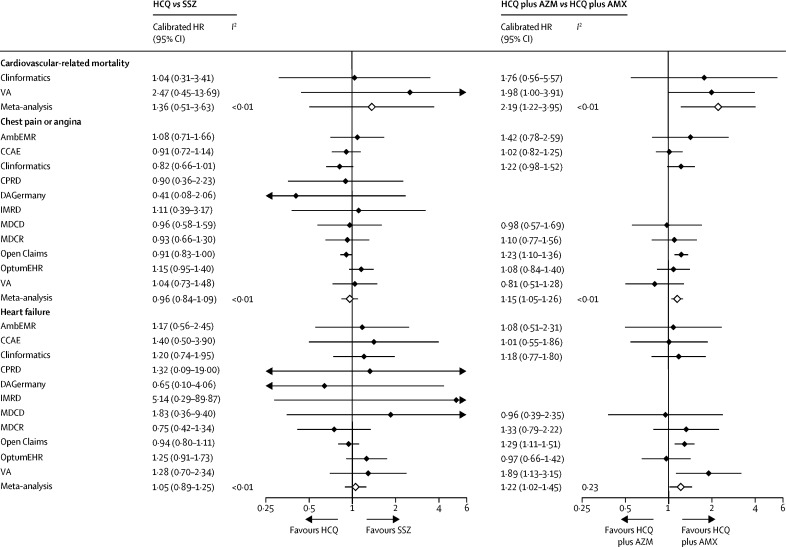
Source-specific and meta-analytic-specific severe adverse event risk estimates for HCQ versus SSZ and HCQ plus AZM versus HCQ plus AMX new users during 30-day (intention-to-treat) follow-up AmbEMR=IQVIA Ambulatory EMR. AMX=amoxicillin. AZM=azithromycin. CCAE=IBM Commercial Claims and Encounters. CPRD=Clinical Practice Research Datalink. DAGermany=IQVIA Disease Analyzer Germany. EMR=electronic medical record. HCQ=hydroxychloroquine. HR=hazard ratio. IMRD=IQVIA UK Integrated Medical Record Data. MDCD=IBM Multi-state Medicaid. MDCR=IBM Medicare Supplemental Database. OptumEHR=Optum de-identified Electronic Health Record. SSZ=sulfasalazine. VA=US Department of Veterans Affairs.

Database and outcome-specific HRs (uncalibrated and calibrated) are reported in full in the form of forest plots in the appendix (pp 72–103). None of the severe adverse events appeared to be consistently increased with the short-term use of hydroxychloroquine (*vs* sulfasalazine) in the 30-day intention-to-treat analyses (figure 1), with meta-analytic calibrated HRs ranging from 0·67 (95% CI 0·45–1·01) for hepatic failure to 1·17 (0·90–1·53) for transient ischaemic attack, and 1·36 (0·51–3·63) for cardiovascular mortality (figure 2). In our published study protocol, we decided a priori that meta-analytic estimates would only be reported if the *I*^2^ value was less than 0·4, indicating that there was low heterogeneity between the results included, and that it was appropriate for them to be pooled to produce this final result.^36^ For all-cause mortality in the on-treatment analysis, the *I*^2^ value was 0·71, indicating substantial heterogeneity between results and therefore a summary estimate was not reported. The same is true for gastrointestinal bleeding (*I*^2^=0·57) and stroke (*I*^2^=0·58) in the on-treatment analysis.

**Figure 3:**
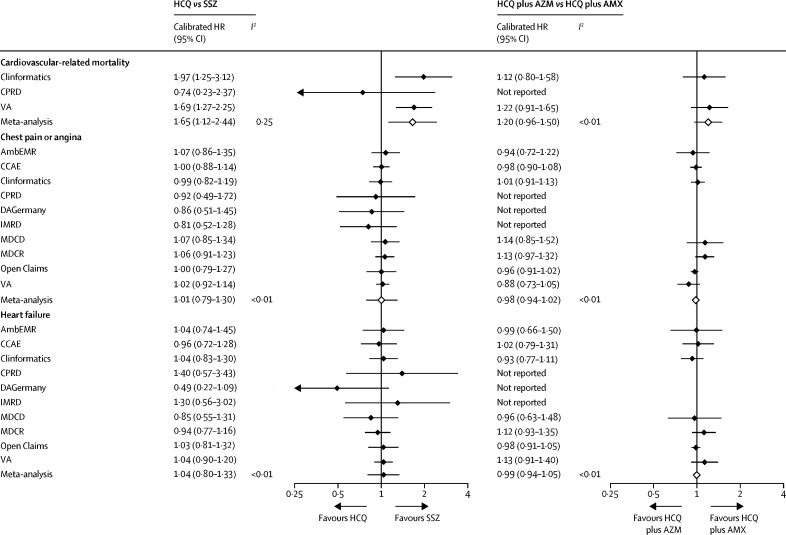
Source-specific and meta-analytic specific severe adverse event risk estimates for HCQ versus SSZ and HCQ plus AZM versus HCQ plus AMX new users during long-term (on-treatment) follow-up AmbEMR=IQVIA Ambulatory EMR. AMX=amoxicillin. AZM=azithromycin. CCAE=IBM Commercial Claims and Encounters. CPRD=Clinical Practice Research Datalink. DAGermany=IQVIA Disease Analyzer Germany. EMR=electronic medical record. HCQ=hydroxychloroquine. HR=hazard ratio. IMRD=IQVIA UK Integrated Medical Record Data. MDCD=IBM Multi-state Medicaid. MDCR=IBM Medicare Supplemental Database. OptumEHR=Optum de-identified Electronic Health Record. SSZ=sulfasalazine. VA=US Department of Veterans Affairs.

Similar findings were seen with the long-term (on-treatment) use of hydroxychloroquine versus sulfasalazine (figure 1; figure 3), with the exception of cardiovascular mortality, which appeared to be inconsistent in the available databases but increased overall in the hydroxychloroquine group when meta-analysed (pooled calibrated HR 1·65 [95% CI 1·12–2·44]).

Self-controlled case series analyses supported the findings of the main analysis, while looking at the effect of hydroxychloroquine use (on treatment *vs* off treatment) regardless of indication, and therefore including patients without rheumatoid arthritis (table 4; full results are given in the appendix pp 119–25).

**Table 4:** Summary of self-controlled case series results for HCQ

	**CCAE**		**Clinformatics**		**CPRD**		**JMDC Claims Database**		**MDCD**		**MDCR**		**VA**	
	Calibrated incidence rate ratio	Calibrated 95% CI	Calibrated incidence rate ratio	Calibrated 95% CI	Calibrated incidence rate ratio	Calibrated 95% CI	Calibrated incidence rate ratio	Calibrated 95% CI	Calibrated incidence rate ratio	Calibrated 95% CI	Calibrated incidence rate ratio	Calibrated 95% CI	Calibrated incidence rate ratio	Calibrated 95% CI
**Myocardial infarction**														
Adjusted analysis*	0·91	0·69–1·21	1·11	0·81–1·54	NA	NA	NA	NA	NA	NA	1·09	0·86–1·39	0·99	0·73–1·34
Primary analysis	0·92	0·70–1·22	1·02	0·74–1·40	NA	NA	NA	NA	0·89	0·70–1·15	0·99	0·78–1·26	1·00	0·74–1·36
**Acute pancreatitis events**														
Adjusted analysis*	NA	NA	1·05	0·75–1·46	NA	NA	2·18	0·11–43·82	1·13	0·86–1·47	1·03	0·77–1·36	0·97	0·71–1·34
Primary analysis	0·90	0·68–1·20	1·05	0·75–1·46	NA	NA	2·11	0·14–31·50	1·12	0·86–1·46	0·99	0·74–1·32	0·98	0·71–1·35
**Acute renal failure**														
Adjusted analysis*	0·88	0·67–1·16	0·96	0·58–1·59	NA	NA	1·33	0·31–5·71	NA	NA	1·08	0·85–1·37	NA	NA
Primary analysis	0·90	0·69–1·19	0·99	0·72–1·37	NA	NA	1·39	0·32–6·12	1	0·80–1·25	0·97	0·76–1·23	1·11	0·83–1·50
**Gastrointestinal bleeding**														
Adjusted analysis*	NA	NA	1·13	0·82–1·55	NA	NA	0·25	0·03–2·44	0·95	0·75–1·20	1·02	0·81–1·30	NA	NA
Primary analysis	1·01	0·76–1·32	1·06	0·77–1·46	NA	NA	0·24	0·03–2·38	0·95	0·76–1·20	0·96	0·76–1·22	0·97	0·72–1·32
**Cardiac arrhythmia**														
Adjusted analysis*	0·95	0·72–1·25	1·03	0·74–1·42	0·95	0·61–1·47	0·62	0·18–2·15	0·93	0·74–1·17	0·85	0·67–1·09	0·86	0·64–1·17
Primary analysis	0·95	0·72–1·26	1·03	0·74–1·43	0·95	0·61–1·48	0·58	0·17–1·98	0·93	0·74–1·17	0·86	0·67–1·10	0·85	0·63–1·15
**Bradycardia**														
Adjusted analysis*	NA	NA	0·91	0·65–1·27	0·65	0·20–2·16	3·67	0·26–50·91	NA	NA	0·87	0·68–1·12	0·88	0·65–1·20
Primary analysis	0·72	0·54–0·96	0·92	0·67–1·28	0·68	0·21–2·18	3·69	0·26–51·54	0·74	0·55–0·99	0·87	0·68–1·12	0·93	0·68–1·26
**Chest pain or angina**														
Adjusted analysis*	0·91	0·69–1·21	1·07	0·77–1·48	0·98	0·63–1·52	0·92	0·45–1·85	1·07	0·84–1·38	0·95	0·75–1·21	NA	NA
Primary analysis	0·91	0·69–1·21	1·06	0·76–1·47	0·98	0·63–1·52	0·91	0·45–1·84	1·07	0·84–1·36	0·94	0·74–1·20	0·98	0·73–1·33
**End-stage renal disease**														
Adjusted analysis*	1·02	0·69–1·51	NA	NA	0·91	0·15–5·49	NA	NA	NA	NA	0·88	0·66–1·18	1·04	0·76–1·44
Primary analysis	1·03	0·76–1·39	1·26	0·90–1·76	0·91	0·15–5·31	NA	NA	1·24	0·93–1·64	0·88	0·66–1·19	1·02	0·74–1·40
**Heart failure**														
Adjusted analysis*	0·99	0·75–1·29	1·15	0·83–1·58	1·20	0·69–2·09	1·02	0·50–2·10	0·95	0·75–1·20	1·12	0·88–1·42	1·03	0·76–1·39
Primary analysis	0·99	0·75–1·30	1·13	0·82–1·56	1·21	0·69–2·11	1·02	0·49–2·08	0·95	0·75–1·20	1·09	0·86–1·39	1·04	0·77–1·40
**Hepatic failure**														
Adjusted analysis*	0·68	0·50–0·92	NA	NA	NA	NA	1·54	0·08–30·15	0·83	0·60–1·16	0·82	0·58–1·17	NA	NA
Primary analysis	0·64	0·47–0·88	0·73	0·52–1·02	0·09	0·01–1·35	1·48	0·07–33·23	0·77	0·55–1·07	0·81	0·57–1·15	0·79	0·56–1·11
**Stroke**														
Adjusted analysis*	NA	NA	0·97	0·70–1·34	NA	NA	1·13	0·36–3·55	0·90	0·71–1·14	1·01	0·80–1·29	0·96	0·71–1·31
Primary analysis	0·80	0·61–1·06	0·90	0·65–1·24	NA	NA	1·14	0·36–3·59	0·85	0·67–1·08	0·93	0·73–1·18	0·98	0·72–1·34
**Cardiovascular events**														
Adjusted analysis*	NA	NA	0·90	0·37–2·21	NA	NA	0·51	0·21–1·25	0·91	0·73–1·15	1·10	0·87–1·40	NA	NA
Primary analysis	0·86	0·66–1·14	0·95	0·69–1·31	NA	NA	0·50	0·20–1·25	0·86	0·68–1·08	1·02	0·80–1·29	1·08	0·80–1·46
**Transient ischaemic attack**														
Adjusted analysis*	0·91	0·69–1·20	0·94	0·68–1·30	NA	NA	NA	NA	0·94	0·72–1·23	1	0·79–1·28	NA	NA
Primary analysis	0·92	0·70–1·21	0·93	0·68–1·29	NA	NA	NA	NA	0·92	0·71–1·20	0·97	0·76–1·24	1·20	0·88–1·65
**Venous thromboembolism**														
Adjusted analysis*	0·79	0·54–1·15	0·86	0·62–1·18	0·70	0·45–1·09	1·51	0·62–3·67	0·88	0·71–1·10	0·76	0·60–0·96	NA	NA
Primary analysis	0·81	0·62–1·07	0·84	0·61–1·16	0·69	0·44–1·07	1·51	0·62–3·67	0·87	0·70–1·09	0·71	0·56–0·91	0·86	0·64–1·16

CCAE=IBM Commercial Claims and Encounters. CPRD=Clinical Practice Research Datalink. HCQ=hydroxychloroquine. JMDC=Japanese Medical Data Center. MDCD=IBM Multi-state Medicaid. MDCR=IBM Medicare Supplemental Database. NA=non-applicable (not reported because of failed diagnostics or on-treatment follow-up unavailable). VA=US Department of Veterans Affairs.

* Adjusted for event-dependent observation.

All of the obtained database-specific and outcome-specific calibrated HRs for the association between short-term (on-treatment) use of hydroxychloroquine plus azithromycin versus hydroxychloroquine plus amoxicillin are depicted as forest plots in the appendix (pp 72–103). Three severe adverse events appeared to be increased with the short-term (30-day intention to treat) use of hydroxychloroquine plus azithromycin compared with hydroxychloroquine plus amoxicillin: chest pain or angina (meta-analytic calibrated HR 1·15 [95% CI 1·05–1·26]), heart failure (1·22 [1·02–1·45]), and cardiovascular mortality (2·19 [1·22–3·95]; figure 2).

Full results from each dataset, including power, attrition, and population characteristics are available online. This site also contains all of the cohort diagnostic tools that were examined before unblinding results and before a dataset was included in the meta-analyses. Each dataset was examined for the risk of observed confounding (within the propensity score model, propensity score distribution, and covariate balance with identified variables) or by unobserved confounding (assessing negative control variables within analysis of the risk of systematic error) before their inclusion. These diagnostic tools can be reviewed for each database for each outcome within the shiny application of R (version 3.61) in order to give full transparency of analysis.

## Discussion

To our knowledge, this study is the largest ever analysis of the safety of hydroxychloroquine and hydroxychloroquine plus azithromycin worldwide, examining more than 950 000 hydroxychloroquine and more than 300 000 hydroxychloroquine plus azithromycin users, respectively. Short-term (up to 30 days) hydroxychloroquine treatment among patients with rheumatoid arthritis showed no excess risk of any of the considered severe adverse events compared with sulfasalazine. Short-term treatment is also proposed for COVID-19 therapy and might be informed by the experience of treatment in patients with rheumatoid arthritis. By comparison, long-term hydroxychloroquine therapy appears to be associated with a relative risk increase in cardiovascular-related mortality compared with a roughly equivalent rheumatoid arthritis therapy (sulfasalazine; calibrated HR 1·65 [95% CI 1·12–2·44]). Perhaps more worryingly, compared with hydroxychloroquine plus amoxicillin, significant risks were identified for the combination of hydroxychloroquine plus azithromycin even in the short term: increased risk of angina or chest pain (calibrated HR 1·15 [95% CI 1·05–1·26]) and heart failure (1·22 [1·02–1·45]), and a doubled risk of cardiovascular mortality in the first month of treatment (2·19 [1·22–3·94]).

A systematic review of reports on the toxicity of hydroxychloroquine has identified cardiac side-effects, including conduction disorders, heart failure, and ventricular hypertrophy resulting in 12·9% irreversible damage and 30% mortality.^19^, ^20^ Furthermore, interrogation of the US Food and Drug Administration Adverse Event Reporting System database identified 357 adverse events reported for chloroquine.^18^ 20% of the events reported were cardiac and included arrhythmia, sudden cardiac death, or heart failure.

Our results suggest that long-term use of hydroxychloroquine leads to increased cardiovascular mortality, which might relate to cumulative effects of hydroxychloroquine leading to an increased risk of QT lengthening and potentially to sudden undetected torsade-de-pointes and cardiovascular death. Although long-term treatment with hydroxychloroquine is not expected for the management of COVID-19, some research suggests that the higher doses prescribed for COVID-19 than for rheumatoid arthritis can, even in the short term, lead to equivalent side-effects given the long half-life of hydroxychloroquine.^19^

In addition, QT lengthening is a known side-effect of all macrolides, including azithromycin, and physicians already use caution when prescribing macrolides concurrently with other medications that can interact to increase the QT interval.^22^, ^23^ In this study, a relative risk of 2·19 (95% CI 1·22–3·94) for cardiovascular death was seen even with short-term hydroxychloroquine plus azithromycin combination therapy, probably arising through their synergistic effects on QT length and subsequent induction of lethal arrhythmia. Considering that hydroxychloroquine and azithromycin are both contraindicated for use in patients with cardiac arrhythmias, this study assumes that clinicians are prescribing these medications for patients as per existing labelling advice. It is therefore concerning that cardiovascular effects were still seen in our study populations, possibly indicating that the true risks of these drugs are understated in the analysis.

It is important to identify potential sources of bias that could limit the study. The analyses are predicated on observing the presence of exposure, outcomes, and covariates in the data, or inferring their absence based on an assumption of complete data capture during a defined observation period during which a person is not expected to be lost to follow-up. In this regard, although there were no missing data that required imputation, each binary variable is subject to potential misclassification error, and the sensitivity and specificity of these variables in each database are unknown. Because of the nature of sudden cardiac death, capturing the true cause of cardiovascular-related mortality is difficult. Although we examined various aspects of cardiac complications as captured by diagnosis codes, the accuracy of evaluations of QT prolongation, ventricular tachycardia, or other arrhythmias would probably be improved with precise electrocardiogram measurements. Exposure misclassification can occur as a result of non-adherence or non-compliance with either treatment and thus could bias the results in either direction, and outcome misclassification might exist because of incomplete or incorrect recording of severe adverse events. Baseline covariates might also be subject to measurement error and, although observing balance on all baseline characteristics after propensity score adjustment provides reassurance that the risk of confounding has been reduced, there remains potential for confounding in any given source for differential misclassification. The consistency of findings across heterogeneous patient populations with disparate data capture processes mitigates this concern. Within the study design, use of routine health-care data in populations across four continents, and including all adults with rheumatoid arthritis was used to minimise selection bias. The self-controlled case series analysis was also added to investigate all users of hydroxychloroquine as an external validation of the hydroxychloroquine findings in the rheumatoid arthritis population via the new user design. To investigate systematic error, study diagnostics were evaluated before unblinding results through interrogation of negative controls.

We have taken into consideration that patients with rheumatoid arthritis taking hydroxychloroquine might also have further autoimmune conditions such as SLE and therefore generate the potential for confounding by indication. We also investigated the incidence of hyperlipidaemia, diabetes, venous thromboembolic disease, and coronary arteriosclerosis before unblinding because of the established evidence that hydroxychloroquine improves survival in patients with SLE through antilipidaemic and antithrombotic mechanisms of action and reduces the development of diabetes in patients with SLE and those with rheumatoid arthritis.^28^, ^37^, ^38^, ^39^ We ensured that, when investigating covariate balance after propensity score stratification and matching and before unblinding study results, we did not see unbalanced proportions of patients with a diagnosis of SLE between the groups. Negative control outcome analyses to assess for systematic error also did not identify any residual unobserved confounding in the propensity score analysis, adjusting for thousands of variables within the large-scale propensity score model. Although we have balanced for the coexistence of other conditions and medications through propensity scores, and we tested for residual unobserved confounding to ensure groups were balanced, no direct measure of severity of rheumatoid arthritis was drawn for patients at baseline. The cohort was made from patients who were new users of both hydroxychloroquine and sulfasalazine with a diagnosis of rheumatoid arthritis and without medication use in the previous 365 days, but the potential for differences in baseline rheumatoid arthritis severity not recorded in routinely collected data is also a limitation of the study.

Another criticism is the choice of sulfasalazine as an active comparator. Both hydroxychloroquine and sulfasalazine are second-line conventional synthetic disease-modifying antirheumatic drugs in the treatment of patients with rheumatoid arthritis, used in addition to, or instead of methotrexate. Although they are not fully equivalent to each other, and no drug can be an exact match, they are each the closest comparator treatment to the other. Appreciating they are not truly equivalent, we took care to ensure that propensity score stratification and negative control analysis for any systematic error ensured that the two groups were as balanced as possible to minimise confounding.

Another potential limitation in this study is the potential for patients to be included in more than one dataset in the USA. Although we ran meta-analyses, which assume populations are independent, we highlight that we are likely to have underestimated variance in our meta-analytic estimates. We also acknowledge the limitation that although 14 databases were used in total, mortality analysis was restricted to databases with good coverage of this outcome (ie CPRD, IMRD, IPCI, VA, and Clinformatics). Similarly, as we do not know the baseline risk of serious adverse events within this population, we cannot report absolute risk of these events in patients with rheumatoid arthritis, and this limitation must be acknowledged.

In this large-scale, international, real-world data network study, hydroxychloroquine appears to be largely safe for short-term use in patients with rheumatoid arthritis compared with sulfasalazine, but when used in combination with azithromycin, this therapy carries a relative risk of 2·19 for cardiovascular death compared with hydroxychloroquine combined with amoxicillin. The collective experience of almost a million patients builds our confidence in the evidence around the safety profile of hydroxychloroquine. In line with consensus expert guidance, our findings suggest that a cautious assessment of cardiovascular risk is needed before initiating high-dose hydroxychloroquine or hydroxychloroquine plus azithromycin combination therapy, and in long-term monitoring of patients with rheumatoid arthritis, especially those with cardiovascular risk factors.^8^

## Data sharing

Open science is a guiding principle within Observational Health Data Sciences and Informatics. As such, we provide unfettered access to all open-source analysis tools used in this study via https://github.com/OHDSI/, as well as all data and results artefacts that do not include patient-level health information via http://evidence.ohdsi.org/Covid19EstimationHydroxychloroquine. Data partners contributing to this study remain custodians of their individual patient-level health information and hold either exemption from institutional review boards or approval for participation. All ethical approvals can be found in the appendix (p 130).
