# Supplementary material for: Risk of hydroxychloroquine alone and in combination with azithromycin in the treatment of rheumatoid arthritis: a multinational, retrospective study
Source: Lancet Rheumatol. 2020 Aug 21;2(11):e698–711. doi: 10.1016/S2665-9913(20)30276-9 (PMC7442425; doi:10.1016/S2665-9913(20)30276-9)
Supplement: Supplementary appendix [file mmc1.pdf]

THE LANCET
Rheumatology

Supplementary appendix

This appendix formed part of the original submission and has been peer reviewed. We post it as supplied by the authors.

Supplement to: Lane JCE, Weaver J, Kostka K, et al. Risk of hydroxychloroquine alone and in combination with azithromycin in the treatment of rheumatoid arthritis: a multinational, retrospective study. *Lancet Rheumatol* 2020; published online Aug 21. https://doi.org/10.1016/S2665-9913(20)30276-9.

Supplemental Appendix to *Risk of hydroxychloroquine alone and in combination with azithromycin in the treatment of rheumatoid arthritis: a multi-national, retrospective study*

Jennifer C.E. Lane, James Weaver, Kristin Kostka, Talita Duarte-Salles, Maria Tereza F. Abrahao, Heba Alghoul, Osaid Alser, Thamir M Alshammari, Patricia Biedermann, Juan M. Banda, Edward Burn, Paula Casajust, Mitch Conover, Aedin C. Culhane. Alexander Davydov, Scott L. DuVall, Dmitry Dymshyts, Sergio Fernandez-Bertolin, Kristina Fišter, Jill Hardin, Laura Hester, George Hripcsak, Seamus Kent, Sajan Khosla, Spyros Kolovos, Christophe G. Lambert, Johan van der Lei, Kristine E. Lynch, Rupa Makadia, Andrea V. Margulis, Michael E. Matheny, Paras Mehta, Daniel R. Morales, Henry Morgan-Stewart, Mees Mosseveldl, Danielle Newby, Fredrik Nyberg, Anna Ostropolets, Rae Woong Park, Albert Prats-Uribe, Junqing Xie, Gowtham A. Rao, Christian Reich, Jenna Reps, Peter Rijnbeek, Selva Muthu Kumaran Sathappan, Martijn Schuemie, Sarah Seager, Anthony Sena, Azza Shoaibi, Matthew Spotnitz, Marc A. Suchard, Carmen O. Torre, David Vizcaya, Haini Wen, Marcel de Wilde, Seng Chan You, Lin Zhang, Oleg Zhuk, Patrick Ryan, and Daniel Prieto-Alhambra

All artefacts generated as part of this study are publicly available in an interactive, web-based application at: http://evidence.ohdsi.org/Covid19EstimationHydroxychloroquine

Contents

S1. Data sources 3
S2. Cohort definitions 5
  S2.1. Exposures 5
    S2.1.1. New users of Hydroxychloroquine with prior rheumatoid arthritis 5
    S2.1.2. New users of sulfasazine with prior rheumatoid arthritis 6
    S2.1.3. Users of Hydroxychloroquine + Azithromycin 7
    S2.1.4. Users of Hydroxychloroquine + Amoxicillin 8
  S2.2. Outcomes 8
S3. Negative control outcomes 10
S4. Covariate construction 11
S5. Exposure cohort counts 12
S6. Covariate balance before and after PS stratification 13
  S6.1. HCQ vs SSZ 13
    S6.1.1. AmbEMR 13
    S6.1.2. CCAE 15
    S6.1.3. CPRD 17
    S6.1.4. Clinformatics 19
    S6.1.5. DAGermany 21
    S6.1.6. IMRD 23
    S6.1.7. IPCI 25
    S6.1.8. JMDC 27
    S6.1.9. MDCD 29
    S6.1.10. MDCR 31
    S6.1.11. OpenClaims 33
    S6.1.12. OptumEHR 35
    S6.1.13. SIDIAP 37
    S6.1.14. VA 39
  S6.2. AZM vs AMX 41
    S6.2.1. AmbEMR 41
    S6.2.2. CCAE 43
    S6.2.3. Clinformatics 45
    S6.2.4. CPRD 47
    S6.2.5. DAGermany 49
    S6.2.6. IMRD 51
    S6.2.8. MDCD 54
    S6.2.9. MDCR 56
    S6.2.10. OpenClaims 58
    S6.2.11. OptumEHR 60
    S6.2.12. SIDIAP 62
    S6.2.13. VA 64
S7. Patient counts, events, incident rates 66
S8. Calibrated hazard ratio forest plots 72
  S8.1. 30-day follow-up 72
    S8.1.1. All-cause mortality 72
    S8.1.2. CV-related mortality 73

S8.1.3. Myocardial infarction 74
S8.1.4. Chest pain or angina 75
S8.1.5. Heart failure 76
S8.1.6. Cardiovascular events 77
S8.1.7. Cardiac arrhythmia 78
S8.1.8. Bradycardia 79
S8.1.9. Transient ischemic attack 80
S8.1.10. Stroke 81
S8.1.11. Venous thromboembolism 82
S8.1.12. Gastrointestinal bleeding 83
S8.1.13. Acute renal failure 84
S8.1.14. End stage renal disease 85
S8.1.15. Hepatic failure 86
S8.1.16. Acute pancreatitis 87
S8.2. On-treatment follow-up 88
S8.2.1. All-cause mortality 88
S8.2.2. CV-related mortality 89
S8.2.3. Myocardial infarction 90
S8.2.4. Chest pain or angina 91
S8.2.5. Heart failure 92
S8.2.6. Cardiovascular events 93
S8.2.7. Cardiac arrhythmia 94
S8.2.8. Bradycardia 95
S8.2.9. Transient ischemic attack 96
S8.2.10. Stroke 97
S8.2.11. Venous thromboembolism 98
S8.2.12. Gastrointestinal bleeding 99
S8.2.13. Acute renal failure 100
S8.2.14. End stage renal disease 101
S8.2.15. Hepatic failure 102
S8.2.16. Acute pancreatitis 103
S9. Evidence evaluation diagnostics 104
S9.1. AmbEMR 105
S9.2. CCAE 106
S9.3. Clinformatics 107
S9.4. CPRD 108
S9.5. DAGermany 109
S9.6. IMRD 110
S9.7. IPCI 111
S9.8. JMDC 112
S9.9. MDCD 113
S9.10. MDCR 114
S9.11. OpenClaims 115
S9.12. OptumEHR 116
S9.13. SIDIAP 117
S9.14. VA 118
S10. Self-controlled case series results for hydroxychloroquine 119
S10.1. CCAE 119
S10.2. Clinformatics 120
S10.3. CPRD 121
S10.4. JMDC 122
S10.5. MDCD 123
S10.6. MDCR 124
S10.7. VA 125
S11. Literature review sources 126
S11.1. PubMed search strategy 126
S11.2. Embase search strategy (1974 to present) 127
S11.3. ClinicalTrials.gov search strategy 129
S11.4. ICTRP strategy 129
S12.Ethical Approval 130
S13.References 131

S1. Data sources

Database name	Abbreviation	Population	Patients (millions)	Data History	Data capture process and short database description
Department of Veterans Affairs	VA	USA (Veteran population)	~12M	2000 –	VA OMOP data reflects the national Department of Veterans Affairs health care system, which is the largest integrated provider of medical and mental health services in the United States. Care is provided at 170 VA Medical Centers and 1,063 outpatient sites serving more than 9 million enrolled Veterans each year.
IQVIA Disease Analyzer Germany	DAGermany	Germany (General population)	37M	1992 –	Anonymized patient records collected from Patient Management software used by general practitioners and selected specialists to document patients' medical records within their office-based practice during a visit.
IQVIA UK Integrated Medical Record Data	IMRD	UK (General population)	15M	1989 –	Pseudonymized Electronic Medical Records collected from patient management software used within UK Primary Care
IQVIA US Ambulatory EMR	AmbEMR	USA (General population)	49M	2006 –	General practice EHR, Outpatient specialist EHR - Dataset consists of longitudinal, de-identified ambulatory electronic health records data
IQVIA US LRxDx Open Claims	OpenClaims	USA (General population)	654M	2010 –	Pre-adjudicated claims at the anonymized patient level collected from office-based physicians and specialists via office management software and clearinghouse switch sources for the purpose of reimbursement.
Clinical Practice Research Datalink	CPRD	UK (General population)	13M	1995 –	De-identified patient data from a network of general practitioners' practices across the UK. Primary care data are linked to a range of other health related data to provide a longitudinal, representative UK population health dataset.
IBM MarketScan Commercial Claims	CCAE	USA (Patients with commercial insurance aged <65 years)	142M	2000 –	Data from individuals enrolled in US employer-sponsored insurance health plans. The data includes adjudicated health insurance claims (e.g. inpatient, outpatient, and outpatient pharmacy) as well as enrollment data from large employers and health plans who provide private healthcare coverage to employees, their spouses,and dependents. Additionally, it captures laboratory tests for a subset of the covered lives.
IBM MarketScan Multi-State Medicaid Database	MDCD	USA	26M	2006 –	Adjudicated US health insurance claims for Medicaid enrollees from multiple states and includes hospital discharge diagnoses, outpatient diagnoses and procedures, and outpatient pharmacy claims as well as ethnicity and Medicare eligibility.
IBM MarketScan Medicare Supplemental Database	MDCR	USA (Patients with commercial insurance aged 65+ years)	10M	2000 –	Represents health services of retirees (aged 65 or older) in the United States with primary or Medicare supplemental coverage through privately insured fee-for-service, point-of-service, or capitated health plans. These data include adjudicated health insurance claims (e.g. inpatient, outpatient, and outpatient pharmacy). Additionally, it captures laboratory tests for a subset of the covered lives.
Integrated Primary Care Information	IPCI	Netherlands (General Population)	2.5M	1996-	Complete medical records collected from 450 general practitioners with geographical representation of the country.
Japan Medical Data Center	JMDC	Japan (insured general population)	5.5M	2005 –	Data from 60 Society-Managed Health Insurance plans covering workers aged 18 to 65 and their dependents (children younger than 18 years old and elderly people older than 65 years old). JMDC data includes membership status of the insured people and claims data provided byinsurers under contract (e.g. patient-level demographic information, inpatient and outpatient data inclusive of diagnosis and procedures, and prescriptions as dispensed claims information)
Optum de-identified Clinformatics® Data Mart Database	Clinformatics	USA (Patients with commercial insurance or commercial Medicare insurance)	85M	2000 -	Inpatient and outpatient healthcare insurance claims

S1, continued.

Database name	Abbreviation	Population	Patients (millions)	Data History	Data capture process and short database description
Optum® de-identified Electronic Health Record Dataset	OptumEHR	USA (General population)	93M	2006 –	Optum's de-identified electronic health record data medical records database. The medical record data includes clinical information, inclusive of prescriptions as prescribed and administered, lab results, vital signs, body measurements, diagnoses, procedures, and information derived from clinical notes using Natural Language Processing (NLP).
The Information System for Research in Primary Care	SIDIAP	Spain-Catalonia (80% of general population)	7.7M	2006 –	Electronic health records from primary care partially linked to inpatient data. SIDIAP is also linked to a pharmacy dispensations and primary care laboratories. Healthcare is universal and taxpayer funded in the region, and primary care physicians are gatekeepers for all care and responsible for repeat prescriptions.

S2. Cohort definitions

S2.1. Exposures

In the HCQ versus SSZ study, the index event was defined as the first recorded dispensing or prescription of the drug in a patient's history. For the study of HCQ combined with AZM, follow up started when the second of the two co-administered treatments was initiated while still exposed to the first treatment (e.g. when AZM started during a period of HCQ use, or when HCQ started during a period of AZM use). HCQ use was assumed to be chronic in the management of RA, and AZM was assumed an acute prescription for infection treatment, and therefore inferred persistent exposure to AZM was assessed by allowing up to 30 days between dispensing or prescription records. Cohorts of combined HCQ and amoxicillin were generated using these same rules as an active comparator.

Exposure commenced on the first day of dispensing or prescription recorded with at least 365 days of prior observation period to increase confidence that the exposure was incident. Exposure interval gaps of ≤90 days (HCQ and SSZ) and of ≤30 days (AZM and AMX) between drug dispensing or prescription records were allowed and inferred as persistent exposure. Drug discontinuation was considered in the HCQ study if a patient switched from one study drug to another. Patients who switched from target exposure to comparator exposure, or vice versa, contributed follow-up time to the exposure cohort that they entered first, and were censored at the time of switching in the 'on treatment' analysis.

To view the concept sets and source code lists (e.g. NDC) associated with each outcome, navigate to the URL associated with each exposure cohort name. Then, navigate to the *Concept Sets* tab. The concept sets used in defining the outcome are listed on this page. After selecting a concept set to view, a user can explore the standard concepts used in the definition or the source codes from which the standard concepts are mapped.

S2.1.1. New users of Hydroxychloroquine with prior rheumatoid arthritis

https://atlas.ohdsi.org/#/cohortdefinition/173

Initial Event Cohort
People having any of the following:

- a drug exposure of [OHDSI Cov19] Hydroxychloroquine[1]
    - for the first time in the person's history
    - with age >= 18

with continuous observation of at least 365 days prior and 0 days after event index date, and limit initial events to: **earliest event per person.**

Inclusion Rules
Inclusion Criteria #1: has rheumatoid arthritis recorded any time on or prior to treatment
Having any of the following criteria:

- at least 1 occurrence of a condition occurrence of [OHDSI Cov19] Rheumatoid arthritis[2] where event starts between all days Before and 0 days After index start date
- or at least 1 occurrence of an observation of [OHDSI Cov19] Rheumatoid arthritis[2] where event starts between all days Before and 0 days After index start date

Limit qualifying cohort to: **earliest event per person.**

End Date Strategy
Custom Drug Era Exit Criteria
This strategy creates a drug era from the codes found in the specified concept set. If the index event is found within an era, the cohort end date will use the era's end date. Otherwise, it will use the observation period end date that contains the index event.
Use the era end date of [OHDSI Cov19] Hydroxychloroquine[1]

- allowing 90 days between exposures
- adding 0 days after exposure end

Cohort Collapse Strategy:
Collapse cohort by era with a gap size of 0 days.

S2.1.2. New users of sulfasazine with prior rheumatoid arthritis

https://atlas.ohdsi.org/#/cohortdefinition/45

Initial Event Cohort
People having any of the following:

- a drug exposure of [OHDSI Cov19] sulfasalazine[2]
    - for the first time in the person's history

with continuous observation of at least 365 days prior and 0 days after event index date, and limit initial events to: **earliest event per person.**

Inclusion Rules
Inclusion Criteria #1: has rheumatoid arthritis recorded any time on or prior to treatment
Having all of the following criteria:

- at least 1 occurrence of a condition occurrence of [OHDSI Cov19] Rheumatoid arthritis[1]
  where event starts between all days Before and 0 days After index start date

Limit qualifying cohort to: **earliest event per person.**

End Date Strategy
Custom Drug Era Exit Criteria
This strategy creates a drug era from the codes found in the specified concept set. If the index event is found within an era, the cohort end date will use the era's end date. Otherwise, it will use the observation period end date that contains the index event.
Use the era end date of [OHDSI Cov19] sulfasalazine[2]

- allowing 90 days between exposures
- adding 0 days after exposure end

Cohort Collapse Strategy:
Collapse cohort by era with a gap size of 0 days.

S2.1.3. Users of Hydroxychloroquine + Azithromycin

https://atlas.ohdsi.org/#/cohortdefinition/170

Initial Event Cohort
People having any of the following:

- a drug exposure of Hydroxychloroquine[1]
  - with age >= 18
- a drug exposure of [OHDSI Covid19] Azithromycin[3]
  - with age >= 18

with continuous observation of at least 365 days prior and 0 days after event index date, and limit initial events to: **all events per person.**

Inclusion Rules
Inclusion Criteria #1: has both drugs on index
Having all of the following criteria:

- at least 1 occurrence of a drug exposure of Hydroxychloroquine[1]
  where event starts between 30 days Before and 0 days After index start date
- and at least 1 occurrence of a drug exposure of [OHDSI Covid19] Azithromycin[3]
  where event starts between 30 days Before and 0 days After index start date

Inclusion Criteria #2: prior diagnosis of RA
Having any of the following criteria:

- at least 1 occurrence of a condition occurrence of [OHDSI Cov19] Rheumatoid arthritis[2]
  where event starts between all days Before and 0 days After index start date
- or at least 1 occurrence of an observation of [OHDSI Cov19] Rheumatoid arthritis[2]
  where event starts between all days Before and 0 days After index start date

Limit qualifying cohort to: **earliest event per person.**

End Date Strategy
Custom Drug Era Exit Criteria
This strategy creates a drug era from the codes found in the specified concept set. If the index event is found within an era, the cohort end date will use the era's end date. Otherwise, it will use the observation period end date that contains the index event.
Use the era end date of [OHDSI Covid19] Azithromycin[3]

- allowing 30 days between exposures
- adding 0 days after exposure end

Cohort Collapse Strategy:
Collapse cohort by era with a gap size of 0 days.

S2.1.4. Users of Hydroxychloroquine + Amoxicillin
https://atlas.ohdsi.org/#/cohortdefinition/171

Initial Event Cohort
People having any of the following:
- a drug exposure of Hydroxychloroquine[1]
  - with age >= 18
- a drug exposure of [OHDSI Covid19] Amoxicillin[3]
  - with age >= 18

with continuous observation of at least 365 days prior and 0 days after event index date, and limit initial events to: **all events per person.**

Inclusion Rules
Inclusion Criteria #1: has both drugs on index
Having all of the following criteria:
- at least 1 occurrences of a drug exposure of Hydroxychloroquine[1]
  where event starts between 30 days Before and 0 days After index start date
- and at least 1 occurrences of a drug exposure of [OHDSI Covid19] Amoxicillin[3]
  where event starts between 30 days Before and 0 days After index start date

Inclusion Criteria #2: Prior diagnosis of RA
Having any of the following criteria:
- at least 1 occurrences of a condition occurrence of [OHDSI Cov19] Rheumatoid arthritis[2]
  where event starts between all days Before and 0 days After index start date
- or at least 1 occurrences of an observation of [OHDSI Cov19] Rheumatoid arthritis[2]
  where event starts between all days Before and 0 days After index start date

Limit qualifying cohort to: **earliest event per person.**

End Date Strategy
Custom Drug Era Exit Criteria
This strategy creates a drug era from the codes found in the specified concept set. If the index event is found within an era, the cohort end date will use the era's end date. Otherwise, it will use the observation period end date that contains the index event.
Use the era end date of [OHDSI Covid19] Amoxicillin[3]
- allowing 30 days between exposures
- adding 0 days after exposure end

Cohort Collapse Strategy:
Collapse cohort by era with a gap size of 0 days.

S2.2. Outcomes
All safety outcomes for this study were defined as described in the supplementary appendix to Suchard *et al.* (2019).[1] Below is an abbreviated version of the safety outcome definition process from that publication.

For each outcome, we developed an operational phenotype definition to determine if observational data could support evaluation of the outcome. We used the same approach to design, implement, and evaluate all phenotypes. Specifically, we conducted a PubMed literature review to identify past observational studies that used the phenotype as an outcome, looking especially for studies where source record verification or other validation approaches were applied. In addition, we reviewed eMERGE PheKB phenotype entries (https://phekb.org/phenotypes). Clinical guidelines and systematic review of clinical trials of hypertension treatments informed our clinical definitions of cardiovascular outcomes. Where possible, concept sets originated with published code lists (e.g. ICD-9-CM and ICD-10). We augmented these with lexical search and semantic exploration of the OHDSI standardized vocabularies. A clinical adjudicator then reviewed the cohort definitions and associated concept sets. We developed concept definitions using ATLAS, the OHDSI open-source platform (https://github.com/OHDSI/atlas). Because the databases used in this study do not all consistently contain laboratory values, diagnosis records alone identified

outcomes. To assess consistency across data sources as well as general clinical reasonableness, we utilized these cohorts to characterize outcome incidence, stratifying by age decile, gender, and index year (in Suchard *et al.* (2019)). We did not perform source record verification or other validation methods.

To view the concept sets and source code lists (e.g. ICD-9-CM, ICD-10-CM) associated with each outcome, navigate to the URL embedded in the phenotype name. Then, navigate to the *Concept Sets* tab. The concept sets used in defining the outcome are listed on this page. After selecting a concept set to view, a user can explore the standard concepts used in the definition or the source codes from which the standard concepts are mapped.

Safety outcomes of Interest

Phenotype	Logical description	Supporting references
All-cause mortality	Death record of any type	3, 4, 5
Cardiovascular-related mortality	Death record with at least 1 cardiovascular-related condition record (myocardial infarction, ischemic stroke, intracranial hemorrhage, sudden cardiac death, hospitalization for heart failure) in 30 days prior to death	3
Myocardial infarction	Acute myocardial infarction condition record during an inpatient or ER visit; successive records with > 180-day gap are considered independent episodes	6, 7, 8, 3, 9, 10
Chest pain or angina	The first condition record of chest pain or angina	11
Heart failure	The first condition record of heart failure, which is followed by at least 1 heart failure condition record in the following year	7, 12, 13, 14, 15, 16, 17, 18, 19, 20, 21
Cardiovascular events	A condition record of ischemic stroke, hemorrhagic stroke, heart failure, acute myocardial infarction or sudden cardiac death during an inpatient or ER visit; successive records with > 180-day gap are considered independent episodes	6, 7, 8, 3, 9, 10, 22
Cardiac arrhythmia	The first condition record of cardiac arrhythmia, which is followed by another cardiac arrhythmia condition record, at least two drug records for a drug used to treat arrhythmias, or a procedure to treat arrhythmias	22, 23, 24, 25, 26, 27, 28
Bradycardia	The first condition record of bradycardia, which is followed by another bradycardia condition record	29, 30
Transient ischemic attack	Transient ischemic attack condition record during an inpatient or ER visit; successive records with > 30-day gap are considered independent episodes	31, 32
Stroke	Stroke (ischemic or hemorrhagic) condition record during an inpatient or ER visit; successive records with > 180-day gap are considered independent episodes	12, 3, 9, 24, 31, 33, 34, 35, 36, 32
Venous thromboembolism	Venous thromboembolism condition record of any type; successive records with > 180-day gap are considered independent episodes	37, 38, 39, 40
Gastrointestinal bleeding	Gastrointestinal hemorrhage condition record during an inpatient or ER visit; successive records with > 30-day gap are considered independent episodes	41, 9, 42, 43, 44, 45, 46
Acute renal failure	A diagnosis of acute renal failure in an inpatient or ER setting; must be at least 30d between inpatient/ER visits to be considered separate episodes	47, 48, 49, 50, 51, 52, 53, 54
End stage renal disease	End stage renal disease (ESRD) is defined by at least one diagnosis in any setting, followed by at least one additional diagnosis or a dialysis-related procedure within 90 days	55, 56, 57
Hepatic failure	The first condition record of hepatic failure, necrosis, or coma	47, 58, 59, 60, 61, 62, 63, 64, 65
Acute pancreatitis	Acute pancreatitis condition record during an inpatient or ER visit; successive records with >30-day gap are considered independent episodes	66, 67, 68, 55

S3. Negative control outcomes

Negative control outcomes are conditions believed to have no causal relationship with hydroxychloroquine, sulfasalazine, azithromycin, or amoxicillin exposure and therefore assumed *a priori* to return a hazard ratio (HR) of 1.0 when their risk is compared between exposure cohorts in a pairwise comparison. A HR of a negative control outcome that differs from 1.0 represents an estimate of the residual error of the analysis that was unaccounted for by the analytic specification such as propensity score adjustment. The distribution of residual error estimates from the negative control outcomes reflects a range of biases inherent to the analysis. The estimates on the negative control outcomes represents the empirical null distribution and we used it to compute hazard ratios and confidence intervals calibrated to reflect the observed residual error of the analysis. The negative controls were selected through a semi-automated process [2] and are listed below with the corresponding SNOMED concept ID.

Concept ID	Concept Name	Concept ID	Concept Name
378256	Abnormal reflex	435516	Lipoprotein deficiency disorder
4092879	Absent kidney	438808	Mammary duct ectasia
433753	Alcohol abuse	441553	Myoclonus
321689	Apnea	4119307	Neurogenic claudication
78200	Benign mammary dysplasia	4209423	Nicotine dependence
4195873	Breath smells unpleasant	438130	Opioid abuse
443792	Calculus of bile duct	313601	Oxygen supply absent
434327	Cannabis abuse	44782778	Pain disorder with psychological factor
197318	Cholesterolosis of gallbladder	4091513	Passing flatus
432303	Cocaine abuse	4022076	Patient dependence on care provider
439125	Complete trisomy 21 syndrome	439971	Poisoning by anticoagulant
433270	Cord entanglement without compression	46286594	Problem related to lifestyle
4311591	Cramp in limb	199876	Prolapse of female genital organs
441267	Cystic fibrosis	4049367	Psychologic conversion disorder
436233	Delayed milestone	440068	Psychosexual dysfunction
40486120	Delay in physiological development	436246	Reduced libido
439791	Emotional upset	73754	Restless legs
374801	Foreign body in ear	4168212	Restlessness and agitation
259995	Foreign body in orifice	138821	Seborrhea
196456	Gallstone	4198492	Shoulder joint unstable
4166231	Genetic predisposition	25518	Sickle cell trait
434164	Glycosuria	4176908	Snapping thumb syndrome
4163735	Hemochromatosis	4248728	Snoring
439871	Hemospermia	138278	Sprains and strains of joints and adjacent muscles
4058388	Hypertrophic scar	4008710	Stenosis due to any device, implant AND/OR graft
435522	Hypervitaminosis D	40479573	Stimulant abuse
443236	Hypnotic or anxiolytic dependence	40483172	Stimulant dependence
4098604	Hypomagnesemia	440233	Strain of supraspinatus muscle AND/OR tendon
435371	Hypothermia	4194160	Thyroid function tests abnormal
443447	Iatrogenic hypotension	4216708	Urgent desire for stool
374375	Impacted cerumen	4275889	Visual hallucinations
4344500	Impingement syndrome of shoulder region	4193634	Worried
440382	Learning difficulties		

S4. Covariate construction

The following consistently extracted set of baseline patient characteristics will be constructed for input in the PS model. From this large set of typically tens of thousands of covariates, key predictors of exposure classification will be selected for inclusion in the PS model. Note that not all data sources necessarily include data for all covariates. Covariates to be included:

- Demographics (age in 5-year bands, sex, race, ethnicity, index year, index month)
- All conditions occurrence records aggregated to SNOMED clinical finding level during the following lookback windows:
    - in 365 days prior to and including index date
    - in 30 days prior to and including index date
- All drug exposure records aggregated to RxNorm ingredient level and ATC classes during the following lookback windows:
    - in 365 days prior to and including index date
    - in 30 days prior to and including index date
    - persistent exposure that overlaps index date
- All procedure occurrence records during the following lookback windows:
    - in 365 days prior to and including index date
    - in 30 days prior to and including index date
- Measurements (including laboratories) within, above, and below normal range during the following lookback window:
    - in 365 days prior to and including index date
- Device exposure records during the following lookback windows:
    - in 365 days prior to and including index date
    - in 30 days prior to and including index date
- Comorbidity or risk scores including:
    - Charlson
    - DCSI
    - CHADS2
    - CHADS2VASc

S5. Exposure cohort counts

The exposure cohort counts are calculated after the following study design criteria have been applied:

- Duplicate patients in the hydroxychloroquine and sulfasalazine with prior RA cohorts are removed from the cohort they qualified for second (i.e. retained in the cohort they qualified for first)
- Duplicate patients in the hydroxychloroquine + azithromycin and hydroxychloroquine + amoxicillin with prior RA cohorts are removed from the cohort they qualified for second (i.e. retained in the cohort they qualified for first)
- Restricted to the calendar time when both exposures in a pairwise comparison (i.e. HCQ vs SSA and HCQ+AZM vs HCQ+AMX) are observed in the database.

After applying these design criteria to the original cohorts, the resulting patients are eligible to contribute data to study diagnostics and potentially to final population-level effect estimates.

Exposure cohort	Database	Patients	Exposure cohort	Database	Patients
Hydroxychloroquine with prior RA N = 956,374	AmbEMR	57,662	Hydroxychloroquine + Azithromycin with prior RA N = 323,122	AmbEMR	13,197
	CCAE	66,756		CCAE	32,693
	Clinformatics	51,352		Clinformatics	23,645
	CPRD	9,129		CPRD	115
	DAGermany	3,887		DAGermany	89
	IMRD	8,855		IMRD	112
	IPCI	1,479		JMDC	22
	JMDC	241		IPCI	121
	MDCD	8,095		MDCD	3,811
	MDCR	15,853		MDCR	8,156
	OpenClaims	621,124		OpenClaims	216,195
	OptumEHR	78,647		OptumEHR	18,592
	SIDIAP	1,247		SIDIAP	130
	VA	32,047		VA	6,244
Sulfasalazine with prior RA N = 310,350	AmbEMR	15,358	Hydroxychloroquine + Amoxicillin with prior RA N = 351,956	AmbEMR	12,084
	CCAE	22,407		CCAE	32,591
	Clinformatics	17,411		Clinformatics	24,547
	CPRD	11,401		CPRD	5,530
	DAGermany	5,047		DAGermany	202
	IMRD	8,463		IMRD	5,104
	IPCI	528		JDMC	30
	JMDC	2,099		IPCI	483
	MDCD	2,200		MDCD	3,818
	MDCR	5,193		MDCR	9,285
	OpenClaims	183,566		OpenClaims	233,107
	OptumEHR	21,944		OptumEHR	16,480
	SIDIAP	381		SIDIAP	686
	VA	14,352		VA	8,009

*Note that HCQ+AZM vs HCQ+AMX cohorts in JDMC were of insufficient sample to fit PS models so were not included in the study diagnostics or population-level effect estimation analyses.

S6. Covariate balance before and after PS stratification

S6.1. HCQ vs SSZ

S6.1.1. AmbEMR

Characteristic	Before stratification			After stratification		
	HCQ	SSZ		HCQ	SSZ	
	%	%	Std. diff	%	%	Std. diff
Age group						
15-19	0.1	0.1	0.00	0.1	0.1	0.00
20-24	0.7	0.6	0.01	0.7	0.6	0.01
25-29	1.3	1.4	-0.01	1.3	1.4	0.00
30-34	2.4	2.2	0.02	2.3	2.3	0.00
35-39	3.5	3.7	-0.01	3.5	3.4	0.01
40-44	5.4	5.0	0.02	5.3	5.1	0.01
45-49	7.4	7.0	0.02	7.4	7.5	0.00
50-54	10.4	11.0	-0.02	10.5	10.5	0.00
55-59	13.1	13.4	-0.01	13.2	12.9	0.01
60-64	13.8	14.4	-0.02	13.9	14.1	0.00
65-69	13.7	14.2	-0.01	13.8	13.7	0.00
70-74	13.1	13.3	-0.01	13.1	13.9	-0.02
75-79	11.2	9.8	0.05	10.9	11.0	0.00
80-84	3.9	3.9	0.00	3.9	3.7	0.01
Gender: female	79.7	74.1	0.13	78.3	78.4	0.00
Race						
race = Asian	1.4	1.5	-0.02	1.4	1.3	0.00
race = White	70.9	71.7	-0.02	71.1	71.3	0.00
race = African American	8.0	7.8	0.01	8.0	7.7	0.01
Ethnicity						
ethnicity = Hispanic or Latino	1.3	1.8	-0.04	1.4	1.5	-0.01
ethnicity = Not Hispanic or Latino	80.3	81.0	-0.02	80.5	80.3	0.00
Medical history: General						
Acute respiratory disease	13.5	14.2	-0.02	13.7	13.8	0.00
Attention deficit hyperactivity disorder	0.6	0.4	0.02	0.6	0.5	0.01
Chronic liver disease	1.5	1.5	0.00	1.5	1.4	0.01
Chronic obstructive lung disease	7.0	7.2	-0.01	7.1	6.9	0.01
Crohn's disease	0.4	1.1	-0.08	0.5	0.6	-0.02
Dementia	0.6	0.5	0.02	0.6	0.6	0.00
Diabetes mellitus	13.2	13.2	0.00	13.2	13.7	-0.02
Gastroesophageal reflux disease	15.9	14.6	0.04	15.6	15.7	0.00
Gastrointestinal hemorrhage	1.2	1.2	0.00	1.2	1.2	0.00
Human immunodeficiency virus infection	0.1	0.1	0.00	0.1	0.1	0.00
Hyperlipidemia	29.0	28.1	0.02	28.8	29.1	-0.01
Hypertensive disorder	38.0	37.1	0.02	37.8	38.2	-0.01
Lesion of liver	0.9	0.8	0.01	0.9	0.8	0.01
Obesity	8.9	8.9	0.00	8.9	9.0	0.00
Osteoarthritis	25.5	27.1	-0.04	25.8	25.2	0.01
Pneumonia	3.1	3.4	-0.01	3.2	3.1	0.01
Psoriasis	1.5	3.2	-0.11	1.7	2.0	-0.02
Renal impairment	5.3	4.8	0.02	5.2	5.3	-0.01
Rheumatoid arthritis	84.0	82.2	0.05	83.6	84.4	-0.02
Ulcerative colitis	0.3	1.2	-0.10	0.4	0.6	-0.03
Urinary tract infectious disease	6.0	5.2	0.04	5.8	5.5	0.01
Viral hepatitis C	1.0	1.0	0.00	1.0	0.9	0.01
Visual system disorder	8.6	8.8	0.00	8.6	8.5	0.00
Medical history: Cardiovascular disease						
Atrial fibrillation	3.3	3.1	0.01	3.2	3.2	0.00
Cerebrovascular disease	3.1	2.8	0.02	3.1	3.1	0.00
Coronary arteriosclerosis	5.7	5.6	0.00	5.7	5.6	0.00
Heart disease	15.9	14.6	0.04	15.6	15.6	0.00
Heart failure	2.6	2.3	0.02	2.6	2.4	0.01
Ischemic heart disease	1.7	1.6	0.01	1.7	1.5	0.02

Characteristic	Before stratification			After stratification		
	HCQ	SSZ		HCQ	SSZ	
	%	%	Std. diff	%	%	Std. diff
Pulmonary embolism	0.8	0.7	0.01	0.8	0.7	0.01
Venous thrombosis	1.3	1.2	0.01	1.3	1.4	-0.01
Medical history: Neoplasms						
Hematologic neoplasm	0.4	0.3	0.02	0.4	0.4	0.01
Malignant lymphoma	0.4	0.5	-0.01	0.4	0.6	-0.03
Malignant neoplasm of anorectum	0.1	0.1	-0.01	0.1	0.1	0.00
Malignant neoplastic disease	6.9	6.6	0.01	6.8	7.0	-0.01
Malignant tumor of breast	1.5	1.4	0.01	1.4	1.6	-0.01
Malignant tumor of colon	0.3	0.3	0.00	0.3	0.3	-0.01
Malignant tumor of lung	0.3	0.4	-0.02	0.3	0.3	-0.01
Malignant tumor of urinary bladder	0.2	0.3	-0.01	0.2	0.2	0.00
Primary malignant neoplasm of prostate	0.4	0.3	0.00	0.4	0.3	0.01
Medication use						
Agents acting on the renin-angiotensin system	33.2	33.6	-0.01	33.3	33.6	-0.01
Antibacterials for systemic use	24.7	25.3	-0.01	24.9	24.5	0.01
Antidepressants	36.1	34.9	0.02	35.7	35.7	0.00
Antiepileptics	24.0	24.1	0.00	24.0	24.0	0.00
Antiinflammatory and antirheumatic products	42.8	44.4	-0.03	43.1	43.4	0.00
Antineoplastic agents	35.9	41.4	-0.11	37.3	37.8	-0.01
Antipsoriatics	0.6	0.7	0.00	0.6	0.7	0.00
Antithrombotic agents	25.2	23.5	0.04	24.9	25.2	-0.01
Beta blocking agents	25.2	25.1	0.00	25.2	25.8	-0.02
Calcium channel blockers	17.5	17.7	0.00	17.5	17.7	0.00
Diuretics	30.6	30.0	0.01	30.5	31.2	-0.01
Drugs for obstructive airway diseases	30.9	30.0	0.02	30.8	31.0	0.00
Drugs used in diabetes	14.2	14.3	0.00	14.3	14.8	-0.01
Immunosuppressants	49.4	61.2	-0.24	52.2	52.1	0.00
Lipid modifying agents	33.2	33.7	-0.01	33.3	33.5	0.00
Opioids	28.4	30.7	-0.05	28.9	28.8	0.00
Psycholeptics	26.3	25.6	0.02	26.1	26.1	0.00
Psychostimulants, agents used for adhd and nootropics	4.0	3.8	0.01	4.0	4.0	0.00

S6.1.2. CCAE

Characteristic	Before stratification			After stratification		
	HCQ	SSZ		HCQ	SSZ	
	%	%	Std. diff	%	%	Std. diff
Age group						
20-24	1.9	1.9	0.00	1.8	2.0	-0.01
25-29	2.6	2.6	0.00	2.5	2.7	-0.01
30-34	4.5	4.6	0.00	4.5	4.4	0.00
40-44	9.8	9.5	0.01	9.7	9.5	0.01
45-49	13.7	12.9	0.02	13.6	13.4	0.00
50-54	18.2	18.1	0.00	18.2	18.0	0.01
55-59	20.6	21.0	-0.01	20.8	20.8	0.00
60-64	19.0	19.8	-0.02	19.4	19.8	-0.01
65-69	1.8	1.7	0.00	1.8	1.6	0.01
Gender: female	82.0	74.3	0.19	80.1	79.7	0.01
Medical history: General						
Acute respiratory disease	35.5	34.4	0.02	35.1	34.8	0.01
Chronic liver disease	3.2	3.2	0.00	3.2	3.3	0.00
Chronic obstructive lung disease	4.2	4.5	-0.01	4.3	4.5	-0.01
Crohn's disease	0.6	1.8	-0.12	0.7	1.1	-0.04
Dementia	0.2	0.2	0.01	0.2	0.2	0.00
Depressive disorder	13.4	13.5	0.00	13.3	13.5	0.00
Diabetes mellitus	13.5	13.4	0.00	13.6	13.8	-0.01
Gastroesophageal reflux disease	13.7	13.5	0.01	13.6	13.5	0.00
Gastrointestinal hemorrhage	2.9	3.4	-0.03	3.0	3.2	-0.01
Human immunodeficiency virus infection	0.1	0.2	-0.01	0.1	0.1	0.00
Hyperlipidemia	31.5	30.5	0.02	31.2	31.4	0.00
Hypertensive disorder	34.7	34.9	0.00	34.8	35.0	0.00
Lesion of liver	0.9	0.8	0.01	0.9	0.8	0.00
Obesity	9.3	9.1	0.00	9.2	9.5	-0.01
Osteoarthritis	43.4	44.4	-0.02	43.5	44.2	-0.01
Pneumonia	4.0	3.9	0.00	4.0	4.0	0.00
Psoriasis	3.0	8.9	-0.25	3.8	5.2	-0.07
Renal impairment	3.1	2.8	0.02	3.0	2.8	0.01
Rheumatoid arthritis	84.2	85.7	-0.04	84.9	85.3	-0.01
Schizophrenia	0.1	0.1	-0.01	0.1	0.1	-0.01
Ulcerative colitis	0.6	1.9	-0.12	0.7	1.0	-0.04
Urinary tract infectious disease	11.9	10.8	0.03	11.6	11.5	0.00
Viral hepatitis C	1.1	1.0	0.01	1.1	1.0	0.01
Visual system disorder	25.7	26.2	-0.01	25.7	25.9	0.00
Medical history: Cardiovascular disease						
Atrial fibrillation	1.4	1.3	0.02	1.4	1.3	0.01
Cerebrovascular disease	2.8	2.6	0.01	2.8	2.9	-0.01
Heart disease	15.7	15.0	0.02	15.5	15.4	0.00
Heart failure	1.9	2.0	0.00	1.9	2.0	0.00
Ischemic heart disease	2.9	3.1	-0.01	3.0	3.1	-0.01
Peripheral vascular disease	1.6	1.5	0.01	1.5	1.6	-0.01
Pulmonary embolism	0.8	0.5	0.03	0.8	0.6	0.02
Medical history: Neoplasms						
Hematologic neoplasm	1.0	1.0	0.00	1.0	1.0	0.00
Malignant lymphoma	0.5	0.5	0.00	0.5	0.4	0.02
Malignant neoplasm of anorectum	0.1	0.1	0.00	0.1	0.1	0.00
Malignant tumor of breast	1.8	1.6	0.02	1.7	1.7	0.00
Malignant tumor of colon	0.2	0.2	0.00	0.2	0.2	0.00
Malignant tumor of lung	0.1	0.2	-0.01	0.2	0.1	0.00
Primary malignant neoplasm of prostate	0.4	0.4	-0.01	0.4	0.4	0.00
Medication use						
Agents acting on the renin-angiotensin system	24.3	24.9	-0.01	24.5	24.6	0.00
Antibacterials for systemic use	43.8	44.3	-0.01	43.8	44.0	0.00
Antidepressants	36.4	36.5	0.00	36.3	36.5	0.00
Antiepileptics	20.3	21.0	-0.02	20.4	20.2	0.00

Characteristic	Before stratification			After stratification		
	HCQ	SSZ		HCQ	SSZ	
	%	%	Std. diff	%	%	Std. diff
Antiinflammatory and antirheumatic products	55.3	57.4	-0.04	55.8	56.7	-0.02
Antineoplastic agents	30.7	37.8	-0.15	33.0	33.1	0.00
Antipsoriatics	0.7	1.2	-0.06	0.7	1.0	-0.03
Antithrombotic agents	7.4	7.3	0.01	7.4	7.3	0.00
Beta blocking agents	15.8	16.2	-0.01	15.9	16.2	-0.01
Calcium channel blockers	11.7	11.5	0.01	11.7	11.8	0.00
Drugs for acid related disorders	32.3	33.6	-0.03	32.6	32.6	0.00
Drugs for obstructive airway diseases	29.7	29.3	0.01	29.5	29.5	0.00
Drugs used in diabetes	10.4	10.5	0.00	10.5	10.8	-0.01
Immunosuppressants	39.6	53.0	-0.27	43.4	43.6	0.00
Lipid modifying agents	22.6	23.5	-0.02	22.8	23.2	-0.01
Opioids	38.5	40.7	-0.05	39.0	39.3	-0.01
Psycholeptics	33.6	33.7	0.00	33.4	33.3	0.00
Psychostimulants, agents used for adhd and nootropics	5.9	5.6	0.01	5.8	5.7	0.01

S6.1.3. CPRD

Characteristic	Before stratification			After stratification		
	HCQ	SSZ		HCQ	SSZ	
	%	%	Std. diff	%	%	Std. diff
Age group						
15-19	0.3	0.2	0.01	0.3	0.2	0.00
20-24	0.8	1.0	-0.02	0.9	0.9	-0.01
25-29	1.2	1.7	-0.04	1.4	1.6	-0.02
30-34	2.7	3.1	-0.02	3.0	3.0	0.00
35-39	4.6	4.6	0.00	4.7	4.6	0.01
40-44	5.6	6.6	-0.04	6.2	6.4	0.00
45-49	8.9	8.1	0.03	8.6	8.5	0.00
50-54	11.4	10.8	0.02	11.2	10.9	0.01
55-59	13.2	12.6	0.02	13.4	12.8	0.02
60-64	13.1	13.2	0.00	12.8	13.0	-0.01
65-69	12.6	12.7	0.00	12.6	12.7	0.00
70-74	10.8	11.6	-0.02	11.2	11.2	0.00
80-84	4.5	4.3	0.01	4.2	4.4	-0.01
85-89	1.6	1.3	0.03	1.3	1.4	-0.01
90-94	0.3	0.2	0.03	0.3	0.2	0.03
Medical history: General						
Acute respiratory disease	9.2	9.1	0.00	9.4	9.3	0.01
Chronic obstructive lung disease	2.3	2.0	0.02	2.2	2.0	0.01
Crohn's disease	<0.1	0.1	-0.03	0.1	0.1	-0.01
Depressive disorder	2.7	3.2	-0.03	3.0	3.1	0.00
Diabetes mellitus	2.2	2.1	0.01	2.1	2.2	-0.01
Gastroesophageal reflux disease	0.5	0.4	0.02	0.4	0.4	0.00
Gastrointestinal hemorrhage	1.0	1.4	-0.04	1.2	1.4	-0.02
Hyperlipidemia	1.2	1.0	0.02	1.2	1.0	0.01
Hypertensive disorder	3.0	3.9	-0.05	3.2	3.6	-0.02
Lesion of liver	0.1	0.1	0.01	0.1	0.1	0.01
Obesity	0.4	0.5	-0.01	0.4	0.5	-0.01
Osteoarthritis	7.6	10.4	-0.10	8.6	8.9	-0.01
Pneumonia	0.8	0.6	0.03	0.9	0.6	0.03
Psoriasis	0.7	1.9	-0.10	1.2	1.5	-0.03
Renal impairment	2.3	1.8	0.03	1.9	2.2	-0.02
Rheumatoid arthritis	61.3	72.3	-0.24	67.4	66.3	0.02
Visual system disorder	7.0	6.9	0.00	6.7	6.9	-0.01
Medical history: Cardiovascular disease						
Atrial fibrillation	0.7	0.7	0.00	0.8	0.7	0.02
Cerebrovascular disease	0.4	0.8	-0.05	0.5	0.7	-0.03
Coronary arteriosclerosis	0.1	0.1	0.00	0.1	0.1	0.00
Heart disease	3.0	3.4	-0.02	3.4	3.1	0.01
Heart failure	0.5	0.7	-0.02	0.7	0.6	0.01
Ischemic heart disease	1.0	1.5	-0.04	1.2	1.3	-0.01
Peripheral vascular disease	0.2	0.2	0.01	0.3	0.2	0.02
Pulmonary embolism	0.2	0.2	-0.01	0.2	0.2	-0.02
Venous thrombosis	0.8	1.0	-0.01	0.9	1.0	0.00
Medical history: Neoplasms						
Malignant neoplastic disease	1.3	1.0	0.02	1.1	1.0	0.01
Malignant tumor of breast	0.1	0.1	0.00	0.1	0.1	-0.01
Medication use						
Agents acting on the renin-angiotensin system	22.0	16.8	0.13	19.4	19.4	0.00
Antibacterials for systemic use	37.3	35.0	0.05	35.7	36.2	-0.01
Antidepressants	26.6	22.6	0.09	25.0	24.9	0.00
Antiepileptics	6.5	4.3	0.10	5.4	5.2	0.01
Antiinflammatory and antirheumatic products	60.5	67.7	-0.15	64.3	63.8	0.01
Antineoplastic agents	51.4	28.6	0.48	39.3	40.8	-0.03
Antipsoriatics	0.7	1.3	-0.06	0.9	1.2	-0.02
Antithrombotic agents	17.9	16.7	0.03	17.3	17.2	0.00
Beta blocking agents	14.4	13.5	0.03	14.4	13.9	0.02

Characteristic	Before stratification			After stratification		
	HCQ	SSZ		HCQ	SSZ	
	%	%	Std. diff	%	%	Std. diff
Calcium channel blockers	15.5	12.1	0.10	13.4	13.6	0.00
Diuretics	19.0	20.9	-0.05	20.0	20.1	0.00
Drugs for acid related disorders	58.3	51.0	0.15	54.1	54.6	-0.01
Drugs used in diabetes	7.1	5.8	0.05	6.2	6.6	-0.02
Immunosuppressants	55.2	30.0	0.53	42.2	43.0	-0.02
Lipid modifying agents	23.0	17.1	0.15	19.5	19.8	-0.01
Opioids	36.3	41.1	-0.10	39.0	38.9	0.00
Psycholeptics	16.5	16.4	0.00	16.7	16.5	0.00
Psychostimulants, agents used for adhd and nootropics	0.1	0.1	0.01	0.1	0.1	0.01

S6.1.4. Clinformatics

Characteristic	Before stratification			After stratification		
	HCQ	SSZ		HCQ	SSZ	
	%	%	Std. diff	%	%	Std. diff
Age group						
15-19	0.3	0.4	-0.01	0.3	0.4	-0.02
20-24	1.1	1.1	-0.01	1.1	1.1	-0.01
25-29	1.8	2.0	-0.01	1.8	1.8	0.00
30-34	3.4	3.2	0.01	3.3	3.3	0.00
35-39	4.9	5.2	-0.02	4.9	4.8	0.00
40-44	6.8	6.7	0.01	6.8	6.5	0.01
45-49	8.9	9.1	0.00	8.9	8.9	0.00
50-54	11.6	11.6	0.00	11.6	11.7	0.00
55-59	12.5	13.3	-0.02	12.7	12.8	0.00
60-64	11.6	12.6	-0.03	11.9	11.9	0.00
65-69	11.3	12.2	-0.03	11.6	11.7	0.00
70-74	10.8	10.6	0.01	10.9	10.6	0.01
75-79	7.9	6.9	0.04	7.7	7.7	0.00
80-84	5.0	3.8	0.06	4.7	4.9	-0.01
85-89	1.9	1.3	0.05	1.8	1.7	0.01
Gender: female	79.6	71.9	0.18	77.6	77.6	0.00
Medical history: General						
Acute respiratory disease	34.7	33.2	0.03	34.2	34.2	0.00
Attention deficit hyperactivity disorder	1.2	1.1	0.00	1.2	1.1	0.00
Chronic liver disease	3.7	3.8	-0.01	3.7	3.9	-0.01
Chronic obstructive lung disease	11.2	10.9	0.01	11.1	11.2	0.00
Crohn's disease	0.7	2.4	-0.14	0.9	1.4	-0.05
Dementia	1.3	1.2	0.01	1.2	1.5	-0.02
Depressive disorder	18.5	18.1	0.01	18.4	18.2	0.00
Diabetes mellitus	20.9	20.2	0.02	20.7	21.1	-0.01
Gastroesophageal reflux disease	21.6	21.1	0.01	21.5	21.8	-0.01
Gastrointestinal hemorrhage	4.0	4.5	-0.02	4.0	4.1	0.00
Human immunodeficiency virus infection	0.2	0.2	-0.01	0.2	0.2	-0.01
Hyperlipidemia	46.8	45.3	0.03	46.4	47.1	-0.01
Hypertensive disorder	51.4	51.0	0.01	51.2	51.9	-0.01
Lesion of liver	1.3	1.2	0.00	1.3	1.3	0.00
Obesity	12.2	12.5	-0.01	12.2	12.2	0.00
Osteoarthritis	56.4	55.4	0.02	56.1	56.7	-0.01
Pneumonia	6.5	5.8	0.03	6.3	6.4	0.00
Psoriasis	3.2	8.0	-0.21	3.9	4.9	-0.05
Renal impairment	9.9	9.2	0.03	9.7	9.9	0.00
Rheumatoid arthritis	84.3	85.5	-0.04	84.8	85.2	-0.01
Schizophrenia	0.2	0.1	0.01	0.2	0.1	0.01
Ulcerative colitis	0.7	2.2	-0.13	0.9	1.3	-0.04
Viral hepatitis C	1.5	1.7	-0.02	1.5	1.7	-0.02
Visual system disorder	40.3	39.7	0.01	40.0	39.8	0.00
Medical history: Cardiovascular disease						
Atrial fibrillation	5.1	4.1	0.04	4.8	4.8	0.00
Cerebrovascular disease	6.3	5.5	0.03	6.1	6.0	0.00
Coronary arteriosclerosis	10.8	10.5	0.01	10.7	11.0	-0.01
Heart disease	27.8	26.1	0.04	27.3	27.9	-0.01
Heart failure	6.3	5.8	0.02	6.1	6.4	-0.01
Ischemic heart disease	6.5	6.4	0.01	6.5	6.6	-0.01
Peripheral vascular disease	4.7	4.3	0.02	4.7	4.6	0.00
Pulmonary embolism	1.2	0.9	0.03	1.2	1.1	0.01
Venous thrombosis	2.3	2.1	0.02	2.2	2.4	-0.01
Medical history: Neoplasms						
Hematologic neoplasm	1.4	1.2	0.02	1.4	1.3	0.01
Malignant lymphoma	0.7	0.8	-0.01	0.7	0.8	-0.01
Malignant neoplasm of anorectum	0.2	0.3	-0.02	0.2	0.2	-0.02
Malignant neoplastic disease	10.4	10.4	0.00	10.4	10.6	-0.01

Characteristic	Before stratification			After stratification		
	HCQ	SSZ		HCQ	SSZ	
	%	%	Std. diff	%	%	Std. diff
Malignant tumor of breast	2.5	2.0	0.04	2.4	2.4	0.00
Malignant tumor of colon	0.4	0.5	-0.01	0.4	0.5	-0.01
Malignant tumor of lung	0.4	0.4	0.01	0.4	0.4	0.01
Malignant tumor of urinary bladder	0.3	0.4	-0.01	0.3	0.3	0.00
Primary malignant neoplasm of prostate	1.0	1.2	-0.02	1.0	1.0	0.00
Medication use						
Agents acting on the renin-angiotensin system	32.3	32.6	-0.01	32.4	32.7	-0.01
Antibacterials for systemic use	41.2	41.9	-0.01	41.3	41.1	0.00
Antidepressants	35.1	35.2	0.00	35.0	34.9	0.00
Antiinflammatory and antirheumatic products	47.9	50.1	-0.04	48.5	49.0	-0.01
Antineoplastic agents	29.8	38.5	-0.18	32.6	32.2	0.01
Antipsoriatics	0.9	1.1	-0.03	0.9	0.9	0.00
Antithrombotic agents	11.8	10.6	0.04	11.5	11.4	0.00
Beta blocking agents	23.2	22.4	0.02	23.1	23.2	0.00
Calcium channel blockers	17.8	16.6	0.03	17.6	17.8	0.00
Diuretics	30.3	29.0	0.03	30.0	30.7	-0.02
Drugs for acid related disorders	33.7	34.4	-0.02	33.8	34.4	-0.01
Drugs for obstructive airway diseases	29.4	28.9	0.01	29.2	29.5	0.00
Drugs used in diabetes	14.1	14.1	0.00	14.1	14.5	-0.01
Immunosuppressants	38.7	53.1	-0.29	43.0	42.2	0.02
Lipid modifying agents	31.9	32.1	0.00	32.0	32.6	-0.01
Opioids	38.4	40.2	-0.04	38.8	38.9	0.00
Psycholeptics	30.4	29.3	0.02	30.1	29.9	0.00
Psychostimulants, agents used for adhd and nootropics	4.0	3.7	0.02	4.0	3.7	0.01

S6.1.5. DAGermany

Characteristic	Before stratification			After stratification		
	HCQ	SSZ		HCQ	SSZ	
	%	%	Std. diff	%	%	Std. diff
Age group						
15-19	0.3	0.5	-0.03	0.3	0.5	-0.03
20-24	1.1	1.3	-0.02	1.1	1.2	-0.01
25-29	1.9	2.4	-0.04	2.0	2.4	-0.03
30-34	2.4	3.5	-0.07	2.9	3.0	-0.01
35-39	4.0	4.1	-0.01	3.9	4.1	-0.01
40-44	5.8	6.6	-0.03	6.1	6.3	-0.01
50-54	12.0	12.7	-0.02	12.7	12.5	0.01
55-59	14.2	14.8	-0.02	14.8	14.9	0.00
60-64	13.2	12.1	0.03	12.3	12.4	0.00
65-69	11.4	10.6	0.03	11.2	10.6	0.02
70-74	10.2	9.5	0.02	9.8	9.7	0.00
75-79	9.1	7.5	0.06	8.2	8.1	0.01
80-84	4.4	3.9	0.02	4.2	4.0	0.01
85-89	1.4	1.0	0.04	1.2	1.0	0.02
90-94	0.2	0.2	0.00	0.2	0.2	0.00
Gender: female	81.4	72.5	0.21	76.0	77.2	-0.03
Medical history: General						
Acute respiratory disease	11.9	12.6	-0.02	12.2	12.0	0.01
Attention deficit hyperactivity disorder	<0.1	0.1	-0.01	<0.1	<0.1	0.02
Chronic liver disease	0.6	0.3	0.05	0.6	0.3	0.05
Chronic obstructive lung disease	3.4	4.0	-0.03	3.4	3.7	-0.02
Crohn's disease	0.2	1.5	-0.14	0.5	1.0	-0.06
Dementia	0.6	0.5	0.01	0.6	0.5	0.01
Depressive disorder	8.3	8.3	0.00	8.2	8.0	0.01
Diabetes mellitus	6.9	7.6	-0.03	7.3	7.1	0.01
Gastroesophageal reflux disease	1.9	2.0	-0.01	1.9	2.0	0.00
Gastrointestinal hemorrhage	0.4	0.6	-0.03	0.5	0.6	-0.02
Hyperlipidemia	7.1	8.4	-0.05	8.0	7.7	0.01
Hypertensive disorder	18.5	19.2	-0.02	19.0	18.3	0.02
Lesion of liver	0.5	0.3	0.03	0.4	0.3	0.02
Obesity	2.2	2.4	-0.01	2.2	2.2	0.00
Osteoarthritis	11.4	13.2	-0.06	12.4	12.1	0.01
Pneumonia	1.9	1.5	0.03	2.0	1.6	0.04
Psoriasis	2.3	4.4	-0.12	3.7	3.5	0.01
Renal impairment	2.6	2.3	0.02	2.4	2.4	0.00
Rheumatoid arthritis	46.4	54.7	-0.17	52.4	50.1	0.04
Schizophrenia	<0.1	0.1	-0.03	<0.1	0.1	-0.01
Ulcerative colitis	0.2	1.6	-0.16	0.4	1.1	-0.08
Urinary tract infectious disease	4.0	4.1	-0.01	4.0	3.9	0.00
Viral hepatitis C	0.3	0.1	0.04	0.3	0.1	0.04
Visual system disorder	5.8	5.6	0.01	5.6	5.5	0.01
Medical history: Cardiovascular disease						
Atrial fibrillation	0.8	0.8	0.00	0.8	0.7	0.00
Cerebrovascular disease	1.4	1.8	-0.03	1.4	1.6	-0.02
Coronary arteriosclerosis	1.0	1.1	-0.01	1.1	1.1	0.00
Heart disease	10.5	11.4	-0.03	10.6	10.6	0.00
Heart failure	2.1	2.6	-0.04	2.0	2.4	-0.03
Ischemic heart disease	4.3	5.2	-0.04	4.7	4.6	0.00
Peripheral vascular disease	1.0	1.0	0.00	0.9	0.9	0.00
Pulmonary embolism	0.6	0.3	0.04	0.6	0.4	0.03
Venous thrombosis	1.3	1.4	-0.02	1.5	1.5	0.00
Medical history: Neoplasms						
Hematologic neoplasm	0.3	0.2	0.03	0.3	0.2	0.02
Malignant lymphoma	0.2	0.2	0.01	0.2	0.1	0.01
Malignant neoplasm of anorectum	0.2	0.2	0.01	0.1	0.1	0.00
Malignant neoplastic disease	3.4	3.2	0.01	3.2	3.2	0.00

Characteristic	Before stratification			After stratification		
	HCQ	SSZ		HCQ	SSZ	
	%	%	Std. diff	%	%	Std. diff
Malignant tumor of breast	0.7	0.6	0.01	0.6	0.6	0.00
Malignant tumor of colon	0.3	0.1	0.03	0.3	0.1	0.04
Malignant tumor of lung	<0.1	<0.1	0.02	<0.1	<0.1	0.00
Malignant tumor of urinary bladder	<0.1	0.2	-0.05	<0.1	0.2	-0.05
Primary malignant neoplasm of prostate	0.2	0.3	-0.03	0.2	0.2	-0.02
Medication use						
Agents acting on the renin-angiotensin system	16.7	17.1	-0.01	16.8	16.5	0.01
Antibacterials for systemic use	12.7	14.7	-0.06	13.5	13.7	0.00
Antiepileptics	3.0	2.3	0.04	2.6	2.2	0.02
Antiinflammatory and antirheumatic products	33.3	36.1	-0.06	35.4	34.7	0.01
Antineoplastic agents	31.8	26.8	0.11	29.4	30.4	-0.02
Antipsoriatics	0.2	0.3	-0.01	0.2	0.2	0.00
Antithrombotic agents	10.1	9.9	0.01	9.6	9.4	0.01
Beta blocking agents	13.7	13.3	0.01	13.0	13.1	0.00
Calcium channel blockers	7.6	7.7	0.00	7.6	7.3	0.01
Diuretics	13.9	14.6	-0.02	14.5	13.7	0.02
Drugs for acid related disorders	32.1	32.7	-0.01	32.3	32.5	0.00
Drugs for obstructive airway diseases	7.0	7.7	-0.03	7.4	7.2	0.01
Drugs used in diabetes	4.4	4.8	-0.02	4.7	4.5	0.01
Immunosuppressants	40.7	35.0	0.12	38.9	38.5	0.01
Lipid modifying agents	7.3	9.2	-0.07	8.2	8.2	0.00
Opioids	12.5	12.9	-0.01	12.2	12.3	0.00
Psycholeptics	4.8	6.0	-0.05	4.8	5.3	-0.02
Psychostimulants, agents used for adhd and nootropics	0.2	0.4	-0.03	0.2	0.3	-0.03

S6.1.6. IMRD

Characteristic	Before stratification			After stratification		
	HCQ	SSZ		HCQ	SSZ	
	%	%	Std. diff	%	%	Std. diff
Age group						
15-19	0.2	0.2	0.02	0.2	0.2	0.01
20-24	0.8	1.0	-0.03	0.8	1.0	-0.02
25-29	1.2	1.6	-0.03	1.4	1.6	-0.01
30-34	2.8	3.2	-0.02	2.9	3.1	-0.01
35-39	4.8	5.0	-0.01	5.0	4.8	0.01
40-44	6.0	6.1	0.00	6.3	6.0	0.01
45-49	8.7	7.8	0.03	8.4	8.5	0.00
50-54	11.3	10.8	0.02	11.2	11.0	0.01
55-59	13.0	12.4	0.02	12.7	12.4	0.01
60-64	13.2	13.8	-0.02	13.5	13.5	0.00
65-69	13.0	12.8	0.01	12.9	13.0	0.00
70-74	11.0	11.4	-0.01	11.2	11.2	0.00
75-79	7.7	8.3	-0.02	7.8	8.0	0.00
80-84	4.4	4.1	0.02	4.1	4.2	0.00
85-89	1.5	1.3	0.02	1.3	1.4	-0.01
90-94	0.3	0.2	0.02	0.2	0.2	0.00
Gender: female	73.2	68.0	0.11	70.7	71.0	0.00
Race						
race = Asian	0.1	0.1	0.02	0.1	0.1	0.00
race = White	29.8	25.8	0.09	27.4	27.6	0.00
race = Asian Indian	0.7	0.4	0.04	0.6	0.5	0.02
race = Bangladeshi	0.1	<0.1	0.02	<0.1	<0.1	0.02
race = Chinese	0.1	<0.1	0.03	0.1	<0.1	0.03
race = Pakistani	0.3	0.2	0.03	0.3	0.2	0.03
race = Black	0.4	0.2	0.03	0.3	0.2	0.02
race = African	0.2	<0.1	0.05	0.2	<0.1	0.04
race = European	0.1	<0.1	0.02	0.1	<0.1	0.02
Medical history: General						
Acute respiratory disease	9.2	9.3	0.00	9.2	9.1	0.00
Chronic liver disease	0.1	0.1	0.00	<0.1	<0.1	0.00
Chronic obstructive lung disease	2.1	2.5	-0.02	2.2	2.3	-0.01
Crohn's disease	<0.1	0.2	-0.05	<0.1	0.1	-0.04
Dementia	0.2	0.1	0.03	0.1	0.1	0.02
Depressive disorder	2.6	2.9	-0.02	2.9	2.7	0.01
Diabetes mellitus	2.3	2.2	0.01	2.2	2.1	0.01
Gastroesophageal reflux disease	0.3	0.6	-0.03	0.4	0.6	-0.03
Gastrointestinal hemorrhage	0.9	1.3	-0.04	1.0	1.1	-0.01
Hyperlipidemia	1.1	1.3	-0.02	1.1	1.2	0.00
Hypertensive disorder	3.1	3.7	-0.03	3.2	3.3	0.00
Lesion of liver	0.1	0.1	0.01	0.1	<0.1	0.01
Obesity	0.3	0.5	-0.03	0.4	0.6	-0.03
Osteoarthritis	7.5	9.4	-0.07	8.1	8.2	0.00
Pneumonia	0.7	0.6	0.00	0.7	0.7	0.00
Psoriasis	0.8	2.2	-0.12	1.3	1.6	-0.02
Rheumatoid arthritis	61.4	69.3	-0.17	65.6	63.7	0.04
Schizophrenia	<0.1	<0.1	0.01	<0.1	<0.1	0.02
Ulcerative colitis	<0.1	<0.1	-0.01	<0.1	<0.1	-0.01
Urinary tract infectious disease	3.1	2.9	0.01	3.1	3.0	0.01
Visual system disorder	6.6	7.0	-0.02	6.6	6.8	0.00
Medical history: Cardiovascular disease						
Atrial fibrillation	0.7	0.7	0.00	0.7	0.6	0.01
Cerebrovascular disease	0.5	0.7	-0.03	0.5	0.7	-0.02
Coronary arteriosclerosis	0.1	0.1	0.01	0.1	0.1	0.02
Heart disease	2.7	2.9	-0.01	2.8	2.6	0.01
Heart failure	0.4	0.4	-0.01	0.4	0.4	0.01
Ischemic heart disease	0.8	1.2	-0.04	1.0	1.0	0.00

Characteristic	Before stratification			After stratification		
	HCQ	SSZ		HCQ	SSZ	
	%	%	Std. diff	%	%	Std. diff
Peripheral vascular disease	0.2	0.2	0.01	0.3	0.2	0.02
Pulmonary embolism	0.2	0.2	-0.01	0.2	0.3	-0.02
Venous thrombosis	0.8	1.1	-0.03	0.8	1.1	-0.02
Medical history: Neoplasms						
Hematologic neoplasm	0.1	0.1	0.01	0.1	<0.1	0.02
Malignant neoplasm of anorectum	<0.1	<0.1	0.02	<0.1	<0.1	0.02
Malignant neoplastic disease	1.3	1.1	0.02	1.2	1.0	0.02
Malignant tumor of breast	0.2	0.2	0.02	0.2	0.2	0.01
Malignant tumor of colon	<0.1	<0.1	-0.01	<0.1	<0.1	0.00
Malignant tumor of lung	0.1	0.1	0.01	0.1	0.1	0.02
Medication use						
Agents acting on the renin-angiotensin system	0.6	0.6	0.00	0.6	0.8	-0.02
Antibacterials for systemic use	3.8	3.4	0.02	3.6	3.5	0.01
Antidepressants	1.1	1.1	-0.01	1.0	1.2	-0.02
Antiepileptics	0.1	0.2	-0.03	0.1	0.2	-0.03
Antiinflammatory and antirheumatic products	1.6	1.9	-0.03	1.6	1.8	-0.01
Antineoplastic agents	1.5	0.6	0.08	1.2	0.9	0.02
Antithrombotic agents	1.1	1.1	0.00	1.2	1.2	0.00
Beta blocking agents	0.4	0.6	-0.02	0.5	0.6	-0.01
Calcium channel blockers	0.6	0.5	0.00	0.6	0.5	0.02
Diuretics	0.8	1.0	-0.02	1.0	0.9	0.01
Drugs for acid related disorders	1.6	1.7	0.00	1.7	1.7	0.01
Drugs for obstructive airway diseases	0.4	0.4	0.00	0.5	0.4	0.00
Drugs used in diabetes	0.2	0.2	0.01	0.2	0.2	0.00
Immunosuppressants	1.6	0.7	0.08	1.3	1.0	0.02
Lipid modifying agents	0.9	0.9	0.00	0.9	1.0	-0.01
Opioids	0.6	0.6	-0.01	0.6	0.6	-0.01
Psycholeptics	0.4	0.5	-0.01	0.4	0.4	-0.01

S6.1.7. IPCI

Characteristic	Before stratification			After stratification		
	HCQ	SSZ		HCQ	SSZ	
	%	%	Std. diff	%	%	Std. diff
Age group						
15-19	0.4	<0.9	0.00	0.4	<0.9	0.02
20-24	0.6	1.3	-0.07	0.6	1.0	-0.04
25-29	1.4	3.0	-0.11	1.5	2.7	-0.08
30-34	1.6	4.4	-0.16	1.8	3.7	-0.12
35-39	3.2	4.0	-0.04	3.4	4.0	-0.03
40-44	6.0	4.4	0.08	6.4	3.4	0.14
45-49	8.5	10.4	-0.06	9.0	10.1	-0.04
50-54	10.5	9.7	0.03	11.0	9.1	0.06
55-59	11.2	14.6	-0.10	11.5	14.4	-0.09
60-64	12.2	12.3	0.00	11.7	12.7	-0.03
65-69	11.8	12.9	-0.03	11.9	12.4	-0.02
70-74	12.3	9.8	0.08	12.0	10.6	0.04
75-79	9.0	8.5	0.02	8.8	9.8	-0.04
80-84	7.4	3.2	0.19	6.7	4.3	0.11
85-89	2.8	0.9	0.14	2.7	1.4	0.09
90-94	0.8	<0.9	0.09	0.8	<0.9	0.08
Gender: female	68.4	68.0	0.01	68.6	67.2	0.03
Medical history: General						
Acute respiratory disease	14.1	16.1	-0.06	14.3	16.2	-0.05
Attention deficit hyperactivity disorder	<0.3	<0.9	-0.03	<0.3	<0.9	-0.01
Depressive disorder	4.7	4.2	0.02	4.8	4.5	0.02
Diabetes mellitus	8.6	8.9	-0.01	8.2	9.9	-0.06
Gastroesophageal reflux disease	0.7	<0.9	0.02	0.7	<0.9	0.04
Gastrointestinal hemorrhage	1.1	1.9	-0.07	1.0	1.8	-0.07
Hyperlipidemia	3.4	4.0	-0.03	3.4	4.3	-0.05
Hypertensive disorder	16.3	17.6	-0.04	15.6	17.6	-0.06
Obesity	1.1	1.1	0.00	1.2	1.1	0.01
Osteoarthritis	9.7	6.4	0.12	9.4	6.7	0.10
Pneumonia	4.1	4.5	-0.02	3.9	4.4	-0.02
Psoriasis	1.1	2.8	-0.12	1.2	2.5	-0.09
Renal impairment	2.1	1.5	0.04	2.0	1.4	0.05
Ulcerative colitis	<0.3	0.9	-0.10	<0.3	1.1	-0.12
Urinary tract infectious disease	9.1	7.4	0.06	9.0	7.0	0.08
Visual system disorder	13.3	13.4	0.00	13.4	12.1	0.04
Medical history: Cardiovascular disease						
Atrial fibrillation	2.9	2.3	0.04	2.7	2.3	0.02
Cerebrovascular disease	2.6	3.0	-0.03	2.4	3.4	-0.06
Coronary arteriosclerosis	0.5	<0.9	0.05	0.5	<0.9	0.07
Heart disease	10.2	9.1	0.04	9.7	10.0	-0.01
Heart failure	1.6	1.1	0.04	1.5	1.4	0.01
Ischemic heart disease	4.5	4.4	0.00	4.3	5.0	-0.03
Peripheral vascular disease	3.6	4.4	-0.04	3.7	4.6	-0.05
Pulmonary embolism	0.4	<0.9	-0.02	0.4	<0.9	-0.01
Venous thrombosis	1.4	0.9	0.04	1.4	<0.9	0.07
Medical history: Neoplasms						
Malignant lymphoma	<0.3	<0.9	-0.02	<0.3	<0.9	0.01
Malignant neoplasm of anorectum	0.5	<0.9	-0.04	0.4	<0.9	-0.04
Malignant neoplastic disease	4.5	4.4	0.01	4.5	4.3	0.01
Malignant tumor of breast	1.1	<0.9	0.06	1.2	<0.9	0.10
Malignant tumor of lung	0.5	0.9	-0.06	0.5	1.3	-0.09
Medication use						
Agents acting on the renin-angiotensin system	29.6	23.5	0.14	28.0	26.6	0.03
Antibacterials for systemic use	30.4	29.5	0.02	30.4	29.1	0.03
Antidepressants	12.8	13.6	-0.02	12.8	13.8	-0.03
Antiepileptics	5.0	4.9	0.00	5.0	5.6	-0.03
Antiinflammatory and antirheumatic products	42.7	45.1	-0.05	42.5	42.9	-0.01

Characteristic	Before stratification			After stratification		
	HCQ	SSZ		HCQ	SSZ	
	%	%	Std. diff	%	%	Std. diff
Antineoplastic agents	42.5	41.1	0.03	41.2	45.5	-0.09
Antipsoriatics	0.7	<0.9	0.00	0.8	0.9	0.00
Antithrombotic agents	22.2	18.4	0.10	20.5	22.6	-0.05
Beta blocking agents	24.0	21.4	0.06	22.7	24.2	-0.04
Calcium channel blockers	16.0	12.5	0.10	15.2	14.5	0.02
Diuretics	25.3	23.1	0.05	24.0	24.5	-0.01
Drugs for acid related disorders	66.9	67.8	-0.02	66.0	67.6	-0.03
Drugs for obstructive airway diseases	25.4	26.7	-0.03	25.6	26.8	-0.03
Drugs used in diabetes	11.5	11.4	0.00	11.0	13.9	-0.09
Immunosuppressants	44.8	46.8	-0.04	44.0	49.1	-0.10
Lipid modifying agents	29.3	25.0	0.10	27.6	29.5	-0.04
Opioids	20.8	21.2	-0.01	20.6	22.7	-0.05
Psycholeptics	19.5	19.3	0.01	19.4	19.9	-0.01
Psychostimulants, agents used for adhd and nootropics	0.9	1.1	-0.03	0.9	1.7	-0.07

S6.1.8. JMDC

Characteristic	Before stratification			After stratification		
	HCQ	SSZ		HCQ	SSZ	
	%	%	Std. diff	%	%	Std. diff
Age group						
15-19	<2.1	<0.2	0.12	<0.8	<0.3	0.04
20-24	4.1	1.1	0.19	1.4	1.2	0.02
25-29	5.4	2.2	0.17	1.8	2.2	-0.03
30-34	9.1	3.8	0.22	3.1	3.8	-0.04
35-39	9.5	7.4	0.08	3.2	7.1	-0.17
40-44	15.4	10.3	0.15	38.4	10.4	0.65
50-54	16.6	16.5	0.00	16.6	16.6	0.00
55-59	13.7	16.0	-0.06	15.6	16.3	-0.02
60-64	4.1	13.4	-0.33	1.4	13.0	-0.46
65-69	3.3	8.4	-0.22	1.1	8.7	-0.35
70-74	<2.1	3.4	-0.22	<0.8	3.6	-0.26
Gender: female	89.6	68.2	0.54	85.5	69.6	0.38
Medical history: General						
Acute respiratory disease	57.3	52.4	0.10	63.6	53.6	0.20
Chronic liver disease	10.8	5.5	0.20	3.7	5.9	-0.10
Chronic obstructive lung disease	<2.1	1.5	0.02	<0.8	1.5	-0.09
Crohn's disease	<2.1	0.5	-0.02	<0.8	0.7	-0.08
Depressive disorder	12.0	8.6	0.11	37.3	9.2	0.66
Diabetes mellitus	39.8	18.5	0.48	13.5	19.8	-0.16
Gastroesophageal reflux disease	<2.1	0.3	0.01	<0.8	0.3	-0.04
Gastrointestinal hemorrhage	5.4	1.3	0.23	12.8	1.4	0.42
Hyperlipidemia	46.5	26.4	0.43	48.9	27.5	0.43
Hypertensive disorder	49.0	23.6	0.55	27.6	24.8	0.06
Lesion of liver	5.8	1.5	0.23	2.0	1.5	0.03
Obesity	<2.1	0.8	-0.05	<0.8	0.9	-0.10
Osteoarthritis	20.3	38.7	-0.41	17.9	38.1	-0.45
Pneumonia	9.5	4.8	0.19	47.4	5.2	1.02
Psoriasis	3.3	5.5	-0.10	1.1	5.6	-0.25
Renal impairment	9.5	1.9	0.34	3.2	2.2	0.06
Rheumatoid arthritis	86.3	99.0	-0.50	95.4	98.9	-0.20
Schizophrenia	3.3	2.3	0.06	1.1	2.5	-0.10
Ulcerative colitis	<2.1	1.5	-0.02	<0.8	1.8	-0.13
Urinary tract infectious disease	4.6	2.5	0.11	34.7	2.8	0.83
Viral hepatitis C	2.5	1.8	0.05	0.8	1.8	-0.08
Visual system disorder	93.4	43.1	1.28	53.6	47.5	0.12
Medical history: Cardiovascular disease						
Cerebrovascular disease	4.1	2.8	0.08	1.4	2.9	-0.10
Coronary arteriosclerosis	<2.1	<0.2	0.05	<0.8	<0.3	-0.02
Heart disease	40.7	12.7	0.67	13.8	13.8	0.00
Heart failure	20.3	5.8	0.44	6.9	6.6	0.01
Ischemic heart disease	22.8	5.1	0.53	7.7	5.7	0.08
Peripheral vascular disease	6.2	2.7	0.17	2.1	2.8	-0.04
Pulmonary embolism	<2.1	0.2	0.12	<0.8	0.4	0.00
Venous thrombosis	9.1	1.6	0.34	3.1	2.0	0.07
Medical history: Neoplasms						
Hematologic neoplasm	<2.1	0.9	0.00	<0.8	0.9	-0.08
Malignant lymphoma	2.5	1.2	0.09	0.8	1.5	-0.06
Malignant neoplastic disease	6.2	6.7	-0.02	2.1	7.1	-0.24
Malignant tumor of breast	<2.1	1.4	-0.06	<0.8	1.5	-0.13
Malignant tumor of colon	<2.1	0.6	-0.03	<0.8	0.6	-0.08
Medication use						
Agents acting on the renin-angiotensin system	26.1	12.7	0.35	8.9	13.5	-0.14
Antibacterials for systemic use	71.4	50.9	0.43	79.3	52.5	0.57
Antidepressants	10.8	7.2	0.13	3.7	7.7	-0.17

Characteristic	Before stratification			After stratification		
	HCQ	SSZ		HCQ	SSZ	
	%	%	Std. diff	%	%	Std. diff
Antiepileptics	11.6	10.9	0.02	3.9	11.6	-0.28
Antiinflammatory and antirheumatic products	39.4	72.9	-0.72	79.5	71.9	0.17
Antineoplastic agents	15.4	28.7	-0.33	16.2	28.5	-0.29
Antipsoriatics	2.1	3.2	-0.07	<0.8	3.4	-0.19
Antithrombotic agents	44.8	12.1	0.78	15.2	13.3	0.05
Beta blocking agents	7.9	3.9	0.17	13.6	4.3	0.31
Calcium channel blockers	19.5	12.4	0.20	17.6	12.8	0.13
Diuretics	9.5	4.0	0.22	3.2	4.3	-0.05
Drugs for acid related disorders	90.0	81.8	0.24	96.6	81.8	0.48
Drugs for obstructive airway diseases	25.7	23.3	0.06	52.9	24.3	0.59
Drugs used in diabetes	8.7	6.6	0.08	3.0	7.1	-0.19
Immunosuppressants	66.8	32.6	0.73	33.6	33.8	0.00
Lipid modifying agents	27.4	14.7	0.32	9.3	15.6	-0.18
Opioids	27.4	23.3	0.09	42.5	24.4	0.37
Psycholeptics	43.2	27.5	0.33	58.8	28.7	0.61
Psychostimulants, agents used for adhd and nootropics	<2.1	0.9	-0.06	<0.8	0.9	-0.11

S6.1.9. MDCD

Characteristic	Before stratification			After stratification		
	HCQ	SSZ		HCQ	SSZ	
	%	%	Std. diff	%	%	Std. diff
Age group						
15-19	1.2	1.2	0.00	1.2	1.3	-0.01
20-24	2.1	2.8	-0.04	2.1	3.1	-0.06
25-29	4.4	3.8	0.03	4.3	4.2	0.00
30-34	6.6	6.8	-0.01	6.4	6.9	-0.02
35-39	9.0	8.8	0.01	8.8	8.8	0.00
40-44	10.2	9.3	0.03	10.0	9.1	0.03
45-49	13.3	13.0	0.01	13.2	13.7	-0.01
50-54	15.9	16.1	0.00	16.2	15.9	0.01
55-59	15.8	16.2	-0.01	15.9	15.5	0.01
60-64	12.0	13.9	-0.06	12.3	12.9	-0.02
65-69	4.5	4.0	0.02	4.6	4.2	0.02
70-74	2.0	1.9	0.01	2.0	1.9	0.00
75-79	1.6	1.0	0.06	1.6	1.0	0.05
80-84	1.0	1.0	0.00	1.0	1.0	0.00
85-89	0.4	<0.2	0.04	0.4	0.3	0.01
Gender: female	85.9	79.0	0.18	84.4	84.5	0.00
Race						
race = Black or African American	28.8	24.7	0.09	27.9	26.2	0.04
race = White	54.4	57.4	-0.06	55.1	54.9	0.00
Ethnicity						
ethnicity = Hispanic or Latino	2.3	1.9	0.03	2.4	2.1	0.02
Medical history: General						
Acute respiratory disease	41.8	40.5	0.03	41.5	41.6	0.00
Attention deficit hyperactivity disorder	2.6	2.0	0.04	2.6	2.1	0.03
Chronic liver disease	6.9	7.4	-0.02	6.9	7.4	-0.02
Chronic obstructive lung disease	21.0	21.6	-0.02	21.2	21.5	-0.01
Crohn's disease	0.8	3.5	-0.19	1.0	1.9	-0.08
Dementia	1.2	0.9	0.03	1.1	1.1	0.01
Depressive disorder	36.0	35.4	0.01	35.5	35.8	-0.01
Gastroesophageal reflux disease	29.5	30.8	-0.03	29.5	30.7	-0.03
Gastrointestinal hemorrhage	4.9	6.4	-0.06	5.0	5.9	-0.04
Human immunodeficiency virus infection	0.5	0.7	-0.02	0.5	0.8	-0.03
Hyperlipidemia	36.4	36.7	-0.01	36.4	38.3	-0.04
Hypertensive disorder	55.7	53.0	0.05	55.2	54.6	0.01
Lesion of liver	2.0	1.9	0.01	2.0	2.2	-0.01
Obesity	21.5	20.0	0.04	21.2	20.2	0.02
Osteoarthritis	57.1	57.0	0.00	57.4	57.6	0.00
Pneumonia	8.7	8.0	0.02	8.5	8.0	0.02
Psoriasis	2.0	6.6	-0.23	2.5	3.4	-0.06
Renal impairment	9.4	8.0	0.05	9.2	8.3	0.03
Rheumatoid arthritis	80.9	84.4	-0.09	81.9	83.4	-0.04
Schizophrenia	1.7	2.1	-0.03	1.6	2.3	-0.05
Ulcerative colitis	0.5	1.7	-0.11	0.6	1.2	-0.06
Urinary tract infectious disease	17.8	16.5	0.04	17.4	16.6	0.02
Viral hepatitis C	5.9	6.0	0.00	5.9	6.3	-0.02
Visual system disorder	39.9	40.7	-0.02	40.0	39.9	0.00
Medical history: Cardiovascular disease						
Atrial fibrillation	3.3	2.3	0.06	3.2	3.2	0.00
Cerebrovascular disease	5.3	5.2	0.00	5.1	5.3	-0.01
Coronary arteriosclerosis	9.7	8.7	0.04	9.6	10.1	-0.02
Heart disease	29.7	26.8	0.07	29.0	28.8	0.00
Ischemic heart disease	7.3	7.0	0.01	7.1	8.0	-0.03
Peripheral vascular disease	4.8	4.1	0.03	4.6	4.3	0.02
Pulmonary embolism	2.0	1.0	0.08	1.9	1.6	0.02
Venous thrombosis	3.1	2.5	0.04	2.9	3.2	-0.02
Medical history: Neoplasms						

Characteristic	Before stratification			After stratification		
	HCQ	SSZ		HCQ	SSZ	
	%	%	Std. diff	%	%	Std. diff
Hematologic neoplasm	1.5	1.2	0.03	1.5	1.2	0.02
Malignant lymphoma	0.4	0.5	0.00	0.4	0.3	0.01
Malignant neoplasm of anorectum	0.2	0.2	-0.02	0.2	<0.2	0.00
Malignant neoplastic disease	7.2	7.5	-0.01	7.1	7.4	-0.02
Malignant tumor of breast	2.0	1.7	0.02	1.9	1.6	0.02
Malignant tumor of colon	0.3	0.3	0.00	0.4	0.3	0.01
Malignant tumor of lung	0.4	0.3	0.02	0.4	0.3	0.02
Malignant tumor of urinary bladder	0.2	<0.2	0.02	0.2	<0.2	0.04
Primary malignant neoplasm of prostate	0.2	0.2	-0.01	0.2	<0.2	0.01
Medication use						
Agents acting on the renin-angiotensin system	32.6	32.2	0.01	32.5	32.9	-0.01
Antibacterials for systemic use	50.4	48.5	0.04	50.1	47.8	0.04
Antidepressants	53.7	53.0	0.01	53.5	53.0	0.01
Antiepileptics	43.0	41.7	0.02	42.7	42.3	0.01
Antiinflammatory and antirheumatic products	58.2	60.0	-0.04	58.4	59.8	-0.03
Antineoplastic agents	30.7	41.0	-0.22	33.9	32.0	0.04
Antipsoriatics	0.8	0.7	0.01	0.8	0.6	0.03
Antithrombotic agents	18.8	17.0	0.05	18.5	17.9	0.02
Beta blocking agents	24.9	22.9	0.05	24.4	23.3	0.03
Calcium channel blockers	20.0	19.2	0.02	19.7	20.4	-0.02
Diuretics	33.7	32.1	0.04	33.3	32.7	0.02
Drugs for acid related disorders	50.0	51.2	-0.02	50.3	48.0	0.05
Drugs for obstructive airway diseases	46.2	46.9	-0.01	46.2	46.5	-0.01
Drugs used in diabetes	18.5	19.6	-0.03	18.4	19.1	-0.02
Immunosuppressants	39.8	56.0	-0.33	44.4	41.8	0.05
Lipid modifying agents	29.2	30.0	-0.02	29.3	30.9	-0.04
Opioids	62.1	62.6	-0.01	62.0	62.7	-0.01
Psycholeptics	49.8	49.5	0.00	49.3	50.0	-0.01
Psychostimulants, agents used for adhd and nootropics	7.4	6.7	0.02	7.2	6.9	0.01

S6.1.10. MDCR

Characteristic	Before stratification			After stratification		
	HCQ	SSZ		HCQ	SSZ	
	%	%	Std. diff	%	%	Std. diff
Age group						
45-49	0.1	<0.1	0.01	0.1	<0.1	0.01
55-59	0.7	1.0	-0.03	0.8	0.8	-0.01
60-64	1.7	2.1	-0.03	1.8	2.0	-0.02
65-69	23.7	26.3	-0.06	24.4	24.4	0.00
70-74	28.1	30.9	-0.06	28.6	28.8	0.00
75-79	22.6	20.6	0.05	22.2	21.9	0.01
80-84	14.6	13.2	0.04	14.3	14.2	0.00
90-94	1.3	1.2	0.02	1.3	1.3	0.00
95-99	0.1	0.1	0.01	0.1	0.1	0.01
Gender: female	72.9	68.6	0.10	71.8	71.5	0.00
Medical history: General						
Acute respiratory disease	27.5	26.4	0.02	27.4	27.2	0.00
Attention deficit hyperactivity disorder	0.2	0.2	0.00	0.2	0.2	0.00
Chronic liver disease	2.1	1.9	0.02	2.1	1.8	0.02
Chronic obstructive lung disease	15.8	16.0	-0.01	15.7	16.0	-0.01
Crohn's disease	0.5	1.3	-0.08	0.6	1.1	-0.05
Dementia	2.0	1.7	0.03	2.0	1.8	0.01
Depressive disorder	9.2	9.2	0.00	9.2	9.2	0.00
Diabetes mellitus	23.1	22.6	0.01	22.9	22.7	0.00
Gastroesophageal reflux disease	16.5	16.6	0.00	16.2	16.5	-0.01
Gastrointestinal hemorrhage	4.8	5.1	-0.01	4.8	4.9	-0.01
Human immunodeficiency virus infection	0.1	<0.1	0.01	0.1	<0.1	0.01
Hyperlipidemia	41.3	39.2	0.04	40.7	41.3	-0.01
Hypertensive disorder	61.8	59.3	0.05	61.1	61.3	0.00
Lesion of liver	1.0	1.0	-0.01	1.0	1.0	-0.01
Obesity	5.6	5.4	0.01	5.5	5.4	0.01
Osteoarthritis	62.0	59.5	0.05	61.2	61.9	-0.01
Pneumonia	9.2	9.2	0.00	9.2	9.1	0.00
Psoriasis	2.3	4.9	-0.14	2.7	3.3	-0.04
Renal impairment	11.3	10.5	0.03	11.1	11.2	0.00
Rheumatoid arthritis	88.4	89.7	-0.04	88.9	89.3	-0.02
Schizophrenia	0.1	<0.1	0.00	0.1	0.1	-0.01
Ulcerative colitis	0.6	2.0	-0.12	0.7	1.2	-0.05
Urinary tract infectious disease	13.6	12.7	0.03	13.5	12.7	0.02
Viral hepatitis C	0.5	0.6	-0.01	0.5	0.5	0.00
Visual system disorder	55.3	55.4	0.00	55.3	55.1	0.00
Medical history: Cardiovascular disease						
Atrial fibrillation	10.6	9.5	0.04	10.4	10.2	0.01
Heart failure	10.1	9.9	0.01	10.0	10.4	-0.01
Ischemic heart disease	10.1	10.1	0.00	10.1	10.4	-0.01
Peripheral vascular disease	6.4	5.5	0.04	6.3	6.0	0.01
Pulmonary embolism	1.5	1.3	0.02	1.5	1.3	0.02
Venous thrombosis	3.4	3.4	0.00	3.3	3.6	-0.01
Medical history: Neoplasms						
Hematologic neoplasm	2.2	2.3	0.00	2.2	2.4	-0.01
Malignant lymphoma	1.3	1.5	-0.02	1.3	1.4	-0.01
Malignant neoplasm of anorectum	0.4	0.4	0.00	0.4	0.4	0.00
Malignant neoplastic disease	18.5	19.8	-0.03	18.7	19.0	-0.01
Malignant tumor of breast	3.8	4.0	-0.01	3.8	3.8	0.00
Malignant tumor of colon	0.8	0.7	0.02	0.9	0.7	0.02
Malignant tumor of lung	0.7	0.6	0.00	0.7	0.5	0.02
Malignant tumor of urinary bladder	1.0	1.1	-0.02	1.0	0.9	0.00
Primary malignant neoplasm of prostate	2.4	2.8	-0.02	2.5	2.4	0.01
Medication use						
Antibacterials for systemic use	46.3	49.0	-0.05	47.0	46.3	0.01
Antidepressants	31.5	31.4	0.00	31.7	31.3	0.01

Characteristic	Before stratification			After stratification		
	HCQ	SSZ		HCQ	SSZ	
	%	%	Std. diff	%	%	Std. diff
Antiepileptics	20.1	21.8	-0.04	20.3	20.6	-0.01
Antiinflammatory and antirheumatic products	44.0	44.4	-0.01	44.1	44.8	-0.02
Antineoplastic agents	32.1	40.3	-0.17	34.8	33.3	0.03
Antipsoriatics	1.0	1.4	-0.03	1.0	1.3	-0.02
Antithrombotic agents	22.8	22.1	0.02	22.6	22.5	0.00
Beta blocking agents	39.4	39.3	0.00	39.3	39.2	0.00
Calcium channel blockers	30.0	28.6	0.03	29.6	30.3	-0.02
Diuretics	47.1	45.2	0.04	46.7	46.2	0.01
Drugs for acid related disorders	44.6	45.6	-0.02	44.7	45.1	-0.01
Drugs for obstructive airway diseases	33.2	32.4	0.02	33.2	32.8	0.01
Drugs used in diabetes	17.4	16.9	0.01	17.3	16.9	0.01
Immunosuppressants	41.2	54.9	-0.28	45.5	42.8	0.05
Lipid modifying agents	49.8	48.5	0.03	49.5	49.4	0.00
Opioids	43.6	44.0	-0.01	43.8	43.2	0.01
Psycholeptics	34.6	34.1	0.01	34.5	33.6	0.02
Psychostimulants, agents used for adhd and nootropics	2.0	2.0	0.00	2.0	1.9	0.01

S6.1.11. OpenClaims

Characteristic	Before stratification			After stratification		
	HCQ	SSZ		HCQ	SSZ	
	%	%	Std. diff	%	%	Std. diff
Age group						
15-19	0.3	0.3	-0.01	0.3	0.3	0.00
20-24	1.2	1.2	-0.01	1.2	1.2	0.00
25-29	1.9	1.9	0.00	1.9	1.8	0.00
30-34	3.0	2.9	0.00	2.9	2.9	0.00
35-39	4.3	4.4	-0.01	4.3	4.3	0.00
40-44	5.8	5.9	0.00	5.8	5.7	0.00
45-49	8.3	8.3	0.00	8.2	8.1	0.00
50-54	11.5	11.9	-0.01	11.5	11.5	0.00
55-59	13.6	14.2	-0.02	13.7	13.8	0.00
60-64	13.4	13.8	-0.01	13.6	13.5	0.00
70-74	9.9	9.6	0.01	9.9	10.0	0.00
75-79	10.7	9.0	0.06	10.3	10.6	-0.01
80-84	3.7	3.5	0.01	3.6	3.6	0.00
85-89	0.2	0.2	0.00	0.2	0.2	0.00
Gender: female	81.0	73.5	0.18	79.3	79.1	0.00
Medical history: General						
Acute respiratory disease	20.1	19.2	0.02	19.9	19.9	0.00
Chronic liver disease	2.1	2.0	0.00	2.0	2.1	0.00
Chronic obstructive lung disease	7.6	7.7	0.00	7.6	7.8	0.00
Crohn's disease	0.4	1.6	-0.11	0.5	0.9	-0.04
Dementia	0.8	0.7	0.01	0.8	0.8	0.00
Depressive disorder	9.7	9.6	0.00	9.6	9.8	-0.01
Diabetes mellitus	15.6	16.1	-0.01	15.7	16.0	-0.01
Gastrointestinal hemorrhage	2.4	2.5	-0.01	2.4	2.4	0.00
Hyperlipidemia	26.0	25.8	0.01	26.0	26.4	-0.01
Hypertensive disorder	35.0	34.5	0.01	34.9	35.2	-0.01
Lesion of liver	0.8	0.7	0.01	0.8	0.8	0.00
Obesity	6.3	6.6	-0.01	6.4	6.5	0.00
Osteoarthritis	36.0	37.0	-0.02	36.1	36.8	-0.02
Psoriasis	1.9	5.4	-0.19	2.2	3.3	-0.06
Renal impairment	6.2	5.5	0.03	6.1	6.0	0.00
Rheumatoid arthritis	69.0	69.6	-0.01	69.3	69.8	-0.01
Schizophrenia	0.2	0.2	0.00	0.2	0.2	0.00
Ulcerative colitis	0.4	1.7	-0.12	0.5	1.0	-0.06
Viral hepatitis C	1.1	1.1	0.00	1.1	1.1	0.00
Visual system disorder	21.9	20.7	0.03	21.6	21.6	0.00
Medical history: Cardiovascular disease						
Atrial fibrillation	3.6	3.4	0.01	3.6	3.6	0.00
Cerebrovascular disease	3.4	2.8	0.03	3.2	3.3	0.00
Coronary arteriosclerosis	6.8	6.8	0.00	6.8	6.9	0.00
Heart disease	18.3	17.3	0.03	18.1	18.3	0.00
Heart failure	4.0	3.7	0.02	3.9	4.0	0.00
Ischemic heart disease	3.5	3.4	0.01	3.5	3.6	0.00
Peripheral vascular disease	9.7	9.1	0.02	9.6	9.6	0.00
Venous thrombosis	1.5	1.4	0.01	1.5	1.5	0.00
Medical history: Neoplasms						
Hematologic neoplasm	0.9	0.9	0.00	0.9	1.0	0.00
Malignant neoplasm of anorectum	0.1	0.1	0.00	0.1	0.1	0.00
Malignant neoplastic disease	7.2	7.2	0.00	7.2	7.3	0.00
Malignant tumor of breast	1.8	1.6	0.01	1.7	1.7	0.00
Malignant tumor of lung	0.3	0.3	0.00	0.3	0.3	0.00
Malignant tumor of urinary bladder	0.2	0.3	-0.01	0.3	0.3	0.00
Medication use						
Agents acting on the renin-angiotensin system	30.8	33.0	-0.05	31.4	31.4	0.00
Antibacterials for systemic use	36.1	38.8	-0.06	36.8	36.6	0.00
Antidepressants	34.5	35.7	-0.03	34.7	34.8	0.00

Characteristic	Before stratification			After stratification		
	HCQ	SSZ		HCQ	SSZ	
	%	%	Std. diff	%	%	Std. diff
Antiepileptics	24.0	26.4	-0.05	24.5	24.6	0.00
Antiinflammatory and antirheumatic products	41.0	45.2	-0.08	42.0	41.9	0.00
Antineoplastic agents	31.6	38.0	-0.14	33.3	33.4	0.00
Antipsoriatics	0.7	1.0	-0.03	0.8	0.9	-0.01
Beta blocking agents	23.1	24.1	-0.02	23.3	23.5	0.00
Drugs used in diabetes	14.0	15.4	-0.04	14.3	14.6	-0.01
Immunosuppressants	41.9	53.4	-0.23	44.7	44.7	0.00
Lipid modifying agents	29.0	31.5	-0.05	29.6	29.8	0.00
Opioids	34.8	37.8	-0.06	35.4	35.5	0.00
Psycholeptics	29.5	30.8	-0.03	29.7	29.9	0.00
Psychostimulants, agents used for adhd and nootropics	4.5	4.8	-0.01	4.5	4.6	0.00

S6.1.12. OptumEHR

Characteristic	Before stratification			After stratification		
	HCQ	SSZ		HCQ	SSZ	
	%	%	Std. diff	%	%	Std. diff
Age group						
15-19	0.2	0.3	-0.02	0.2	0.3	-0.01
20-24	1.0	1.0	0.00	1.0	1.0	0.00
25-29	1.8	1.9	-0.01	1.8	1.9	-0.01
30-34	3.1	3.0	0.00	3.1	3.0	0.00
35-39	4.2	4.6	-0.02	4.2	4.2	0.00
40-44	5.9	5.8	0.01	5.9	5.6	0.01
45-49	7.9	8.2	-0.01	7.9	7.9	0.00
50-54	11.5	11.9	-0.01	11.6	11.5	0.00
55-59	13.8	14.7	-0.03	14.0	14.3	-0.01
60-64	13.3	13.9	-0.02	13.5	13.4	0.00
65-69	12.1	12.7	-0.02	12.3	12.6	-0.01
70-74	10.1	9.6	0.02	10.0	9.9	0.00
75-79	8.1	6.9	0.04	7.8	7.9	0.00
80-84	5.5	4.2	0.06	5.2	5.3	-0.01
85-89	1.6	1.2	0.03	1.5	1.4	0.00
Gender: female	80.1	72.4	0.18	78.2	78.6	-0.01
Race						
race = Asian	1.5	1.1	0.04	1.5	1.4	0.01
race = Black or African American	10.1	8.4	0.06	9.7	9.6	0.00
race = White	81.4	83.3	-0.05	81.8	81.9	0.00
Ethnicity						
ethnicity = Hispanic or Latino	5.3	6.1	-0.04	5.4	5.4	0.00
Medical history: General						
Acute respiratory disease	17.9	18.3	-0.01	17.9	18.1	0.00
Attention deficit hyperactivity disorder	1.0	0.8	0.01	0.9	0.8	0.01
Chronic liver disease	2.7	3.0	-0.02	2.8	2.9	-0.01
Chronic obstructive lung disease	8.5	9.2	-0.03	8.6	8.9	-0.01
Crohn's disease	0.5	1.6	-0.12	0.6	0.9	-0.04
Dementia	1.1	0.9	0.02	1.0	1.1	-0.01
Depressive disorder	15.7	15.4	0.01	15.6	15.7	0.00
Diabetes mellitus	14.9	15.0	0.00	15.0	15.3	-0.01
Gastroesophageal reflux disease	18.4	18.1	0.01	18.3	18.1	0.00
Gastrointestinal hemorrhage	2.1	2.4	-0.02	2.1	2.2	0.00
Human immunodeficiency virus infection	0.1	0.2	-0.01	0.1	0.2	-0.01
Hyperlipidemia	30.4	31.1	-0.02	30.5	30.6	0.00
Hypertensive disorder	38.9	38.6	0.01	38.8	39.2	-0.01
Lesion of liver	1.1	0.9	0.02	1.0	1.0	0.01
Obesity	11.3	12.1	-0.03	11.4	11.6	-0.01
Osteoarthritis	35.1	36.2	-0.02	35.3	35.7	-0.01
Pneumonia	5.0	5.1	0.00	5.0	5.0	0.00
Psoriasis	1.7	4.4	-0.16	2.1	2.7	-0.04
Renal impairment	7.7	6.7	0.04	7.4	7.7	-0.01
Rheumatoid arthritis	84.8	84.7	0.00	84.9	85.5	-0.02
Ulcerative colitis	0.4	1.5	-0.11	0.5	0.8	-0.04
Urinary tract infectious disease	7.6	6.3	0.05	7.2	7.1	0.00
Viral hepatitis C	1.2	1.5	-0.02	1.3	1.4	-0.01
Visual system disorder	13.8	13.6	0.00	13.7	13.4	0.01
Medical history: Cardiovascular disease						
Coronary arteriosclerosis	8.3	8.5	0.00	8.3	8.5	-0.01
Heart disease	20.9	20.2	0.02	20.7	21.1	-0.01
Heart failure	4.7	4.4	0.02	4.7	4.9	-0.01
Ischemic heart disease	4.4	4.5	0.00	4.4	4.5	0.00
Peripheral vascular disease	2.6	2.4	0.01	2.6	2.5	0.00
Pulmonary embolism	1.0	1.0	0.00	1.0	1.0	-0.01
Venous thrombosis	1.9	2.0	-0.01	1.9	2.0	-0.01
Medical history: Neoplasms						

Characteristic	Before stratification			After stratification		
	HCQ	SSZ		HCQ	SSZ	
	%	%	Std. diff	%	%	Std. diff
Hematologic neoplasm	1.1	1.2	-0.01	1.1	1.2	-0.01
Malignant lymphoma	0.6	0.7	-0.01	0.6	0.7	0.00
Malignant neoplasm of anorectum	0.2	0.2	0.01	0.2	0.2	0.01
Malignant neoplastic disease	8.5	8.8	-0.01	8.5	8.8	-0.01
Malignant tumor of breast	1.9	1.7	0.02	1.9	1.9	0.00
Malignant tumor of colon	0.4	0.3	0.00	0.4	0.4	0.00
Malignant tumor of lung	0.4	0.4	-0.01	0.4	0.4	-0.01
Malignant tumor of urinary bladder	0.2	0.4	-0.03	0.2	0.3	-0.02
Primary malignant neoplasm of prostate	0.6	0.8	-0.03	0.7	0.7	0.00
Medication use						
Agents acting on the renin-angiotensin system	30.6	31.6	-0.02	30.8	31.3	-0.01
Antibacterials for systemic use	28.1	29.8	-0.04	28.3	28.2	0.00
Antidepressants	36.0	35.6	0.01	35.8	36.3	-0.01
Antiepileptics	24.0	24.8	-0.02	24.2	24.4	-0.01
Antiinflammatory and antirheumatic products	43.7	47.2	-0.07	44.5	44.6	0.00
Antineoplastic agents	34.4	38.1	-0.08	35.4	36.4	-0.02
Antipsoriatics	0.6	0.7	-0.01	0.6	0.6	0.00
Antithrombotic agents	33.0	31.7	0.03	32.6	33.2	-0.01
Beta blocking agents	26.7	27.0	-0.01	26.8	27.2	-0.01
Calcium channel blockers	17.2	16.7	0.01	17.1	17.5	-0.01
Diuretics	29.2	28.3	0.02	29.1	29.4	-0.01
Drugs for acid related disorders	45.1	45.5	-0.01	45.1	45.4	0.00
Drugs for obstructive airway diseases	34.5	35.2	-0.02	34.6	35.0	-0.01
Drugs used in diabetes	15.1	15.7	-0.02	15.3	15.8	-0.01
Immunosuppressants	47.1	56.1	-0.18	49.2	50.2	-0.02
Lipid modifying agents	30.2	31.7	-0.03	30.6	31.0	-0.01
Opioids	39.3	41.1	-0.04	39.6	40.3	-0.01
Psycholeptics	35.2	35.0	0.00	35.1	35.5	-0.01
Psychostimulants, agents used for adhd and nootropics	4.3	4.0	0.01	4.2	4.1	0.01

S6.1.13. SIDIAP

Characteristic	Before stratification			After stratification		
	HCQ	SSZ		HCQ	SSZ	
	%	%	Std. diff	%	%	Std. diff
Age group						
20-24	0.6	<1.3	-0.02	0.6	<1.0	-0.02
25-29	2.2	2.9	-0.05	2.0	4.6	-0.15
30-34	4.4	4.7	-0.02	4.6	4.3	0.01
35-39	7.1	9.7	-0.09	7.2	13.3	-0.20
40-44	8.0	6.8	0.05	8.3	5.6	0.11
45-49	7.5	9.4	-0.07	7.4	7.3	0.00
50-54	10.1	12.3	-0.07	10.5	7.7	0.10
55-59	11.5	9.4	0.07	11.5	9.5	0.06
60-64	11.2	10.8	0.02	10.9	11.1	-0.01
65-69	10.8	10.2	0.02	11.1	8.7	0.08
70-74	9.9	6.8	0.11	9.6	9.6	0.00
75-79	7.5	9.4	-0.07	7.6	9.1	-0.05
80-84	6.2	5.8	0.02	6.0	8.0	-0.08
85-89	2.0	<1.3	0.13	2.0	<1.0	0.16
90-94	0.6	<1.3	0.06	0.6	<1.0	0.06
Gender: female	79.2	66.7	0.29	75.9	79.8	-0.09
Medical history: General						
Acute respiratory disease	17.9	17.3	0.02	17.4	26.6	-0.22
Chronic liver disease	1.0	<1.3	-0.01	1.0	<1.0	0.04
Chronic obstructive lung disease	2.6	2.6	0.00	2.4	1.7	0.05
Depressive disorder	3.0	4.7	-0.09	3.1	9.0	-0.25
Diabetes mellitus	2.9	3.1	-0.02	2.8	2.3	0.03
Gastroesophageal reflux disease	1.0	1.3	-0.02	1.1	1.5	-0.04
Gastrointestinal hemorrhage	2.7	3.7	-0.05	2.9	3.6	-0.04
Hyperlipidemia	4.3	3.4	0.04	4.0	7.8	-0.16
Hypertensive disorder	5.3	6.6	-0.05	5.7	7.9	-0.09
Obesity	4.8	4.2	0.03	4.6	3.3	0.07
Osteoarthritis	3.0	3.1	-0.01	3.2	4.9	-0.09
Pneumonia	2.0	2.4	-0.02	2.2	7.3	-0.24
Psoriasis	0.7	2.1	-0.12	0.6	2.0	-0.12
Renal impairment	1.8	2.4	-0.04	2.0	2.1	-0.01
Rheumatoid arthritis	35.6	26.5	0.20	33.2	42.3	-0.19
Urinary tract infectious disease	7.5	5.8	0.07	7.4	6.0	0.06
Viral hepatitis C	0.9	<1.3	0.04	0.9	<1.0	0.07
Visual system disorder	16.0	17.3	-0.03	15.6	19.5	-0.10
Medical history: Cardiovascular disease						
Atrial fibrillation	0.6	1.3	-0.08	0.5	4.1	-0.24
Cerebrovascular disease	2.2	<1.3	0.09	2.2	<1.0	0.13
Heart disease	7.2	6.8	0.02	7.0	9.2	-0.08
Heart failure	1.1	<1.3	0.01	1.1	3.3	-0.15
Ischemic heart disease	1.6	<1.3	0.10	1.5	<1.0	0.13
Peripheral vascular disease	3.6	2.4	0.07	3.8	1.7	0.13
Pulmonary embolism	0.4	<1.3	0.02	0.4	<1.0	0.04
Medical history: Neoplasms						
Hematologic neoplasm	<0.4	<1.3	-0.08	<0.4	<1.0	-0.06
Malignant neoplastic disease	3.0	3.4	-0.02	3.0	2.4	0.04
Malignant tumor of breast	0.4	<1.3	-0.02	<0.4	<1.0	-0.02
Malignant tumor of colon	<0.4	<1.3	0.01	<0.4	<1.0	0.04
Malignant tumor of lung	<0.4	<1.3	-0.04	<0.4	<1.0	-0.03
Malignant tumor of urinary bladder	<0.4	<1.3	-0.05	<0.4	<1.0	-0.02
Medication use						
Agents acting on the renin-angiotensin system	26.2	30.7	-0.10	27.2	27.0	0.00
Antibacterials for systemic use	17.4	17.8	-0.01	17.1	19.2	-0.05
Antidepressants	21.9	22.8	-0.02	21.9	27.7	-0.13
Antiepileptics	9.9	11.8	-0.06	9.9	11.9	-0.06
Antiinflammatory and antirheumatic products	70.0	71.7	-0.04	69.8	68.2	0.03

Characteristic	Before stratification			After stratification		
	HCQ	SSZ		HCQ	SSZ	
	%	%	Std. diff	%	%	Std. diff
Antineoplastic agents	21.3	21.0	0.01	21.3	20.6	0.02
Antipsoriatics	1.3	1.6	-0.02	1.4	<1.0	0.04
Antithrombotic agents	17.4	16.3	0.03	17.3	15.6	0.05
Beta blocking agents	11.2	10.5	0.02	11.5	10.1	0.04
Calcium channel blockers	10.3	6.0	0.16	9.8	9.7	0.00
Diuretics	22.9	21.8	0.03	22.6	19.2	0.08
Drugs for acid related disorders	77.9	83.5	-0.14	78.8	78.0	0.02
Drugs for obstructive airway diseases	24.7	25.5	-0.02	24.2	21.1	0.07
Drugs used in diabetes	10.5	10.8	-0.01	10.6	9.2	0.05
Immunosuppressants	32.6	36.2	-0.08	33.1	30.3	0.06
Lipid modifying agents	25.3	21.5	0.09	24.5	23.3	0.03
Opioids	22.6	23.4	-0.02	22.9	23.5	-0.01
Psycholeptics	36.3	40.7	-0.09	37.1	44.2	-0.14
Psychostimulants, agents used for adhd and nootropics	1.3	3.1	-0.13	1.3	2.0	-0.05

S6.1.14. VA

Characteristic	Before stratification			After stratification		
	HCQ	SSZ		HCQ	SSZ	
	%	%	Std. diff	%	%	Std. diff
Age group						
20-24	0.1	0.1	0.00	0.1	0.2	-0.02
25-29	0.6	0.6	0.00	0.6	0.6	-0.01
30-34	1.2	1.0	0.02	1.1	1.1	0.00
35-39	1.6	1.7	0.00	1.5	1.7	-0.02
40-44	2.7	3.0	-0.02	2.7	2.8	-0.01
45-49	5.1	5.1	0.00	5.1	5.0	0.00
50-54	8.4	9.7	-0.05	8.8	8.7	0.00
55-59	12.8	14.5	-0.05	13.4	13.1	0.01
60-64	17.1	17.8	-0.02	17.3	17.6	-0.01
65-69	16.9	17.0	0.00	17.1	17.0	0.00
70-74	13.8	13.7	0.00	13.9	13.7	0.00
75-79	10.0	9.1	0.03	9.8	9.7	0.00
80-84	6.5	4.7	0.08	5.9	6.2	-0.01
85-89	2.5	1.6	0.06	2.2	2.2	0.00
90-94	0.5	0.3	0.03	0.5	0.3	0.03
Gender: female	14.5	10.4	0.13	13.2	13.1	0.00
Race						
race = Asian	0.5	0.4	0.00	0.5	0.4	0.01
race = Black or African American	14.4	12.9	0.04	13.9	13.6	0.01
race = White	73.6	74.6	-0.02	73.9	74.1	0.00
race = Unknown	9.9	10.4	-0.02	10.1	10.2	0.00
race = Native Hawaiian or Other Pacific Islander	0.7	0.8	-0.01	0.7	0.8	-0.02
race = American Indian or Alaska Native	0.9	0.9	0.00	0.9	0.8	0.01
Ethnicity						
ethnicity = Hispanic or Latino	4.8	4.9	0.00	4.9	4.7	0.00
ethnicity = Not Hispanic or Latino	89.3	88.6	0.02	89.0	89.1	0.00
Medical history: General						
Acute respiratory disease	11.4	12.3	-0.03	11.8	11.3	0.02
Attention deficit hyperactivity disorder	0.4	0.4	0.00	0.4	0.5	-0.01
Chronic liver disease	3.4	3.4	0.00	3.5	3.6	0.00
Crohn's disease	0.3	0.9	-0.07	0.4	0.6	-0.02
Dementia	1.2	1.0	0.02	1.1	1.1	0.00
Depressive disorder	21.9	21.6	0.01	21.8	21.6	0.00
Diabetes mellitus	24.5	23.8	0.02	24.1	24.1	0.00
Gastroesophageal reflux disease	5.8	5.4	0.01	5.6	5.5	0.01
Gastrointestinal hemorrhage	2.1	2.8	-0.04	2.3	2.3	0.00
Human immunodeficiency virus infection	0.2	0.2	0.00	0.2	0.2	0.00
Hypertensive disorder	58.1	57.6	0.01	58.0	58.0	0.00
Lesion of liver	1.4	1.4	0.00	1.4	1.4	0.01
Obesity	14.3	14.3	0.00	14.2	14.3	0.00
Psoriasis	2.2	7.0	-0.23	3.2	4.1	-0.05
Renal impairment	7.2	6.6	0.02	7.0	7.1	0.00
Rheumatoid arthritis	90.4	89.6	0.02	90.5	90.2	0.01
Schizophrenia	0.6	0.7	-0.01	0.6	0.6	0.01
Ulcerative colitis	0.4	1.0	-0.06	0.5	0.7	-0.02
Urinary tract infectious disease	3.5	3.1	0.02	3.4	3.1	0.02
Viral hepatitis C	3.7	3.7	0.00	3.7	3.8	0.00
Visual system disorder	38.5	41.9	-0.07	39.5	38.7	0.02
Medical history: Cardiovascular disease						
Atrial fibrillation	6.1	5.4	0.03	5.9	5.8	0.00
Cerebrovascular disease	4.3	4.0	0.02	4.3	4.3	0.00
Coronary arteriosclerosis	14.7	15.1	-0.01	14.8	15.2	-0.01
Heart disease	30.5	30.4	0.00	30.6	31.1	-0.01
Heart failure	5.9	6.1	-0.01	5.9	6.1	-0.01
Ischemic heart disease	11.0	11.2	0.00	11.1	11.1	0.00
Pulmonary embolism	0.7	0.8	0.00	0.7	0.8	-0.01

Characteristic	Before stratification			After stratification		
	HCQ	SSZ		HCQ	SSZ	
	%	%	Std. diff	%	%	Std. diff
Venous thrombosis	1.4	1.3	0.01	1.4	1.3	0.01
Medical history: Neoplasms						
Hematologic neoplasm	1.1	1.0	0.02	1.1	1.0	0.01
Malignant lymphoma	0.8	0.6	0.01	0.8	0.6	0.02
Malignant neoplasm of anorectum	0.1	0.1	0.00	0.2	0.1	0.01
Malignant tumor of breast	0.4	0.2	0.02	0.3	0.3	0.01
Malignant tumor of colon	0.6	0.6	0.00	0.6	0.6	0.00
Malignant tumor of lung	0.3	0.4	-0.01	0.4	0.4	0.00
Malignant tumor of urinary bladder	0.8	0.8	-0.01	0.7	0.9	-0.01
Medication use						
Agents acting on the renin-angiotensin system	38.5	39.2	-0.01	38.8	39.0	0.00
Antibacterials for systemic use	22.8	25.3	-0.06	23.8	23.2	0.01
Antidepressants	35.0	36.1	-0.02	35.3	34.8	0.01
Antiepileptics	21.3	20.9	0.01	21.1	21.0	0.00
Antiinflammatory and antirheumatic products	47.8	52.1	-0.09	49.2	47.8	0.03
Antineoplastic agents	36.8	41.7	-0.10	38.8	38.2	0.01
Antipsoriatics	1.0	2.0	-0.09	1.1	1.5	-0.03
Antithrombotic agents	28.6	28.9	-0.01	28.8	28.6	0.01
Beta blocking agents	32.0	32.2	0.00	32.0	32.1	0.00
Calcium channel blockers	21.8	21.8	0.00	21.9	22.3	-0.01
Diuretics	32.8	33.5	-0.02	33.1	33.2	0.00
Drugs for acid related disorders	52.3	55.9	-0.07	53.5	52.8	0.01
Drugs for obstructive airway diseases	27.8	28.9	-0.02	28.2	28.0	0.00
Drugs used in diabetes	19.7	19.4	0.01	19.4	19.6	0.00
Immunosuppressants	44.0	51.9	-0.16	47.0	46.3	0.01
Lipid modifying agents	45.7	45.1	0.01	45.5	45.6	0.00
Opioids	31.0	34.1	-0.06	32.0	31.5	0.01
Psycholeptics	23.7	24.8	-0.03	24.0	24.0	0.00
Psychostimulants, agents used for adhd and nootropics	1.8	1.6	0.02	1.8	1.6	0.01

S6.2. AZM vs AMX

S6.2.1. AmbEMR

Characteristic	Before stratification			After stratification		
	AZM	AMX		AZM	AMX	
	%	%	Std. diff	%	%	Std. diff
Age group						
20-24	0.8	0.7	0.01	0.8	0.7	0.01
25-29	1.2	1.3	-0.01	1.3	1.2	0.00
30-34	2.5	2.3	0.02	2.6	2.3	0.02
35-39	3.5	3.9	-0.02	3.7	3.7	0.00
40-44	5.8	5.1	0.03	5.8	5.4	0.02
45-49	7.5	7.3	0.01	7.7	7.3	0.01
50-54	10.1	10.4	-0.01	10.1	10.5	-0.01
55-59	13.4	13.7	-0.01	13.5	14.0	-0.01
60-64	14.1	14.4	-0.01	13.9	14.2	-0.01
65-69	13.8	14.1	-0.01	13.7	14.1	-0.01
70-74	12.7	12.1	0.02	12.2	11.9	0.01
75-79	10.6	10.1	0.02	10.6	10.3	0.01
80-84	3.9	4.5	-0.03	4.1	4.2	-0.01
Gender: female	83.2	82.5	0.02	83.2	82.9	0.01
Race						
race = Asian	1.5	1.1	0.03	1.4	1.1	0.02
race = White	75.6	75.3	0.01	75.7	75.6	0.00
race = African American	8.2	8.1	0.00	8.2	8.1	0.00
Ethnicity						
ethnicity = Hispanic or Latino	1.5	1.2	0.03	1.4	1.2	0.02
ethnicity = Not Hispanic or Latino	85.2	84.5	0.02	85.3	84.8	0.02
Medical history: General						
Acute respiratory disease	43.3	34.9	0.17	39.9	40.4	-0.01
Attention deficit hyperactivity disorder	0.7	0.9	-0.03	0.7	0.9	-0.03
Chronic liver disease	1.5	1.7	-0.02	1.5	1.7	-0.02
Chronic obstructive lung disease	10.6	8.4	0.07	9.2	10.4	-0.04
Crohn's disease	0.4	0.6	-0.02	0.4	0.5	-0.01
Dementia	0.7	0.9	-0.03	0.7	0.9	-0.02
Depressive disorder	19.4	19.5	0.00	19.8	19.7	0.00
Diabetes mellitus	14.0	15.1	-0.03	14.6	14.9	-0.01
Gastroesophageal reflux disease	20.6	21.1	-0.01	20.9	21.3	-0.01
Gastrointestinal hemorrhage	1.6	1.8	-0.01	1.7	1.7	0.00
Human immunodeficiency virus infection	0.1	0.1	0.00	0.1	0.1	0.00
Hyperlipidemia	33.2	32.8	0.01	33.2	32.8	0.01
Hypertensive disorder	41.4	42.2	-0.02	41.8	41.6	0.00
Lesion of liver	0.8	1.1	-0.03	0.9	1.1	-0.02
Obesity	11.7	11.6	0.00	12.0	11.8	0.00
Osteoarthritis	28.3	29.7	-0.03	28.8	28.8	0.00
Pneumonia	6.5	5.0	0.06	5.7	6.5	-0.03
Psoriasis	1.6	1.8	-0.02	1.6	1.7	-0.01
Renal impairment	6.5	7.3	-0.03	6.7	7.2	-0.02
Rheumatoid arthritis	80.0	78.7	0.03	79.5	79.2	0.01
Schizophrenia	0.1	0.1	0.01	0.1	0.1	0.01
Ulcerative colitis	0.5	0.4	0.02	0.6	0.4	0.04
Urinary tract infectious disease	9.5	11.9	-0.08	10.4	10.5	-0.01
Viral hepatitis C	0.8	1.1	-0.03	0.8	1.0	-0.02
Visual system disorder	12.0	12.4	-0.01	12.2	12.2	0.00
Medical history: Cardiovascular disease						
Atrial fibrillation	3.3	4.0	-0.04	3.4	3.9	-0.03
Cerebrovascular disease	3.5	3.7	-0.01	3.6	3.4	0.01
Heart disease	17.5	18.4	-0.02	17.7	18.2	-0.01
Heart failure	3.5	3.6	-0.01	3.4	3.7	-0.02
Ischemic heart disease	1.9	2.0	0.00	2.0	1.9	0.01
Peripheral vascular disease	2.0	2.2	-0.01	2.0	2.0	0.00
Pulmonary embolism	1.0	1.0	0.00	1.0	1.0	0.00

Characteristic	Before stratification			After stratification		
	**AZM**	**AMX**		**AZM**	**AMX**	
	**%**	**%**	**Std. diff**	**%**	**%**	**Std. diff**
Venous thrombosis	1.6	1.9	-0.03	1.7	1.8	-0.01
Medical history: Neoplasms						
Hematologic neoplasm	0.6	0.4	0.02	0.5	0.5	0.00
Malignant neoplastic disease	7.4	7.7	-0.01	7.4	7.7	-0.01
Malignant tumor of breast	1.6	1.6	0.00	1.6	1.6	0.00
Malignant tumor of lung	0.5	0.4	0.02	0.4	0.5	-0.01
Malignant tumor of urinary bladder	0.2	0.3	-0.01	0.2	0.3	-0.01
Primary malignant neoplasm of prostate	0.3	0.2	0.03	0.3	0.2	0.02
Medication use						
Agents acting on the renin-angiotensin system	35.5	36.7	-0.02	35.8	36.0	0.00
Antibacterials for systemic use	41.3	44.0	-0.05	42.9	43.4	-0.01
Antidepressants	43.1	44.5	-0.03	44.2	44.2	0.00
Antiepileptics	28.9	30.3	-0.03	30.1	29.8	0.01
Antiinflammatory and antirheumatic products	46.2	47.0	-0.02	46.7	46.7	0.00
Antineoplastic agents	39.6	38.0	0.03	38.4	39.1	-0.02
Antipsoriatics	0.9	0.9	0.00	1.0	0.8	0.02
Antithrombotic agents	25.1	27.4	-0.05	25.9	26.1	0.00
Beta blocking agents	26.4	28.5	-0.05	27.4	27.6	0.00
Calcium channel blockers	19.5	20.2	-0.02	19.9	19.9	0.00
Diuretics	35.3	35.5	0.00	35.6	35.4	0.00
Drugs for acid related disorders	49.4	51.4	-0.04	50.5	50.9	-0.01
Drugs for obstructive airway diseases	53.3	45.1	0.16	49.3	50.8	-0.03
Drugs used in diabetes	14.6	15.9	-0.04	15.2	15.4	0.00
Immunosuppressants	55.9	54.7	0.02	55.1	55.5	-0.01
Lipid modifying agents	34.4	34.8	-0.01	34.9	34.5	0.01
Opioids	34.8	36.9	-0.04	36.1	35.4	0.02
Psychostimulants, agents used for adhd and nootropics	5.4	5.4	0.00	5.5	5.4	0.01

S6.2.2. CCAE

Characteristic	Before stratification			After stratification		
	AZM	AMX		AZM	AMX	
	%	%	Std. diff	%	%	Std. diff
Age group						
15-19	0.5	0.4	0.00	0.5	0.5	0.00
20-24	1.4	1.3	0.01	1.4	1.4	0.00
25-29	2.2	2.1	0.01	2.2	2.2	0.00
30-34	4.0	3.7	0.02	4.0	3.9	0.01
35-39	6.8	6.5	0.02	6.8	6.7	0.00
40-44	9.5	8.9	0.02	9.3	9.3	0.00
45-49	13.1	13.2	0.00	13.2	13.3	0.00
50-54	18.3	17.9	0.01	18.1	18.0	0.00
55-59	21.5	22.0	-0.01	21.5	21.8	-0.01
60-64	20.6	21.8	-0.03	21.1	21.1	0.00
65-69	2.0	2.1	0.00	2.0	2.0	0.00
Medical history: General						
Acute respiratory disease	62.5	50.7	0.24	58.0	57.5	0.01
Attention deficit hyperactivity disorder	1.4	1.5	-0.01	1.4	1.5	0.00
Chronic liver disease	2.7	2.9	-0.01	2.7	2.8	-0.01
Chronic obstructive lung disease	5.6	4.4	0.06	5.0	5.2	-0.01
Dementia	0.3	0.2	0.01	0.3	0.2	0.01
Depressive disorder	14.7	14.7	0.00	14.7	14.8	0.00
Diabetes mellitus	12.7	13.3	-0.02	13.2	13.1	0.00
Gastroesophageal reflux disease	16.5	16.8	-0.01	16.7	16.8	0.00
Human immunodeficiency virus infection	0.1	0.1	0.01	0.1	0.1	0.01
Hyperlipidemia	30.4	30.4	0.00	30.4	30.3	0.00
Lesion of liver	0.8	0.8	-0.01	0.8	0.8	0.00
Obesity	9.4	10.8	-0.05	10.2	10.1	0.00
Osteoarthritis	40.1	42.2	-0.04	41.5	41.0	0.01
Pneumonia	6.8	4.3	0.11	5.7	5.5	0.01
Psoriasis	3.1	3.0	0.00	3.0	3.0	0.00
Renal impairment	3.9	4.3	-0.02	4.2	4.1	0.00
Rheumatoid arthritis	83.6	82.2	0.04	82.9	83.0	0.00
Ulcerative colitis	0.7	0.7	0.00	0.7	0.7	0.00
Urinary tract infectious disease	13.6	14.3	-0.02	14.0	13.9	0.00
Viral hepatitis C	0.7	0.9	-0.02	0.8	0.9	-0.01
Visual system disorder	36.8	37.0	0.00	36.9	37.1	0.00
Medical history: Cardiovascular disease						
Atrial fibrillation	1.5	1.9	-0.03	1.7	1.8	0.00
Cerebrovascular disease	3.2	3.1	0.00	3.1	3.2	-0.01
Coronary arteriosclerosis	4.8	5.0	-0.01	5.0	4.9	0.00
Heart disease	17.3	18.2	-0.02	17.8	17.9	0.00
Heart failure	2.5	2.3	0.01	2.5	2.4	0.01
Ischemic heart disease	3.2	3.2	0.00	3.3	3.1	0.01
Peripheral vascular disease	1.6	1.7	-0.01	1.6	1.7	-0.01
Pulmonary embolism	0.9	1.0	-0.01	0.9	1.0	-0.01
Venous thrombosis	1.6	1.8	-0.01	1.7	1.8	0.00
Medical history: Neoplasms						
Hematologic neoplasm	1.2	1.1	0.01	1.2	1.1	0.00
Malignant lymphoma	0.7	0.6	0.00	0.6	0.6	0.00
Malignant neoplastic disease	7.6	7.5	0.00	7.6	7.5	0.00
Malignant tumor of colon	0.2	0.2	-0.01	0.2	0.2	0.00
Malignant tumor of lung	0.2	0.2	0.01	0.2	0.2	0.01
Malignant tumor of urinary bladder	0.2	0.1	0.00	0.1	0.1	0.00
Primary malignant neoplasm of prostate	0.2	0.3	-0.02	0.2	0.3	-0.01
Medication use						
Agents acting on the renin-angiotensin system	26.6	27.2	-0.01	27.1	26.9	0.00
Antibacterials for systemic use	50.6	50.3	0.00	51.1	51.0	0.00
Antidepressants	43.3	42.0	0.02	43.0	42.8	0.00
Antiinflammatory and antirheumatic products	51.0	53.1	-0.04	51.9	52.0	0.00

Characteristic	Before stratification			After stratification		
	AZM	AMX		AZM	AMX	
	%	%	Std. diff	%	%	Std. diff
Antipsoriatics	0.9	0.9	-0.01	0.9	0.9	0.00
Antithrombotic agents	8.9	10.8	-0.06	9.8	9.9	0.00
Beta blocking agents	18.1	18.6	-0.01	18.7	18.4	0.01
Calcium channel blockers	13.8	14.0	-0.01	14.1	13.9	0.00
Diuretics	28.0	28.0	0.00	28.3	28.3	0.00
Drugs for acid related disorders	40.7	40.1	0.01	40.5	40.8	-0.01
Drugs for obstructive airway diseases	43.6	37.0	0.13	41.1	40.7	0.01
Drugs used in diabetes	9.8	10.4	-0.02	10.3	10.2	0.00
Immunosuppressants	52.5	49.8	0.05	51.1	51.2	0.00
Lipid modifying agents	22.5	22.9	-0.01	22.7	22.6	0.00
Opioids	40.1	42.0	-0.04	41.4	41.2	0.00
Psycholeptics	38.6	37.4	0.02	38.2	38.1	0.00
Psychostimulants, agents used for adhd and nootropics	7.3	7.1	0.01	7.3	7.4	0.00

S6.2.3. Clinformatics

Characteristic	Before stratification			After stratification		
	AZM	AMX		AZM	AMX	
	%	%	Std. diff	%	%	Std. diff
Age group						
15-19	0.3	0.2	0.00	0.2	0.3	0.00
20-24	0.8	0.8	0.00	0.9	1.0	-0.01
25-29	1.5	1.4	0.01	1.5	1.4	0.01
30-34	3.1	2.7	0.02	3.0	2.8	0.01
35-39	4.4	4.2	0.01	4.3	4.4	-0.01
40-44	6.8	6.0	0.03	6.6	6.4	0.01
45-49	8.6	8.6	0.00	8.6	8.9	-0.01
55-59	14.0	13.5	0.01	13.9	13.6	0.01
60-64	13.3	13.2	0.00	13.3	13.0	0.01
65-69	12.1	12.8	-0.02	12.4	12.6	-0.01
70-74	10.2	10.8	-0.02	10.2	10.3	0.00
75-79	7.2	7.8	-0.02	7.2	7.4	-0.01
80-84	4.4	4.8	-0.02	4.4	4.5	-0.01
85-89	1.7	1.9	-0.02	1.8	1.8	0.00
Gender: female	84.9	82.9	0.06	84.0	83.9	0.00
Medical history: General						
Acute respiratory disease	64.9	48.7	0.33	57.7	57.7	0.00
Chronic obstructive lung disease	14.8	11.0	0.11	13.0	13.1	0.00
Crohn's disease	0.7	0.7	0.01	0.8	0.6	0.02
Dementia	1.5	1.9	-0.03	1.7	1.9	-0.02
Depressive disorder	20.7	21.4	-0.02	21.3	21.2	0.00
Diabetes mellitus	20.4	20.7	-0.01	20.4	20.7	-0.01
Gastroesophageal reflux disease	25.6	26.1	-0.01	26.1	25.8	0.01
Gastrointestinal hemorrhage	4.2	4.5	-0.02	4.4	4.5	0.00
Human immunodeficiency virus infection	0.2	0.2	0.00	0.2	0.2	0.00
Hyperlipidemia	46.3	46.7	-0.01	46.4	46.3	0.00
Hypertensive disorder	53.9	55.5	-0.03	54.6	54.8	0.00
Lesion of liver	1.1	1.3	-0.02	1.1	1.3	-0.02
Osteoarthritis	52.5	56.2	-0.07	54.5	54.5	0.00
Pneumonia	10.7	7.2	0.12	9.0	9.0	0.00
Psoriasis	3.3	3.0	0.01	3.2	3.1	0.01
Renal impairment	11.1	12.4	-0.04	11.5	11.7	-0.01
Rheumatoid arthritis	84.5	83.3	0.03	84.0	84.0	0.00
Schizophrenia	0.2	0.2	-0.01	0.2	0.2	0.00
Ulcerative colitis	0.7	0.8	-0.01	0.8	0.8	0.00
Urinary tract infectious disease	17.6	19.3	-0.04	18.6	18.5	0.00
Viral hepatitis C	1.1	1.4	-0.03	1.2	1.3	-0.01
Medical history: Cardiovascular disease						
Atrial fibrillation	4.7	6.0	-0.06	5.2	5.5	-0.01
Cerebrovascular disease	6.5	7.0	-0.02	6.6	6.8	-0.01
Coronary arteriosclerosis	11.4	12.1	-0.02	11.6	11.8	0.00
Heart disease	29.8	31.2	-0.03	30.4	30.6	0.00
Heart failure	7.5	7.4	0.00	7.6	7.4	0.01
Ischemic heart disease	7.0	7.3	-0.01	7.1	7.3	0.00
Peripheral vascular disease	4.9	5.2	-0.02	5.1	5.0	0.01
Pulmonary embolism	1.3	1.4	-0.01	1.3	1.4	0.00
Venous thrombosis	2.5	2.9	-0.02	2.6	2.8	-0.01
Medical history: Neoplasms						
Hematologic neoplasm	1.6	1.5	0.01	1.7	1.5	0.01
Malignant lymphoma	0.9	0.9	0.00	0.9	0.9	0.01
Malignant neoplasm of anorectum	0.1	0.2	-0.02	0.1	0.2	-0.01
Malignant neoplastic disease	11.3	11.9	-0.02	11.4	11.6	-0.01
Malignant tumor of breast	2.7	2.7	0.00	2.6	2.7	0.00
Malignant tumor of colon	0.4	0.4	-0.01	0.3	0.4	-0.01
Malignant tumor of lung	0.6	0.4	0.02	0.5	0.5	-0.01
Malignant tumor of urinary bladder	0.3	0.4	-0.03	0.3	0.4	-0.01

Characteristic	Before stratification			After stratification		
	AZM	AMX		AZM	AMX	
	%	%	Std. diff	%	%	Std. diff
Primary malignant neoplasm of prostate	0.8	0.9	-0.02	0.8	0.8	0.00
Medication use						
Antidepressants	42.5	42.6	0.00	43.0	42.8	0.00
Antiepileptics	27.7	28.2	-0.01	28.4	27.9	0.01
Antiinflammatory and antirheumatic products	44.2	45.7	-0.03	45.5	45.2	0.01
Antineoplastic agents	35.2	33.1	0.04	33.4	34.2	-0.02
Antipsoriatics	1.1	1.0	0.01	1.1	1.1	0.01
Antithrombotic agents	13.1	15.4	-0.07	14.0	14.2	0.00
Beta blocking agents	25.8	27.1	-0.03	26.5	26.4	0.00
Drugs for acid related disorders	41.8	41.9	0.00	41.9	41.8	0.00
Drugs for obstructive airway diseases	45.4	36.4	0.18	41.2	41.1	0.00
Immunosuppressants	49.8	47.4	0.05	48.3	48.4	0.00
Lipid modifying agents	32.4	33.2	-0.02	32.6	32.6	0.00
Opioids	41.0	43.3	-0.05	42.7	42.0	0.01
Psycholeptics	35.8	36.3	-0.01	36.5	36.6	0.00
Psychostimulants, agents used for adhd and nootropics	5.2	4.8	0.02	5.3	5.0	0.02

S6.2.4. CPRD

Characteristic	Before stratification			After stratification		
	AZM	AMX		AZM	AMX	
	%	%	Std. diff	%	%	Std. diff
Age group						
20-24	<4.3	0.3	0.07	2.8	0.3	0.20
25-29	<4.3	1.0	-0.01	2.8	1.0	0.13
35-39	<4.3	3.4	0.00	9.4	3.4	0.24
40-44	<4.3	4.8	-0.07	8.9	4.8	0.16
45-49	7.0	7.2	-0.01	4.4	7.2	-0.12
50-54	5.2	10.1	-0.18	8.1	10.0	-0.07
55-59	13.9	12.9	0.03	9.4	12.9	-0.11
60-64	20.9	14.2	0.18	13.3	14.2	-0.03
65-69	13.0	14.3	-0.04	9.2	14.3	-0.16
70-74	14.8	12.2	0.08	13.3	12.2	0.03
75-79	8.7	9.6	-0.03	5.3	9.6	-0.17
80-84	6.1	5.6	0.02	10.3	5.6	0.17
85-89	<4.3	1.9	-0.09	<1.9	1.9	-0.16
90-94	<4.3	0.6	0.03	2.5	0.6	0.15
Gender: female	78.3	73.4	0.11	73.9	73.4	0.01
Medical history: General						
Acute respiratory disease	30.4	20.9	0.22	29.2	21.1	0.19
Chronic obstructive lung disease	13.0	3.7	0.34	6.7	3.8	0.13
Depressive disorder	<4.3	2.5	0.06	<1.9	2.5	-0.10
Diabetes mellitus	4.3	2.3	0.11	3.6	2.3	0.08
Gastroesophageal reflux disease	<4.3	0.7	0.02	<1.9	0.7	-0.06
Gastrointestinal hemorrhage	<4.3	1.6	-0.07	<1.9	1.6	-0.14
Hyperlipidemia	<4.3	0.9	-0.01	<1.9	0.9	-0.08
Hypertensive disorder	<4.3	2.7	-0.01	<1.9	2.7	-0.14
Lesion of liver	<4.3	0.1	0.11	<1.9	<0.1	0.04
Obesity	<4.3	0.3	0.07	<1.9	0.3	-0.01
Osteoarthritis	6.1	4.4	0.07	8.1	4.5	0.15
Pneumonia	<4.3	1.4	0.09	<1.9	1.4	-0.05
Renal impairment	<4.3	3.5	-0.18	2.5	3.5	-0.06
Rheumatoid arthritis	45.2	36.8	0.17	36.4	36.9	-0.01
Urinary tract infectious disease	<4.3	5.2	-0.25	<1.9	5.2	-0.30
Visual system disorder	7.0	8.3	-0.05	9.4	8.3	0.04
Medical history: Cardiovascular disease						
Atrial fibrillation	<4.3	1.1	0.12	<1.9	1.1	-0.03
Heart disease	<4.3	3.8	-0.02	<1.9	3.8	-0.18
Medical history: Neoplasms						
Hematologic neoplasm	<4.3	<0.1	0.12	<1.9	<0.1	0.05
Malignant lymphoma	<4.3	<0.1	0.12	<1.9	<0.1	0.05
Malignant neoplastic disease	<4.3	1.8	0.11	3.3	1.8	0.10
Malignant tumor of breast	<4.3	0.1	0.10	2.5	0.1	0.20
Medication use						
Agents acting on the renin-angiotensin system	28.7	26.5	0.05	22.2	26.5	-0.10
Antibacterials for systemic use	73.9	44.5	0.63	49.2	45.0	0.08
Antidepressants	38.3	31.8	0.14	29.7	31.9	-0.05
Antiepileptics	15.7	8.7	0.21	5.0	8.8	-0.15
Antiinflammatory and antirheumatic products	53.0	52.1	0.02	50.3	52.2	-0.04
Antineoplastic agents	37.4	52.7	-0.31	45.3	52.5	-0.14
Antipsoriatics	<4.3	0.9	-0.01	2.5	0.9	0.12
Antithrombotic agents	27.0	23.8	0.07	15.8	23.9	-0.20
Beta blocking agents	10.4	16.7	-0.18	7.8	16.7	-0.27
Calcium channel blockers	18.3	18.8	-0.02	11.9	18.8	-0.19
Diuretics	18.3	23.3	-0.12	15.0	23.3	-0.21
Drugs for acid related disorders	74.8	63.9	0.24	64.7	64.1	0.01
Drugs for obstructive airway diseases	69.6	32.3	0.80	29.2	33.0	-0.08
Drugs used in diabetes	10.4	8.8	0.06	10.3	8.8	0.05
Immunosuppressants	47.0	58.1	-0.22	50.6	57.9	-0.15

Characteristic	Before stratification			After stratification		
	AZM	AMX		AZM	AMX	
	%	%	Std. diff	%	%	Std. diff
Lipid modifying agents	32.2	28.3	0.08	16.9	28.4	-0.27
Opioids	46.1	39.2	0.14	45.3	39.3	0.12
Psycholeptics	20.9	19.9	0.02	15.6	19.9	-0.11

S6.2.5. DAGermany

Characteristic	Before stratification			After stratification		
	AZM	AMX		AZM	AMX	
	%	%	Std. diff	%	%	Std. diff
Age group						
20-24	<5.6	<2.5	0.02	<5.6	<2.5	0.02
30-34	<5.6	4.0	-0.10	<5.6	4.0	-0.10
35-39	6.7	4.5	0.10	6.7	4.5	0.10
40-44	12.4	4.0	0.31	12.4	4.0	0.31
45-49	7.9	9.4	-0.06	7.9	9.4	-0.06
50-54	13.5	9.4	0.13	13.5	9.4	0.13
55-59	16.9	13.4	0.10	16.9	13.4	0.10
60-64	12.4	10.9	0.05	12.4	10.9	0.05
65-69	10.1	12.4	-0.07	10.1	12.4	-0.07
70-74	7.9	9.4	-0.06	7.9	9.4	-0.06
75-79	5.6	6.9	-0.05	5.6	6.9	-0.05
80-84	<5.6	6.9	-0.22	<5.6	6.9	-0.22
Gender: female	89.9	79.2	0.30	89.9	79.2	0.30
Medical history: General						
Acute respiratory disease	61.8	55.4	0.13	61.8	55.4	0.13
Chronic liver disease	<5.6	<2.5	-0.07	<5.6	<2.5	-0.07
Chronic obstructive lung disease	6.7	11.4	-0.16	6.7	11.4	-0.16
Depressive disorder	14.6	10.4	0.13	14.6	10.4	0.13
Diabetes mellitus	5.6	13.4	-0.27	5.6	13.4	-0.27
Hyperlipidemia	9.0	13.4	-0.14	9.0	13.4	-0.14
Hypertensive disorder	28.1	40.1	-0.26	28.1	40.1	-0.25
Obesity	<5.6	5.0	-0.02	<5.6	5.0	-0.02
Osteoarthritis	11.2	13.4	-0.06	11.2	13.4	-0.06
Pneumonia	<5.6	6.9	-0.22	<5.6	6.9	-0.22
Psoriasis	<5.6	<2.5	0.15	<5.6	<2.5	0.15
Renal impairment	<5.6	4.5	-0.06	<5.6	4.5	-0.06
Rheumatoid arthritis	56.2	57.9	-0.04	56.2	57.9	-0.04
Urinary tract infectious disease	5.6	9.9	-0.16	5.6	9.9	-0.16
Visual system disorder	11.2	16.8	-0.16	11.2	16.8	-0.16
Medical history: Cardiovascular disease						
Cerebrovascular disease	<5.6	<2.5	0.14	<5.6	<2.5	0.14
Coronary arteriosclerosis	<5.6	<2.5	0.06	<5.6	<2.5	0.06
Heart disease	11.2	21.8	-0.29	11.2	21.8	-0.29
Heart failure	<5.6	6.9	-0.30	<5.6	6.9	-0.30
Ischemic heart disease	<5.6	7.9	-0.14	<5.6	7.9	-0.14
Peripheral vascular disease	<5.6	2.5	-0.10	<5.6	2.5	-0.10
Venous thrombosis	<5.6	5.9	-0.12	<5.6	5.9	-0.12
Medical history: Neoplasms						
Malignant neoplastic disease	<5.6	6.4	-0.08	<5.6	6.4	-0.08
Medication use						
Agents acting on the renin-angiotensin system	29.2	39.6	-0.22	29.2	39.6	-0.22
Antibacterials for systemic use	29.2	37.6	-0.18	29.2	37.6	-0.18
Antidepressants	16.9	18.8	-0.05	16.9	18.8	-0.05
Antiepileptics	<5.6	7.9	-0.14	<5.6	7.9	-0.14
Antiinflammatory and antirheumatic products	43.8	46.5	-0.06	43.8	46.5	-0.05
Antineoplastic agents	29.2	27.2	0.04	29.2	27.2	0.04
Antithrombotic agents	13.5	26.2	-0.32	13.5	26.2	-0.32
Beta blocking agents	25.8	35.6	-0.21	25.8	35.6	-0.21
Calcium channel blockers	16.9	18.3	-0.04	16.9	18.3	-0.04
Diuretics	19.1	39.1	-0.45	19.1	39.1	-0.45
Drugs for acid related disorders	65.2	69.8	-0.10	65.2	69.8	-0.10
Drugs for obstructive airway diseases	19.1	21.8	-0.07	19.1	21.8	-0.07
Drugs used in diabetes	6.7	12.9	-0.21	6.7	12.9	-0.21
Immunosuppressants	37.1	32.7	0.09	37.1	32.7	0.09
Lipid modifying agents	5.6	17.8	-0.39	5.6	17.8	-0.38
Opioids	20.2	27.2	-0.16	20.2	27.2	-0.16

Characteristic	Before stratification			After stratification		
	AZM	AMX		AZM	AMX	
	%	%	Std. diff	%	%	Std. diff
Psycholeptics	16.9	10.4	0.19	16.9	10.4	0.19

S6.2.6. IMRD

Characteristic	Before stratification			After stratification		
	AZM	AMX		AZM	AMX	
	%	%	Std. diff	%	%	Std. diff
Age group						
25-29	<4.5	0.9	0.13	3.6	0.9	0.18
30-34	<4.5	1.7	-0.07	6.7	1.7	0.25
35-39	4.5	3.1	0.07	10.0	3.1	0.28
40-44	<4.5	4.3	-0.09	3.3	4.3	-0.05
45-49	5.4	7.3	-0.08	4.0	7.2	-0.14
50-54	<4.5	10.1	-0.26	3.8	10.1	-0.25
55-59	9.8	12.4	-0.08	2.6	12.4	-0.38
60-64	17.9	14.6	0.09	16.6	14.5	0.06
65-69	13.4	14.6	-0.04	19.7	14.7	0.13
70-74	19.6	12.7	0.19	14.2	12.7	0.04
75-79	9.8	9.5	0.01	6.9	9.5	-0.10
80-84	8.0	5.7	0.09	8.3	5.7	0.10
85-89	<4.5	2.0	-0.10	<1.6	2.1	-0.17
90-94	<4.5	0.5	0.04	<1.6	0.5	-0.05
Gender: female	78.6	73.8	0.11	74.1	73.8	0.01
Race						
race = White	30.4	30.8	-0.01	48.2	30.7	0.36
race = Asian Indian	<4.5	0.6	0.03	2.9	0.6	0.17
race = Pakistani	<4.5	0.4	0.07	1.7	0.4	0.13
Medical history: General						
Acute respiratory disease	29.5	20.8	0.20	20.6	20.9	-0.01
Chronic obstructive lung disease	17.0	3.7	0.45	5.9	3.9	0.09
Depressive disorder	4.5	2.5	0.11	2.6	2.5	0.00
Diabetes mellitus	8.0	2.1	0.27	8.3	2.1	0.28
Gastroesophageal reflux disease	<4.5	0.6	0.03	<1.6	0.6	-0.06
Gastrointestinal hemorrhage	<4.5	1.4	-0.05	<1.6	1.4	-0.13
Hyperlipidemia	<4.5	1.0	-0.01	<1.6	1.0	-0.10
Hypertensive disorder	<4.5	2.6	0.00	<1.6	2.6	-0.15
Obesity	<4.5	0.4	0.14	3.1	0.4	0.21
Osteoarthritis	<4.5	4.6	-0.05	7.4	4.6	0.12
Pneumonia	<4.5	1.1	0.17	<1.6	1.1	-0.02
Renal impairment	<4.5	3.1	-0.03	<1.6	3.2	-0.18
Rheumatoid arthritis	33.9	37.3	-0.07	42.0	37.4	0.09
Urinary tract infectious disease	<4.5	4.5	-0.16	5.2	4.6	0.03
Visual system disorder	9.8	8.3	0.05	10.2	8.3	0.06
Medical history: Cardiovascular disease						
Atrial fibrillation	<4.5	1.1	-0.02	<1.6	1.1	-0.11
Cerebrovascular disease	<4.5	0.6	0.11	<1.6	0.6	-0.02
Heart disease	<4.5	3.8	-0.12	<1.6	3.8	-0.23
Venous thrombosis	<4.5	0.8	0.01	6.7	0.8	0.31
Medical history: Neoplasms						
Malignant lymphoma	<4.5	0.1	0.11	<1.6	0.1	0.03
Malignant neoplastic disease	<4.5	1.9	0.06	5.5	1.9	0.19
Malignant tumor of breast	<4.5	0.2	0.09	5.0	0.2	0.30
Medication use						
Antibacterials for systemic use	9.8	4.6	0.20	2.6	4.6	-0.11
Antidepressants	<4.5	1.1	0.16	<1.6	1.1	-0.02
Antiinflammatory and antirheumatic products	<4.5	1.2	-0.03	<1.6	1.2	-0.11
Antineoplastic agents	<4.5	1.6	-0.06	<1.6	1.6	-0.14
Antithrombotic agents	<4.5	1.4	0.10	3.3	1.4	0.13
Beta blocking agents	<4.5	0.5	0.05	<1.6	0.5	-0.04
Diuretics	<4.5	1.0	-0.01	<1.6	1.0	-0.10
Drugs for acid related disorders	<4.5	1.8	-0.08	<1.6	1.8	-0.16
Drugs for obstructive airway diseases	<4.5	0.7	0.02	<1.6	0.7	-0.07
Immunosuppressants	<4.5	1.8	-0.08	<1.6	1.8	-0.16
Lipid modifying agents	<4.5	1.3	0.10	3.3	1.3	0.13

S6.2.7. IPCI

Characteristic	Before stratification			After stratification		
	AZM	AMX		AZM	AMX	
	%	%	Std. diff	%	%	Std. diff
Age group						
20-24	<4.1	<1.0	0.05	<4.1	<1.0	0.05
35-39	<4.1	<1.0	0.19	<4.1	<1.0	0.19
40-44	6.6	4.6	0.09	6.6	4.6	0.09
45-49	9.1	6.6	0.09	9.1	6.6	0.09
50-54	7.4	6.8	0.02	7.4	6.8	0.02
55-59	8.3	11.0	-0.09	8.3	11.0	-0.09
60-64	13.2	14.9	-0.05	13.2	14.9	-0.05
65-69	16.5	10.6	0.18	16.5	10.6	0.18
70-74	12.4	13.5	-0.03	12.4	13.5	-0.03
75-79	9.9	11.8	-0.06	9.9	11.8	-0.06
80-84	8.3	8.9	-0.02	8.3	8.9	-0.02
85-89	<4.1	6.8	-0.16	<4.1	6.8	-0.16
Gender: female	65.3	72.0	-0.15	65.3	72.0	-0.15
Medical history: General						
Acute respiratory disease	37.2	31.9	0.11	37.2	31.9	0.11
Depressive disorder	<4.1	3.7	-0.02	<4.1	3.7	-0.02
Diabetes mellitus	6.6	8.3	-0.06	6.6	8.3	-0.06
Gastrointestinal hemorrhage	<4.1	1.7	0.06	<4.1	1.7	0.06
Hyperlipidemia	<4.1	2.7	-0.14	<4.1	2.7	-0.14
Hypertensive disorder	19.0	17.6	0.04	19.0	17.6	0.04
Obesity	<4.1	1.4	0.12	<4.1	1.4	0.12
Osteoarthritis	4.1	7.0	-0.13	4.1	7.0	-0.13
Pneumonia	14.9	14.9	0.00	14.9	14.9	0.00
Renal impairment	<4.1	3.5	-0.06	<4.1	3.5	-0.06
Ulcerative colitis	<4.1	<1.0	0.15	<4.1	<1.0	0.15
Urinary tract infectious disease	9.9	18.8	-0.26	9.9	18.8	-0.26
Visual system disorder	21.5	15.9	0.14	21.5	15.9	0.14
Medical history: Cardiovascular disease						
Atrial fibrillation	<4.1	4.1	-0.04	<4.1	4.1	-0.04
Cerebrovascular disease	<4.1	2.7	-0.01	<4.1	2.7	-0.01
Heart disease	12.4	14.5	-0.06	12.4	14.5	-0.06
Heart failure	4.1	3.5	0.03	4.1	3.5	0.03
Ischemic heart disease	4.1	6.0	-0.08	4.1	6.0	-0.08
Peripheral vascular disease	<4.1	5.0	-0.25	<4.1	5.0	-0.25
Venous thrombosis	<4.1	1.0	-0.02	<4.1	<1.0	-0.02
Medical history: Neoplasms						
Malignant neoplastic disease	7.4	7.7	-0.01	7.4	7.7	-0.01
Medication use						
Agents acting on the renin-angiotensin system	34.7	35.4	-0.02	34.7	35.4	-0.01
Antibacterials for systemic use	48.8	47.0	0.04	48.8	47.0	0.04
Antidepressants	18.2	19.9	-0.04	18.2	19.9	-0.04
Antiepileptics	9.1	8.9	0.01	9.1	8.9	0.01
Antiinflammatory and antirheumatic products	36.4	41.8	-0.11	36.4	41.8	-0.11
Antineoplastic agents	46.3	48.4	-0.04	46.3	48.4	-0.04
Antipsoriatics	<4.1	1.9	-0.09	<4.1	1.9	-0.09
Antithrombotic agents	30.6	32.5	-0.04	30.6	32.5	-0.04
Beta blocking agents	27.3	31.9	-0.10	27.3	31.9	-0.10
Calcium channel blockers	19.8	20.3	-0.01	19.8	20.3	-0.01
Diuretics	32.2	32.9	-0.02	32.2	32.9	-0.02
Drugs for acid related disorders	77.7	80.3	-0.06	77.7	80.3	-0.06
Drugs for obstructive airway diseases	54.5	42.0	0.25	54.5	42.0	0.25
Drugs used in diabetes	13.2	12.0	0.04	13.2	12.0	0.04
Immunosuppressants	53.7	52.4	0.03	53.7	52.4	0.03
Lipid modifying agents	34.7	38.1	-0.07	34.7	38.1	-0.07
Opioids	24.8	28.8	-0.09	24.8	28.8	-0.09
Psycholeptics	33.9	27.3	0.14	33.9	27.3	0.14

Characteristic	Before stratification			After stratification		
	AZM	AMX		AZM	AMX	
	%	%	Std. diff	%	%	Std. diff
Psychostimulants, agents used for adhd and nootropics	<4.1	<1.0	0.15	<4.1	<1.0	0.15

S6.2.8. MDCD

Characteristic	Before stratification			After stratification		
	AZM	AMX		AZM	AMX	
	%	%	Std. diff	%	%	Std. diff
Age group						
15-19	1.3	1.5	-0.02	1.2	1.5	-0.02
20-24	2.4	2.3	0.01	2.7	2.3	0.03
25-29	4.5	4.9	-0.02	5.1	4.5	0.03
30-34	6.1	7.4	-0.05	6.9	7.0	-0.01
35-39	9.1	10.2	-0.04	9.0	9.5	-0.02
40-44	10.5	11.1	-0.02	10.8	10.7	0.00
45-49	13.1	13.6	-0.02	12.8	13.7	-0.03
50-54	16.5	15.3	0.03	16.3	16.0	0.01
55-59	15.6	15.8	-0.01	15.5	16.2	-0.02
60-64	12.3	10.8	0.05	11.8	11.0	0.02
65-69	4.3	3.2	0.06	4.1	3.5	0.03
70-74	2.2	1.7	0.04	2.0	1.8	0.02
85-89	0.3	0.4	0.00	0.3	0.4	-0.02
Gender: female	89.1	89.7	-0.02	89.3	89.9	-0.02
Race						
race = Black or African American	27.4	29.9	-0.06	27.5	28.0	-0.01
race = White	57.3	54.2	0.06	56.9	56.3	0.01
Medical history: General						
Acute respiratory disease	69.7	54.0	0.33	61.4	63.4	-0.04
Attention deficit hyperactivity disorder	2.6	2.6	0.00	2.7	2.7	0.00
Chronic liver disease	6.5	6.8	-0.01	6.8	6.8	0.00
Chronic obstructive lung disease	28.0	20.4	0.18	22.3	25.6	-0.08
Crohn's disease	1.1	0.7	0.04	1.1	0.7	0.04
Dementia	1.2	1.5	-0.03	1.2	1.6	-0.03
Depressive disorder	39.9	41.4	-0.03	40.9	41.1	0.00
Diabetes mellitus	26.6	25.6	0.02	26.3	26.1	0.00
Gastroesophageal reflux disease	35.4	33.9	0.03	34.5	34.6	0.00
Gastrointestinal hemorrhage	5.0	5.8	-0.04	5.1	5.8	-0.03
Human immunodeficiency virus infection	0.3	0.7	-0.06	0.5	0.6	-0.02
Hyperlipidemia	38.8	35.6	0.07	37.9	37.0	0.02
Hypertensive disorder	59.2	58.0	0.03	59.0	59.4	-0.01
Lesion of liver	1.3	1.7	-0.03	1.5	1.9	-0.03
Obesity	23.3	24.8	-0.04	23.6	24.0	-0.01
Osteoarthritis	55.2	55.1	0.00	54.6	55.7	-0.02
Pneumonia	15.9	9.1	0.21	12.1	12.4	-0.01
Psoriasis	2.1	1.9	0.01	2.2	2.1	0.00
Renal impairment	11.6	12.0	-0.01	10.8	12.0	-0.04
Rheumatoid arthritis	81.8	83.1	-0.03	82.4	83.2	-0.02
Schizophrenia	1.6	1.7	-0.01	1.7	1.7	0.00
Ulcerative colitis	0.5	0.6	-0.01	0.7	0.6	0.01
Urinary tract infectious disease	20.6	22.1	-0.04	21.3	21.2	0.00
Viral hepatitis C	4.8	5.5	-0.03	5.5	5.6	0.00
Visual system disorder	50.0	50.4	-0.01	50.1	51.1	-0.02
Medical history: Cardiovascular disease						
Atrial fibrillation	3.1	2.8	0.02	2.8	3.0	-0.01
Cerebrovascular disease	5.9	5.8	0.00	6.2	6.1	0.00
Coronary arteriosclerosis	11.3	9.0	0.08	9.8	10.0	0.00
Heart disease	35.0	31.0	0.09	32.2	33.2	-0.02
Heart failure	10.9	9.2	0.06	9.4	10.5	-0.04
Ischemic heart disease	9.4	6.9	0.09	8.4	7.5	0.04
Peripheral vascular disease	4.6	5.2	-0.03	4.3	5.2	-0.04
Pulmonary embolism	2.6	2.8	-0.01	2.4	2.9	-0.04
Venous thrombosis	3.5	3.9	-0.02	3.6	4.0	-0.02
Medical history: Neoplasms						
Hematologic neoplasm	1.4	1.5	0.00	1.5	1.5	-0.01
Malignant lymphoma	0.5	0.5	0.01	0.6	0.4	0.03

Characteristic	Before stratification			After stratification		
	AZM	AMX		AZM	AMX	
	%	%	Std. diff	%	%	Std. diff
Malignant neoplasm of anorectum	<0.1	0.2	-0.03	0.1	0.2	-0.02
Malignant neoplastic disease	7.2	7.0	0.01	7.1	6.9	0.01
Malignant tumor of lung	0.5	0.6	-0.01	0.4	0.6	-0.03
Malignant tumor of urinary bladder	<0.1	<0.1	0.02	<0.1	<0.1	0.02
Primary malignant neoplasm of prostate	<0.1	0.2	-0.02	<0.1	0.1	-0.01
Medication use						
Agents acting on the renin-angiotensin system	36.3	34.4	0.04	35.0	35.0	0.00
Antibacterials for systemic use	60.5	59.7	0.02	60.1	59.8	0.00
Antidepressants	62.3	61.3	0.02	62.3	62.4	0.00
Antiepileptics	51.0	49.8	0.02	51.1	50.2	0.02
Antiinflammatory and antirheumatic products	56.8	59.4	-0.05	59.3	58.0	0.03
Antineoplastic agents	36.9	34.2	0.06	36.0	35.6	0.01
Antipsoriatics	0.7	1.0	-0.03	0.6	1.1	-0.05
Antithrombotic agents	22.8	22.6	0.00	22.1	22.9	-0.02
Beta blocking agents	28.6	27.3	0.03	27.7	28.3	-0.01
Calcium channel blockers	23.4	22.0	0.03	22.3	22.7	-0.01
Diuretics	40.4	37.6	0.06	39.1	39.2	0.00
Drugs for acid related disorders	61.7	59.8	0.04	60.3	61.6	-0.03
Drugs for obstructive airway diseases	65.5	53.5	0.25	59.0	60.3	-0.03
Drugs used in diabetes	19.8	18.8	0.02	19.5	18.9	0.02
Immunosuppressants	53.1	49.4	0.07	51.5	51.8	0.00
Lipid modifying agents	32.0	30.2	0.04	31.0	31.3	-0.01
Opioids	66.6	68.3	-0.04	67.6	68.7	-0.02
Psycholeptics	58.0	56.7	0.03	57.4	57.6	0.00
Psychostimulants, agents used for adhd and nootropics	8.3	7.7	0.02	8.2	7.7	0.02

S6.2.9. MDCR

Characteristic	Before stratification			After stratification		
	AZM	AMX		AZM	AMX	
	%	%	Std. diff	%	%	Std. diff
Age group						
45-49	0.1	0.1	0.02	0.1	0.1	0.02
55-59	1.0	1.1	0.00	1.0	1.2	-0.02
60-64	2.1	2.2	-0.01	2.2	2.3	-0.01
65-69	29.2	28.1	0.03	29.0	28.7	0.01
70-74	27.7	28.2	-0.01	27.8	28.0	0.00
75-79	19.6	20.2	-0.01	19.8	20.0	0.00
80-84	12.3	13.0	-0.02	12.4	12.6	-0.01
85-89	5.8	5.3	0.02	5.7	5.3	0.02
90-94	1.4	1.4	0.00	1.4	1.3	0.01
95-99	0.1	0.2	-0.01	0.1	0.2	-0.02
Gender: female	78.1	74.0	0.10	75.7	75.7	0.00
Medical history: General						
Acute respiratory disease	53.3	34.2	0.39	42.5	44.4	-0.04
Attention deficit hyperactivity disorder	0.1	0.2	-0.01	0.1	0.2	-0.01
Chronic liver disease	1.7	2.0	-0.03	1.7	2.1	-0.03
Chronic obstructive lung disease	20.3	14.7	0.15	16.7	18.1	-0.04
Crohn's disease	0.6	0.5	0.02	0.7	0.5	0.03
Dementia	2.1	2.3	-0.02	2.2	2.3	0.00
Depressive disorder	10.4	11.0	-0.02	10.3	11.0	-0.02
Diabetes mellitus	20.7	20.7	0.00	20.6	20.4	0.00
Gastroesophageal reflux disease	19.4	18.8	0.02	19.2	19.7	-0.01
Gastrointestinal hemorrhage	4.4	5.1	-0.03	4.6	5.0	-0.02
Human immunodeficiency virus infection	<0.1	0.1	-0.02	<0.1	0.1	-0.02
Hyperlipidemia	39.1	40.0	-0.02	39.8	39.7	0.00
Hypertensive disorder	62.2	61.6	0.01	61.9	61.4	0.01
Lesion of liver	0.9	1.1	-0.02	0.9	1.1	-0.02
Obesity	5.5	6.3	-0.04	5.8	6.2	-0.02
Osteoarthritis	53.3	57.7	-0.09	56.1	55.5	0.01
Pneumonia	14.3	9.2	0.16	11.6	12.0	-0.01
Psoriasis	2.0	2.4	-0.03	2.2	2.4	-0.02
Renal impairment	12.6	12.5	0.00	12.7	12.5	0.01
Rheumatoid arthritis	87.0	86.5	0.02	86.7	86.6	0.00
Schizophrenia	<0.1	0.1	-0.01	<0.1	0.1	-0.01
Ulcerative colitis	0.7	0.7	-0.01	0.6	0.7	-0.01
Urinary tract infectious disease	15.2	17.1	-0.05	15.6	16.1	-0.01
Viral hepatitis C	0.4	0.5	-0.02	0.4	0.5	-0.01
Visual system disorder	61.5	61.0	0.01	61.3	60.8	0.01
Medical history: Cardiovascular disease						
Atrial fibrillation	9.6	11.3	-0.06	10.3	10.6	-0.01
Cerebrovascular disease	12.0	11.3	0.02	11.8	11.3	0.02
Coronary arteriosclerosis	19.8	20.3	-0.01	20.7	20.5	0.01
Heart disease	44.9	46.4	-0.03	45.7	46.0	-0.01
Heart failure	11.9	10.5	0.04	11.4	11.2	0.01
Ischemic heart disease	10.5	10.0	0.02	10.7	10.2	0.02
Peripheral vascular disease	6.3	6.4	0.00	6.5	6.0	0.02
Pulmonary embolism	1.4	1.4	0.01	1.5	1.3	0.02
Venous thrombosis	3.2	3.8	-0.04	3.5	3.7	-0.02
Medical history: Neoplasms						
Hematologic neoplasm	2.0	2.3	-0.02	2.1	2.2	-0.01
Malignant lymphoma	1.5	1.4	0.01	1.6	1.4	0.02
Malignant neoplasm of anorectum	0.3	0.4	-0.02	0.3	0.4	-0.02
Malignant neoplastic disease	18.8	19.7	-0.02	19.4	19.1	0.01
Malignant tumor of breast	4.1	3.9	0.01	4.3	3.9	0.02
Malignant tumor of colon	0.6	0.8	-0.02	0.7	0.8	-0.01
Malignant tumor of lung	0.8	0.9	-0.01	0.8	1.0	-0.03
Malignant tumor of urinary bladder	0.9	0.9	0.00	0.9	0.9	0.00

Characteristic	Before stratification			After stratification		
	AZM	AMX		AZM	AMX	
	%	%	Std. diff	%	%	Std. diff
Primary malignant neoplasm of prostate	1.9	2.4	-0.03	2.2	2.1	0.01
Medication use						
Agents acting on the renin-angiotensin system	48.0	47.7	0.00	47.7	48.3	-0.01
Antibacterials for systemic use	53.1	51.8	0.03	52.4	52.7	-0.01
Antidepressants	36.6	35.7	0.02	36.2	37.1	-0.02
Antiepileptics	23.7	23.4	0.01	23.9	23.7	0.01
Antiinflammatory and antirheumatic products	37.0	41.3	-0.09	40.3	39.5	0.02
Antineoplastic agents	37.1	36.1	0.02	36.5	37.2	-0.02
Antipsoriatics	1.3	1.2	0.01	1.3	1.3	0.00
Antithrombotic agents	23.3	26.2	-0.07	24.7	24.6	0.00
Beta blocking agents	40.3	41.7	-0.03	41.4	41.0	0.01
Calcium channel blockers	30.5	29.5	0.02	30.2	29.9	0.01
Diuretics	48.1	47.5	0.01	47.6	48.4	-0.02
Drugs for acid related disorders	52.0	49.7	0.05	51.2	51.6	-0.01
Drugs for obstructive airway diseases	49.3	36.0	0.27	41.8	43.0	-0.02
Drugs used in diabetes	15.5	15.1	0.01	15.5	15.1	0.01
Immunosuppressants	49.6	47.6	0.04	48.5	49.3	-0.02
Lipid modifying agents	47.4	47.6	0.00	48.0	47.9	0.00
Opioids	41.9	44.9	-0.06	42.9	43.2	0.00
Psycholeptics	38.7	37.1	0.03	37.9	38.2	-0.01
Psychostimulants, agents used for adhd and nootropics	2.3	2.6	-0.02	2.4	2.7	-0.02

S6.2.10. OpenClaims

Characteristic	Before stratification			After stratification		
	AZM	AMX		AZM	AMX	
	%	%	Std. diff	%	%	Std. diff
Age group						
15-19	0.3	0.3	0.00	0.3	0.3	0.00
20-24	1.0	1.0	0.00	1.0	1.0	0.00
25-29	1.8	1.7	0.00	1.8	1.8	0.00
30-34	3.0	2.9	0.01	3.0	2.9	0.00
40-44	6.1	5.7	0.02	6.0	5.9	0.00
50-54	11.7	11.1	0.02	11.5	11.5	0.00
55-59	14.0	13.9	0.00	14.0	14.0	0.00
60-64	13.8	14.1	-0.01	13.9	14.1	0.00
65-69	12.4	13.0	-0.02	12.7	12.7	0.00
80-84	4.0	4.6	-0.03	4.3	4.2	0.00
85-89	0.2	0.2	0.00	0.2	0.2	0.01
Gender: female	84.7	82.1	0.07	83.5	83.5	0.00
Medical history: General						
Acute respiratory disease	36.6	27.3	0.20	33.1	31.5	0.03
Attention deficit hyperactivity disorder	0.9	0.8	0.00	0.9	0.8	0.00
Chronic liver disease	2.1	2.2	-0.01	2.2	2.2	0.00
Chronic obstructive lung disease	10.6	8.0	0.09	9.4	9.3	0.00
Crohn's disease	0.5	0.5	0.00	0.5	0.5	0.00
Dementia	0.9	1.0	-0.01	0.9	0.9	0.00
Depressive disorder	11.3	11.6	-0.01	11.7	11.5	0.01
Diabetes mellitus	16.5	16.9	-0.01	16.8	16.7	0.00
Gastroesophageal reflux disease	5.5	6.4	-0.04	6.0	5.9	0.00
Gastrointestinal hemorrhage	2.5	2.7	-0.01	2.6	2.6	0.00
Hyperlipidemia	26.6	27.3	-0.02	27.1	26.8	0.01
Hypertensive disorder	37.0	38.1	-0.02	37.9	37.4	0.01
Lesion of liver	0.8	0.9	-0.01	0.9	0.9	0.00
Obesity	7.6	7.8	-0.01	7.8	7.7	0.00
Osteoarthritis	35.5	38.0	-0.05	37.0	36.6	0.01
Pneumonia	7.1	4.8	0.10	6.1	5.8	0.01
Psoriasis	1.9	1.9	0.00	2.0	1.9	0.00
Renal impairment	7.2	7.7	-0.02	7.5	7.5	0.00
Rheumatoid arthritis	68.4	67.8	0.01	68.1	68.0	0.00
Schizophrenia	0.2	0.2	0.01	0.2	0.2	0.01
Ulcerative colitis	0.5	0.5	0.00	0.5	0.5	0.00
Urinary tract infectious disease	10.0	11.0	-0.03	10.6	10.5	0.00
Viral hepatitis C	1.0	1.1	-0.01	1.0	1.0	0.00
Visual system disorder	26.7	27.3	-0.01	27.1	27.0	0.00
Medical history: Cardiovascular disease						
Atrial fibrillation	3.5	4.4	-0.05	4.0	4.0	0.00
Cerebrovascular disease	3.5	3.5	0.00	3.6	3.5	0.00
Coronary arteriosclerosis	7.3	7.7	-0.02	7.7	7.6	0.00
Heart disease	19.8	20.8	-0.02	20.4	20.3	0.00
Ischemic heart disease	3.9	3.9	0.00	4.0	3.9	0.00
Peripheral vascular disease	10.4	11.2	-0.03	11.0	10.8	0.00
Pulmonary embolism	0.9	1.0	-0.01	1.0	1.0	0.00
Venous thrombosis	1.6	1.8	-0.01	1.7	1.7	0.00
Medical history: Neoplasms						
Hematologic neoplasm	1.0	1.1	-0.01	1.1	1.1	0.00
Malignant lymphoma	0.7	0.7	0.00	0.7	0.7	0.00
Malignant neoplasm of anorectum	0.1	0.1	0.00	0.1	0.1	0.00
Malignant neoplastic disease	8.0	8.4	-0.01	8.3	8.2	0.00
Malignant tumor of breast	2.1	2.1	0.00	2.2	2.1	0.00
Malignant tumor of colon	0.3	0.3	-0.01	0.3	0.3	0.00
Malignant tumor of lung	0.4	0.3	0.01	0.4	0.4	0.00
Malignant tumor of urinary bladder	0.2	0.3	-0.01	0.2	0.3	0.00
Primary malignant neoplasm of prostate	0.6	0.7	-0.01	0.6	0.6	0.00

Characteristic	Before stratification			After stratification		
	AZM	AMX		AZM	AMX	
	%	%	Std. diff	%	%	Std. diff
Medication use						
Agents acting on the renin-angiotensin system	37.3	38.2	-0.02	38.0	37.9	0.00
Antibacterials for systemic use	49.3	49.7	-0.01	49.9	49.9	0.00
Antidepressants	45.6	45.4	0.00	46.0	45.8	0.00
Antiepileptics	32.9	33.3	-0.01	33.5	33.3	0.00
Antiinflammatory and antirheumatic products	45.3	47.7	-0.05	46.8	46.6	0.00
Antineoplastic agents	38.6	35.9	0.06	37.0	37.3	0.00
Antithrombotic agents	15.7	18.3	-0.07	17.2	17.0	0.00
Beta blocking agents	28.8	30.2	-0.03	29.7	29.6	0.00
Calcium channel blockers	21.7	22.0	-0.01	22.0	21.9	0.00
Diuretics	35.6	35.8	0.00	35.9	35.9	0.00
Drugs for acid related disorders	47.6	47.4	0.00	47.7	47.8	0.00
Drugs for obstructive airway diseases	50.2	40.6	0.19	45.8	45.5	0.01
Immunosuppressants	52.8	50.2	0.05	51.4	51.5	0.00
Lipid modifying agents	34.8	35.9	-0.02	35.6	35.5	0.00
Opioids	41.8	44.3	-0.05	43.5	43.2	0.01
Psycholeptics	39.2	38.4	0.02	39.3	39.2	0.00
Psychostimulants, agents used for adhd and nootropics	6.7	6.5	0.01	6.8	6.7	0.00

S6.2.11. OptumEHR

Characteristic	Before stratification			After stratification		
	AZM	AMX		AZM	AMX	
	%	%	Std. diff	%	%	Std. diff
Age group						
15-19	0.2	0.3	-0.02	0.2	0.3	-0.02
20-24	0.9	0.9	0.00	1.1	0.8	0.03
25-29	1.6	1.7	0.00	1.9	1.6	0.02
30-34	2.7	3.1	-0.03	2.9	2.9	0.00
35-39	3.7	4.5	-0.04	4.1	4.1	0.00
40-44	5.3	5.9	-0.03	5.7	6.0	-0.01
45-49	7.1	7.4	-0.01	7.3	7.4	0.00
50-54	10.5	10.5	0.00	10.5	10.3	0.01
55-59	13.1	13.8	-0.02	13.2	13.9	-0.02
60-64	13.7	13.6	0.00	13.9	13.8	0.00
65-69	12.6	12.2	0.01	12.4	12.5	0.00
70-74	10.9	10.5	0.01	10.5	10.6	0.00
75-79	8.6	7.6	0.04	8.2	7.9	0.01
80-84	6.4	6.0	0.02	5.9	5.8	0.00
85-89	2.6	2.1	0.04	2.3	2.0	0.02
Gender: female	82.3	82.1	0.00	82.9	82.7	0.01
Race						
race = Asian	1.1	1.0	0.01	1.0	1.0	0.01
race = Black or African American	10.5	9.4	0.04	10.0	9.4	0.02
race = White	83.4	84.3	-0.02	84.1	84.4	-0.01
Ethnicity						
ethnicity = Hispanic or Latino	4.6	4.4	0.01	4.5	4.5	0.00
Medical history: General						
Acute respiratory disease	57.2	43.2	0.28	52.6	51.8	0.02
Attention deficit hyperactivity disorder	1.0	1.5	-0.04	1.1	1.4	-0.03
Chronic liver disease	3.3	3.4	-0.01	3.4	3.4	0.00
Chronic obstructive lung disease	19.5	11.0	0.24	15.2	15.1	0.00
Crohn's disease	0.6	0.6	0.00	0.7	0.5	0.02
Dementia	2.2	1.9	0.02	1.9	1.9	0.00
Depressive disorder	23.7	23.4	0.01	23.9	23.4	0.01
Diabetes mellitus	19.3	18.3	0.03	18.8	18.4	0.01
Gastroesophageal reflux disease	29.6	28.1	0.03	29.2	28.8	0.01
Human immunodeficiency virus infection	0.2	0.1	0.01	0.2	0.2	0.00
Hyperlipidemia	40.8	38.8	0.04	39.7	39.4	0.01
Hypertensive disorder	51.4	49.7	0.03	50.4	50.4	0.00
Lesion of liver	1.5	1.5	0.00	1.6	1.4	0.01
Obesity	17.5	18.1	-0.02	17.6	17.8	-0.01
Osteoarthritis	41.1	42.2	-0.02	41.4	41.0	0.01
Pneumonia	20.6	7.6	0.38	14.7	12.4	0.07
Psoriasis	2.1	2.1	0.00	2.2	2.1	0.01
Renal impairment	14.7	12.8	0.06	13.2	12.6	0.02
Rheumatoid arthritis	84.8	83.4	0.04	84.0	84.3	-0.01
Schizophrenia	0.3	0.3	-0.01	0.2	0.3	-0.01
Ulcerative colitis	0.5	0.7	-0.03	0.6	0.6	0.00
Urinary tract infectious disease	13.1	14.1	-0.03	13.7	13.2	0.02
Viral hepatitis C	1.3	1.1	0.02	1.3	1.1	0.01
Visual system disorder	21.4	22.6	-0.03	21.5	21.6	0.00
Medical history: Cardiovascular disease						
Atrial fibrillation	7.5	6.9	0.02	6.9	7.1	-0.01
Cerebrovascular disease	5.1	4.5	0.03	4.8	4.5	0.01
Coronary arteriosclerosis	13.4	12.1	0.04	12.5	12.5	0.00
Heart disease	33.3	29.6	0.08	31.1	30.3	0.02
Heart failure	11.3	8.0	0.12	9.4	8.6	0.03
Ischemic heart disease	8.0	6.7	0.05	7.2	7.0	0.01
Pulmonary embolism	1.7	1.8	-0.01	1.7	1.7	0.00
Venous thrombosis	3.3	3.4	-0.01	3.3	3.1	0.01

Characteristic	Before stratification			After stratification		
	AZM	AMX		AZM	AMX	
	%	%	Std. diff	%	%	Std. diff
Medical history: Neoplasms						
Hematologic neoplasm	1.7	1.7	0.00	1.8	1.6	0.01
Malignant lymphoma	0.8	1.0	-0.02	0.8	0.9	-0.02
Malignant neoplasm of anorectum	0.4	0.5	-0.02	0.3	0.4	0.00
Malignant neoplastic disease	11.8	13.3	-0.05	12.2	12.5	-0.01
Malignant tumor of breast	2.5	3.1	-0.03	2.7	2.8	-0.01
Malignant tumor of colon	0.5	0.6	-0.01	0.5	0.5	0.01
Malignant tumor of lung	1.0	0.7	0.04	0.9	0.9	-0.01
Malignant tumor of urinary bladder	0.3	0.4	-0.02	0.3	0.3	-0.01
Primary malignant neoplasm of prostate	0.8	0.8	0.00	0.9	0.7	0.02
Medication use						
Agents acting on the renin-angiotensin system	37.6	36.5	0.02	36.9	36.5	0.01
Antibacterials for systemic use	53.0	51.6	0.03	52.1	50.8	0.03
Antidepressants	47.6	48.1	-0.01	48.1	48.3	0.00
Antiepileptics	33.3	34.7	-0.03	33.8	34.1	0.00
Antiinflammatory and antirheumatic products	47.1	50.6	-0.07	48.8	49.7	-0.02
Antineoplastic agents	39.5	36.8	0.06	38.1	38.6	-0.01
Antithrombotic agents	47.7	44.7	0.06	45.4	44.1	0.03
Beta blocking agents	35.2	35.8	-0.01	35.0	34.9	0.00
Calcium channel blockers	24.0	22.2	0.04	22.9	22.8	0.00
Diuretics	40.3	38.3	0.04	39.3	38.9	0.01
Drugs for acid related disorders	61.8	61.0	0.02	61.0	61.0	0.00
Drugs for obstructive airway diseases	64.7	52.3	0.25	58.6	59.1	-0.01
Drugs used in diabetes	19.7	19.2	0.02	19.1	19.1	0.00
Immunosuppressants	56.0	53.6	0.05	55.1	55.5	-0.01
Lipid modifying agents	37.0	36.1	0.02	36.2	35.9	0.01
Opioids	52.5	53.9	-0.03	52.3	52.2	0.00
Psycholeptics	49.8	49.6	0.00	49.2	49.5	-0.01
Psychostimulants, agents used for adhd and nootropics	5.6	6.6	-0.04	6.0	6.3	-0.01

S6.2.12. SIDIAP

Characteristic	Before stratification			After stratification		
	AZM	AMX		AZM	AMX	
	%	%	Std. diff	%	%	Std. diff
Age group						
20-24	<3.8	<0.7	0.09	<2.8	<0.7	0.07
30-34	<3.8	4.1	-0.10	<2.8	4.0	-0.12
35-39	3.8	5.5	-0.08	6.1	5.4	0.03
40-44	6.2	6.3	0.00	3.3	6.1	-0.13
45-49	4.6	6.0	-0.06	5.7	5.8	-0.01
50-54	13.1	8.3	0.16	17.6	8.3	0.28
55-59	15.4	10.3	0.15	17.5	10.5	0.20
60-64	10.8	12.7	-0.06	12.9	12.9	0.00
65-69	7.7	12.2	-0.15	5.0	12.5	-0.27
70-74	12.3	11.1	0.04	10.0	11.0	-0.03
75-79	7.7	8.9	-0.04	4.5	8.6	-0.17
80-84	7.7	8.2	-0.02	9.0	8.3	0.03
85-89	6.2	3.1	0.15	4.0	3.3	0.03
90-94	<3.8	<0.7	0.09	<2.8	<0.7	0.12
Gender: female	80.0	77.6	0.06	79.9	77.9	0.05
Medical history: General						
Acute respiratory disease	26.9	20.1	0.16	29.4	20.6	0.20
Chronic liver disease	<3.8	1.2	-0.04	<2.8	1.2	-0.09
Chronic obstructive lung disease	4.6	2.6	0.11	3.7	2.7	0.05
Dementia	<3.8	0.9	-0.01	<2.8	0.9	-0.06
Depressive disorder	<3.8	4.7	-0.08	<2.8	4.9	-0.11
Diabetes mellitus	<3.8	3.6	-0.13	<2.8	3.5	-0.10
Gastroesophageal reflux disease	<3.8	1.3	0.02	<2.8	1.3	-0.04
Gastrointestinal hemorrhage	5.4	2.0	0.18	5.5	2.1	0.18
Hyperlipidemia	4.6	6.4	-0.08	3.8	6.3	-0.12
Hypertensive disorder	6.2	7.1	-0.04	6.3	7.0	-0.03
Obesity	7.7	8.0	-0.01	9.8	7.9	0.07
Osteoarthritis	4.6	3.8	0.04	5.2	3.7	0.07
Pneumonia	<3.8	3.4	-0.06	<2.8	3.4	-0.14
Renal impairment	<3.8	2.9	-0.04	<2.8	2.8	-0.03
Rheumatoid arthritis	20.8	29.3	-0.20	22.9	28.8	-0.13
Urinary tract infectious disease	8.5	10.8	-0.08	5.5	10.8	-0.19
Viral hepatitis C	<3.8	<0.7	0.02	<2.8	<0.7	-0.04
Visual system disorder	17.7	20.8	-0.08	14.6	20.9	-0.16
Medical history: Cardiovascular disease						
Cerebrovascular disease	4.6	4.1	0.03	5.5	4.3	0.06
Heart disease	6.9	10.1	-0.11	6.9	10.0	-0.11
Heart failure	<3.8	1.5	-0.07	<2.8	1.5	-0.10
Ischemic heart disease	<3.8	3.4	-0.02	<2.8	3.4	-0.09
Peripheral vascular disease	8.5	6.4	0.08	5.8	6.4	-0.02
Venous thrombosis	<3.8	<0.7	0.04	<2.8	<0.7	-0.01
Medical history: Neoplasms						
Malignant neoplastic disease	<3.8	4.7	-0.13	<2.8	4.8	-0.18
Medication use						
Agents acting on the renin-angiotensin system	26.9	31.2	-0.09	21.0	31.1	-0.23
Antibacterials for systemic use	27.7	27.1	0.01	23.2	27.6	-0.10
Antidepressants	30.8	28.9	0.04	33.8	29.2	0.10
Antiepileptics	16.2	14.0	0.06	14.3	14.2	0.00
Antiinflammatory and antirheumatic products	62.3	64.4	-0.04	55.1	64.6	-0.20
Antineoplastic agents	27.7	17.6	0.24	18.1	19.1	-0.03
Antipsoriatics	<3.8	1.2	-0.04	<2.8	1.1	0.08
Antithrombotic agents	20.8	25.2	-0.11	22.5	25.0	-0.06
Beta blocking agents	15.4	15.0	0.01	15.3	14.9	0.01
Calcium channel blockers	9.2	9.8	-0.02	10.1	9.5	0.02
Diuretics	30.0	29.3	0.02	30.3	29.3	0.02
Drugs for acid related disorders	89.2	83.2	0.18	82.0	83.9	-0.05

Characteristic	Before stratification			After stratification		
	AZM	AMX		AZM	AMX	
	%	%	Std. diff	%	%	Std. diff
Drugs for obstructive airway diseases	48.5	38.9	0.19	34.3	41.2	-0.14
Drugs used in diabetes	11.5	14.7	-0.09	11.8	14.5	-0.08
Immunosuppressants	37.7	29.4	0.18	26.9	30.8	-0.08
Lipid modifying agents	26.9	27.4	-0.01	27.1	27.4	-0.01
Opioids	16.2	22.2	-0.15	13.2	22.5	-0.24
Psycholeptics	43.8	41.3	0.05	41.1	41.6	-0.01
Psychostimulants, agents used for adhd and nootropics	5.4	2.5	0.15	3.6	2.5	0.06

S6.2.13. VA

Characteristic	Before stratification			After stratification		
	AZM	AMX		AZM	AMX	
	%	%	Std. diff	%	%	Std. diff
Age group						
20-24	<0.1	0.1	0.00	<0.1	0.1	0.00
25-29	0.4	0.2	0.03	0.4	0.3	0.03
30-34	1.0	1.0	0.00	1.2	1.1	0.02
35-39	1.3	1.5	-0.02	1.3	1.4	-0.01
40-44	2.5	2.8	-0.02	3.1	2.7	0.02
45-49	4.9	5.5	-0.03	5.9	5.4	0.02
50-54	8.8	9.1	-0.01	9.6	9.0	0.02
55-59	13.4	14.6	-0.03	12.9	14.3	-0.04
60-64	17.6	17.2	0.01	17.1	18.0	-0.02
65-69	18.2	17.5	0.02	17.2	17.0	0.00
70-74	13.7	13.9	-0.01	14.5	14.2	0.01
75-79	8.9	8.6	0.01	8.2	8.6	-0.01
80-84	5.7	5.4	0.02	5.4	5.2	0.00
85-89	2.9	2.0	0.05	2.5	2.0	0.03
90-94	0.6	0.5	0.02	0.5	0.6	-0.01
95-99	<0.1	<0.1	0.01	<0.1	<0.1	0.00
Gender: female	18.6	16.7	0.05	18.7	17.5	0.03
Race						
race = Asian	0.4	0.3	0.01	0.3	0.3	0.01
race = Black or African American	16.5	17.8	-0.04	17.0	17.7	-0.02
race = White	74.0	71.1	0.06	73.2	71.8	0.03
race = Unknown	7.7	8.9	-0.04	8.0	8.4	-0.02
race = Native Hawaiian or Other Pacific Islander	0.6	0.8	-0.02	0.7	0.8	-0.01
race = American Indian or Alaska Native	0.8	1.1	-0.02	0.8	1.0	-0.02
Ethnicity						
ethnicity = Hispanic or Latino	4.7	5.2	-0.02	3.9	4.8	-0.04
ethnicity = Not Hispanic or Latino	91.1	89.3	0.06	91.6	89.9	0.06
Medical history: General						
Acute respiratory disease	48.2	29.9	0.38	40.3	40.4	0.00
Attention deficit hyperactivity disorder	0.4	0.6	-0.04	0.4	0.6	-0.03
Chronic liver disease	3.7	4.6	-0.04	3.7	4.2	-0.03
Chronic obstructive lung disease	33.7	22.5	0.25	26.1	27.6	-0.03
Crohn's disease	0.4	0.4	0.00	0.4	0.3	0.02
Depressive disorder	28.4	27.9	0.01	29.5	28.5	0.02
Diabetes mellitus	27.4	29.5	-0.05	28.0	28.2	0.00
Gastroesophageal reflux disease	7.2	8.0	-0.03	7.4	7.7	-0.01
Gastrointestinal hemorrhage	3.2	3.7	-0.03	3.8	3.2	0.03
Human immunodeficiency virus infection	0.3	0.4	-0.02	0.3	0.4	-0.02
Hyperlipidemia	48.8	46.6	0.04	47.0	47.0	0.00
Hypertensive disorder	64.7	63.3	0.03	63.8	63.3	0.01
Lesion of liver	1.5	1.9	-0.03	1.7	1.9	-0.02
Obesity	18.4	18.4	0.00	17.8	18.5	-0.02
Osteoarthritis	42.8	43.8	-0.02	42.6	42.9	0.00
Pneumonia	18.2	7.9	0.31	12.9	11.4	0.04
Psoriasis	2.4	2.5	0.00	2.5	2.2	0.02
Renal impairment	11.9	11.8	0.00	11.4	11.2	0.01
Rheumatoid arthritis	87.3	86.0	0.04	86.0	86.7	-0.02
Schizophrenia	0.7	1.1	-0.04	1.1	1.1	0.00
Ulcerative colitis	0.4	0.5	-0.02	0.5	0.4	0.01
Urinary tract infectious disease	5.9	8.3	-0.09	7.4	7.2	0.01
Visual system disorder	65.7	65.2	0.01	65.2	64.9	0.01
Medical history: Cardiovascular disease						
Atrial fibrillation	7.4	8.3	-0.03	7.9	8.1	-0.01
Cerebrovascular disease	6.1	5.6	0.02	6.1	5.4	0.03
Coronary arteriosclerosis	19.5	18.3	0.03	18.6	18.0	0.02
Heart disease	39.8	38.2	0.03	38.7	37.7	0.02

Characteristic	Before stratification			After stratification		
	AZM	AMX		AZM	AMX	
	%	%	Std. diff	%	%	Std. diff
Heart failure	11.8	9.9	0.06	11.3	10.2	0.04
Ischemic heart disease	15.7	15.2	0.01	15.2	15.1	0.00
Peripheral vascular disease	17.6	18.4	-0.02	18.1	18.0	0.00
Pulmonary embolism	1.5	1.6	-0.01	1.7	1.5	0.02
Venous thrombosis	2.3	2.5	-0.01	2.4	2.2	0.01
Medical history: Neoplasms						
Hematologic neoplasm	1.8	2.2	-0.03	1.9	2.2	-0.02
Malignant lymphoma	1.2	1.3	-0.01	1.2	1.2	0.00
Malignant neoplasm of anorectum	0.2	0.2	0.00	0.1	0.1	0.00
Malignant neoplastic disease	16.3	16.4	0.00	17.1	16.0	0.03
Malignant tumor of breast	0.8	0.4	0.06	0.8	0.4	0.05
Malignant tumor of colon	0.6	0.8	-0.03	0.5	0.7	-0.03
Malignant tumor of lung	1.1	1.0	0.00	1.0	1.2	-0.02
Malignant tumor of urinary bladder	1.2	1.1	0.00	1.3	1.1	0.02
Primary malignant neoplasm of prostate	4.6	5.0	-0.02	5.0	4.9	0.01
Medication use						
Agents acting on the renin-angiotensin system	45.4	45.3	0.00	44.8	44.9	0.00
Antiepileptics	29.8	31.3	-0.03	32.1	30.4	0.04
Antiinflammatory and antirheumatic products	49.5	51.7	-0.04	51.6	51.5	0.00
Antineoplastic agents	45.5	39.0	0.13	41.5	42.1	-0.01
Antipsoriatics	1.8	1.6	0.02	2.2	1.4	0.06
Antithrombotic agents	45.3	45.3	0.00	45.7	43.9	0.04
Beta blocking agents	40.4	41.4	-0.02	41.0	40.2	0.02
Calcium channel blockers	27.4	27.6	-0.01	28.2	27.0	0.03
Diuretics	42.4	41.4	0.02	42.3	41.3	0.02
Drugs for acid related disorders	71.1	70.4	0.01	71.1	70.3	0.02
Drugs for obstructive airway diseases	57.5	41.7	0.32	48.3	49.0	-0.01
Drugs used in diabetes	23.1	25.1	-0.05	24.4	23.9	0.01
Immunosuppressants	58.8	52.8	0.12	54.7	55.5	-0.02
Lipid modifying agents	51.9	49.9	0.04	50.4	49.7	0.01
Opioids	43.7	46.6	-0.06	45.3	44.1	0.02
Psycholeptics	35.3	36.3	-0.02	36.2	35.7	0.01
Psychostimulants, agents used for adhd and nootropics	2.7	2.9	-0.02	2.9	2.9	0.00

S7. Patient counts, events, incident rates

Patient counts, event counts, and incidence rates per 1,000 person-years are reported below for hydroxychloroquine vs sulfasalazine and hydroxychloroquine+azithromycin vs hydroxychloroquine+amoxicillin during 30-day and on-treatment follow-up periods for outcomes not reported in the manuscript. Note that the IR does not account for stratification.

Comparison	Outcome	Database	30-day follow-up						On-treatment follow-up					
			Patients		Events		IR		Patients		Events		IR	
			T	C	T	C	T	C	T	C	T	C	T	C
HCQ vs SSZ	All-cause mortality	Clinformatics	51,280	17,389	20	10	4.81	7.09	51,280	17,389	527	66	9.88	5.29
		CPRD	9,127	11,398	6	5	8.03	5.35	9,127	11,398	253	386	14.02	14.56
		IMRD	8,851	8,460	<5	6	<6.91	8.66	8,851	8,460	214	241	12.32	12.72
		VA	32,028	14,349	45	17	17.13	14.45	32,028	14,349	1,356	327	24.51	18.65
		Meta-analysis	101,286	51,596	<76	38	<9.20	9.02	NA	NA	NA	NA	NA	NA
	Myocardial infarction	CCAE	66,630	22,374	29	11	5.36	6.05	66,630	22,374	240	48	3.64	3.28
		Clinformatics	51,244	17,380	51	20	12.27	14.19	51,244	17,380	517	121	9.79	9.81
		MDCD	8,079	2,196	13	5	19.75	27.98	8,079	2,196	83	14	14.32	11.39
		MDCR	15,827	5,190	26	10	20.17	23.68	15,827	5,190	287	70	15.93	17.55
		OpenClaims	620,751	183,482	369	89	7.24	5.91	620,751	183,482	5,396	956	6.25	5.57
		OptumEHR	78,424	21,885	59	12	9.29	6.77	NA	NA	NA	NA	NA	NA
		VA	32,009	14,342	30	9	11.43	7.65	32,009	14,342	395	137	7.22	7.89
		Meta-analysis	872,964	266,849	577	156	8.08	7.14	794,540	244,964	6,918	1,346	6.52	6.08
	Cardiovascular events	CCAE	66,533	22,357	97	36	17.96	19.83	66,533	22,357	839	197	12.86	13.6
		Clinformatics	51,095	17,340	215	76	51.96	54.11	51,095	17,340	2,232	490	43.8	40.92
		MDCD	8,041	2,186	35	11	53.5	61.95	8,041	2,186	388	78	70.54	66.08
		MDCR	15,759	5,167	117	39	91.39	93.05	15,759	5,167	1,295	310	76.12	82.67
		OpenClaims	619,493	183,209	1,636	470	32.2	31.28	619,493	183,209	22,657	4,394	26.94	26.16
		OptumEHR	77,825	21,752	294	70	46.7	39.79	NA	NA	NA	NA	NA	NA
		VA	31,913	14,300	120	46	45.92	39.28	31,913	14,300	1,577	546	29.84	32.57
		Meta-analysis	870,659	266,311	2,514	748	35.31	34.35	792,834	244,559	28,988	6,015	28.07	27.83
	Cardiac arrhythmia	AmbEMR	56,743	15,208	147	43	31.61	34.5	56,743	15,208	467	163	25.54	29.19
		CCAE	66,430	22,337	153	58	28.38	32	66,430	22,337	1,301	348	20.3	24.42
		Clinformatics	51,069	17,320	159	61	38.43	43.46	51,069	17,320	1,557	461	30.64	39.13
		CPRD	9,120	11,390	7	14	9.38	15	9,120	11,390	113	180	6.37	6.95
		DAGermany	3,883	5,040	<5	5	<15.70	12.09	3,883	5,040	22	38	8.68	10.82
		IMRD	8,847	8,452	8	12	11.06	17.35	8,847	8,452	101	123	5.9	6.64
		MDCD	8,051	2,191	37	10	56.48	56.15	8,051	2,191	232	40	41.47	33.06
		MDCR	15,763	5,165	73	24	56.93	57.2	15,763	5,165	726	183	42.35	48.33
		OpenClaims	619,285	183,147	1,505	490	29.63	32.62	619,285	183,147	19,445	4,134	23.37	24.86
		OptumEHR	77,008	21,581	472	115	75.89	65.97	NA	NA	NA	NA	NA	NA
		VA	31,766	14,275	107	45	41.12	38.48	31,766	14,275	1,391	508	26.84	30.95
		Meta-analysis	947,965	306,106	<2,673	877	<34.48	35.03	NA	NA	NA	NA	NA	NA

Table S7, continued.

Comparison	Outcome	Database	30-day follow-up						On-treatment follow-up					
			Patients		Events		IR		Patients		Events		IR	
			T	C	T	C	T	C	T	C	T	C	T	C
HCQ vs SSZ	Bradycardia	AmbEMR	57,594	15,350	17	<5	3.6	<3.97	57,594	15,350	60	15	3.2	2.63
		CCAE	66,640	22,373	5	<5	0.92	<2.75	66,640	22,373	69	13	1.04	0.89
		Clinformatics	51,259	17,386	23	<5	5.53	<3.54	51,259	17,386	256	55	4.83	4.43
		CPRD	NA	NA	NA	NA	NA	NA	9,127	11,398	<5	<5	<0.28	<0.19
		IMRD	NA	NA	NA	NA	NA	NA	8,852	8,460	<5	<5	<0.29	<0.26
		MDCD	8,085	2,197	6	<5	9.1	<27.96	8,085	2,197	30	<5	5.14	<4.02
		MDCR	NA	NA	NA	NA	NA	NA	15,830	5,190	106	25	5.84	6.21
		OpenClaims	620,919	183,510	155	56	3.04	3.72	620,919	183,510	2,791	507	3.22	2.95
		OptumEHR	78,425	21,911	62	16	9.76	9.02	NA	NA	NA	NA	NA	NA
		VA	32,018	14,345	9	<5	3.43	<4.25	32,018	14,345	231	62	4.21	3.56
		Meta-analysis	914,940	277,072	277	<97	3.7	<4.28	870,324	280,209	<3,553	<692	<3.18	<2.53
	Transient ischemic attack	CCAE	66,617	22,372	23	6	4.25	3.3	66,617	22,372	187	35	2.84	2.39
		Clinformatics	51,235	17,377	38	9	9.14	6.38	51,235	17,377	375	75	7.09	6.06
		MDCD	8,078	2,198	7	<5	10.63	<27.96	8,078	2,198	58	10	9.97	8.08
		MDCR	15,821	5,187	17	7	13.19	16.59	15,821	5,187	202	47	11.18	11.74
		OpenClaims	620,812	183,511	261	56	5.12	3.72	620,812	183,511	3,381	615	3.91	3.58
		OptumEHR	78,520	21,921	26	7	4.09	3.94	NA	NA	NA	NA	NA	NA
		VA	32,010	14,346	10	5	3.81	4.25	32,010	14,346	153	49	2.78	2.81
		Meta-analysis	873,093	266,912	382	<95	5.34	<4.35	794,573	244,991	4,356	831	4.1	3.75
	Stroke	CCAE	66,619	22,374	26	8	4.8	4.4	66,619	22,374	221	41	3.35	2.81
		Clinformatics	51,239	17,376	41	12	9.86	8.51	51,239	17,376	562	106	10.66	8.56
		MDCD	8,073	2,196	13	<5	19.76	<27.98	8,073	2,196	103	15	17.82	12.13
		MDCR	15,825	5,186	29	9	22.5	21.34	15,825	5,186	300	66	16.64	16.53
		OpenClaims	620,748	183,482	383	98	7.52	6.51	620,748	183,482	5,413	1,122	6.27	6.54
		OptumEHR	78,442	21,897	52	10	8.18	5.64	NA	NA	NA	NA	NA	NA
		VA	32,008	14,341	19	8	7.24	6.8	32,008	14,341	241	70	4.38	4.01
		Meta-analysis	872,954	266,852	563	<150	7.88	<6.87	NA	NA	NA	NA	NA	NA
	Venous thromboembolism	AmbEMR	57,262	15,281	52	17	11.07	13.56	57,262	15,281	201	68	10.81	12.05
		CCAE	66,528	22,358	70	23	12.96	12.67	66,528	22,358	650	152	9.96	10.48
		Clinformatics	51,153	17,355	104	31	25.08	22.03	51,153	17,355	949	207	18.23	16.87
		CPRD	9,118	11,390	<5	11	<6.70	11.79	9,118	11,390	130	177	7.35	6.79
		DAGermany	3,881	5,033	<5	6	<15.70	14.53	3,881	5,033	34	47	13.56	13.43
		IMRD	8,841	8,455	5	10	6.92	14.45	8,841	8,455	116	123	6.79	6.59
		MDCD	8,059	2,196	24	<5	36.57	<27.98	8,059	2,196	157	19	27.6	15.47
		MDCR	15,780	5,176	41	8	31.9	18.99	15,780	5,176	442	89	24.86	22.63
		OpenClaims	620,093	183,330	788	237	15.48	15.75	620,093	183,330	10,006	2,001	11.74	11.77
		OptumEHR	78,092	21,804	241	52	38.15	29.47	NA	NA	NA	NA	NA	NA
		VA	31,962	14,328	42	22	16.03	18.73	31,962	14,328	583	208	10.76	12.09
		Meta-analysis	950,769	306,706	<1,377	<422	<17.70	<16.81	872,677	284,902	13,268	3,091	12.03	11.32

Table S7, continued.

Comparison	Outcome	Database	30-day follow-up						On-treatment follow-up					
			Patients		Events		IR		Patients		Events		IR	
			T	C	T	C	T	C	T	C	T	C	T	C
HCQ vs SSZ	Gastrointestinal bleeding	CCAE	66,621	22,357	40	12	7.39	6.61	66,621	22,357	332	54	5.05	3.69
		Clinformatics	51,218	17,345	68	13	16.37	9.24	51,218	17,345	658	118	12.51	9.53
		MDCD	8,071	2,191	16	<5	24.33	<28.05	8,071	2,191	109	13	18.9	10.51
		MDCR	15,813	5,177	31	7	24.06	16.62	15,813	5,177	350	62	19.49	15.49
		OpenClaims	620,631	183,371	438	126	8.6	8.37	620,631	183,371	6,086	1,108	7.06	6.46
		OptumEHR	78,411	21,890	78	18	12.28	10.15	NA	NA	NA	NA	NA	NA
		VA	31,993	14,334	26	16	9.91	13.62	31,993	14,334	390	143	7.12	8.23
		Meta-analysis	872,758	266,665	697	<197	9.76	<9.03	NA	NA	NA	NA	NA	NA
	Acute renal failure	CCAE	66,584	22,362	72	27	13.32	14.87	66,584	22,362	526	96	8.01	6.59
		Clinformatics	51,166	17,353	163	54	39.32	38.39	51,166	17,353	1,377	298	26.47	24.32
		MDCD	8,051	2,189	41	13	62.61	73.1	8,051	2,189	256	44	45.23	36.48
		MDCR	15,797	5,181	60	17	46.67	40.37	15,797	5,181	608	133	34.22	33.69
		OpenClaims	620,101	183,354	1,061	308	20.85	20.47	620,101	183,354	13,812	2,404	16.18	14.1
		OptumEHR	78,010	21,797	186	42	29.45	23.81	NA	NA	NA	NA	NA	NA
		VA	31,947	14,317	102	35	38.98	29.84	31,947	14,317	1,214	307	22.63	17.85
		Meta-analysis	871,656	266,553	1,685	496	23.63	22.75	793,646	244,756	17,793	3,282	16.97	14.94
	End stage renal disease	AmbEMR	NA	NA	NA	NA	NA	NA	57,647	15,355	12	<5	0.64	<0.88
		CCAE	66,641	22,378	<5	<5	<0.92	<2.75	66,641	22,378	48	8	0.73	0.55
		Clinformatics	51,271	17,389	7	<5	1.68	<3.54	51,271	17,389	118	19	2.22	1.52
		MDCD	NA	NA	NA	NA	NA	NA	8,087	2,198	15	<5	2.56	<4.01
		MDCR	15,838	5,191	<5	<5	<3.87	<11.83	15,838	5,191	56	7	3.07	1.73
		OpenClaims	621,009	183,543	70	8	1.37	0.53	621,009	183,543	1,298	174	1.49	1.01
		OptumEHR	78,526	21,924	27	<5	4.24	<2.82	NA	NA	NA	NA	NA	NA
		VA	32,020	14,348	5	<5	1.9	<4.25	32,020	14,348	86	27	1.56	1.54
		Meta-analysis	865,305	264,773	<119	<33	<1.68	<1.52	852,513	260,402	1,633	<245	1.5	<1.07
	Hepatic failure	CCAE	66,638	22,378	6	<5	1.11	<2.75	66,638	22,378	50	11	0.76	0.75
		Clinformatics	51,267	17,387	13	<5	3.13	<3.54	51,267	17,387	102	26	1.92	2.09
		DAGermany	NA	NA	NA	NA	NA	NA	3,887	5,047	<5	<5	<1.95	<1.40
		MDCD	8,085	2,198	<5	<5	<7.58	<27.95	8,085	2,198	25	6	4.26	4.82
		MDCR	NA	NA	NA	NA	NA	NA	15,841	5,189	32	5	1.75	1.23
		OpenClaims	621,042	183,551	42	23	0.82	1.53	621,042	183,551	729	151	0.84	0.87
		OptumEHR	78,529	21,916	32	9	5.03	5.07	NA	NA	NA	NA	NA	NA
		VA	32,025	14,348	5	<5	1.9	<4.25	32,025	14,348	61	14	1.1	0.8
		Meta-analysis	857,586	261,778	<103	<52	<1.47	<2.43	798,785	250,098	<1,004	<218	<0.94	<0.96
	Acute pancreatitis	CCAE	66,637	22,374	22	7	4.06	3.85	66,637	22,374	143	34	2.17	2.32
		Clinformatics	51,259	17,384	21	9	5.05	6.38	51,259	17,384	171	50	3.22	4.02
		MDCD	8,077	2,196	10	<5	15.19	<27.97	8,077	2,196	55	8	9.47	6.45
		MDCR	15,833	5,188	6	<5	4.65	<11.84	15,833	5,188	62	15	3.4	3.7
		OpenClaims	620,956	183,525	155	50	3.04	3.32	620,956	183,525	1,881	390	2.17	2.26
		OptumEHR	78,522	21,921	21	5	3.3	2.82	NA	NA	NA	NA	NA	NA
		VA	32,023	14,347	12	<5	4.57	<4.25	32,023	14,347	106	46	1.92	2.63
		Meta-analysis	873,307	266,935	247	<86	3.45	<3.94	794,785	245,014	2,418	543	2.27	2.44

Table S7, continued.

Comparison	Outcome	Database	30-day follow-up						On-treatment follow-up					
			Patients		Events		IR		Patients		Events		IR	
			T	C	T	C	T	C	T	C	T	C	T	C
		Clinformatics	23,597	24,521	17	17	8.88	8.55	23,597	24,521	268	276	15.56	18.85
		VA	6,234	8,005	91	52	179.23	79.42	6,234	8,005	550	518	51.16	45.97
		Meta-analysis	29,831	32,526	108	69	44.58	26.12	29,831	32,526	818	794	29.24	30.64
		CCAE	32,610	32,507	13	11	4.92	4.17	32,610	32,507	117	94	4.33	4.33
		Clinformatics	23,565	24,484	30	29	15.7	14.62	23,565	24,484	179	147	10.6	10.19
	All-cause mortality	MDCD	3,803	3,808	<5	6	<16.21	19.4	3,803	3,808	29	27	11.46	13.46
		MDCR	8,119	9,254	16	9	24.33	11.96	8,119	9,254	166	140	20.41	17.34
		OpenClaims	216,028	232,938	182	173	10.26	9.05	216,028	232,938	2,065	1,732	8.11	7.94
		OptumEHR	18,477	16,424	26	20	17.35	15.01	NA	NA	NA	NA	NA	NA
		VA	6,203	7,978	33	19	65.53	29.15	6,203	7,978	154	127	14.79	11.59
		Meta-analysis	308,805	327,393	<305	267	<12.08	9.97	290,328	310,969	2,710	2,267	8.48	8.24
	Cardiovascular events	CCAE	32,540	32,453	68	57	25.8	21.67	32,540	32,453	467	351	17.73	16.54
		Clinformatics	23,434	24,361	147	137	77.54	69.59	23,434	24,361	723	669	45.56	49.38
		MDCD	3,759	3,781	31	30	102.07	97.98	3,759	3,781	157	122	69.38	67.74
		MDCR	8,030	9,188	93	59	143.76	79.2	8,030	9,188	657	573	92.6	78.6
		OpenClaims	215,278	232,274	901	784	51.08	41.18	215,278	232,274	7,835	6,884	32.54	33.34
		OptumEHR	17,963	16,221	123	80	84.71	60.92	NA	NA	NA	NA	NA	NA
		VA	6,047	7,866	183	87	378.81	136.03	6,047	7,866	544	445	58.44	44.08
AZM vs AMX		Meta-analysis	NA	NA	NA	NA	NA	NA	289,088	309,923	10,383	9,044	34.42	34.73
	Cardiac arrhythmia	AmbEMR	13,076	11,977	27	25	25.18	25.46	13,076	11,977	116	90	20.7	18.79
		CCAE	32,510	32,423	113	67	42.94	25.5	32,510	32,423	711	486	28.53	23.76
		Clinformatics	23,475	24,403	90	76	47.34	38.49	23,475	24,403	556	482	35.52	36.2
		MDCD	3,781	3,787	14	18	45.72	58.59	3,781	3,787	88	77	37.42	41.8
		MDCR	8,084	9,210	45	48	68.86	64.24	8,084	9,210	354	361	47.87	49.98
		OpenClaims	215,473	232,391	568	589	32.15	30.91	215,473	232,391	5,724	4,989	23.82	24.22
		OptumEHR	18,022	16,138	153	111	105.13	85.09	NA	NA	NA	NA	NA	NA
		VA	6,168	7,935	58	47	116.1	72.64	6,168	7,935	297	289	31.93	29.25
	Bradycardia	AmbEMR	NA	NA	NA	NA	NA	NA	13,193	12,080	14	14	2.36	2.81
		CCAE	32,619	32,516	<5	<5	<1.89	<1.90	32,619	32,516	43	50	1.58	2.29
		Clinformatics	23,580	24,509	10	17	5.23	8.56	23,580	24,509	108	96	6.34	6.61
		MDCD	3,807	3,810	<5	<5	<16.19	<16.15	3,807	3,810	19	11	7.41	5.42
		MDCR	8,124	9,268	5	<5	7.59	<6.63	8,124	9,268	70	86	8.51	10.63
		OpenClaims	216,124	233,035	72	78	4.06	4.08	216,124	233,035	1,115	1,069	4.35	4.88
		OptumEHR	18,546	16,432	15	23	9.97	17.26	NA	NA	NA	NA	NA	NA
		VA	6,227	8,000	<5	<5	<9.86	<7.64	6,227	8,000	64	60	6.1	5.42
		Meta-analysis	309,027	327,570	<117	<138	<4.63	<5.15	303,674	323,218	1,433	1,386	4.37	4.93

Table S7, continued.

Comparison	Outcome	Database	30-day follow-up						On-treatment follow-up					
			Patients		Events		IR		Patients		Events		IR	
			T	C	T	C	T	C	T	C	T	C	T	C
AZM vs AMX	Transient ischemic attack	CCAE	32,609	32,505	5	11	1.89	4.17	32,609	32,505	111	77	4.11	3.55
		Clinformatics	23,577	24,501	20	24	10.46	12.09	23,577	24,501	122	120	7.18	8.32
		MDCD	3,802	3,808	7	<5	22.72	<16.16	3,802	3,808	27	15	10.62	7.51
		MDCR	8,121	9,259	9	10	13.67	13.28	8,121	9,259	118	102	14.53	12.6
		OpenClaims	216,102	232,985	96	102	5.41	5.33	216,102	232,985	1,306	1,102	5.11	5.04
		OptumEHR	18,563	16,456	9	5	5.98	3.74	NA	NA	NA	NA	NA	NA
		VA	6,231	8,000	12	5	23.67	7.64	6,231	8,000	54	41	5.11	3.69
		Meta-analysis	309,005	327,514	158	<162	6.25	<6.05	290,442	311,058	1,738	1,457	5.42	5.28
	Stroke	CCAE	32,609	32,508	12	11	4.54	4.17	32,609	32,508	131	94	4.85	4.33
		Clinformatics	23,572	24,490	33	34	17.26	17.14	23,572	24,490	174	157	10.28	10.87
		MDCD	3,803	3,808	7	7	22.71	22.63	3,803	3,808	36	29	14.16	14.55
		MDCR	8,118	9,257	17	13	25.85	17.27	8,118	9,257	156	173	19.33	21.57
		OpenClaims	216,089	232,948	155	159	8.74	8.32	216,089	232,948	2,063	1,858	8.12	8.55
		OptumEHR	18,538	16,433	25	9	16.63	6.75	NA	NA	NA	NA	NA	NA
		VA	6,215	7,995	10	15	19.77	22.96	6,215	7,995	76	83	7.23	7.5
		Meta-analysis	308,944	327,439	259	248	10.25	9.26	290,406	311,006	2,636	2,394	8.26	8.72
	Venous thromboembolism	AmbEMR	13,133	12,024	11	19	10.21	19.27	13,133	12,024	51	57	8.71	11.72
		CCAE	32,559	32,467	40	40	15.16	15.2	32,559	32,467	339	227	12.8	10.59
		Clinformatics	23,540	24,459	54	63	28.3	31.82	23,540	24,459	330	303	20.08	21.49
		MDCD	3,793	3,797	8	18	26.03	58.49	3,793	3,797	81	61	33.31	31.49
		MDCR	8,104	9,239	23	24	35.04	31.96	8,104	9,239	228	204	28.93	26.03
		OpenClaims	215,805	232,676	320	357	18.07	18.7	215,805	232,676	3,662	3,137	14.74	14.73
		OptumEHR	18,394	16,335	112	57	75.28	43.08	NA	NA	NA	NA	NA	NA
		VA	6,203	7,977	32	23	63.53	35.31	6,203	7,977	175	165	17.32	15.45
		Meta-analysis	321,531	338,974	600	601	22.82	21.68	303,137	322,639	4,866	4,154	15.32	15.17
	Gastrointestinal bleeding	CCAE	32,609	32,486	20	31	7.56	11.77	32,609	32,486	137	128	5.08	5.9
		Clinformatics	23,568	24,453	39	37	20.41	18.68	23,568	24,453	227	189	13.45	13.14
		MDCD	3,805	3,797	8	11	25.93	35.67	3,805	3,797	58	35	23.17	17.82
		MDCR	8,118	9,231	16	28	24.33	37.34	8,118	9,231	172	170	21.23	21.34
		OpenClaims	216,032	232,795	213	220	12.01	11.52	216,032	232,795	2,245	2,009	8.85	9.26
		OptumEHR	18,530	16,391	37	22	24.62	16.54	NA	NA	NA	NA	NA	NA
		VA	6,211	7,952	31	31	61.43	47.76	6,211	7,952	122	125	11.67	11.44
		Meta-analysis	308,873	327,105	364	380	14.41	14.2	290,343	310,714	2,961	2,656	9.3	9.69
	Acute renal failure	CCAE	32,576	32,451	50	36	18.94	13.68	32,576	32,451	286	220	10.67	10.23
		Clinformatics	23,534	24,413	104	87	54.57	44.05	23,534	24,413	476	444	28.9	31.72
		MDCD	3,774	3,789	21	27	68.81	87.98	3,774	3,789	110	77	46.36	40.34
		MDCR	8,091	9,224	47	31	71.85	41.38	8,091	9,224	348	311	44.2	40.07
		OpenClaims	215,716	232,429	500	603	28.26	31.64	215,716	232,429	4,977	4,565	20.04	21.49
		OptumEHR	18,134	16,262	86	70	58.59	53.16	NA	NA	NA	NA	NA	NA
		VA	6,133	7,907	146	117	296.69	182.62	6,133	7,907	416	410	42.7	39.97
		Meta-analysis	NA	NA	NA	NA	NA	NA	289,824	310,213	6,613	6,027	21.22	22.5

Table S7, continued.

Comparison	Outcome	Database	30-day follow-up						On-treatment follow-up					
			Patients		Events		IR		Patients		Events		IR	
			T	C	T	C	T	C	T	C	T	C	T	C
AZM vs AMX	End stage renal disease	AmbEMR	NA	NA	NA	NA	NA	NA	13,197	12,079	7	5	1.18	1
		CCAE	32,620	32,518	<5	<5	<1.89	<1.90	32,620	32,518	30	20	1.1	0.91
		Clinformatics	23,594	24,521	<5	6	<2.61	3.02	23,594	24,521	31	36	1.8	2.46
		MDCD	3,806	3,809	<5	<5	<16.20	<16.15	3,806	3,809	18	10	6.96	4.96
		MDCR	8,130	9,270	7	<5	10.62	<6.63	8,130	9,270	23	28	2.75	3.4
		OpenClaims	216,166	233,078	36	41	2.03	2.14	216,166	233,078	558	507	2.16	2.3
		OptumEHR	18,557	16,465	9	5	5.98	3.74	NA	NA	NA	NA	NA	NA
		VA	6,232	8,003	8	<5	15.77	<7.64	6,232	8,003	37	38	3.48	3.39
		Meta-analysis	309,105	327,664	<75	<72	<2.97	<2.69	303,745	323,278	704	644	2.13	2.27
	Hepatic failure	CCAE	32,621	32,519	<5	5	<1.89	1.9	32,621	32,519	30	28	1.1	1.28
		Clinformatics	23,592	24,516	<5	<5	<2.61	<2.52	23,592	24,516	38	27	2.2	1.84
		MDCD	3,807	3,809	<5	<5	<16.19	<16.15	3,807	3,809	6	6	2.3	2.94
		MDCR	8,130	9,269	<5	<5	<7.58	<6.63	8,130	9,269	18	15	2.15	1.81
		OpenClaims	216,181	233,088	22	27	1.24	1.41	216,181	233,088	306	311	1.18	1.41
		OptumEHR	18,548	16,466	15	11	9.97	8.23	NA	NA	NA	NA	NA	NA
		VA	6,233	8,005	6	<5	11.82	<7.64	6,233	8,005	26	19	2.42	1.69
		Meta-analysis	309,112	327,672	<63	<63	<2.49	<2.35	290,564	311,206	424	406	1.31	1.45
	Acute pancreatitis	CCAE	32,611	32,513	6	8	2.27	3.03	32,611	32,513	70	55	2.58	2.52
		Clinformatics	23,590	24,511	11	10	5.75	5.03	23,590	24,511	62	49	3.62	3.36
		MDCD	3,800	3,808	<5	5	<16.22	16.16	3,800	3,808	23	21	9.06	10.48
		MDCR	8,129	9,268	<5	<5	<7.59	<6.63	8,129	9,268	32	32	3.84	3.89
		OpenClaims	216,145	232,998	64	80	3.61	4.18	216,145	232,998	713	595	2.77	2.7
		OptumEHR	18,572	16,463	7	9	4.65	6.74	NA	NA	NA	NA	NA	NA
		VA	6,232	7,995	7	6	13.8	9.18	6,232	7,995	39	41	3.68	3.68
		Meta-analysis	309,079	327,556	<105	<123	<4.15	<4.59	290,507	311,093	939	793	2.91	2.85

T=target therapy; C=comparator therapy; IR=incidence rate; py=person-years at risk; NA=non-applicable (not reported because of failed diagnostics or on-treatment follow-up unavailable); CV=cardiovascular; HCQ=hydroxychloroquine; SSZ=sulfasalazine; AZM=HCQ+Azithromycin; AMX=HCQ+amoxicillin; AmbEMR=IQVIA Ambulatory EMR; CCAE=IBM Commercial Database; Clinformatics=Optum de-identified Clinformatics Data Mart Database; CPRD=Clinical Practice Research Datalink; DAGermany=IQVIA Disease Analyzer Germany; IMRD=IQVIA UK Integrated Medical Record Data; MDCD=IBM IBM Multi-state Medicaid; MDCR=IBM Medicare Supplemental Database; OpenClaims=IQVIA Open Claims; OptumEHR=Optum de-identified Electronic Health Record dataset; VA=Department of Veterans Affairs

S8. Calibrated hazard ratio forest plots
S8.1. 30-day follow-up
S8.1.1. All-cause mortality

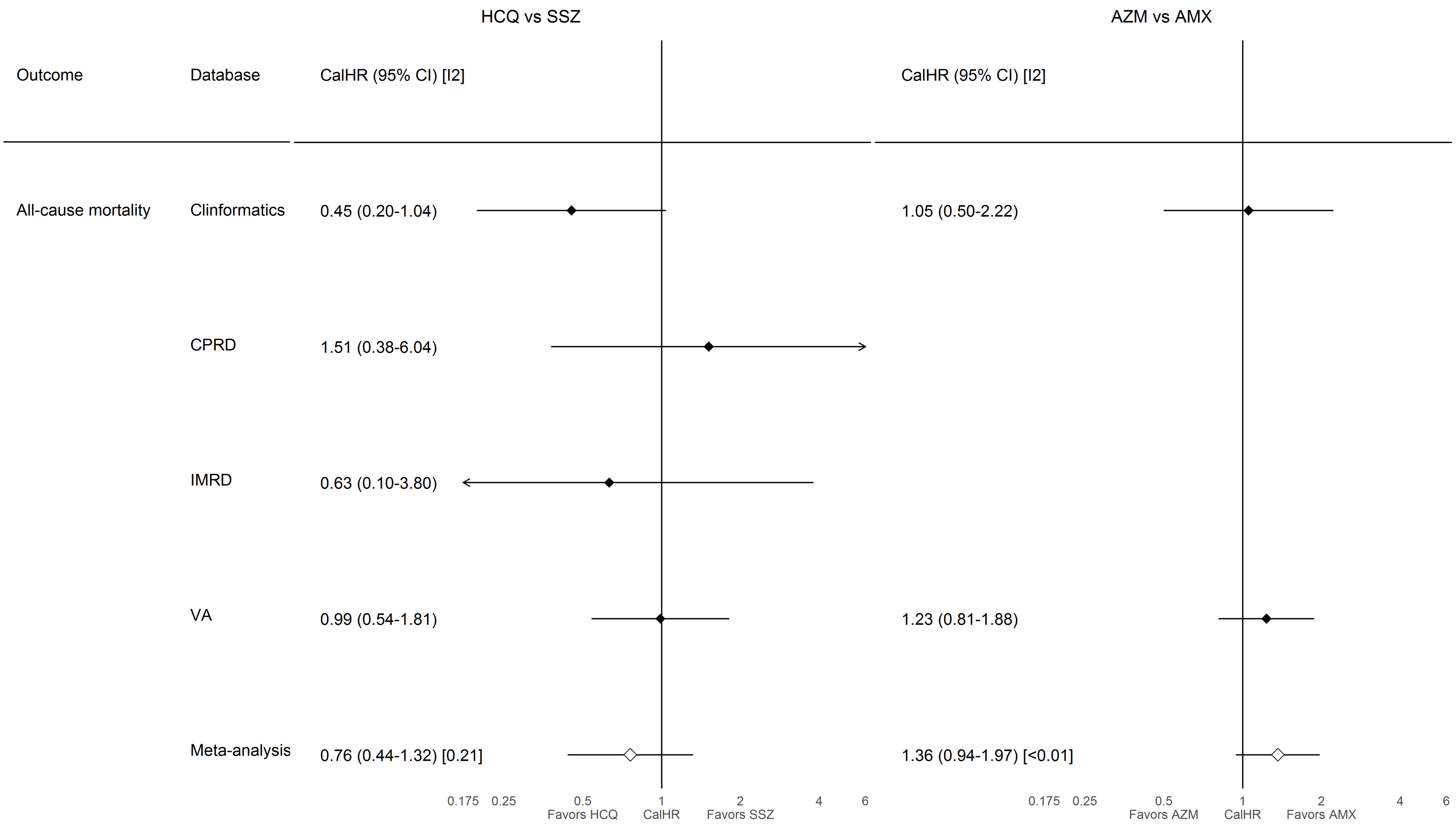

S8.1.2. CV-related mortality

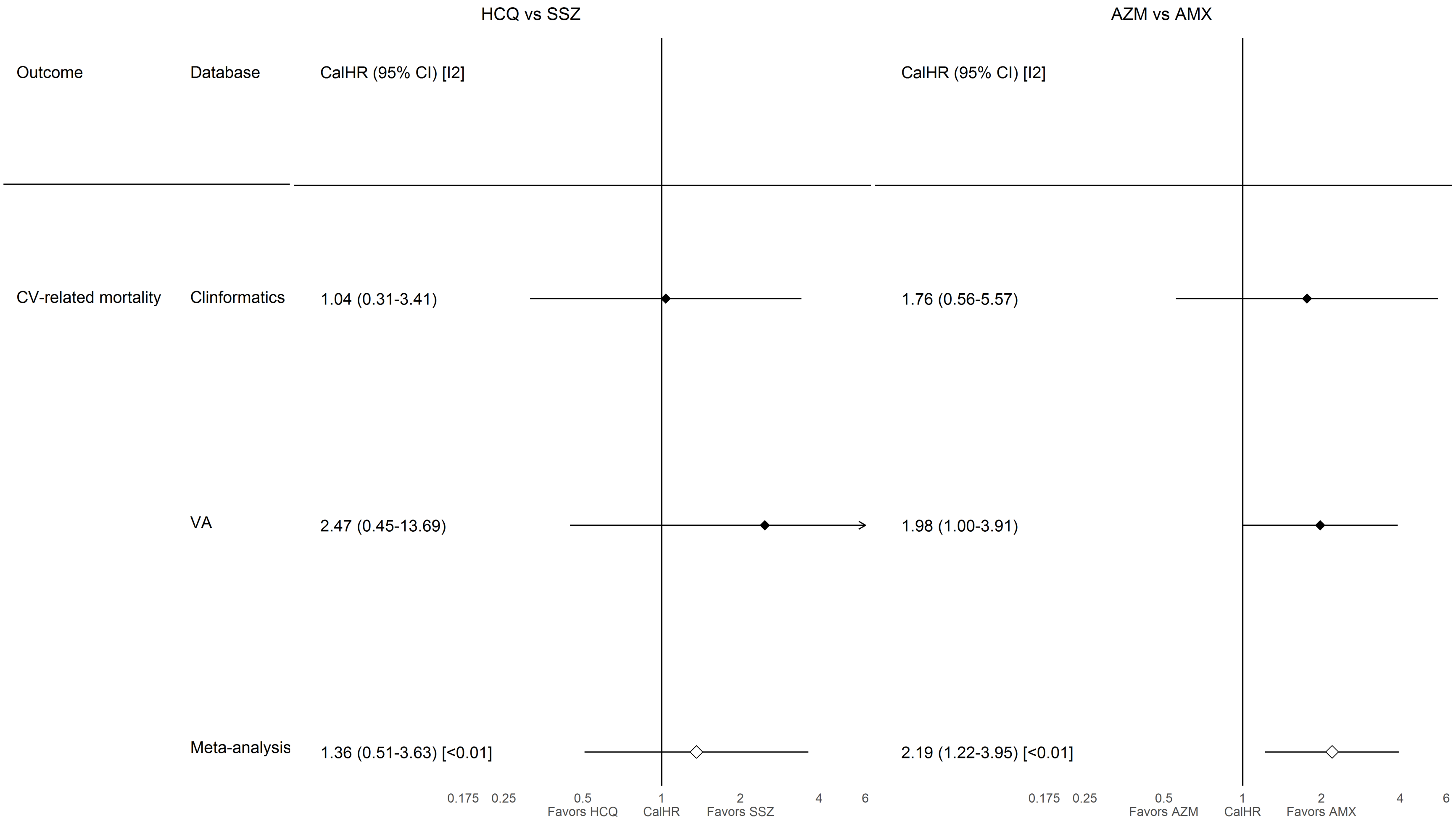

S8.1.3. Myocardial infarction

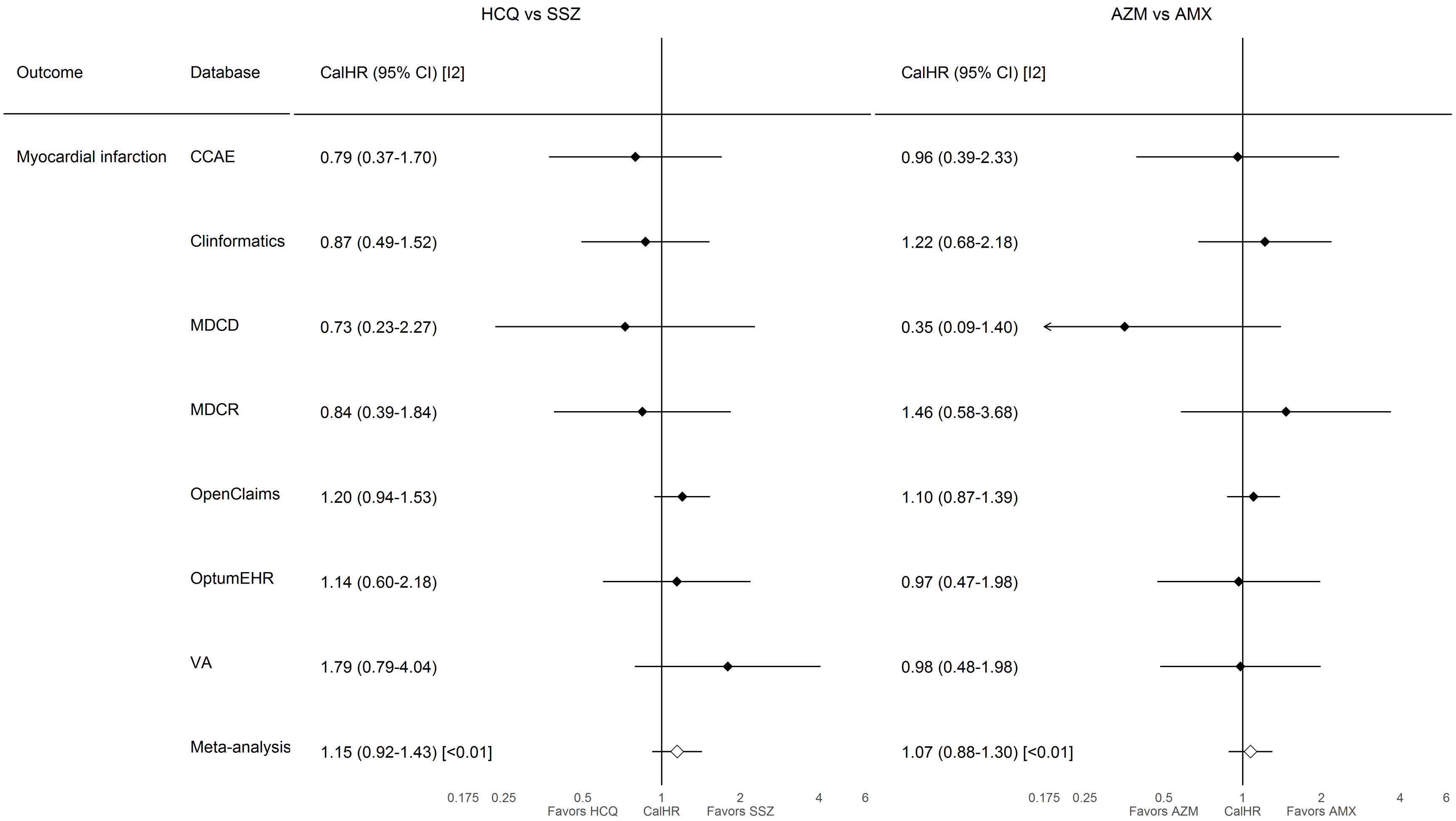

S8.1.4. Chest pain or angina

Outcome	Database	HCQ vs SSZ CalHR (95% CI) [I2]	AZM vs AMX CalHR (95% CI) [I2]
Chest pain or angina	AmbEMR	1.08 (0.71-1.66)	1.42 (0.78-2.59)
	CCAE	0.91 (0.72-1.14)	1.02 (0.82-1.25)
	Clinformatics	0.82 (0.66-1.01)	1.22 (0.98-1.52)
	CPRD	0.90 (0.36-2.23)	
	DAGermany	0.41 (0.08-2.06)	
	IMRD	1.11 (0.39-3.17)	
	MDCD	0.96 (0.58-1.59)	0.98 (0.57-1.69)
	MDCR	0.93 (0.66-1.30)	1.10 (0.77-1.56)
	OpenClaims	0.91 (0.83-1.00)	1.23 (1.10-1.36)
	OptumEHR	1.15 (0.95-1.40)	1.08 (0.84-1.40)
	VA	1.04 (0.73-1.48)	0.81 (0.51-1.28)
	Meta-analysis	0.96 (0.84-1.09) [<0.01]	1.15 (1.05-1.26) [<0.01]

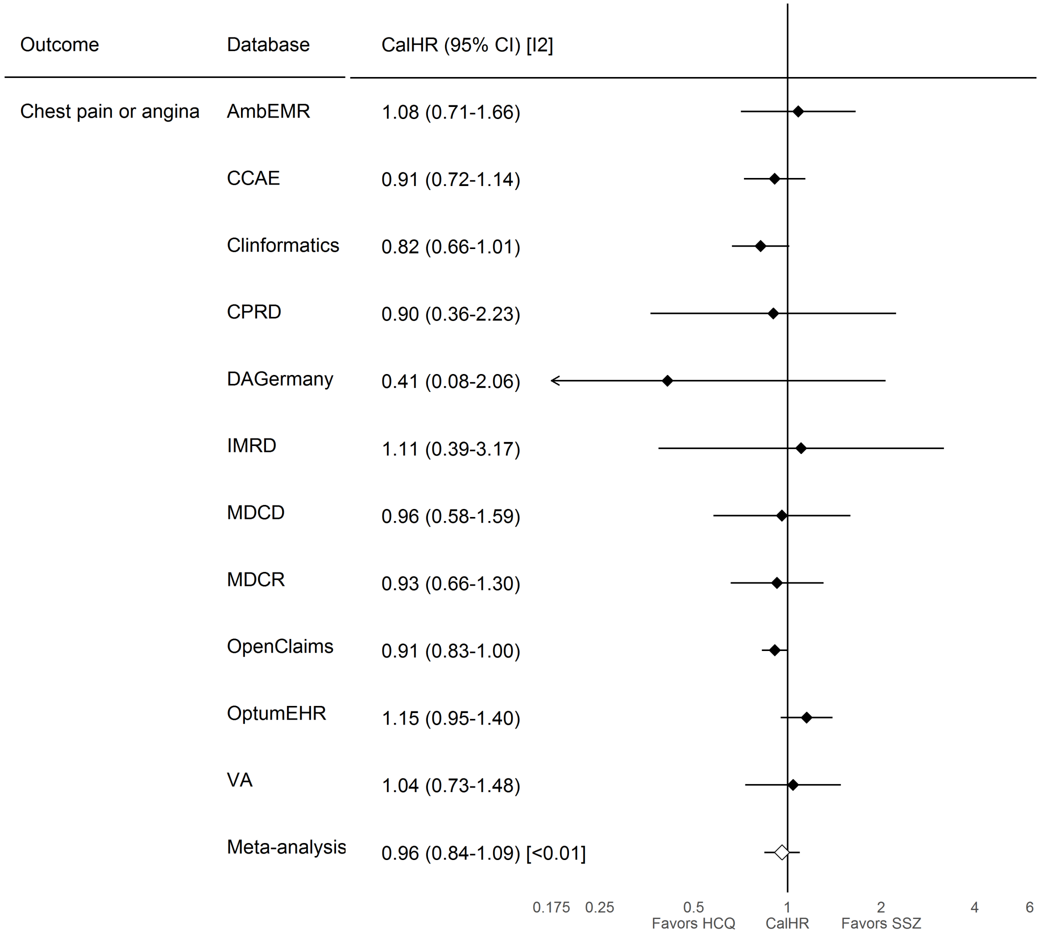

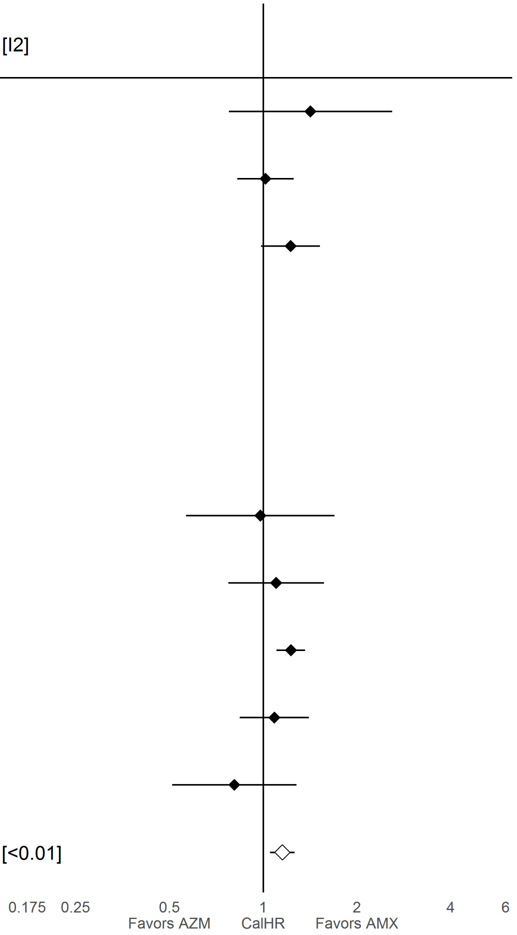

S8.1.5. Heart failure

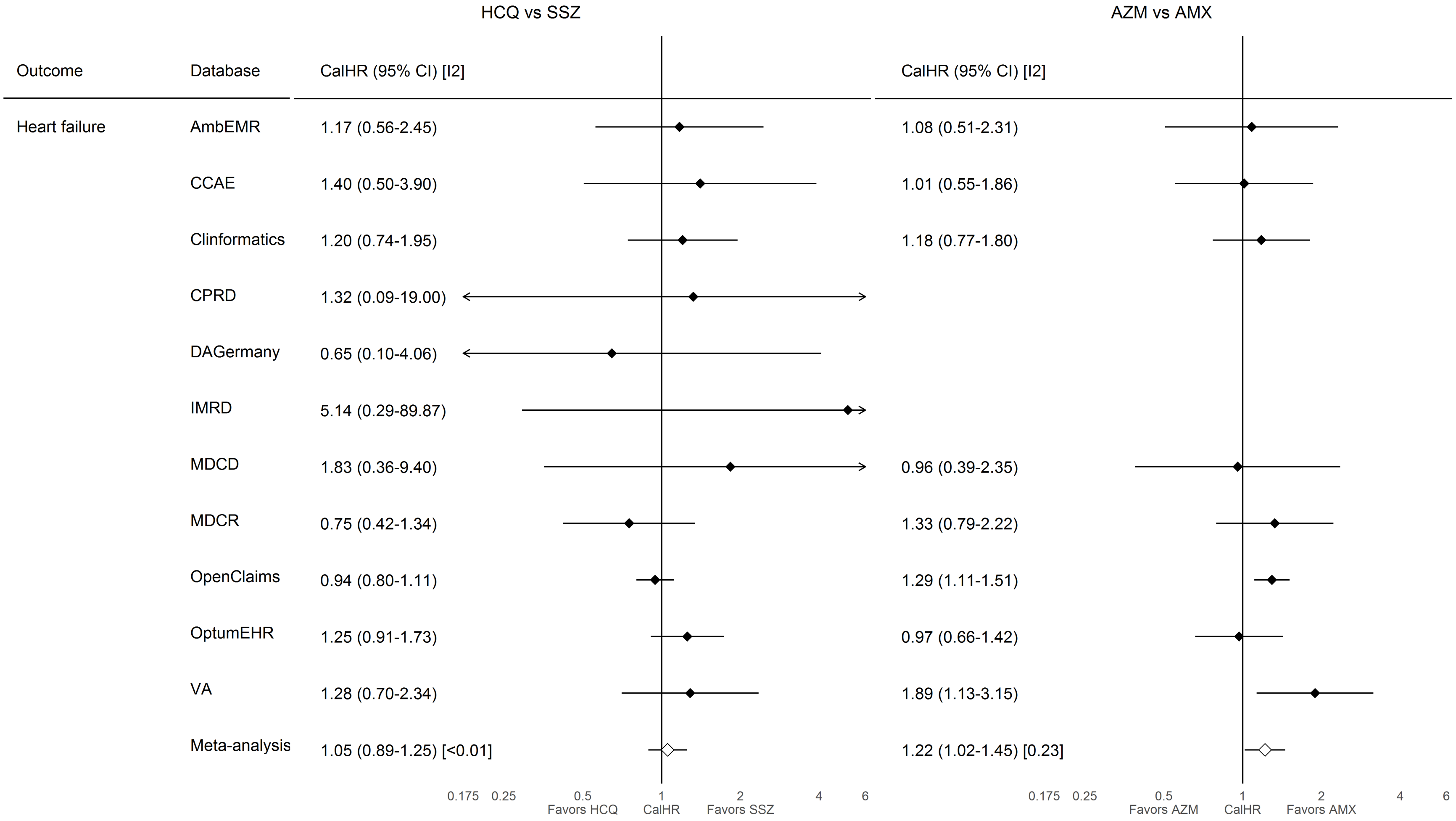

S8.1.6. Cardiovascular events

Outcome	Database	HCQ vs SSZ CalHR (95% CI) [I2]	AZM vs AMX CalHR (95% CI) [I2]
Cardiovascular events	CCAE	0.72 (0.47-1.10)	0.93 (0.63-1.39)
	Clinformatics	0.97 (0.72-1.29)	1.07 (0.81-1.40)
	MDCD	0.85 (0.40-1.80)	0.82 (0.46-1.43)
	MDCR	1.02 (0.70-1.50)	1.64 (1.13-2.38)
	OpenClaims	0.96 (0.86-1.08)	1.24 (1.10-1.38)
	OptumEHR	1.05 (0.80-1.39)	1.04 (0.74-1.47)
	VA	1.24 (0.85-1.80)	1.48 (1.07-2.04)
	Meta-analysis	1.01 (0.87-1.16) [<0.01]	

S8.1.7. Cardiac arrhythmia

Outcome	Database	HCQ vs SSZ CalHR (95% CI) [I2]	AZM vs AMX CalHR (95% CI) [I2]
Cardiac arrhythmia	AmbEMR	0.97 (0.67-1.40)	1.09 (0.60-1.97)
	CCAE	0.78 (0.55-1.09)	1.73 (1.24-2.43)
	Clinformatics	0.88 (0.64-1.22)	1.22 (0.86-1.72)
	CPRD	0.56 (0.20-1.59)	
	DAGermany	0.83 (0.18-3.78)	
	IMRD	0.74 (0.26-2.11)	
	MDCD	1.08 (0.50-2.30)	0.96 (0.43-2.14)
	MDCR	1.05 (0.65-1.70)	0.97 (0.61-1.55)
	OpenClaims	0.82 (0.73-0.92)	0.99 (0.87-1.14)
	OptumEHR	1.11 (0.89-1.37)	0.86 (0.64-1.15)
	VA	0.93 (0.64-1.37)	0.98 (0.60-1.60)
	Meta-analysis	0.90 (0.78-1.03) [<0.01]	

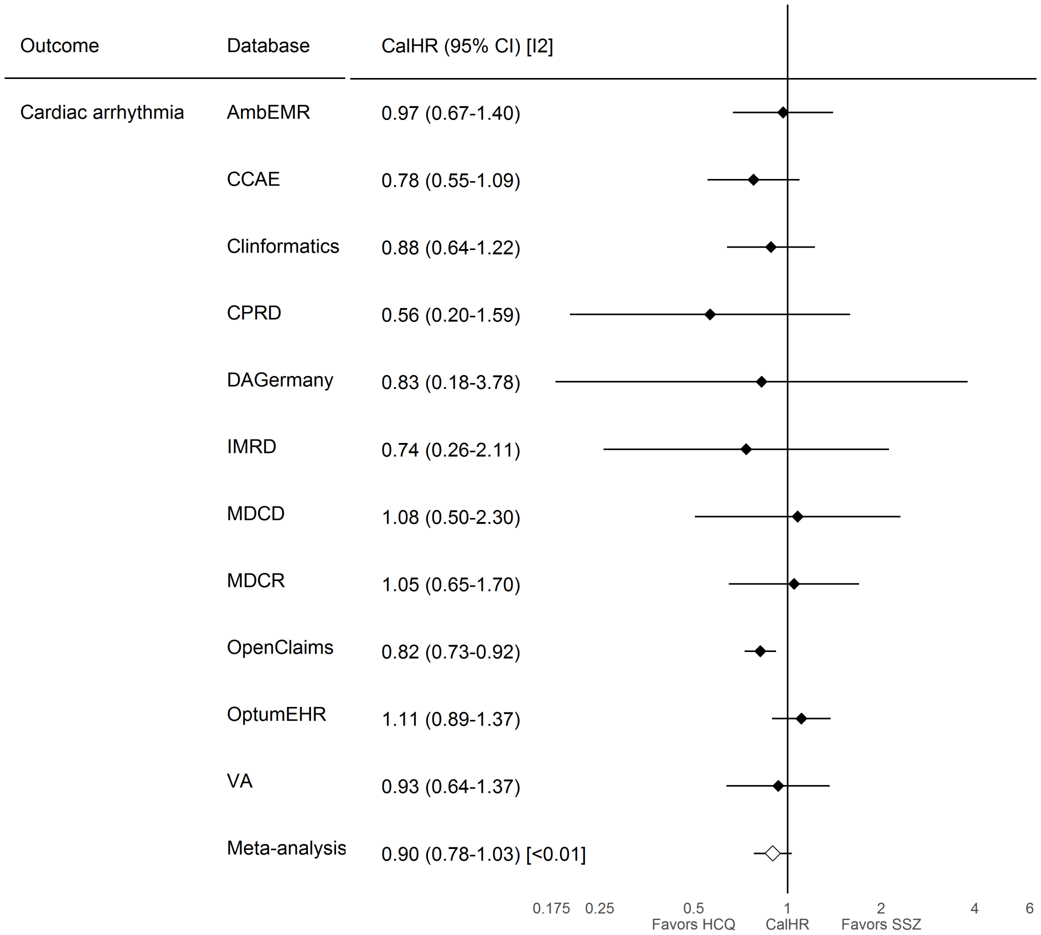

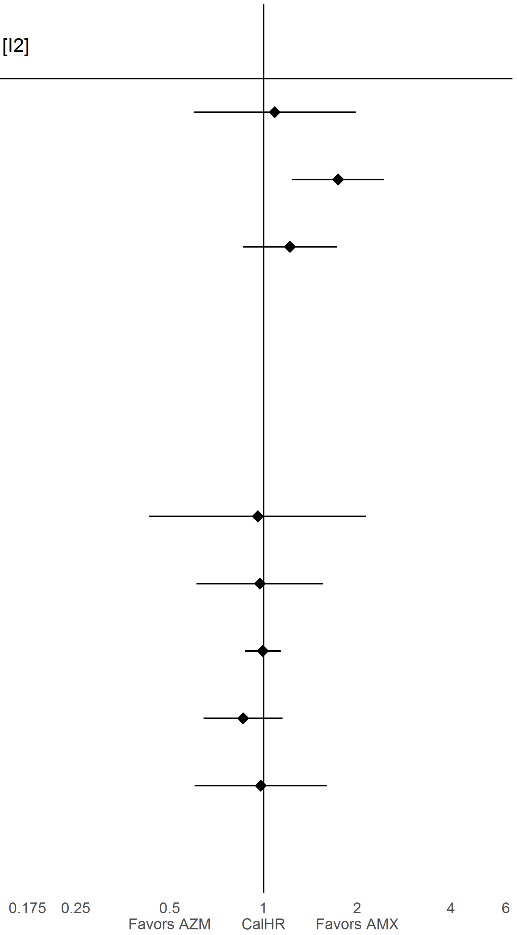

S8.1.8. Bradycardia

Outcome	Database	HCQ vs SSZ CalHR (95% CI) [I2]	AZM vs AMX CalHR (95% CI) [I2]
Bradycardia	AmbEMR	1.59 (0.42-5.97)	
	CCAE	0.75 (0.16-3.59)	0.99 (0.16-6.03)
	Clinformatics	1.83 (0.59-5.68)	0.59 (0.24-1.45)
	MDCD	1.71 (0.14-20.83)	1.54 (0.06-39.86)
	MDCR		0.64 (0.16-2.64)
	OpenClaims	0.78 (0.56-1.07)	1.17 (0.82-1.66)
	OptumEHR	0.86 (0.48-1.53)	0.54 (0.25-1.19)
	VA	0.69 (0.19-2.48)	0.78 (0.11-5.42)
	Meta-analysis	0.88 (0.66-1.16) [<0.01]	0.93 (0.70-1.25) [<0.01]

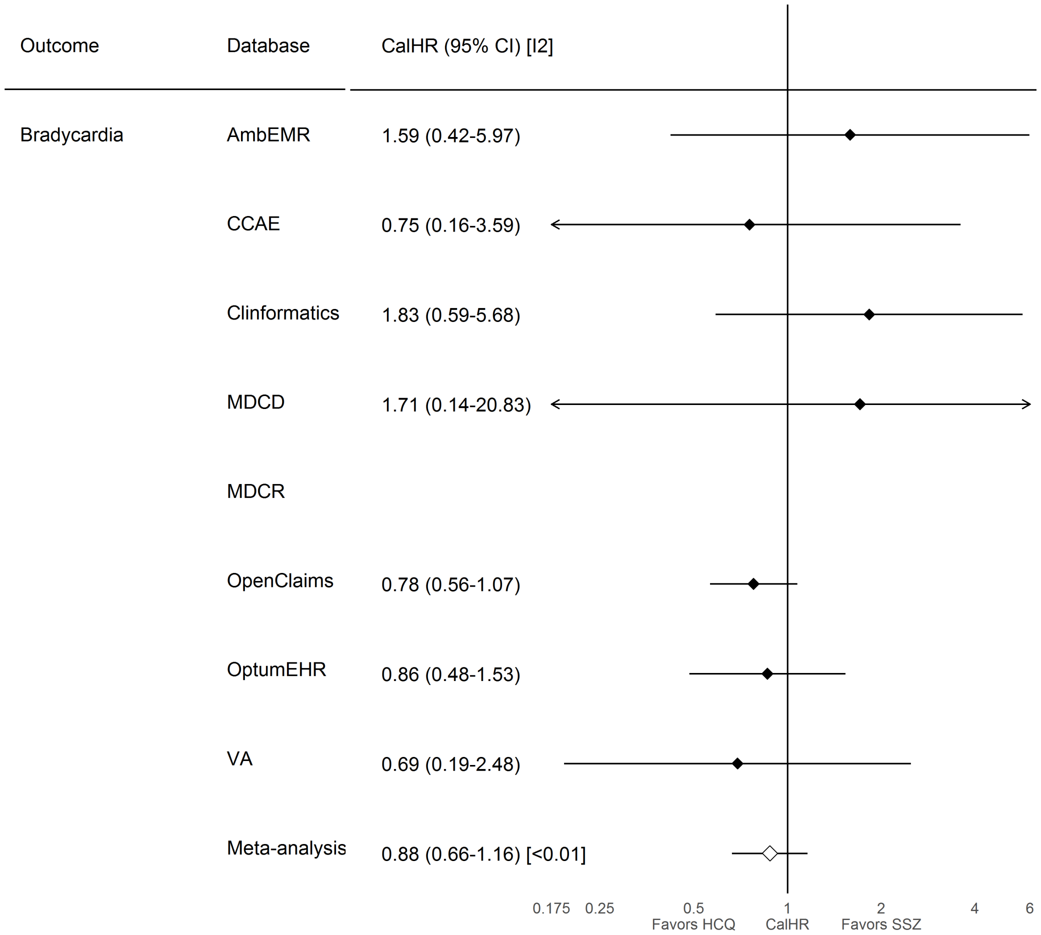

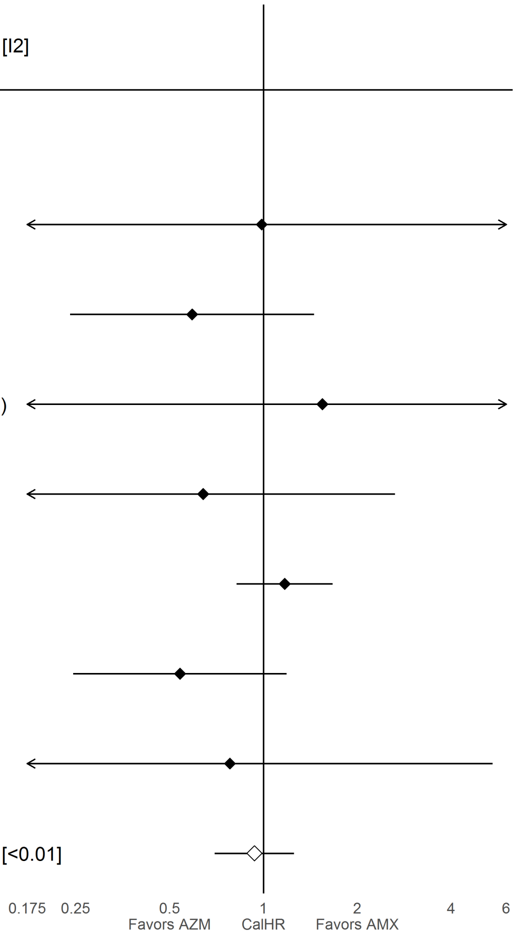

S8.1.9. Transient ischemic attack

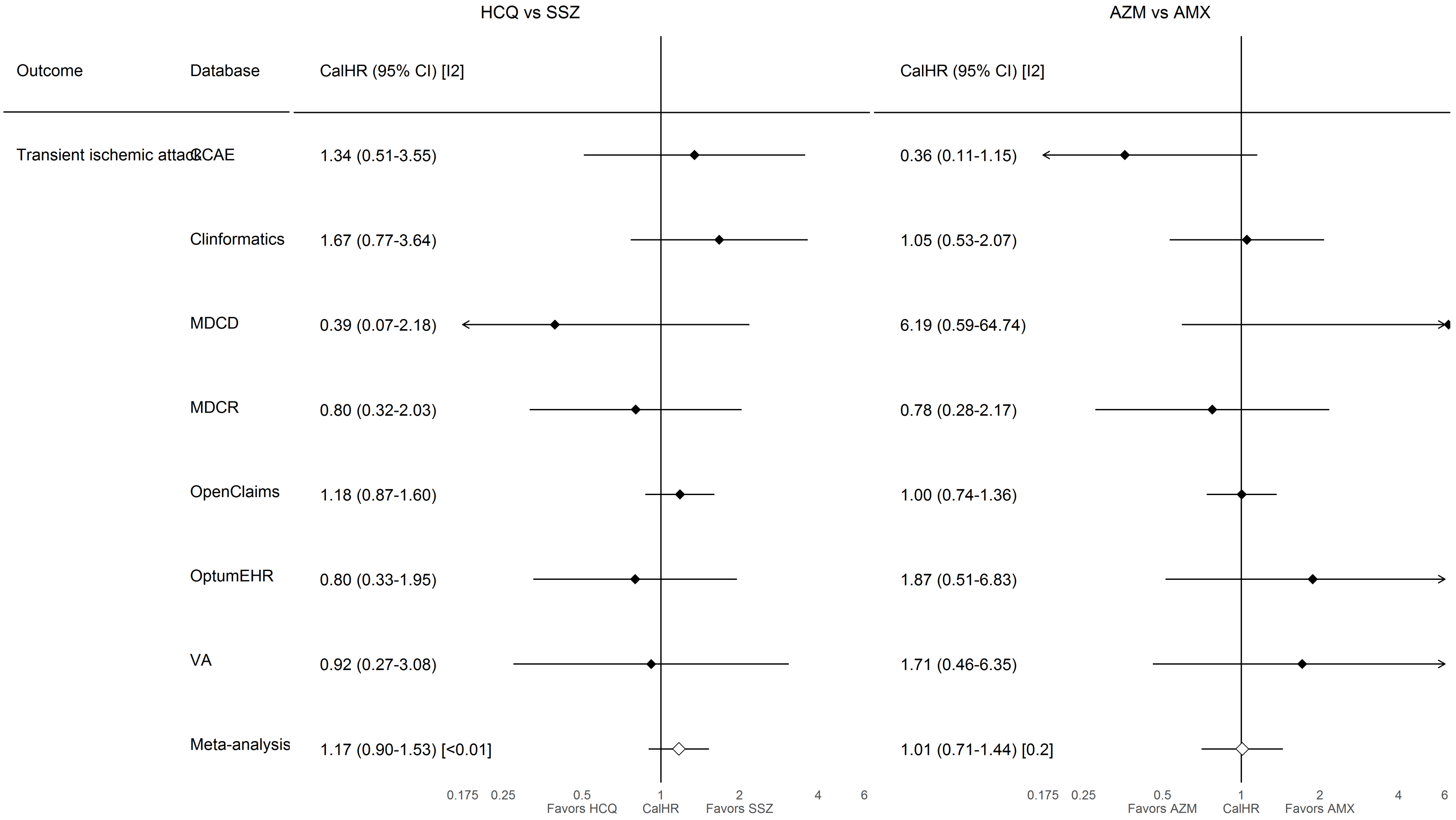

S8.1.10. Stroke

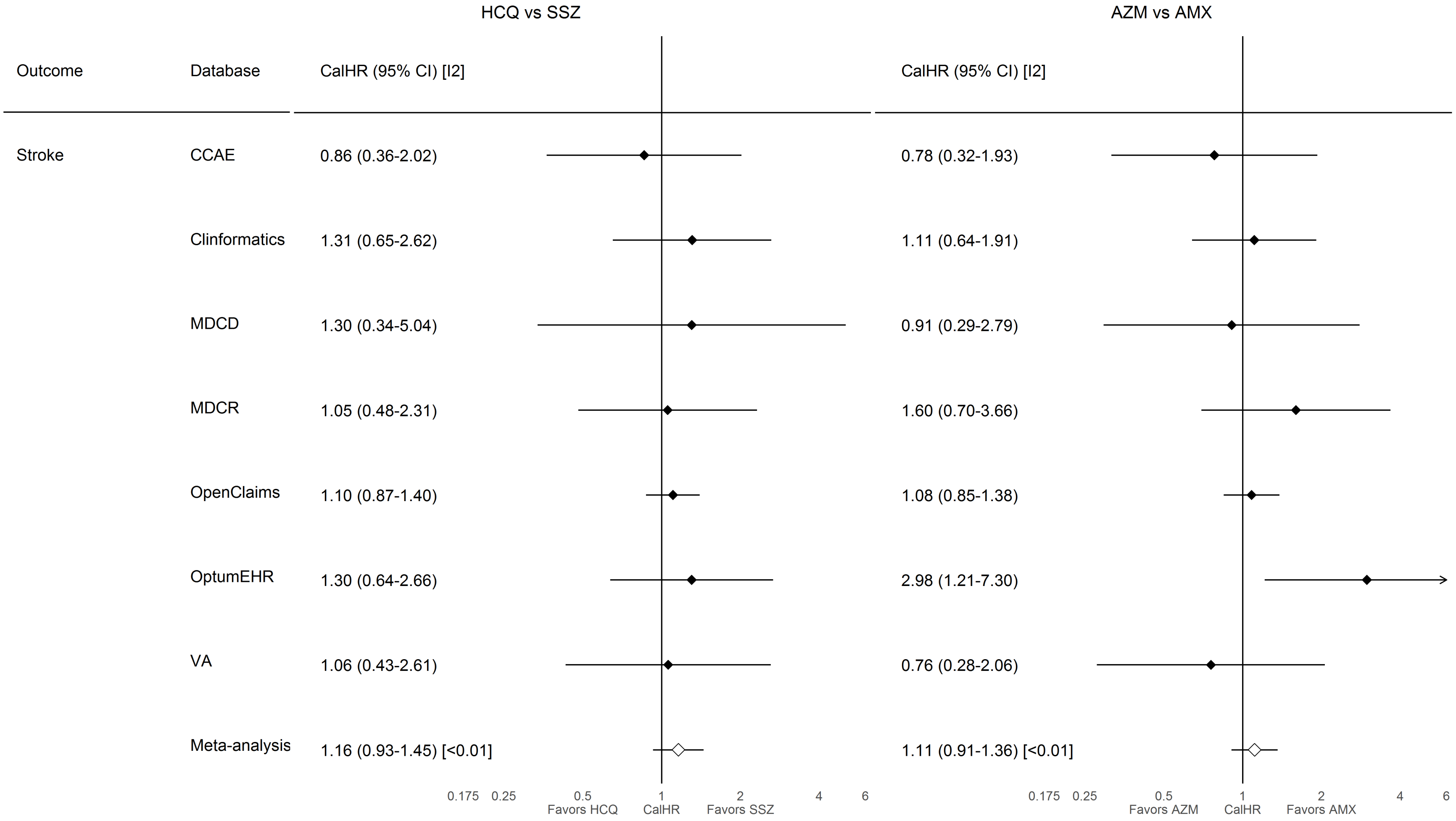

S8.1.11. Venous thromboembolism

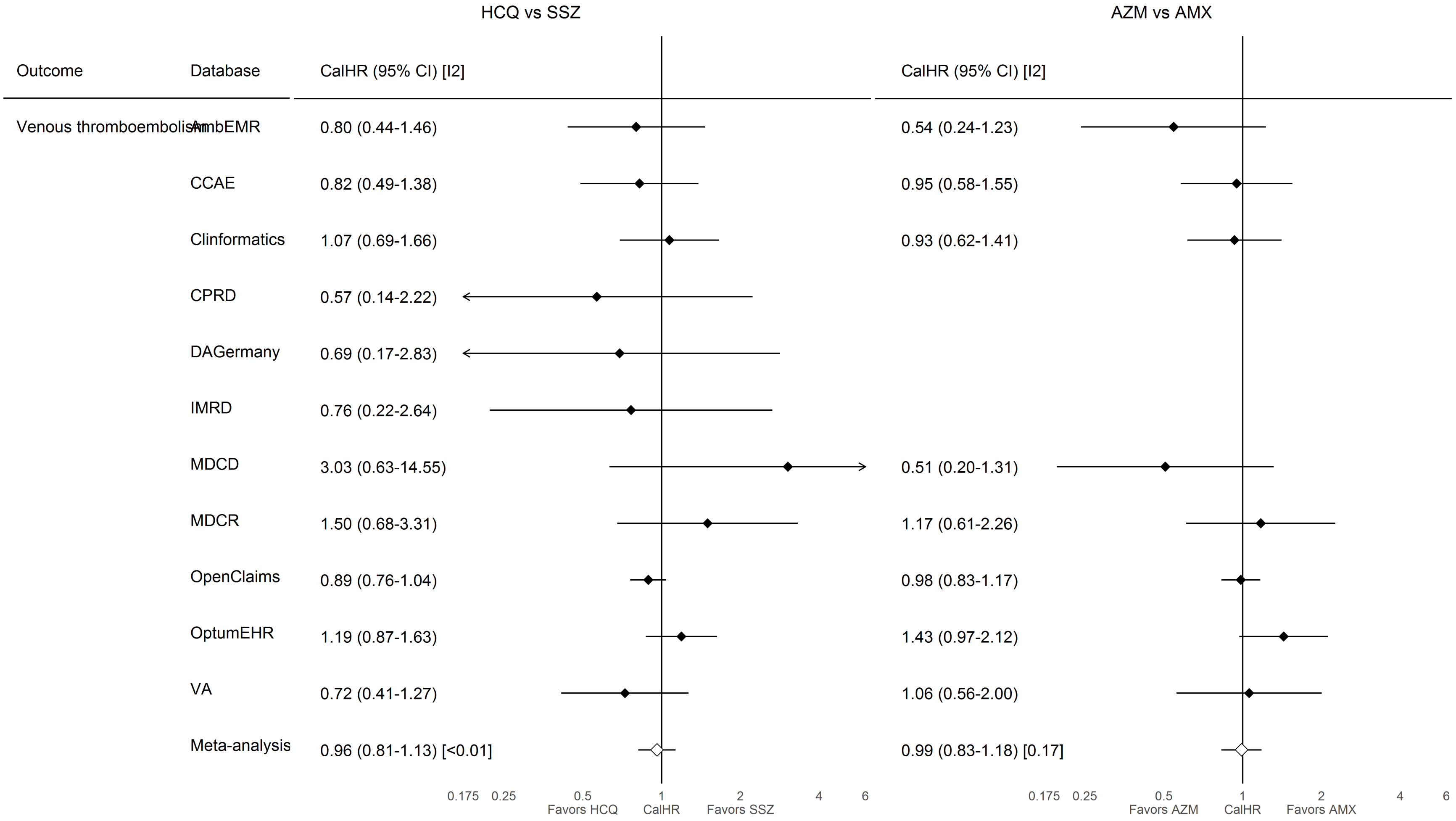

S8.1.12. Gastrointestinal bleeding

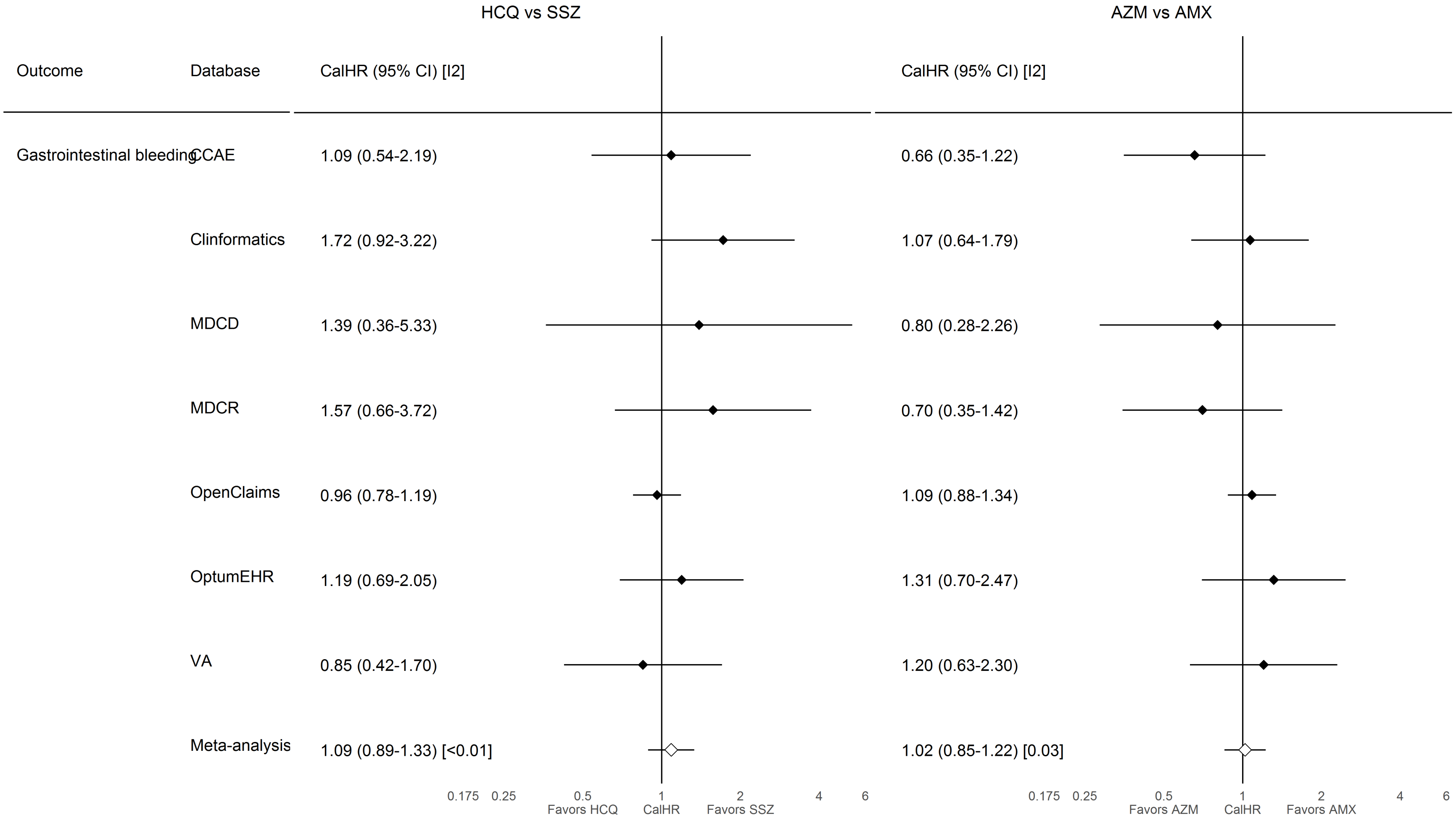

S8.1.13. Acute renal failure

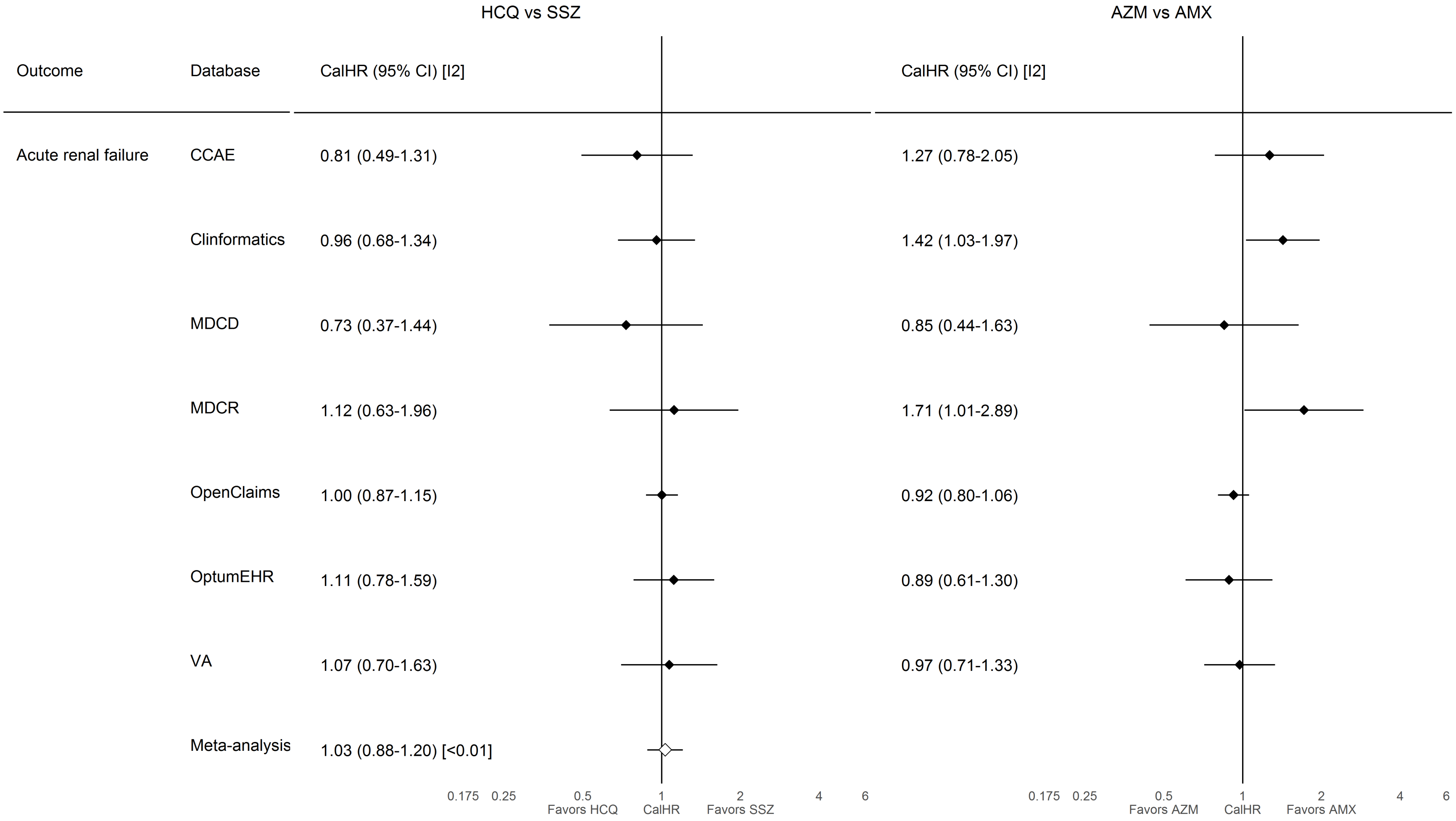

S8.1.14. End stage renal disease

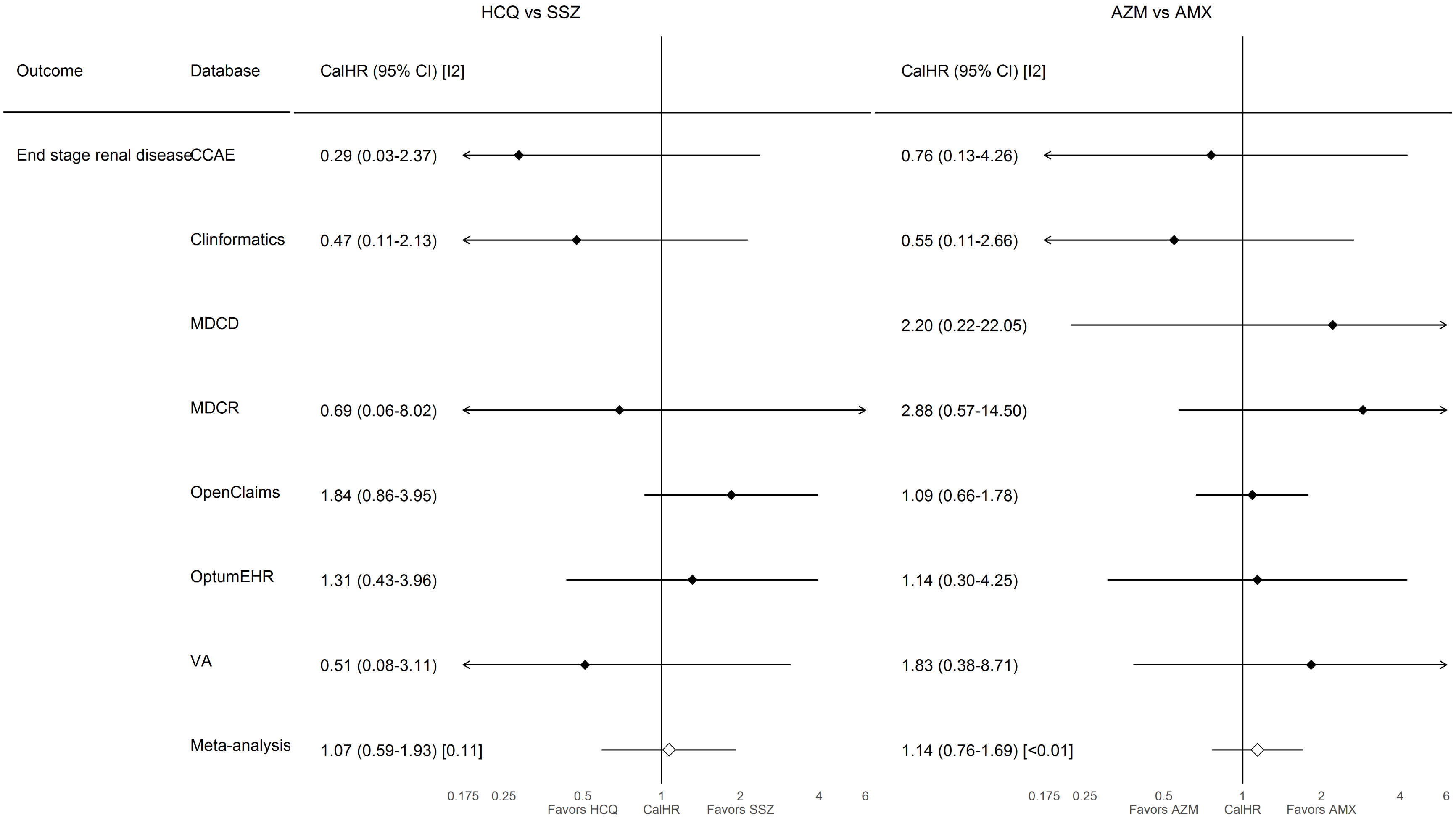

S8.1.15. Hepatic failure

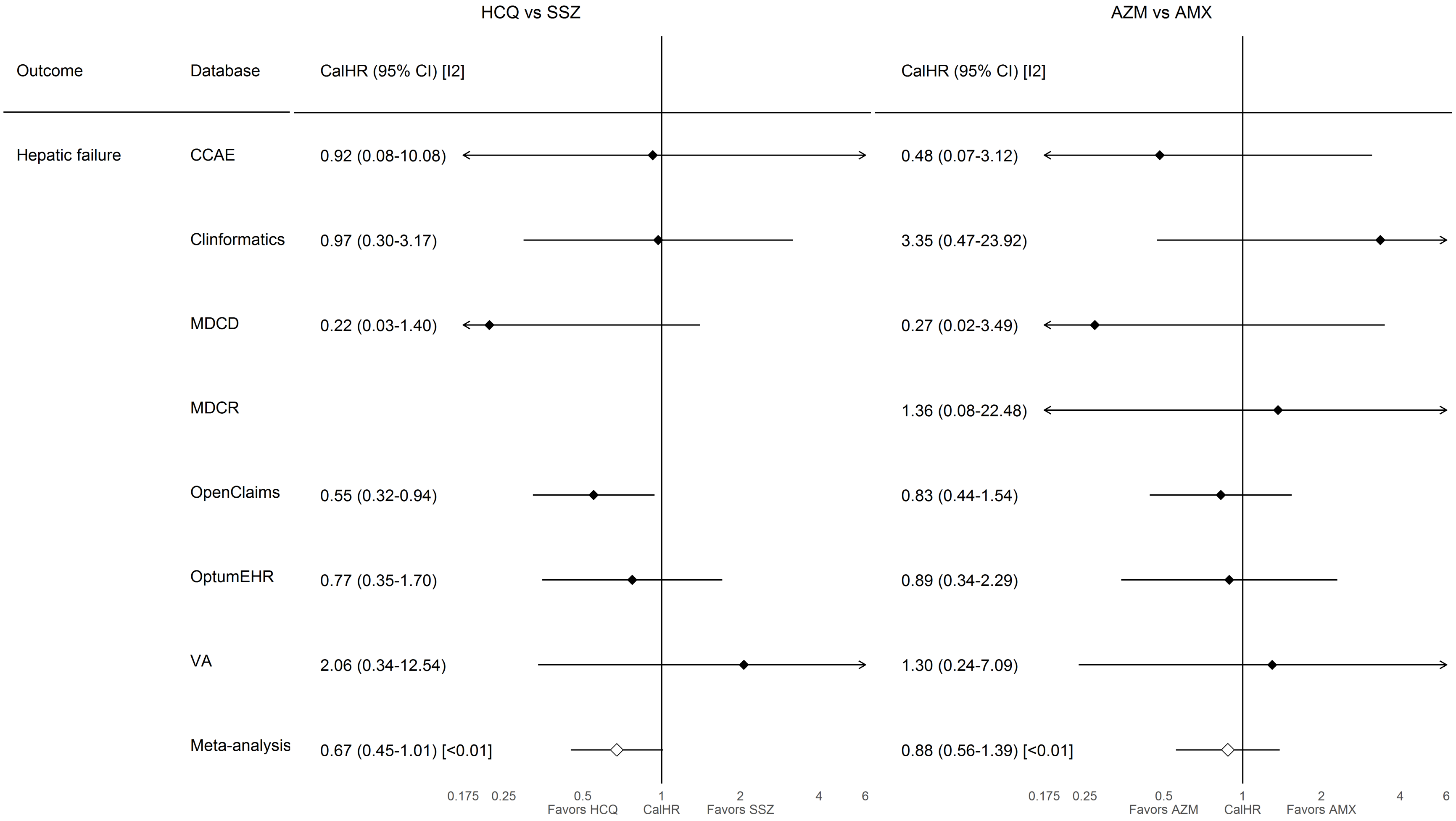

S8.1.16. Acute pancreatitis

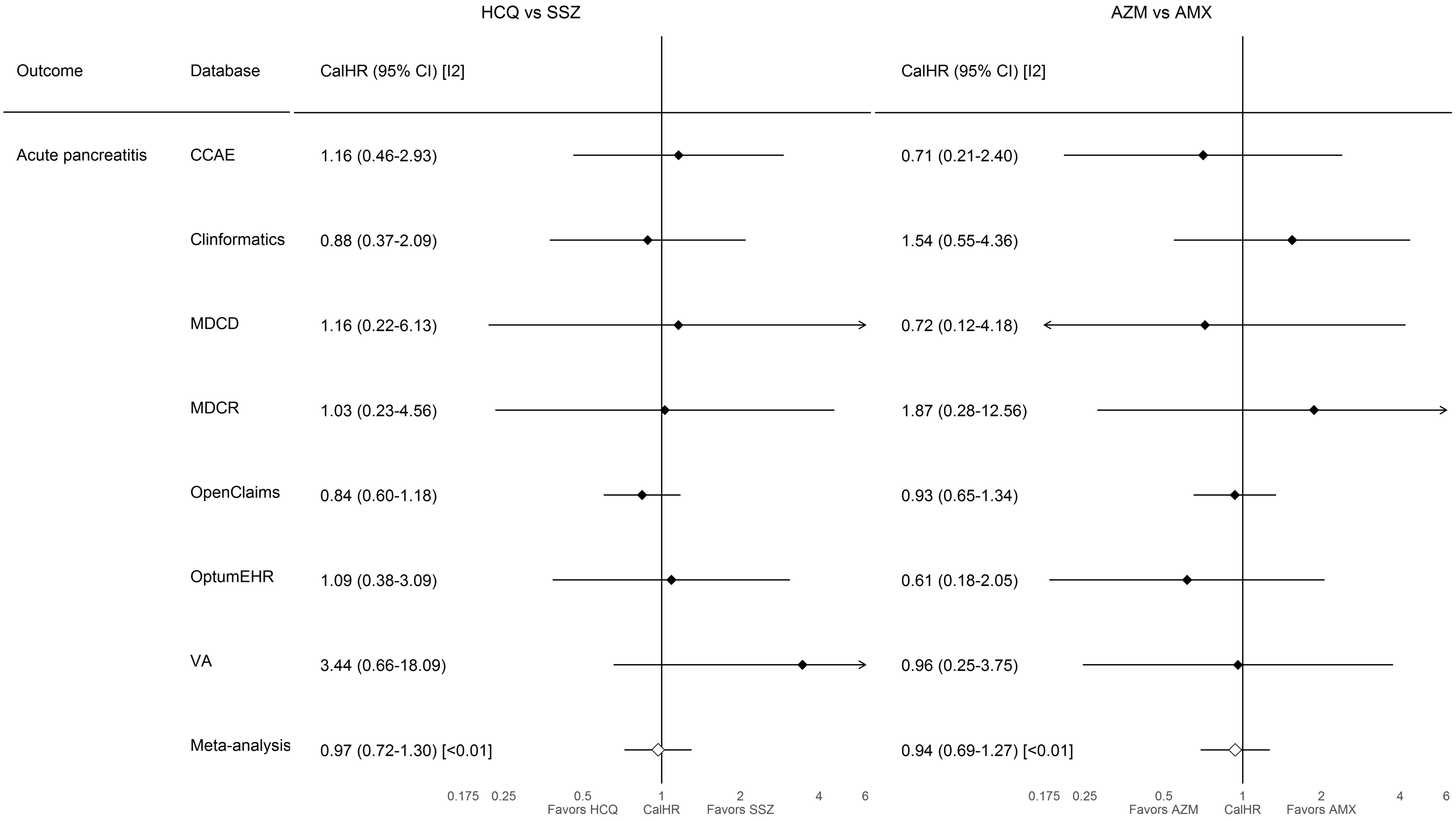

Outcome	Database	HCQ vs SSZ CalHR (95% CI) [I2]	AZM vs AMX CalHR (95% CI) [I2]
Acute pancreatitis	CCAE	1.16 (0.46-2.93)	0.71 (0.21-2.40)
	Clinformatics	0.88 (0.37-2.09)	1.54 (0.55-4.36)
	MDCD	1.16 (0.22-6.13)	0.72 (0.12-4.18)
	MDCR	1.03 (0.23-4.56)	1.87 (0.28-12.56)
	OpenClaims	0.84 (0.60-1.18)	0.93 (0.65-1.34)
	OptumEHR	1.09 (0.38-3.09)	0.61 (0.18-2.05)
	VA	3.44 (0.66-18.09)	0.96 (0.25-3.75)
	Meta-analysis	0.97 (0.72-1.30) [<0.01]	0.94 (0.69-1.27) [<0.01]

S8.2. On-treatment follow-up

S8.2.1. All-cause mortality

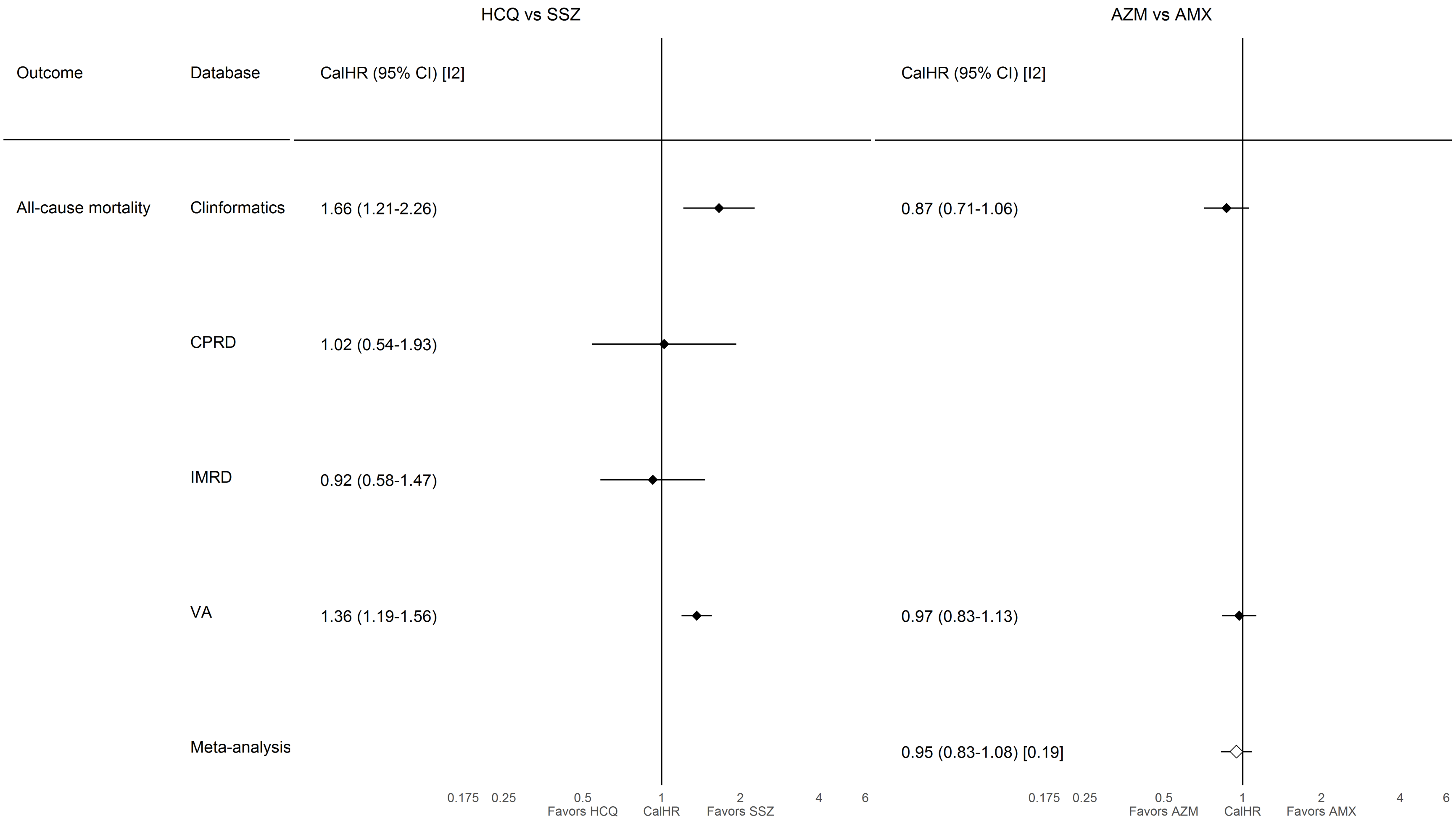

S8.2.2. CV-related mortality

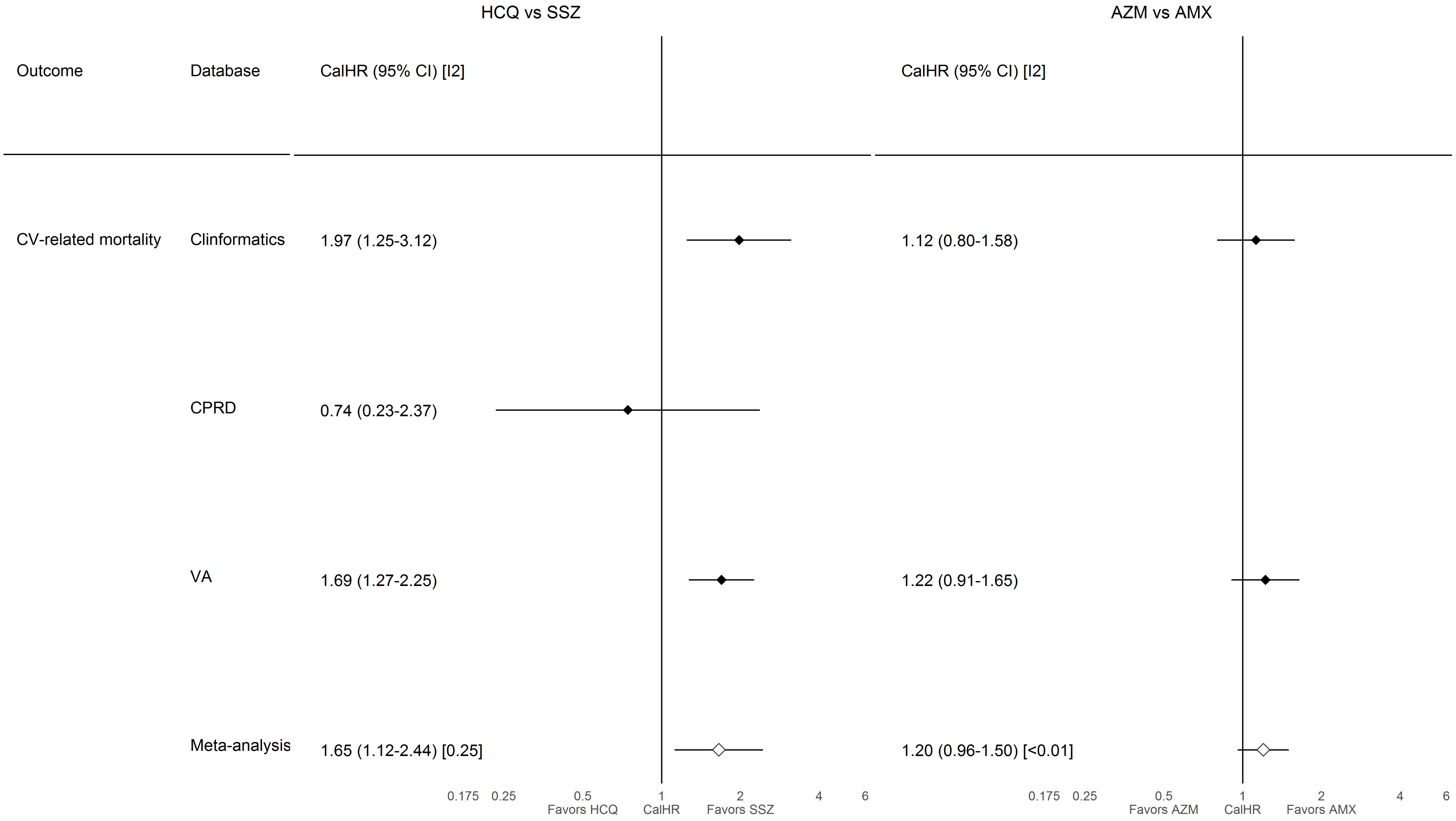

S8.2.3. Myocardial infarction

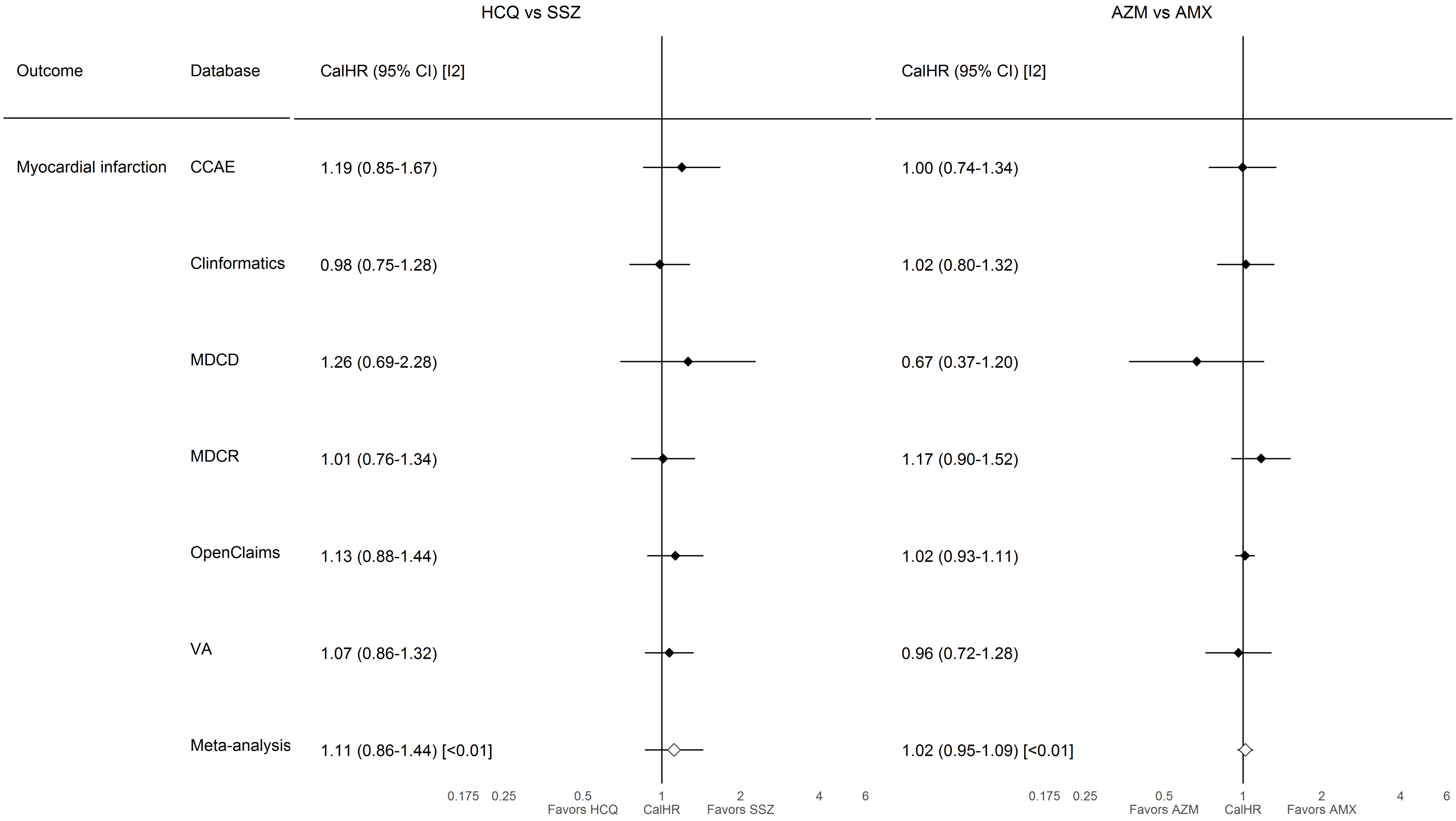

S8.2.4. Chest pain or angina

Outcome	Database	HCQ vs SSZ CalHR (95% CI) [I2]	AZM vs AMX CalHR (95% CI) [I2]
Chest pain or angina	AmbEMR	1.07 (0.86-1.35)	0.94 (0.72-1.22)
	CCAE	1.00 (0.88-1.14)	0.98 (0.90-1.08)
	Clinformatics	0.99 (0.82-1.19)	1.01 (0.91-1.13)
	CPRD	0.92 (0.49-1.72)	
	DAGermany	0.86 (0.51-1.45)	
	IMRD	0.81 (0.52-1.28)	
	MDCD	1.07 (0.85-1.34)	1.14 (0.85-1.52)
	MDCR	1.06 (0.91-1.23)	1.13 (0.97-1.32)
	OpenClaims	1.00 (0.79-1.27)	0.96 (0.91-1.02)
	VA	1.02 (0.92-1.14)	0.88 (0.73-1.05)
	Meta-analysis	1.01 (0.79-1.30) [<0.01]	0.98 (0.94-1.02) [<0.01]

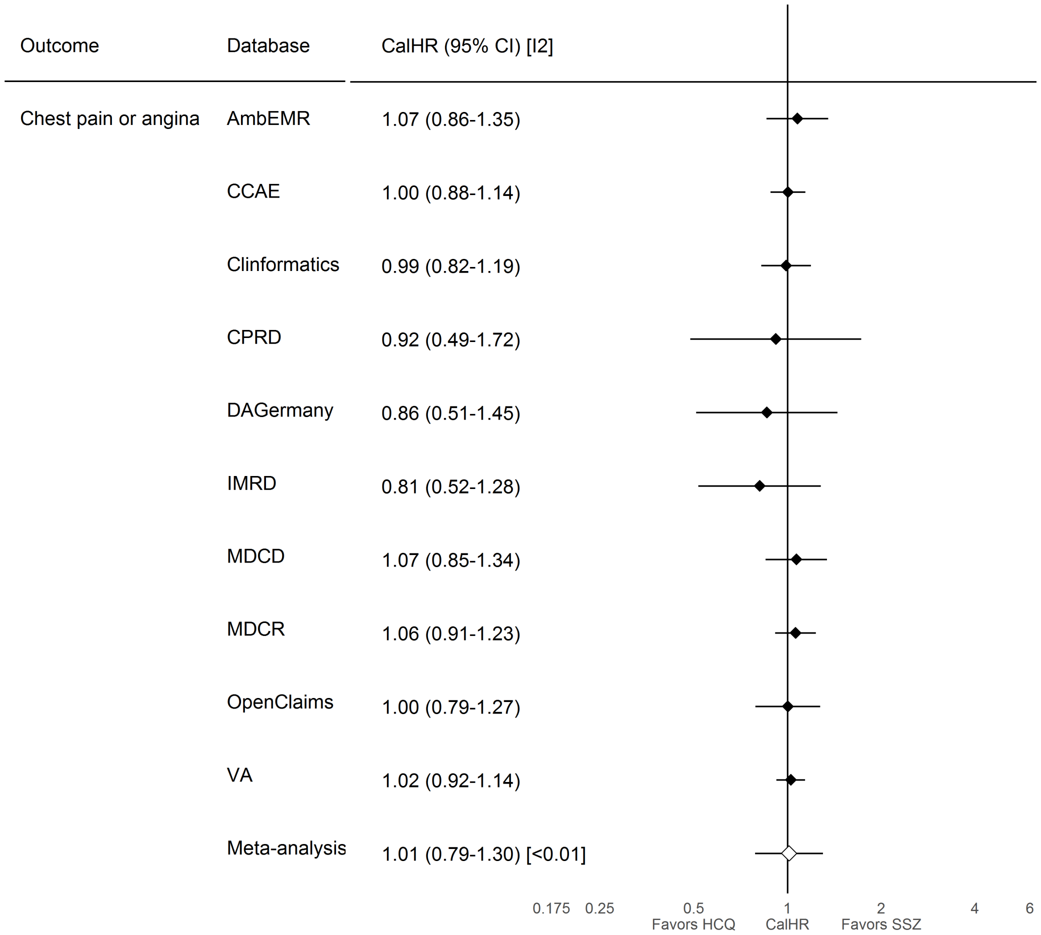

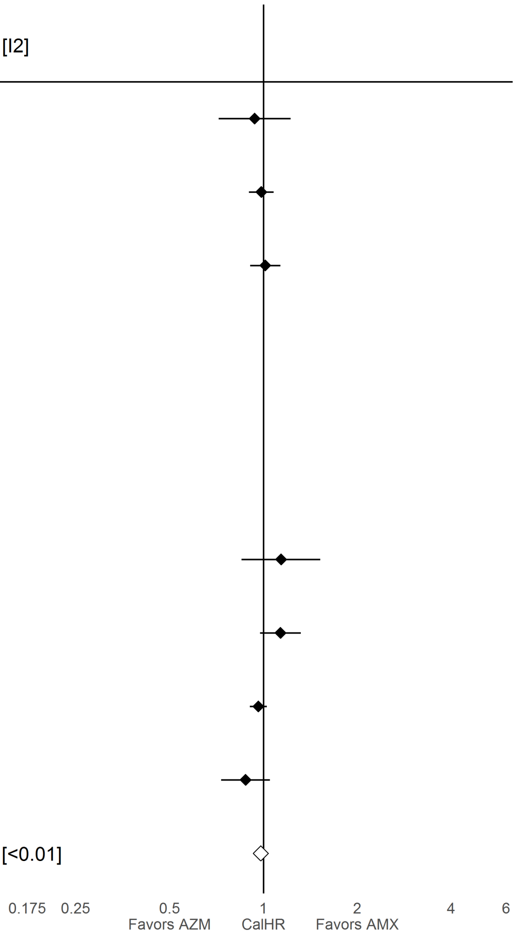

S8.2.5. Heart failure

Outcome	Database	HCQ vs SSZ CalHR (95% CI) [I2]	AZM vs AMX CalHR (95% CI) [I2]
Heart failure	AmbEMR	1.04 (0.74-1.45)	0.99 (0.66-1.50)
	CCAE	0.96 (0.72-1.28)	1.02 (0.79-1.31)
	Clinformatics	1.04 (0.83-1.30)	0.93 (0.77-1.11)
	CPRD	1.40 (0.57-3.43)	
	DAGermany	0.49 (0.22-1.09)	
	IMRD	1.30 (0.56-3.02)	
	MDCD	0.85 (0.55-1.31)	0.96 (0.63-1.48)
	MDCR	0.94 (0.77-1.16)	1.12 (0.93-1.35)
	OpenClaims	1.03 (0.81-1.32)	0.98 (0.91-1.05)
	VA	1.04 (0.90-1.20)	1.13 (0.91-1.40)
	Meta-analysis	1.04 (0.80-1.33) [<0.01]	0.99 (0.94-1.05) [<0.01]

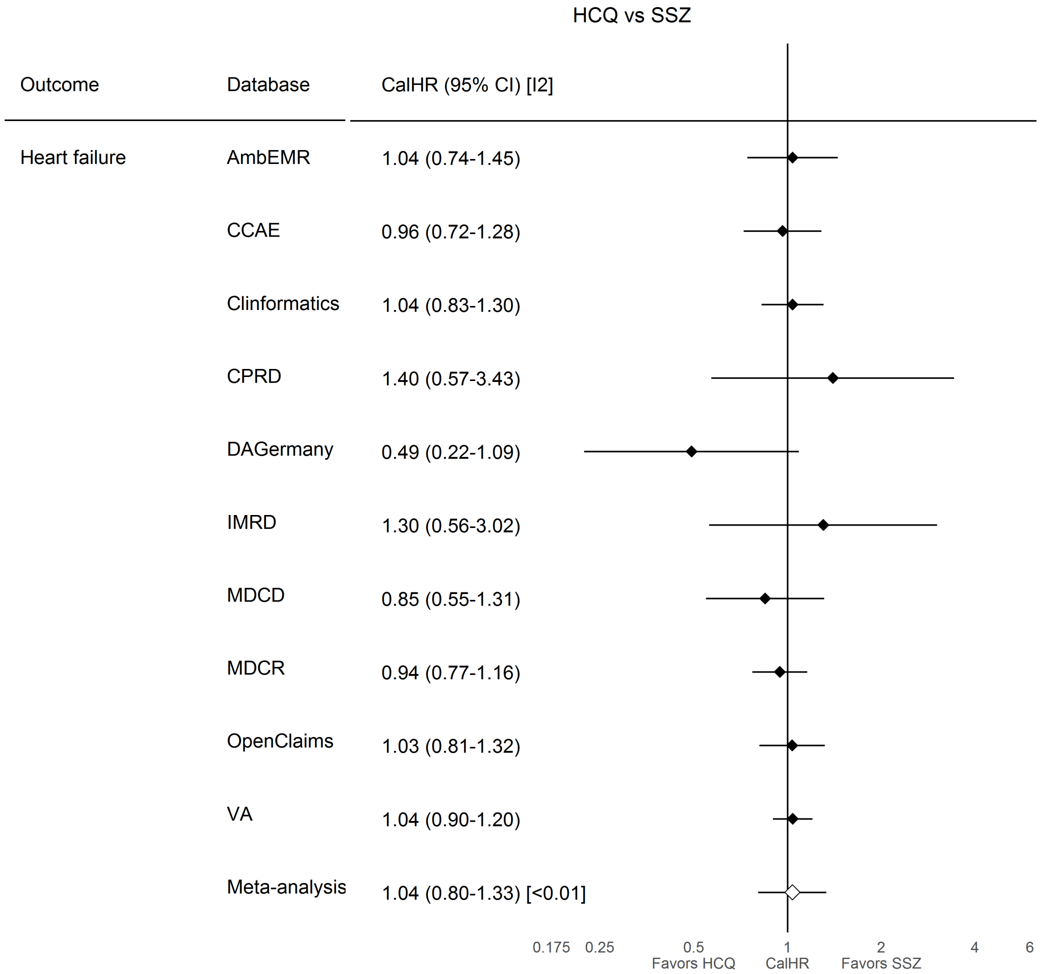

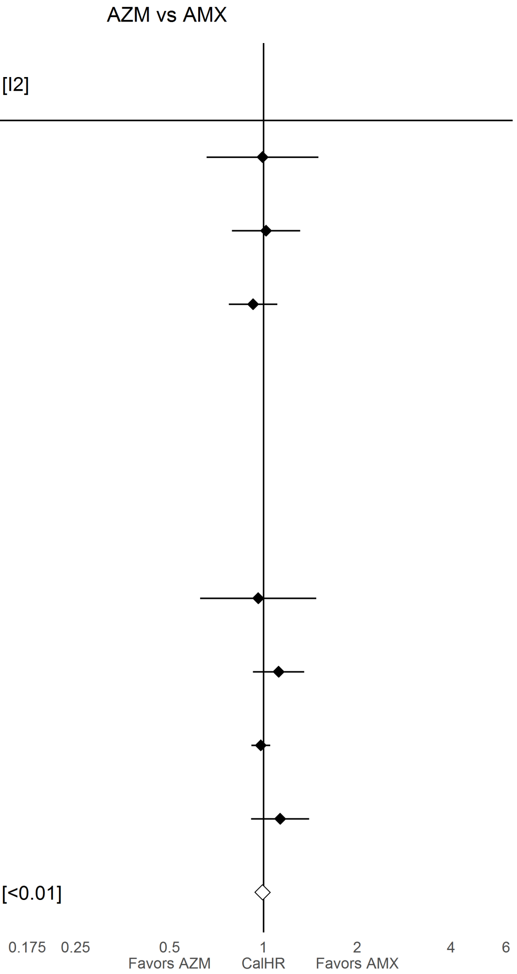

S8.2.6. Cardiovascular events

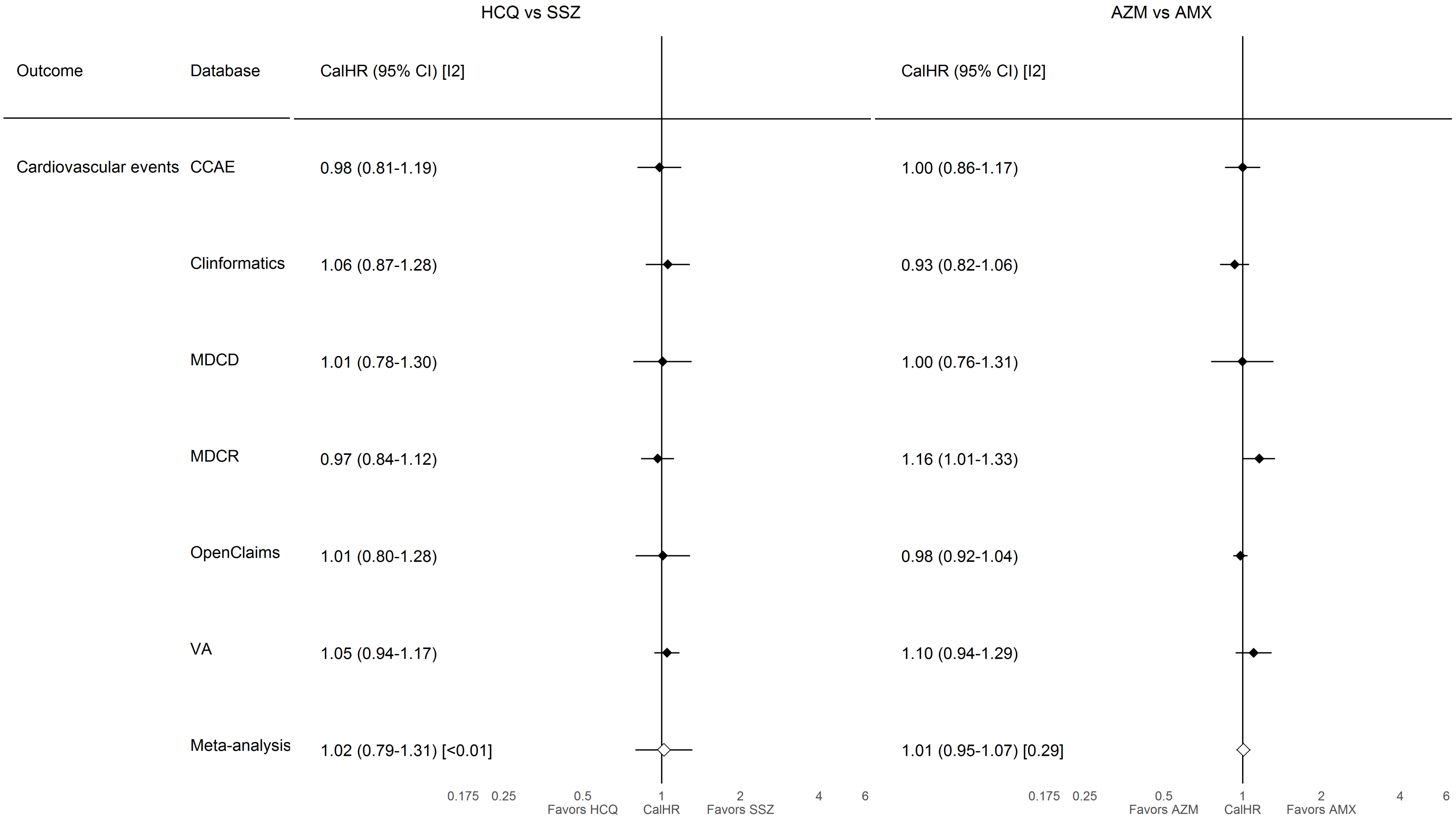

S8.2.7. Cardiac arrhythmia

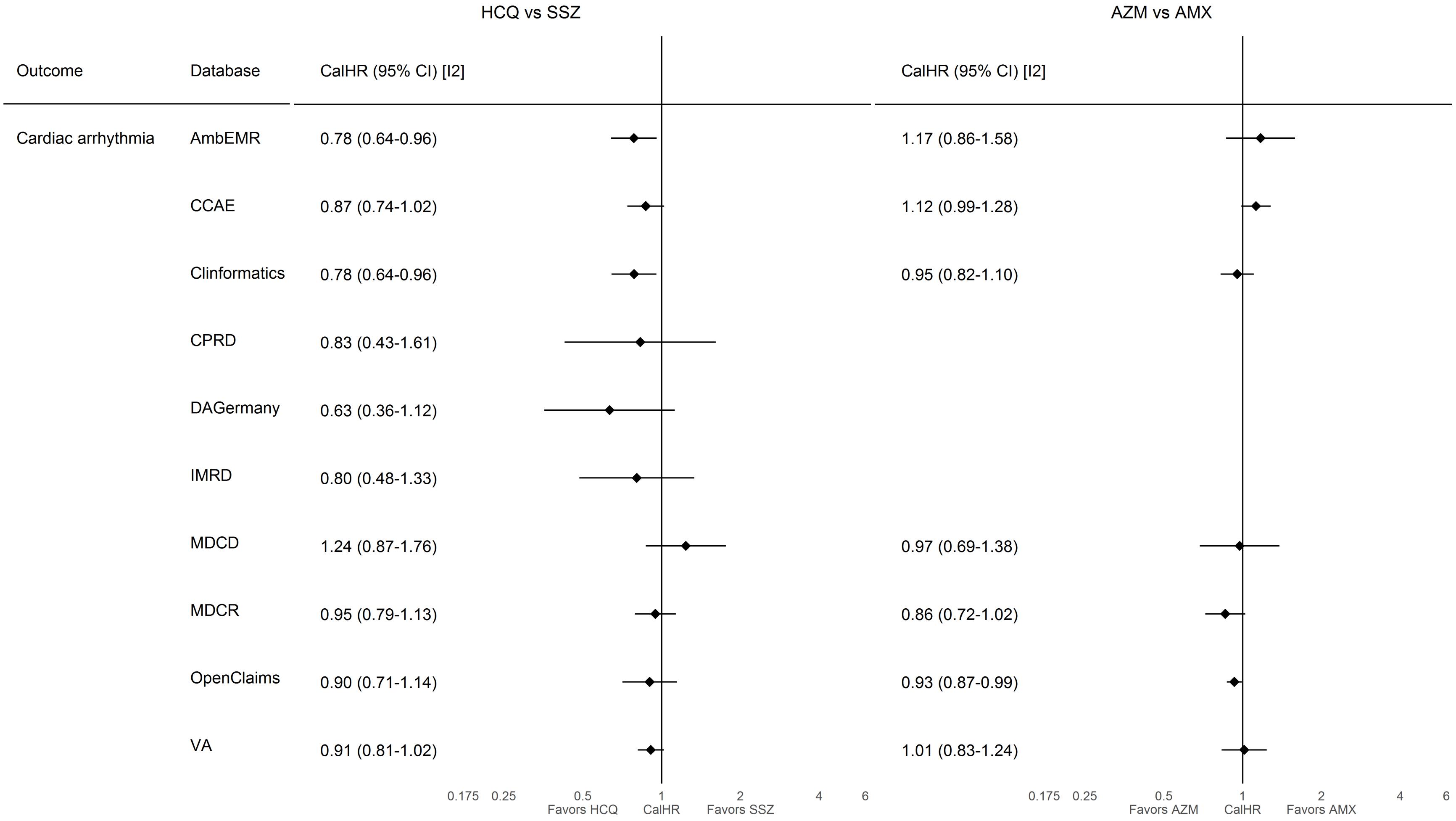

S8.2.8. Bradycardia

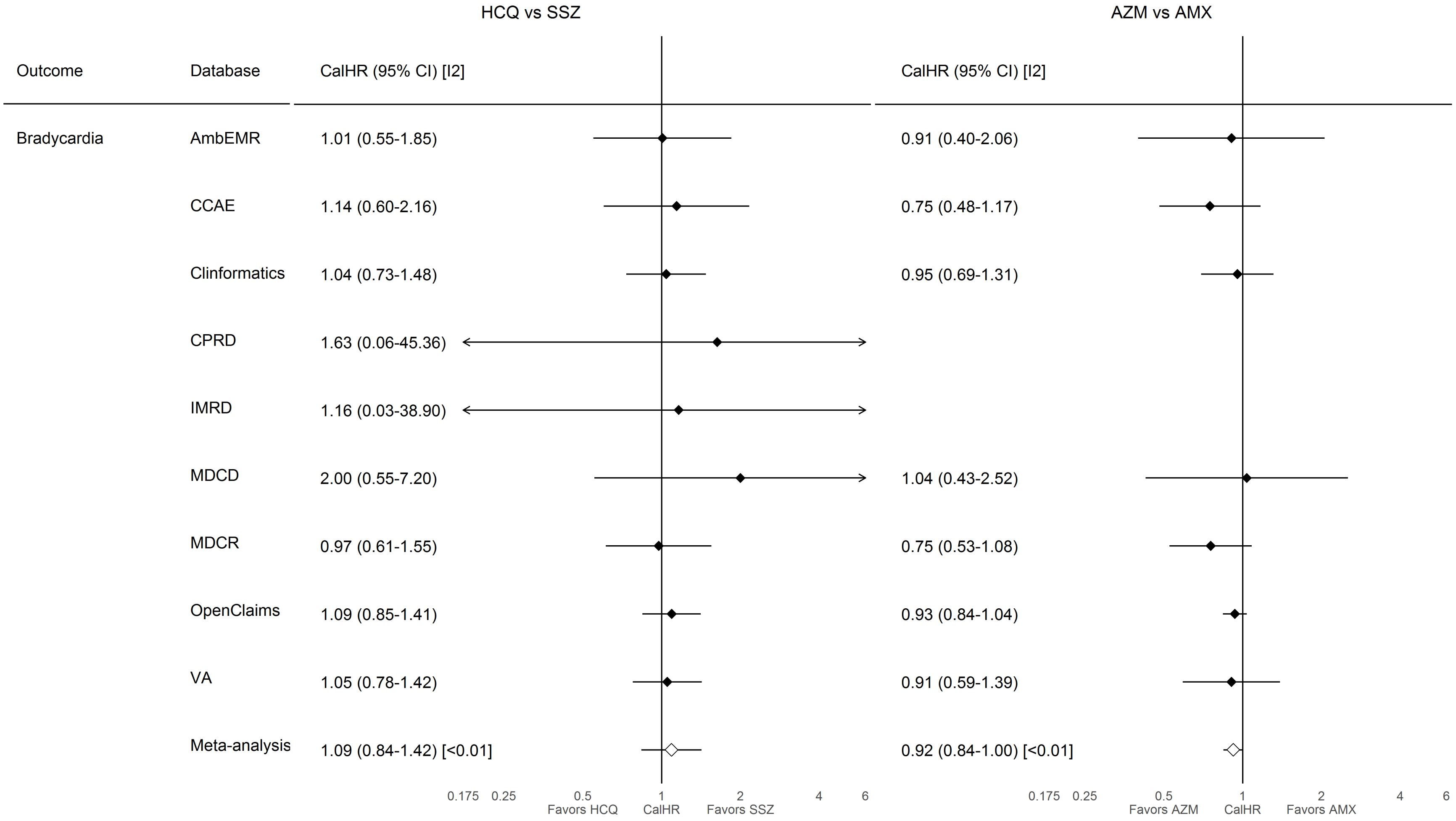

S8.2.9. Transient ischemic attack

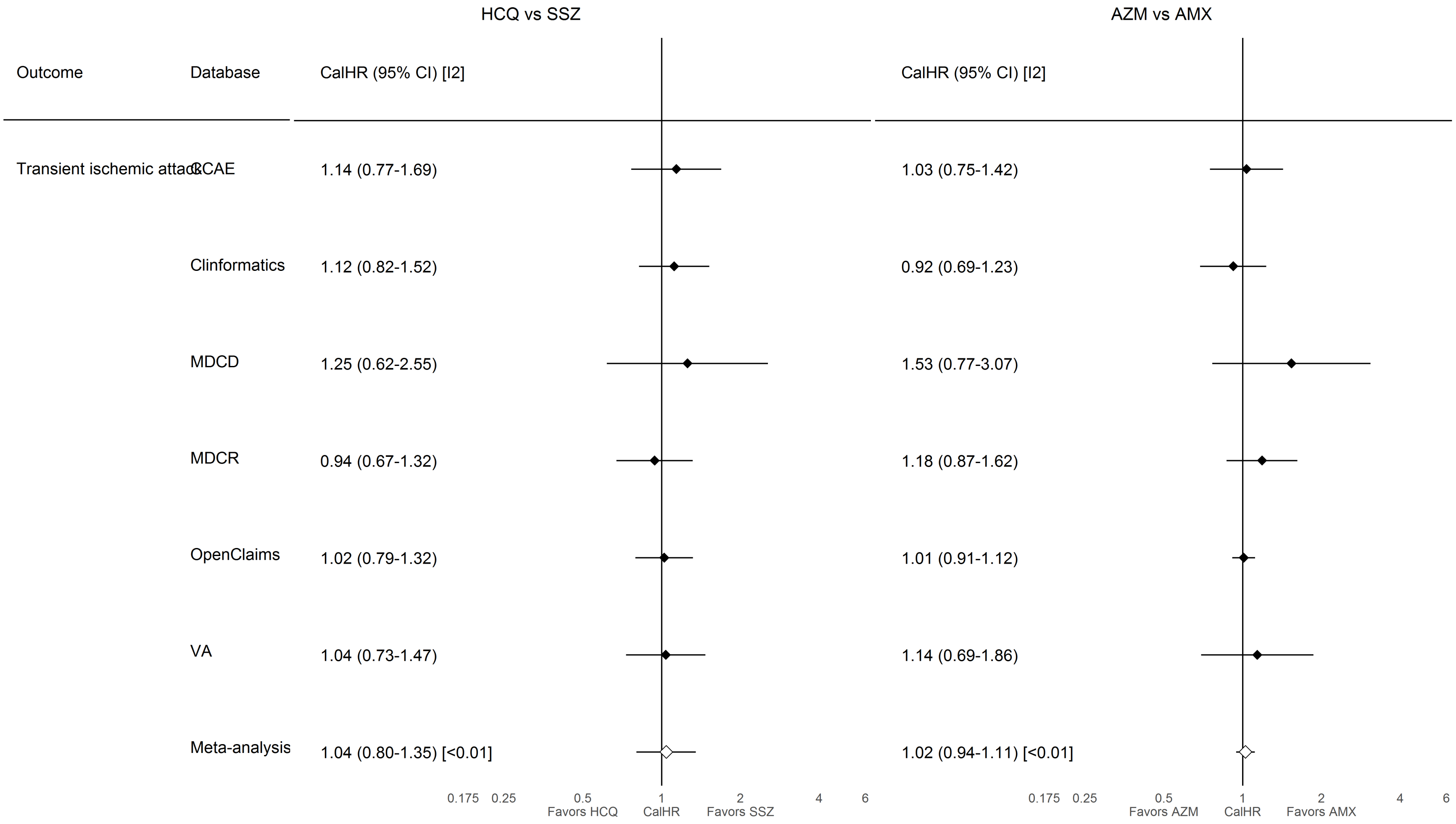

S8.2.10. Stroke

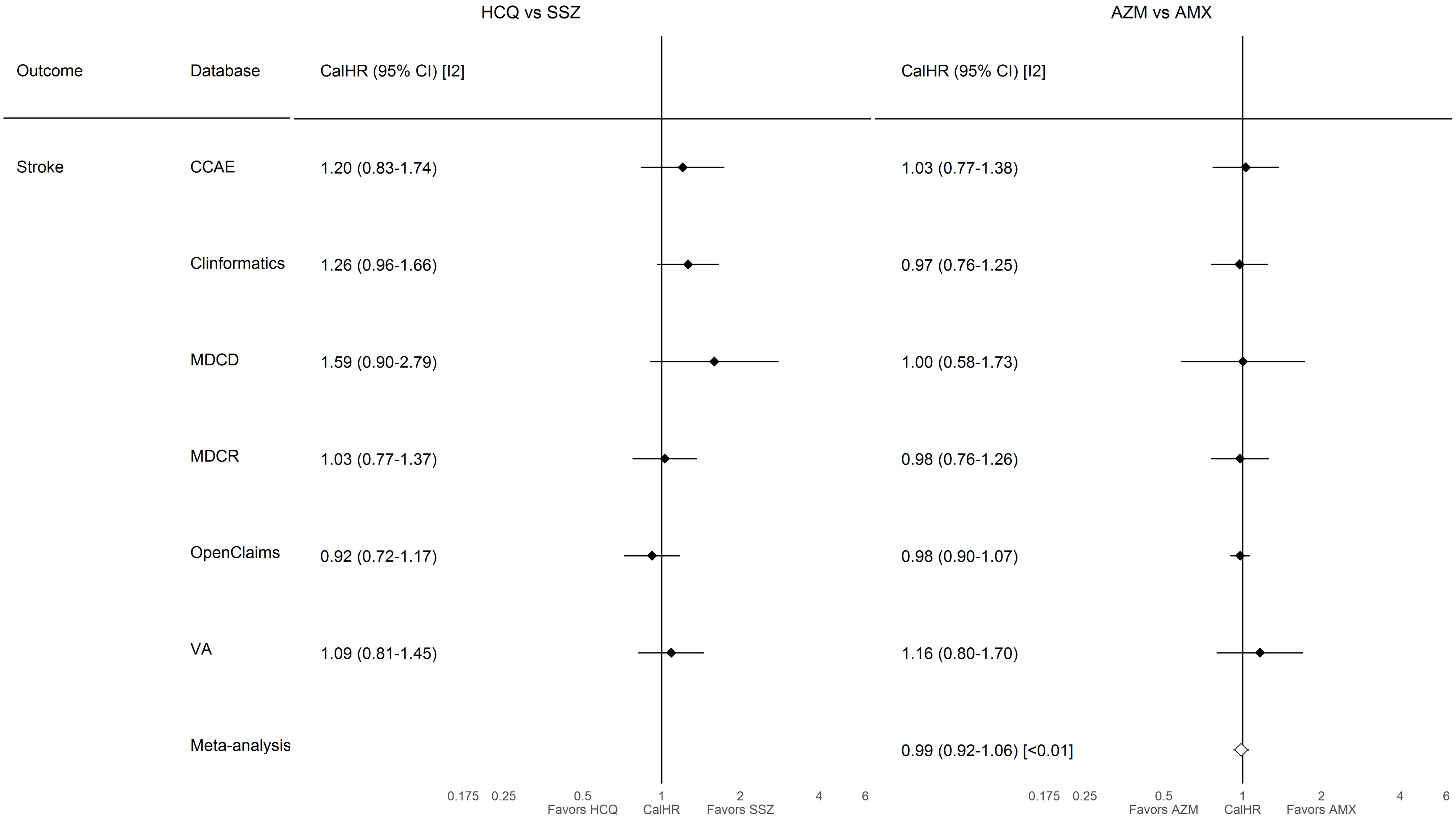

S8.2.11. Venous thromboembolism

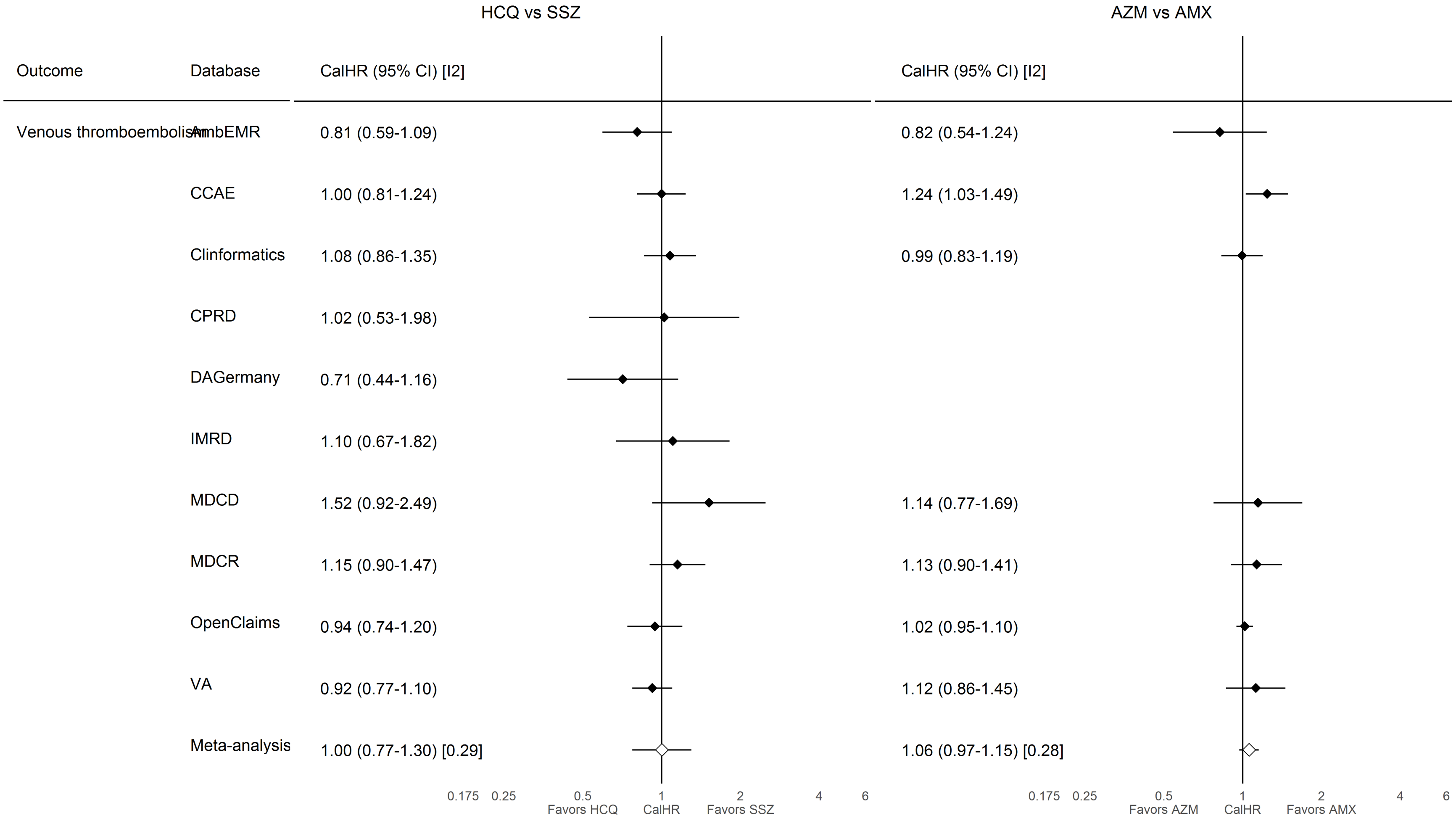

S8.2.12. Gastrointestinal bleeding

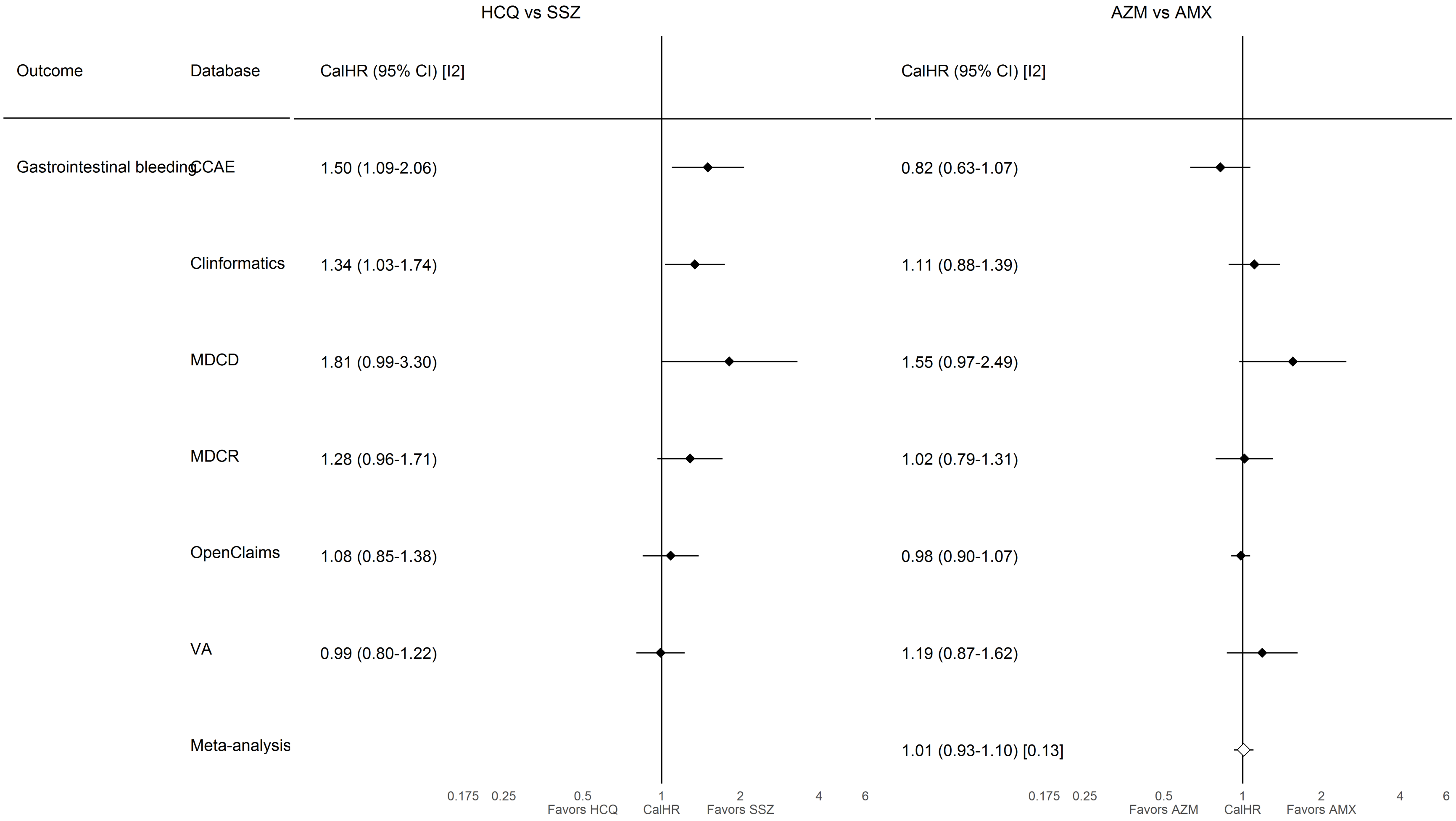

S8.2.13. Acute renal failure

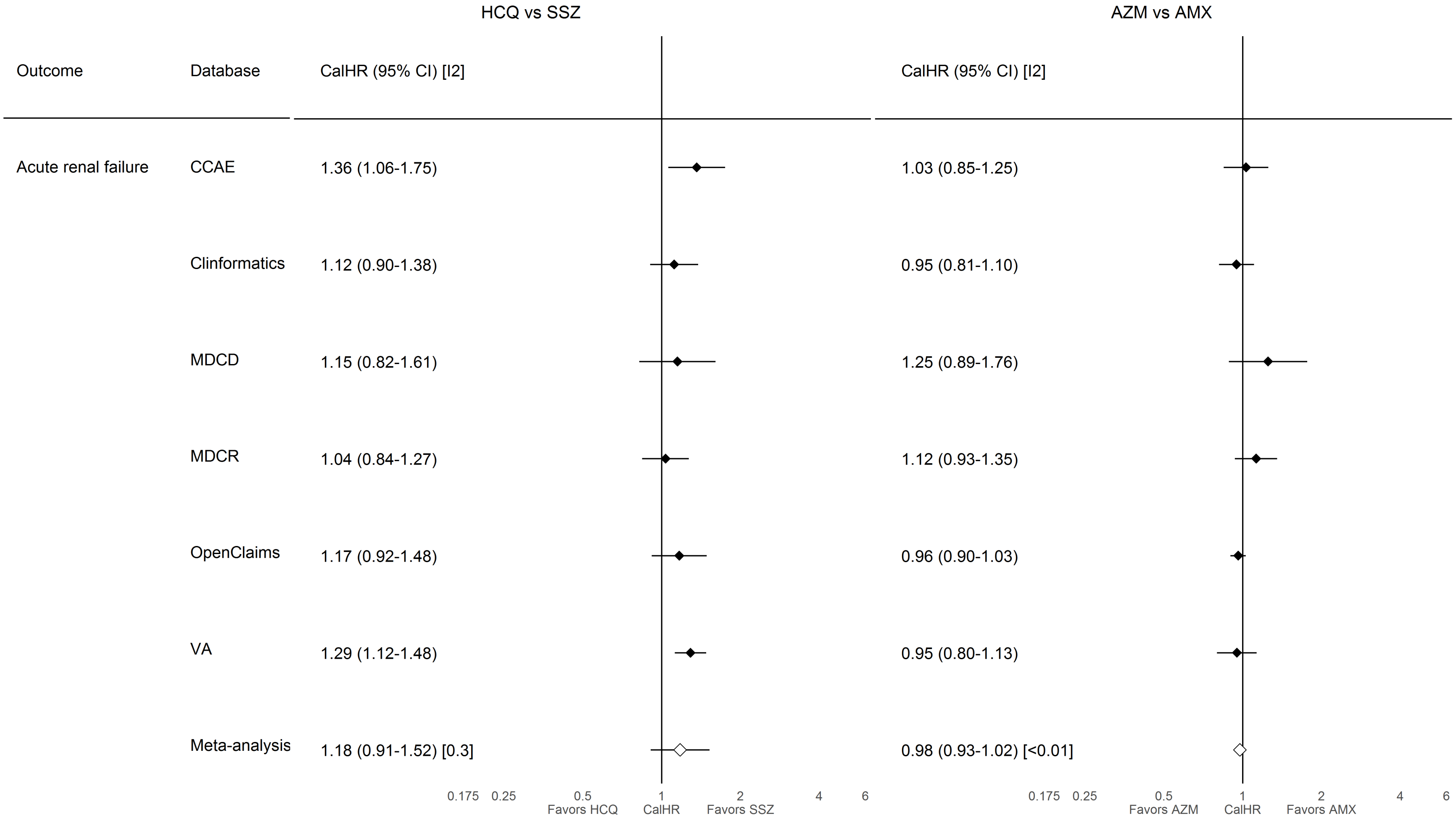

S8.2.14. End stage renal disease

Outcome	Database	HCQ vs SSZ CalHR (95% CI) [I2]	AZM vs AMX CalHR (95% CI) [I2]
End stage renal disease	AmbEMR	1.90 (0.18-20.50)	1.45 (0.43-4.93)
	CCAE	1.10 (0.49-2.44)	1.13 (0.61-2.09)
	Clinformatics	1.10 (0.65-1.88)	0.58 (0.33-1.01)
	MDCD	3.50 (0.36-33.94)	1.54 (0.65-3.64)
	MDCR	1.72 (0.76-3.88)	0.90 (0.47-1.70)
	OpenClaims	1.27 (0.95-1.69)	0.92 (0.80-1.06)
	VA	0.84 (0.53-1.34)	1.01 (0.58-1.77)
	Meta-analysis	1.23 (0.92-1.63) [<0.01]	0.93 (0.82-1.05) [<0.01]

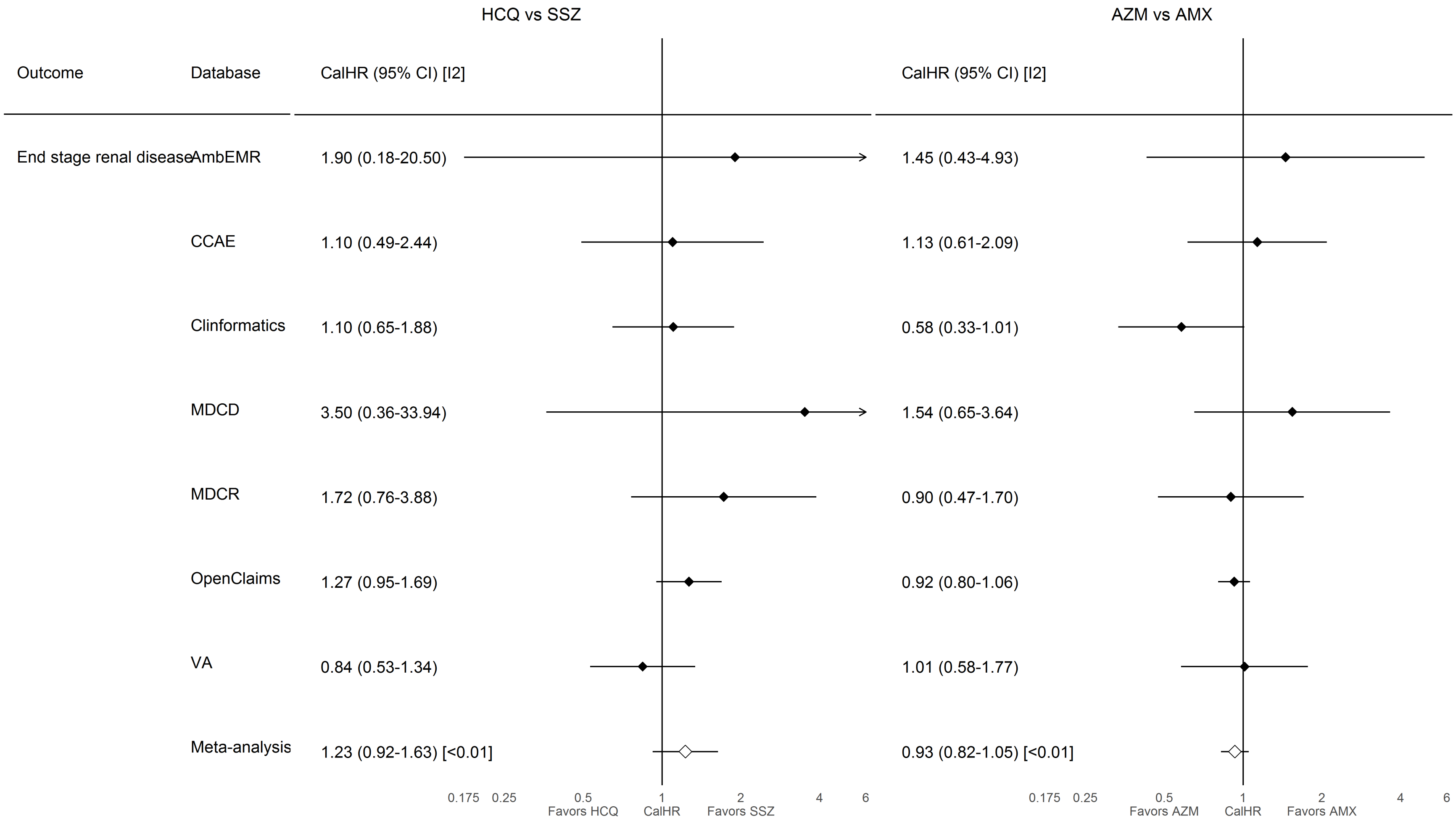

S8.2.15. Hepatic failure

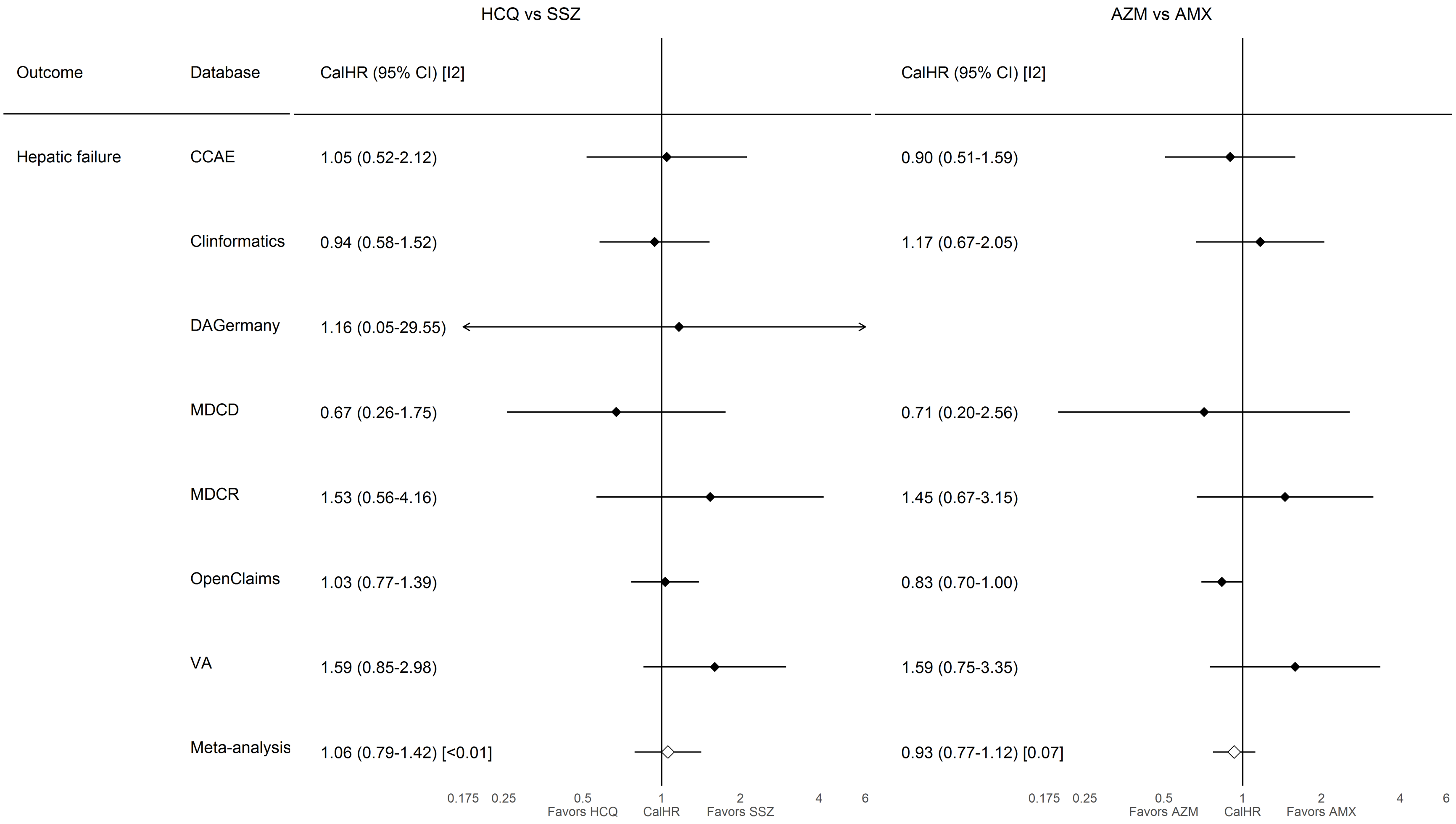

S8.2.16. Acute pancreatitis

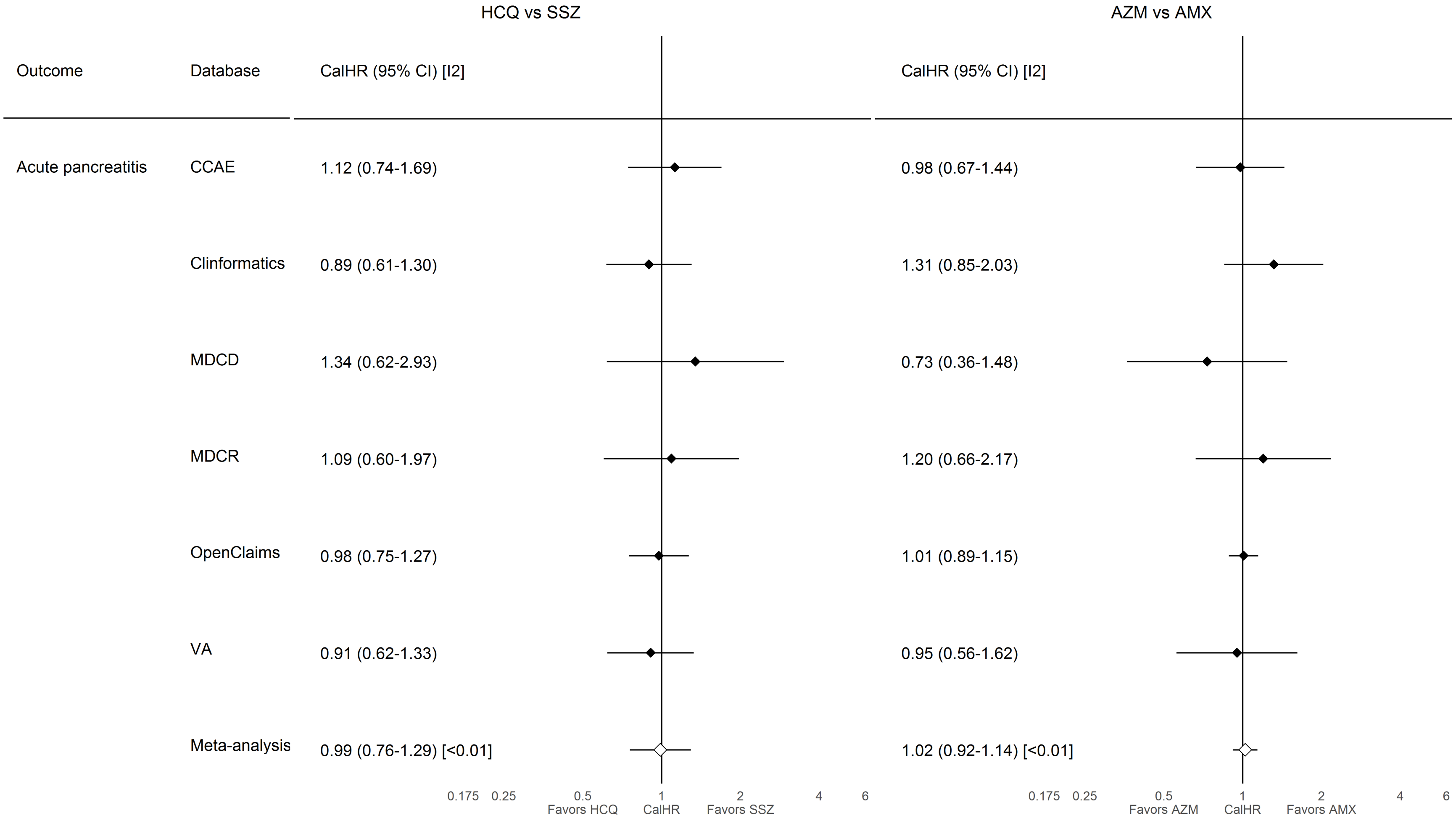

S9. Evidence evaluation diagnostics

Preference score overlap, covariate balance, and empirical calibration plots are reported below. The preference score is a transformation of the propensity score that adjusts for differences in the sizes of the two exposure cohorts in a pairwise comparison. A higher overlap indicates patients in the two cohorts were more similar in terms of their predicted probability of receiving one exposure relative to the other. In the covariate balance plots, each dot represents the standardizes difference of means for a single covariate before and after propensity score stratification. The empirical calibration plots show effect estimates for the negative controls where the true hazard ratio is expected to equal 1. Estimates below the diagonal dashed lines are statistically significantly different (alpha = 0.05) from the true effect size. A well-calibrated estimator should produce the true effect sizes within the 95 percent confidence interval 95 percent of the time. Negative control estimates are not reported if fewer than 5 events are observed. *On-treatment* time-at-risk was unavailable in the PanTher databases so no negative control effects were estimated. In DAGermany and JMDC, the hydroxychloroquine+azithromycin and hydroxychloroquine+amoxicillin cohorts were of insufficient sample size for the cross-validation procedure for hyperparameter selection in the regularized regression used for fitting propensity scores, so diagnostics were not reported.

S9.1. AmbEMR

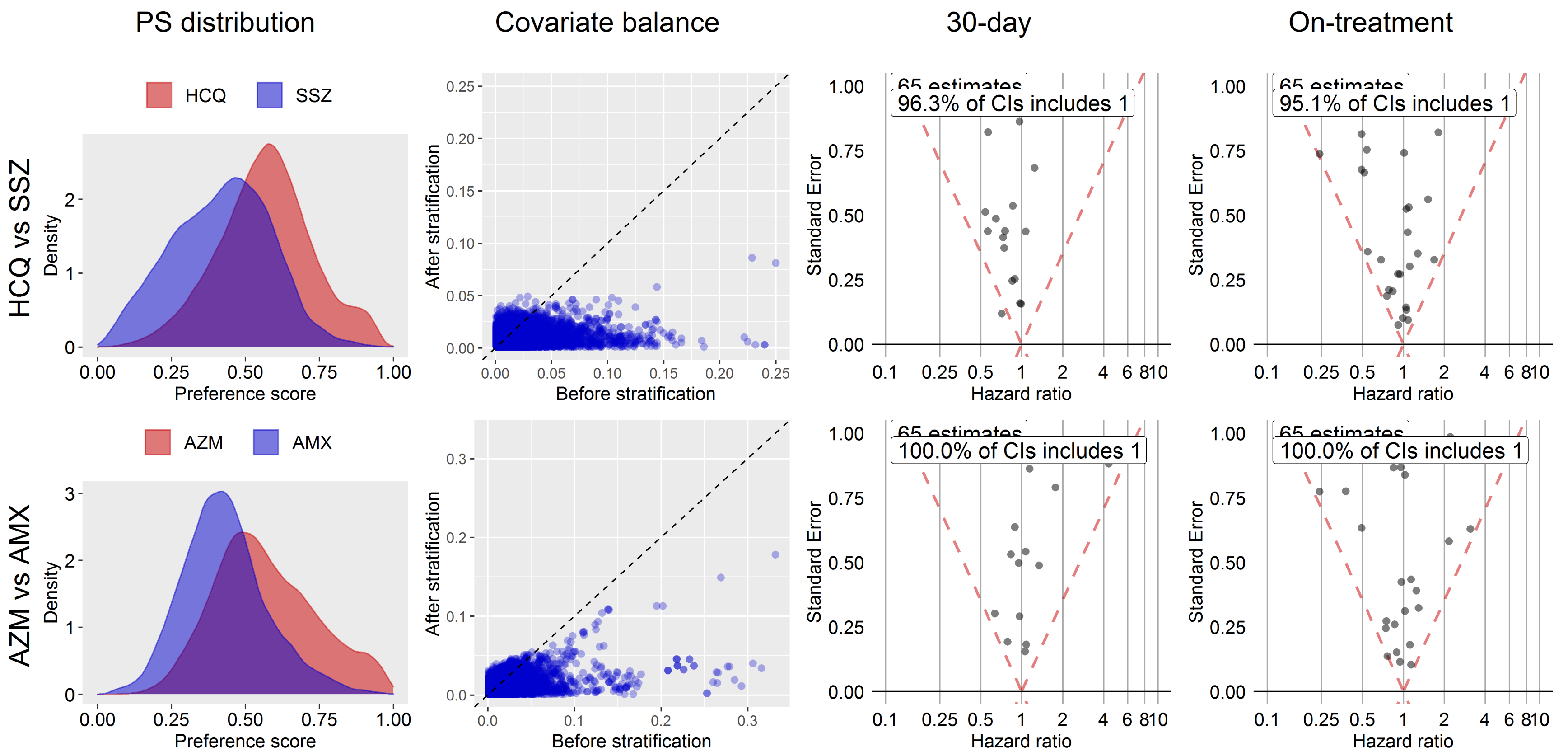
PS distribution
Covariate balance
30-day
On-treatment
HCQ vs SSZ
HCQ
SSZ
Density
Preference score
After stratification
Before stratification
65 estimates
96.3% of CIs includes 1
95.1% of CIs includes 1
Standard Error
Hazard ratio
AZM vs AMX
AZM
AMX
100.0% of CIs includes 1
100.0% of CIs includes 1

S9.2. CCAE

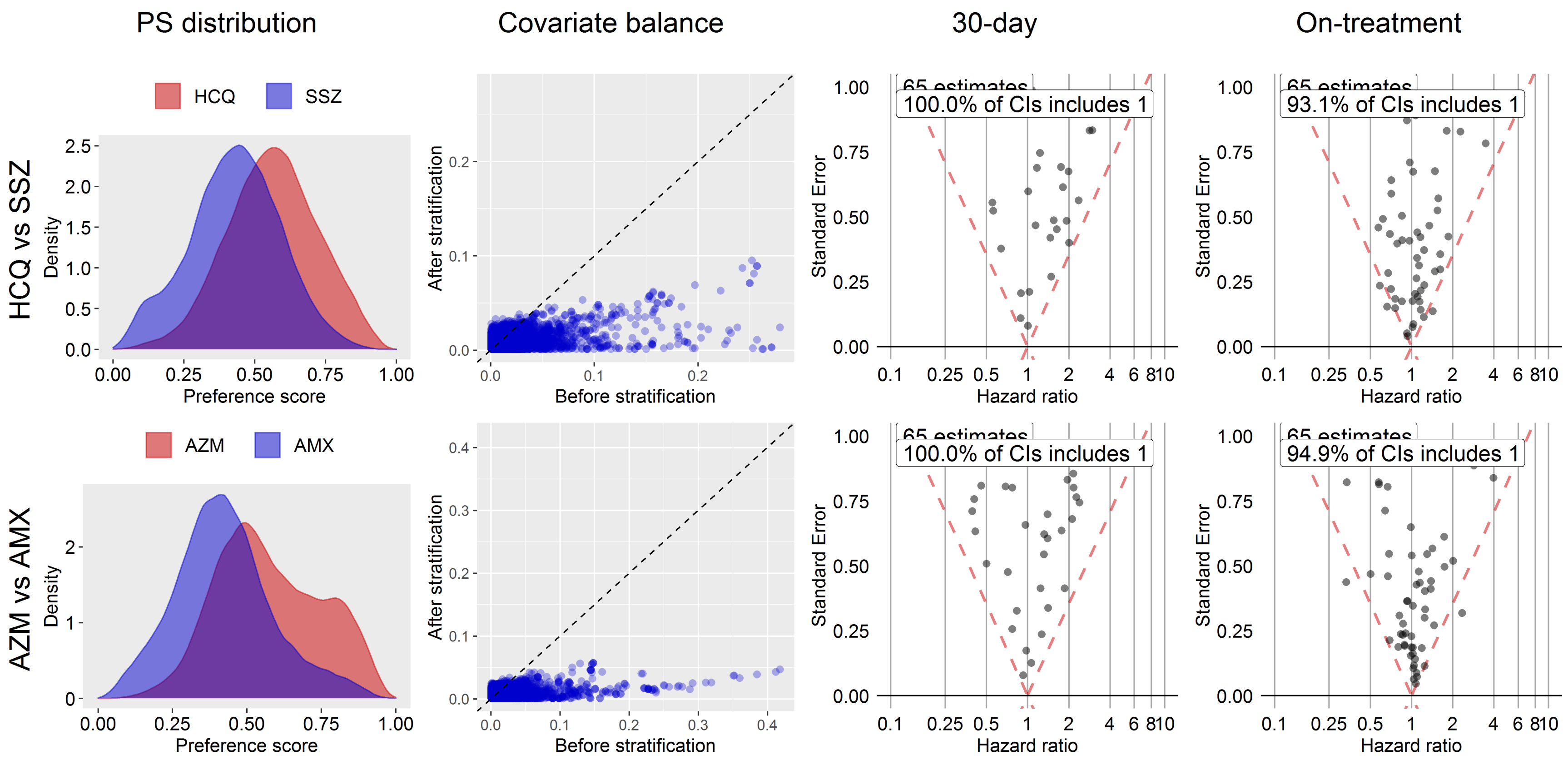
PS distribution
Covariate balance
30-day
On-treatment
HCQ vs SSZ
HCQ
SSZ
Density
Preference score
After stratification
Before stratification
Standard Error
Hazard ratio
65 estimates
100.0% of CIs includes 1
93.1% of CIs includes 1
AZM vs AMX
AZM
AMX
94.9% of CIs includes 1

S9.3. Clinformatics

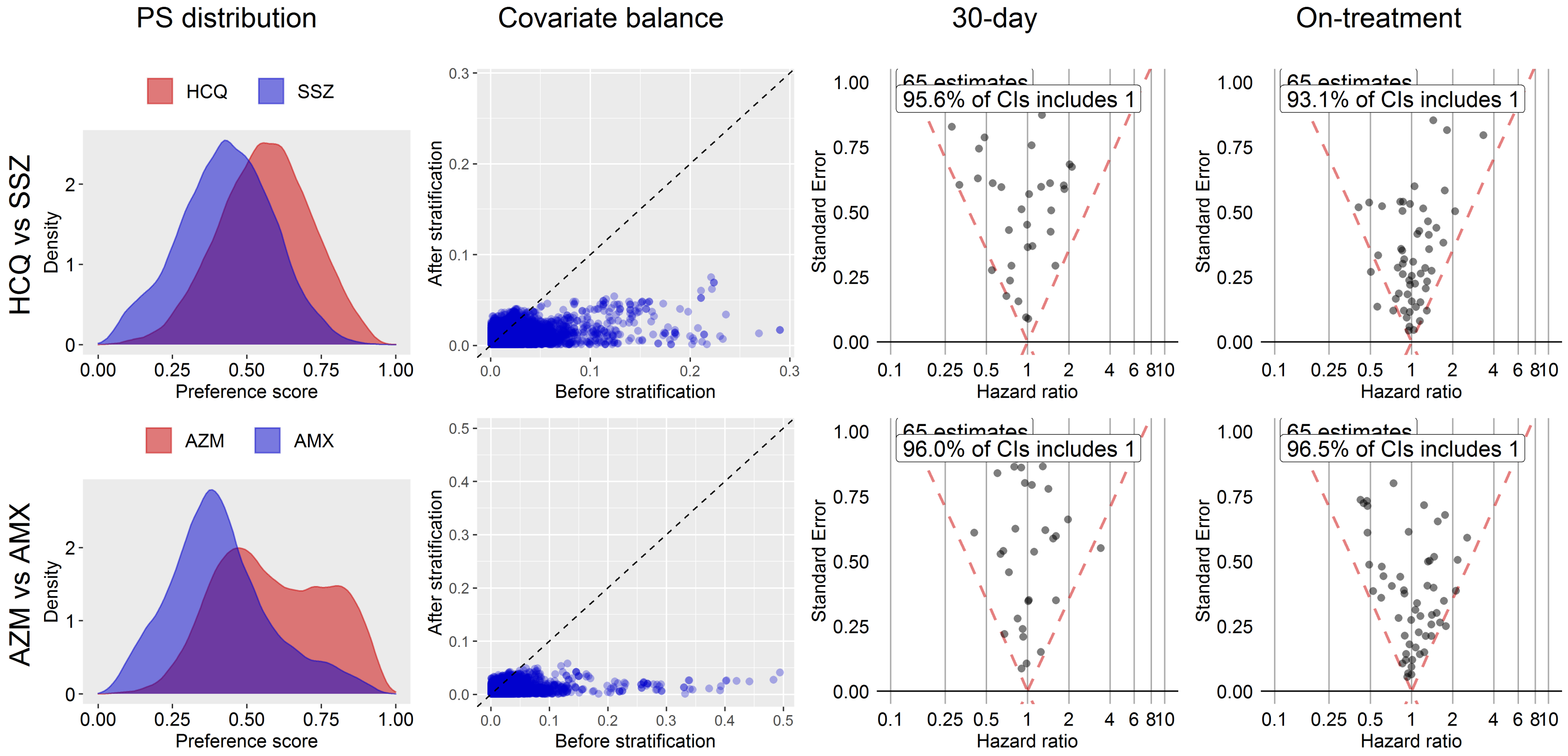

S9.4. CPRD

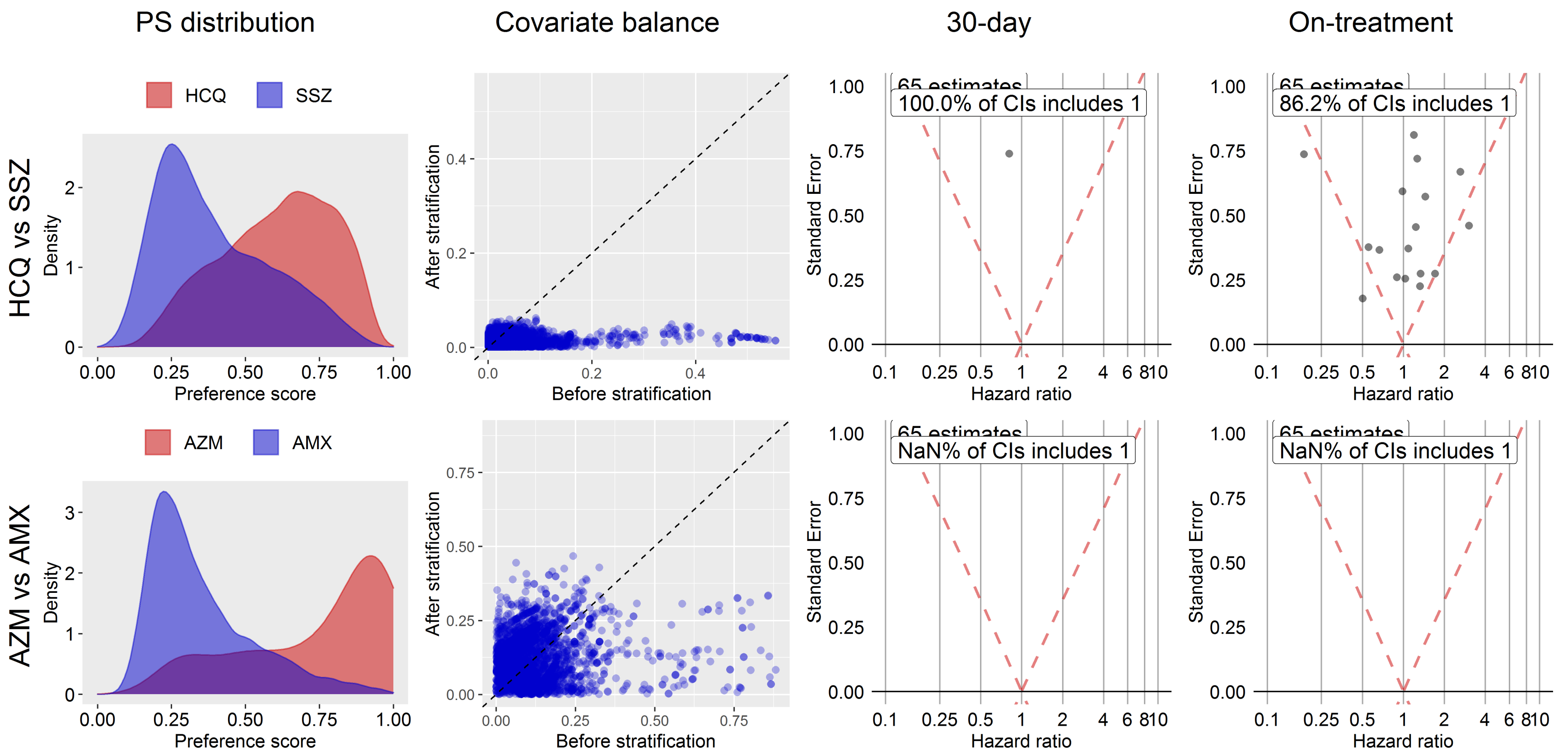

PS distribution
Covariate balance
30-day
On-treatment
HCQ vs SSZ
HCQ
SSZ
Density
Preference score
After stratification
Before stratification
Standard Error
Hazard ratio
65 estimates
100.0% of CIs includes 1
86.2% of CIs includes 1
AZM vs AMX
AZM
AMX
NaN% of CIs includes 1

S9.5. DAGermany

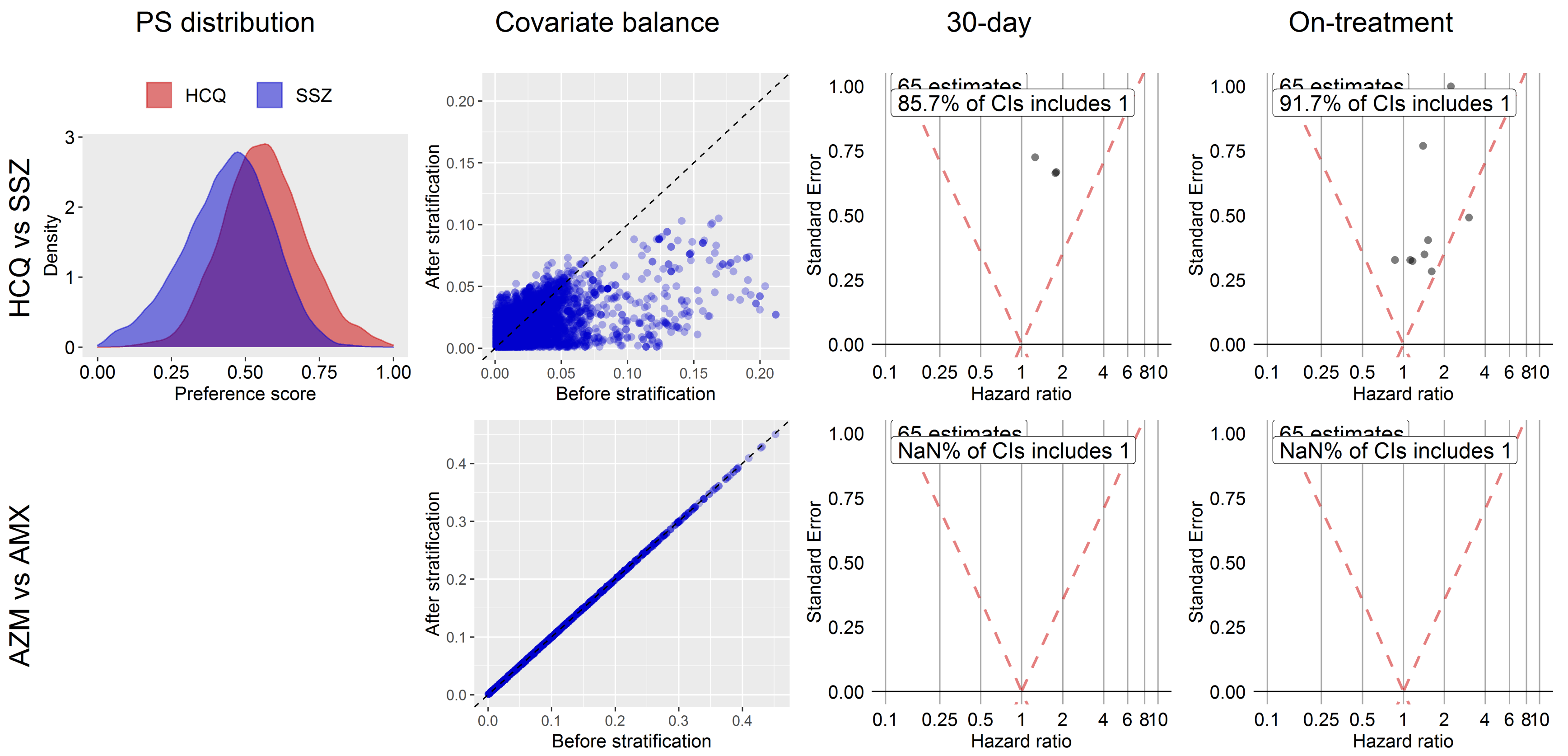

S9.6. IMRD

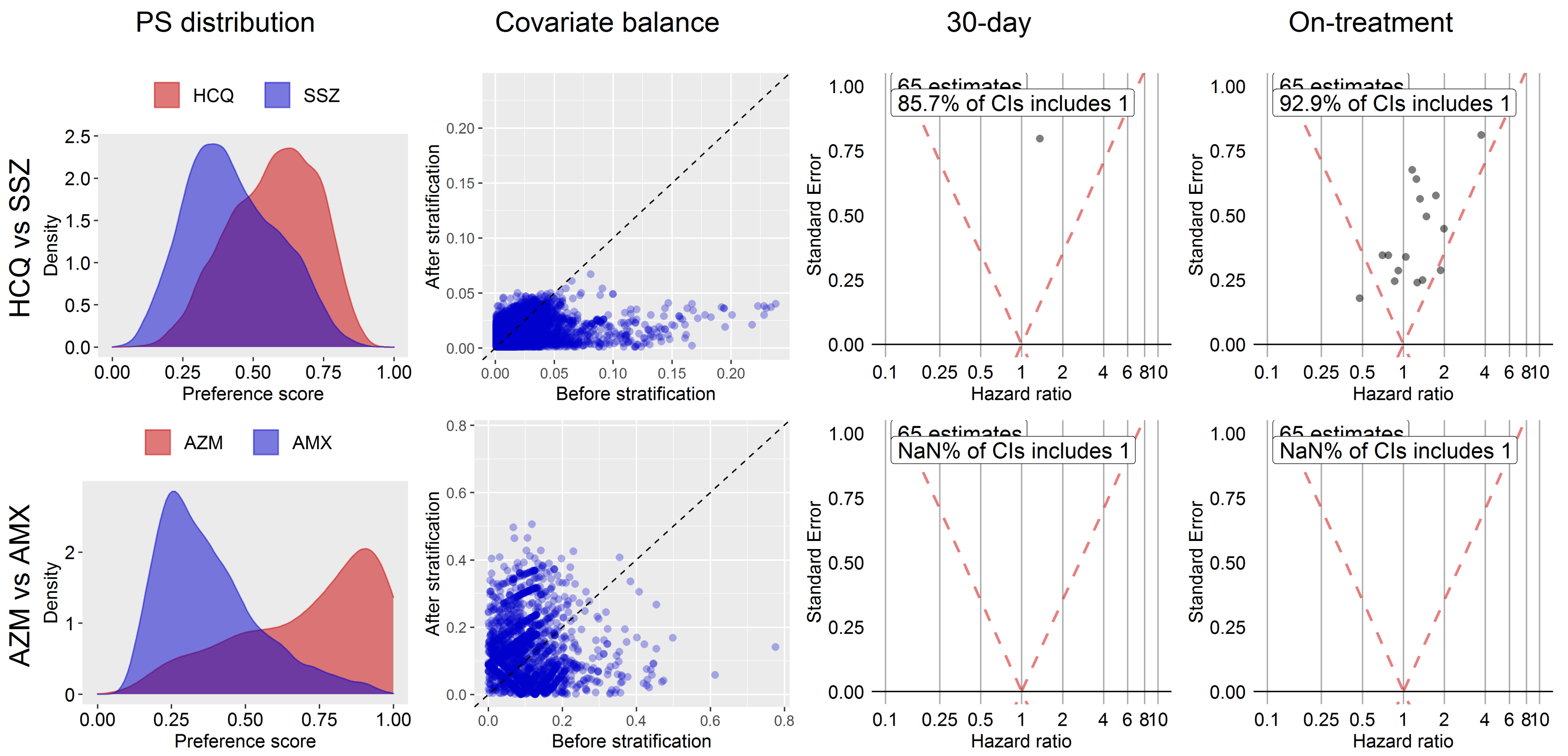
PS distribution
Covariate balance
30-day
On-treatment
HCQ vs SSZ
HCQ
SSZ
Density
Preference score
After stratification
Before stratification
Standard Error
Hazard ratio
65 estimates
85.7% of CIs includes 1
92.9% of CIs includes 1
AZM vs AMX
AZM
AMX
NaN% of CIs includes 1

S9.7. IPCI

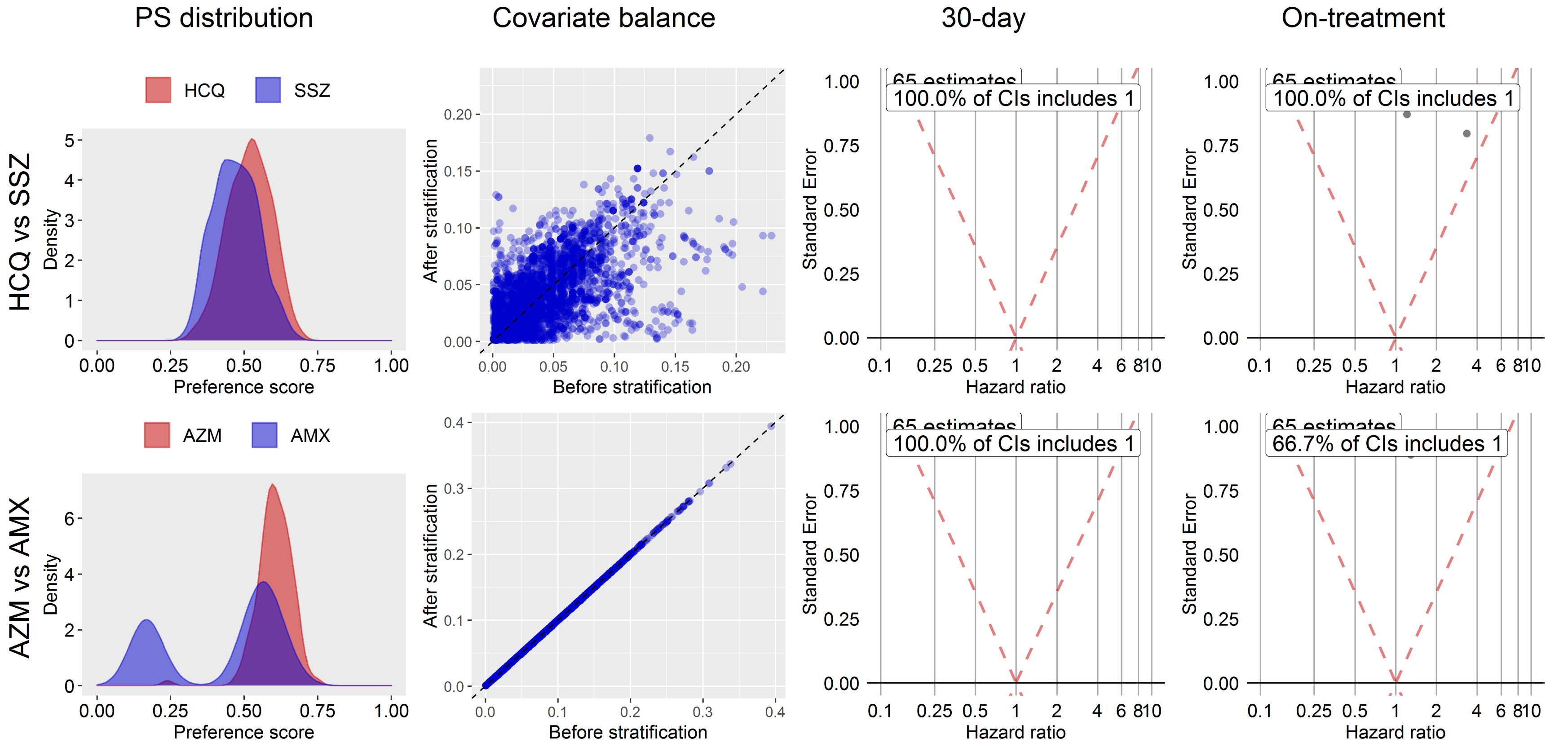
PS distribution
Covariate balance
30-day
On-treatment
HCQ vs SSZ
HCQ
SSZ
Density
Preference score
After stratification
Before stratification
65 estimates
100.0% of CIs includes 1
Standard Error
Hazard ratio
AZM vs AMX
AZM
AMX
65 estimates
100.0% of CIs includes 1
66.7% of CIs includes 1

S9.8. JMDC

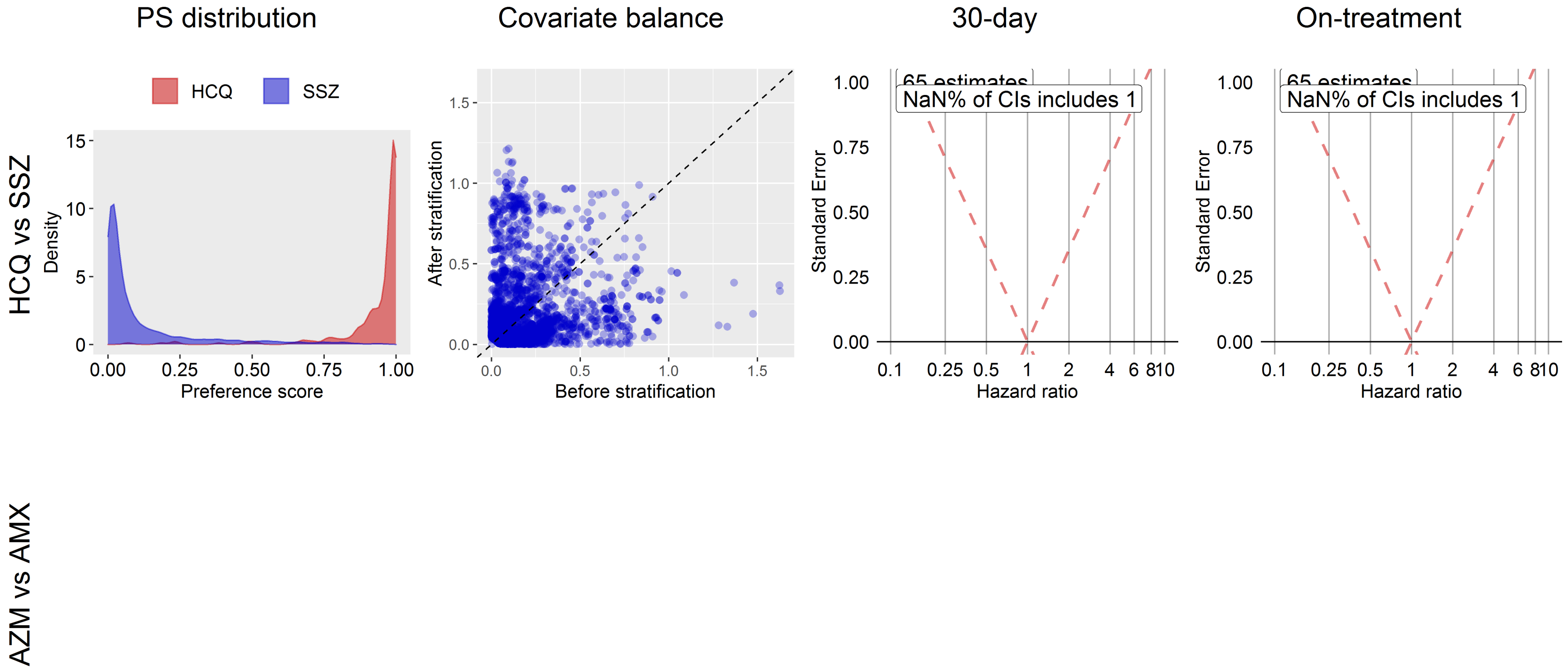
PS distribution
Covariate balance
30-day
On-treatment
HCQ vs SSZ
AZM vs AMX
HCQ
SSZ
Density
Preference score
After stratification
Before stratification
Standard Error
Hazard ratio
65 estimates
NaN% of CIs includes 1

S9.9. MDCD

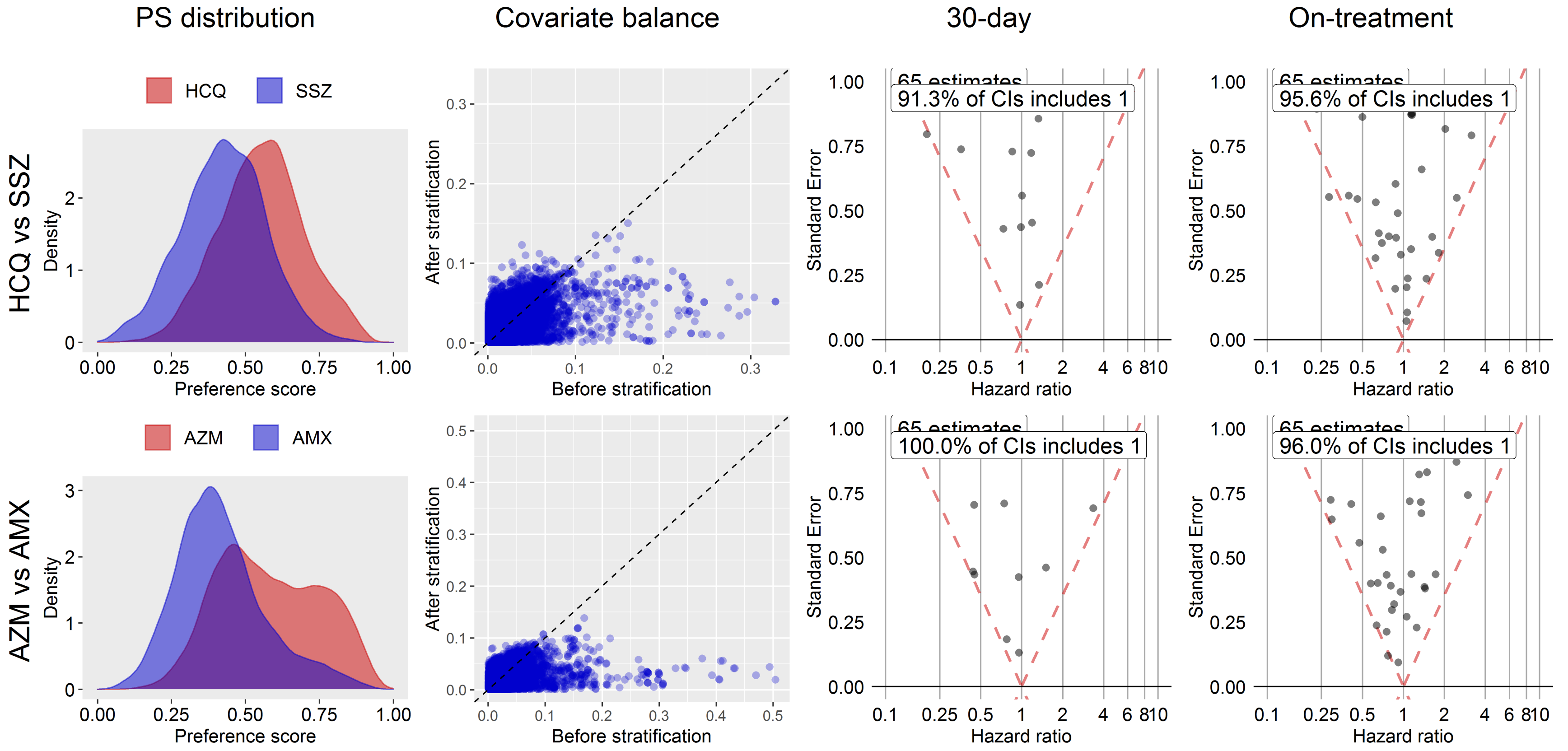
PS distribution
Covariate balance
30-day
On-treatment
HCQ vs SSZ
HCQ
SSZ
Density
Preference score
After stratification
Before stratification
65 estimates
91.3% of CIs includes 1
95.6% of CIs includes 1
Standard Error
Hazard ratio
AZM vs AMX
AZM
AMX
100.0% of CIs includes 1
96.0% of CIs includes 1

S9.10. MDCR

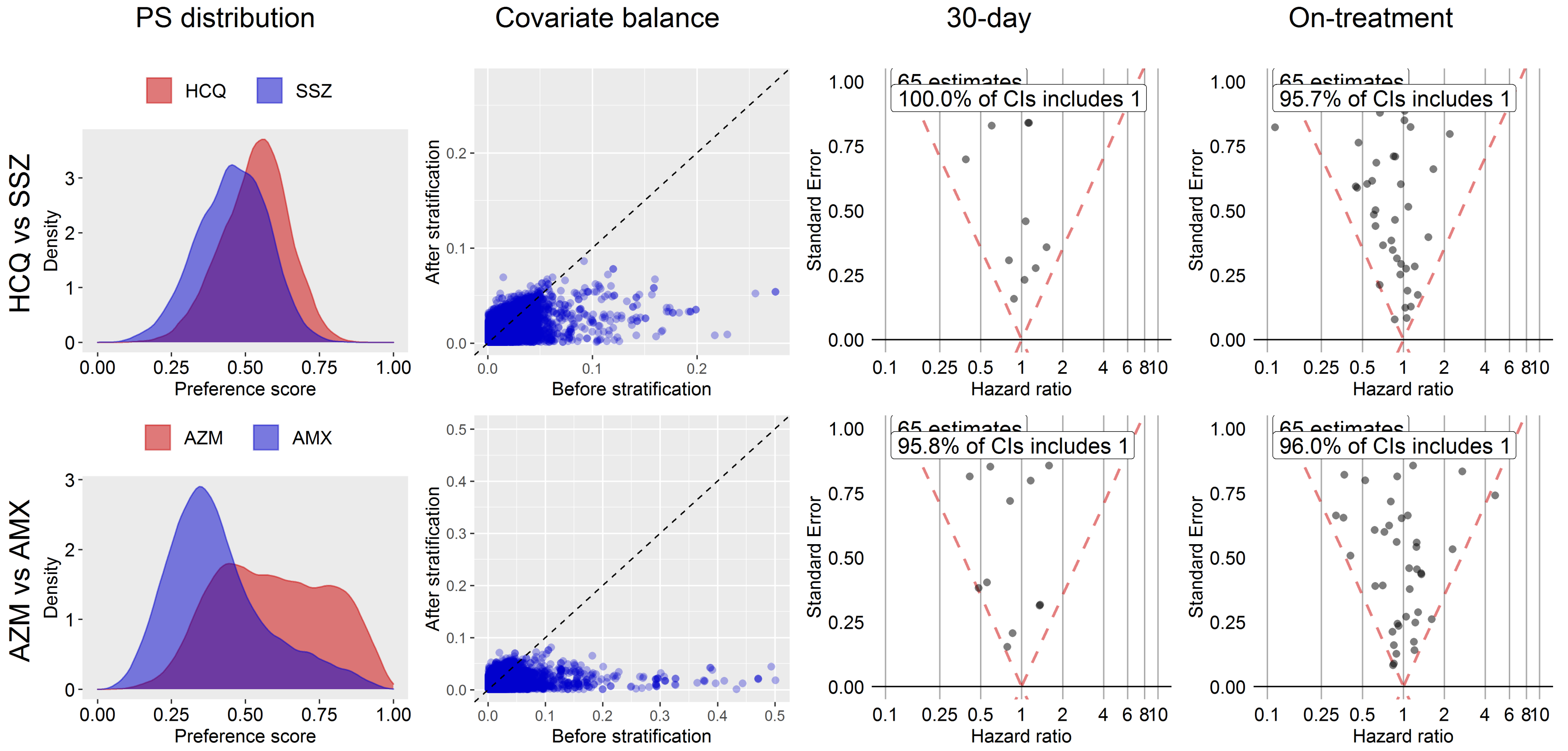
PS distribution
Covariate balance
30-day
On-treatment
HCQ vs SSZ
AZM vs AMX
HCQ
SSZ
AZM
AMX
Density
Preference score
After stratification
Before stratification
Standard Error
Hazard ratio
65 estimates
100.0% of CIs includes 1
95.7% of CIs includes 1
95.8% of CIs includes 1
96.0% of CIs includes 1

S9.11. OpenClaims

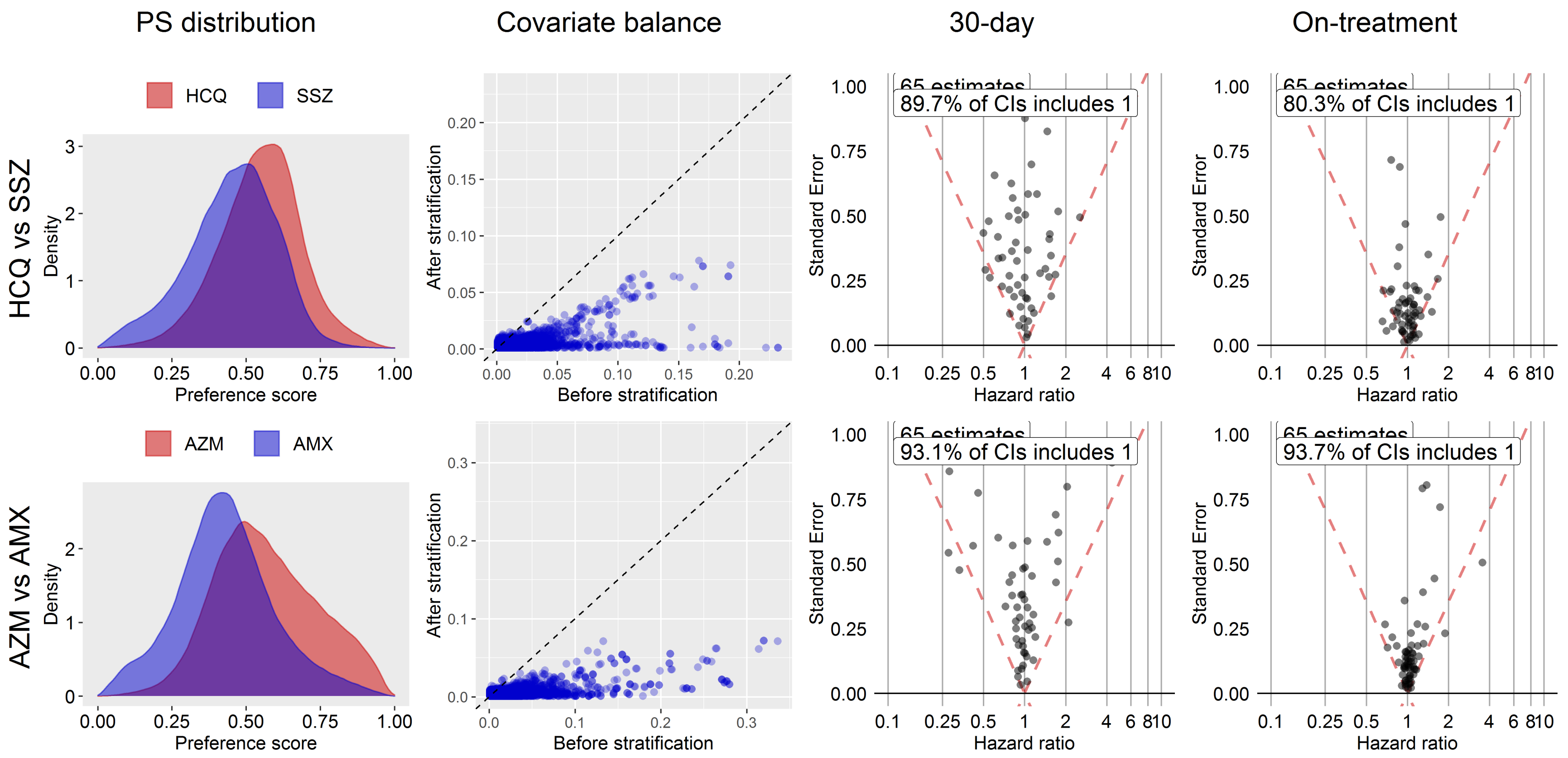

S9.12. OptumEHR

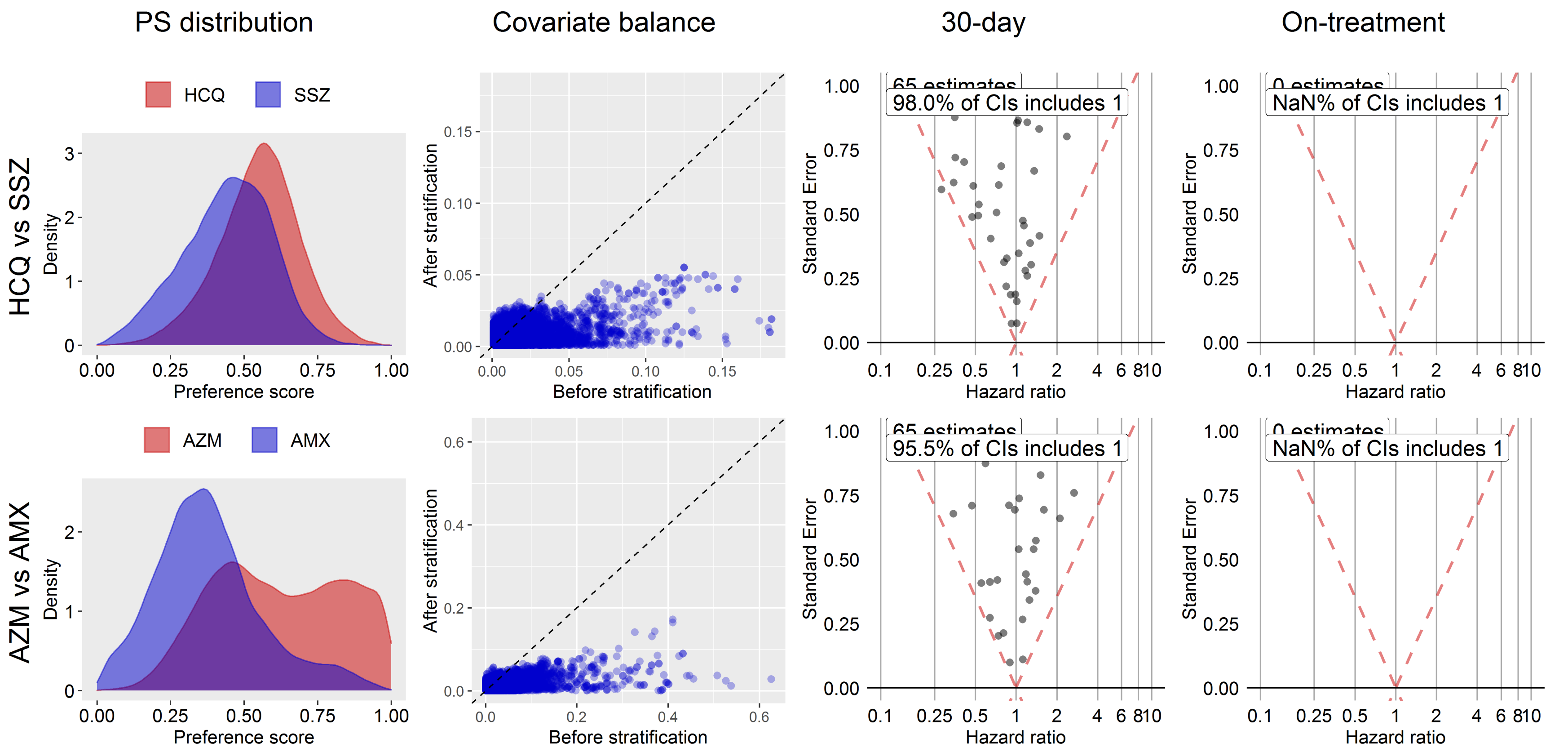

S9.13. SIDIAP

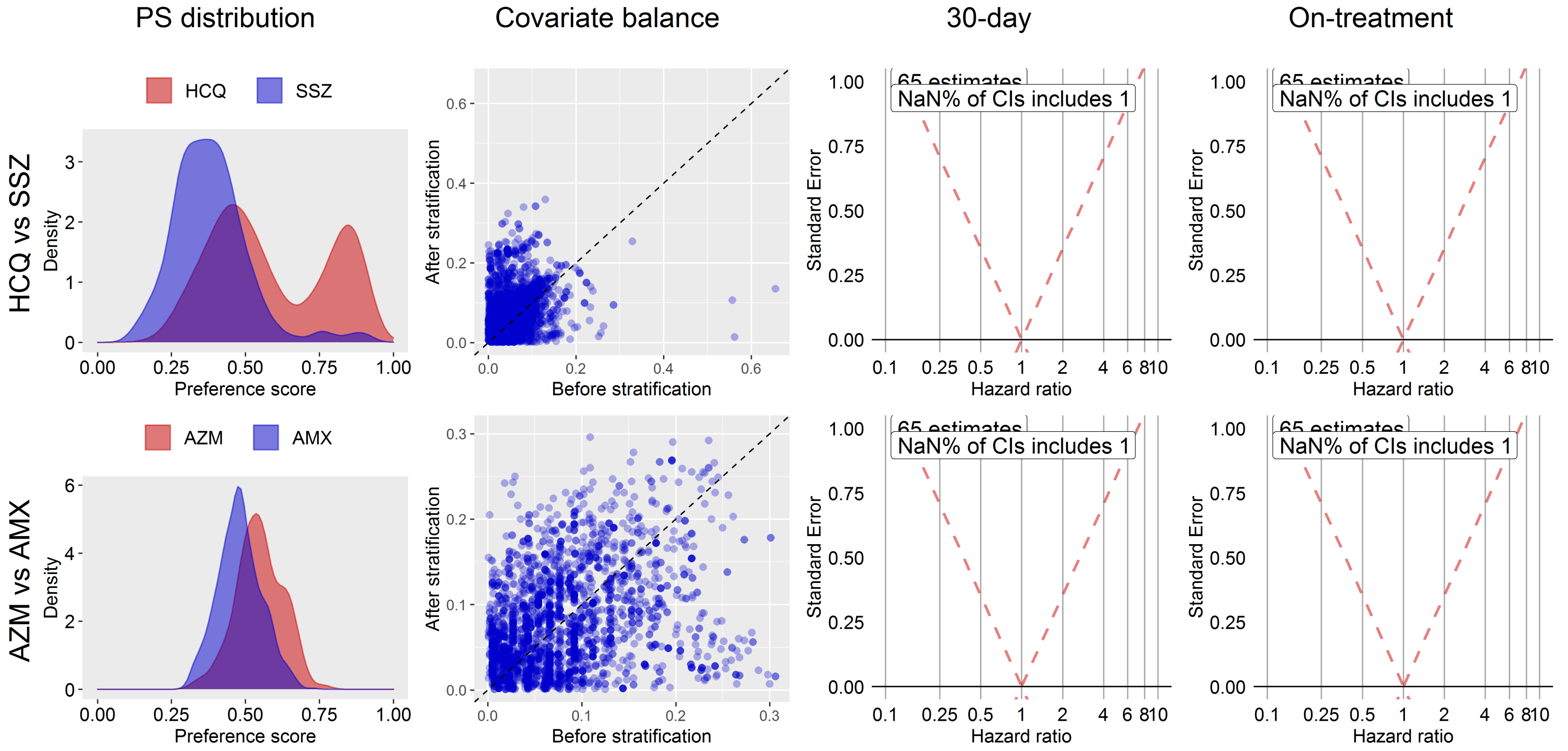
PS distribution
Covariate balance
30-day
On-treatment
HCQ vs SSZ
HCQ
SSZ
Density
Preference score
After stratification
Before stratification
65 estimates
NaN% of CIs includes 1
Standard Error
Hazard ratio
AZM vs AMX
AZM
AMX

S9.14. VA

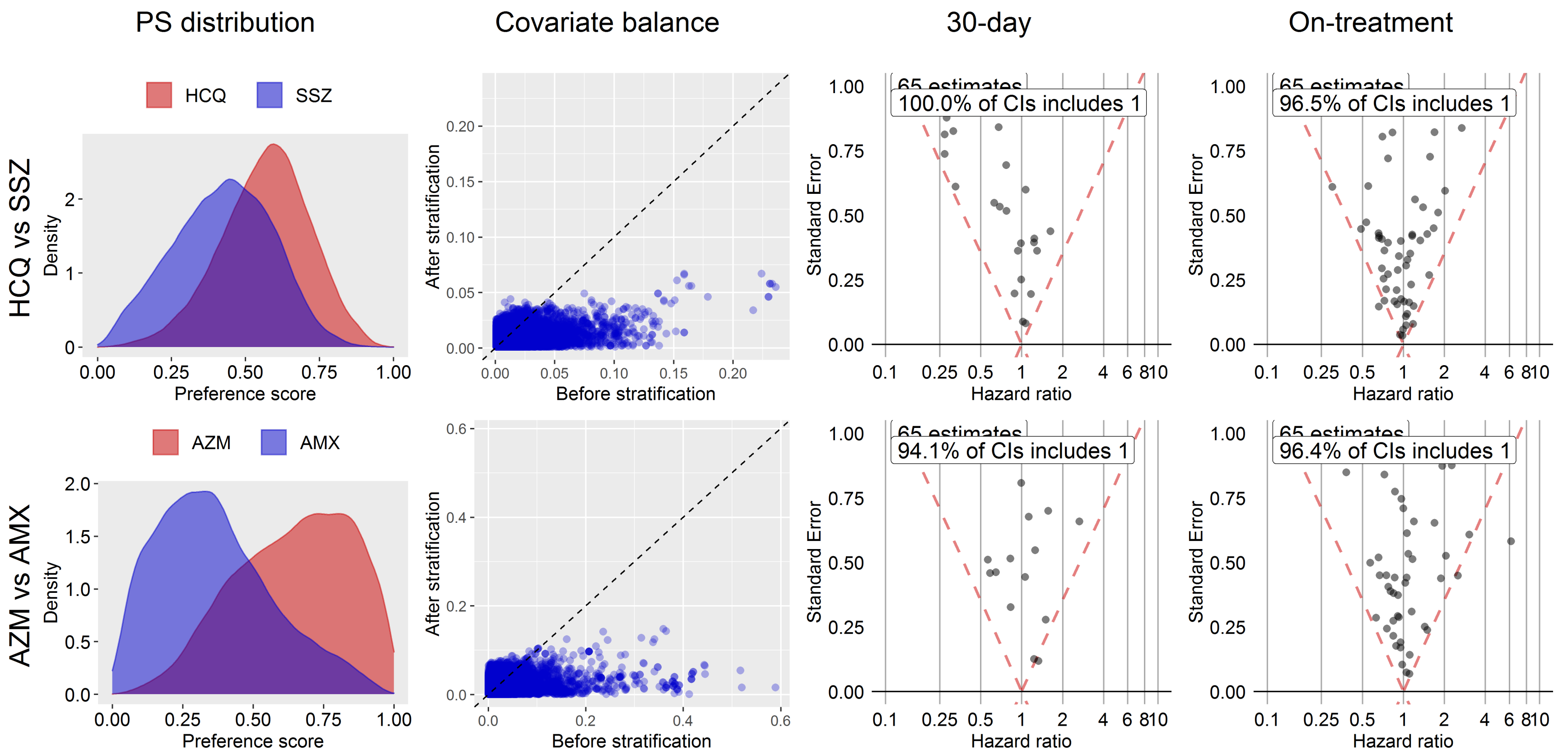
PS distribution
Covariate balance
30-day
On-treatment
HCQ vs SSZ
AZM vs AMX
HCQ
SSZ
AZM
AMX
Density
Preference score
After stratification
Before stratification
Standard Error
Hazard ratio
65 estimates
100.0% of CIs includes 1
96.5% of CIs includes 1
94.1% of CIs includes 1
96.4% of CIs includes 1

S10. Self-controlled case series results for hydroxychloroquine

S10.1. CCAE

Outcome	Analysis	Cases	IRR	95% CI LB	95% CI UB	Calibrated P	Calibrated IRR	Calibrated 95% CI LB	Calibrated 95% CI UB
Myocardial infarction	Adjusting for event-dependent observation	14,483	0.91	0.83	0.99	0.53	0.91	0.69	1.21
	Primary analysis	14,483	0.91	0.84	1	0.57	0.92	0.7	1.22
Acute pancreatitis events	Adjusting for event-dependent observation	13,221	NA	NA	NA	NA	NA	NA	NA
	Primary analysis	13,221	0.89	0.81	0.99	0.48	0.9	0.68	1.2
Acute renal failure	Adjusting for event-dependent observation	17,178	0.88	0.82	0.94	0.38	0.88	0.67	1.16
	Primary analysis	17,178	0.9	0.84	0.96	0.47	0.9	0.69	1.19
Gastrointestinal bleeding	Adjusting for event-dependent observation	15,638	NA	NA	NA	NA	NA	NA	NA
	Primary analysis	15,638	1	0.92	1.07	0.94	1.01	0.76	1.32
Cardiac arrhythmia	Adjusting for event-dependent observation	14,328	0.94	0.86	1.03	0.71	0.95	0.72	1.25
	Primary analysis	14,328	0.94	0.86	1.03	0.74	0.95	0.72	1.26
Bradycardia	Adjusting for event-dependent observation	12,248	NA	NA	NA	NA	NA	NA	NA
	Primary analysis	12,248	0.71	0.63	0.81	0.03	0.72	0.54	0.96
Chest pain or angina	Adjusting for event-dependent observation	13,399	0.9	0.81	1	0.53	0.91	0.69	1.21
	Primary analysis	13,399	0.9	0.81	1	0.52	0.91	0.69	1.21
End stage renal disease	Adjusting for event-dependent observation	11,303	1.01	0.65	1.16	0.93	1.02	0.69	1.51
	Primary analysis	11,303	1.02	0.88	1.18	0.85	1.03	0.76	1.39
Heart failure	Adjusting for event-dependent observation	15,626	0.98	0.9	1.05	0.91	0.99	0.75	1.29
	Primary analysis	15,626	0.98	0.91	1.06	0.93	0.99	0.75	1.3
Hepatic failure	Adjusting for event-dependent observation	11,254	0.67	0.57	0.78	0.01	0.68	0.5	0.92
	Primary analysis	11,254	0.64	0.54	0.75	0.01	0.64	0.47	0.88
Stroke	Adjusting for event-dependent observation	15,535	NA	NA	NA	NA	NA	NA	NA
	Primary analysis	15,535	0.79	0.73	0.86	0.12	0.8	0.61	1.06
Cardiovascular events	Adjusting for event-dependent observation	15,200	NA	NA	NA	NA	NA	NA	NA
	Primary analysis	15,200	0.86	0.79	0.93	0.3	0.86	0.66	1.14
Transient ischemic attack	Adjusting for event-dependent observation	14,563	0.9	0.85	0.98	0.51	0.91	0.69	1.2
	Primary analysis	14,563	0.91	0.83	0.99	0.55	0.92	0.7	1.21
Venous thromboembolism	Adjusting for event-dependent observation	16,595	0.78	0.5	0.84	0.22	0.79	0.54	1.15
	Primary analysis	16,595	0.8	0.75	0.86	0.14	0.81	0.62	1.07

IRR= Incidence Rate Ratio **95%CI LB**= 95% Confidence Interval Lower Boundary **95%CI UB**= 95% Confidence Interval Upper Boundary

S10.2. Clinformatics

Outcome	Analysis	Cases	IRR	95% CI LB	95% CI UB	Calibrated P	Calibrated IRR	Calibrated 95% CI LB	Calibrated 95% CI UB
Myocardial infarction	Adjusting for event-dependent observation	14,351	1.11	1.02	1.21	0.51	1.11	0.81	1.54
	Primary analysis	14,351	1.01	0.92	1.1	0.92	1.02	0.74	1.4
Acute pancreatitis events	Adjusting for event-dependent observation	12,730	1.04	0.93	1.16	0.78	1.05	0.75	1.46
	Primary analysis	12,730	1.04	0.93	1.16	0.78	1.05	0.75	1.46
Acute renal failure	Adjusting for event-dependent observation	14,919	0.96	0.48	1.04	0.88	0.96	0.58	1.59
	Primary analysis	14,919	0.99	0.91	1.07	0.94	0.99	0.72	1.37
Gastrointestinal bleeding	Adjusting for event-dependent observation	14,884	1.12	1.03	1.22	0.46	1.13	0.82	1.55
	Primary analysis	14,884	1.05	0.97	1.14	0.74	1.06	0.77	1.46
Cardiac arrhythmia	Adjusting for event-dependent observation	13,388	1.02	0.92	1.13	0.88	1.03	0.74	1.42
	Primary analysis	13,388	1.03	0.93	1.13	0.85	1.03	0.74	1.43
Bradycardia	Adjusting for event-dependent observation	13,682	0.9	0.77	1	0.58	0.91	0.65	1.27
	Primary analysis	13,682	0.92	0.83	1.01	0.63	0.92	0.67	1.28
Chest pain or angina	Adjusting for event-dependent observation	13,093	1.06	0.96	1.18	0.7	1.07	0.77	1.48
	Primary analysis	13,093	1.06	0.95	1.17	0.72	1.06	0.76	1.47
End stage renal disease	Adjusting for event-dependent observation	11,891	NA	NA	NA	NA	NA	NA	NA
	Primary analysis	11,891	1.26	1.11	1.42	0.17	1.26	0.9	1.76
Heart failure	Adjusting for event-dependent observation	13,817	1.14	1.04	1.25	0.41	1.15	0.83	1.58
	Primary analysis	13,817	1.12	1.02	1.23	0.47	1.13	0.82	1.56
Hepatic failure	Adjusting for event-dependent observation	11,571	NA	NA	NA	NA	NA	NA	NA
	Primary analysis	11,571	0.72	0.62	0.83	0.07	0.73	0.52	1.02
Stroke	Adjusting for event-dependent observation	14,342	0.97	0.89	1.05	0.86	0.97	0.7	1.34
	Primary analysis	14,342	0.89	0.82	0.97	0.51	0.9	0.65	1.24
Cardiovascular events	Adjusting for event-dependent observation	14,189	0.9	0.18	0.98	0.82	0.9	0.37	2.21
	Primary analysis	14,189	0.94	0.86	1.03	0.75	0.95	0.69	1.31
Transient ischemic attack	Adjusting for event-dependent observation	14,895	0.94	0.86	1.02	0.73	0.94	0.68	1.3
	Primary analysis	14,895	0.93	0.85	1.01	0.68	0.93	0.68	1.29
Venous thromboembolism	Adjusting for event-dependent observation	15,377	0.85	0.79	0.92	0.36	0.86	0.62	1.18
	Primary analysis	15,377	0.84	0.77	0.91	0.3	0.84	0.61	1.16

IRR= Incidence Rate Ratio **95%CI LB**= 95% Confidence Interval Lower Boundary **95%CI UB**= 95% Confidence Interval Upper Boundary

S10.3. CPRD

Outcome	Analysis	Cases	IRR	95% CI LB	95% CI UB	Calibrated P	Calibrated IRR	Calibrated 95% CI LB	Calibrated 95% CI UB
Myocardial infarction	Adjusting for event-dependent observation	0	NA	NA	NA	NA	NA	NA	NA
	Primary analysis	0	NA	NA	NA	NA	NA	NA	NA
Acute pancreatitis events	Adjusting for event-dependent observation	0	NA	NA	NA	NA	NA	NA	NA
	Primary analysis	0	NA	NA	NA	NA	NA	NA	NA
Acute renal failure	Adjusting for event-dependent observation	0	NA	NA	NA	NA	NA	NA	NA
	Primary analysis	0	NA	NA	NA	NA	NA	NA	NA
Gastrointestinal bleeding	Adjusting for event-dependent observation	0	NA	NA	NA	NA	NA	NA	NA
	Primary analysis	0	NA	NA	NA	NA	NA	NA	NA
Cardiac arrhythmia	Adjusting for event-dependent observation	12,614	0.86	0.77	0.97	0.81	0.95	0.61	1.47
	Primary analysis	12,614	0.87	0.77	0.98	0.83	0.95	0.61	1.48
Bradycardia	Adjusting for event-dependent observation	7,094	0.6	0.18	1.68	0.49	0.65	0.2	2.16
	Primary analysis	7,094	0.62	0.19	1.69	0.51	0.68	0.21	2.18
Chest pain or angina	Adjusting for event-dependent observation	13,376	0.89	0.8	1	0.9	0.98	0.63	1.52
	Primary analysis	13,376	0.89	0.8	1	0.9	0.98	0.63	1.52
End stage renal disease	Adjusting for event-dependent observation	1,496	0.84	0.14	4.51	0.92	0.91	0.15	5.49
	Primary analysis	1,496	0.83	0.14	4.41	0.91	0.91	0.15	5.31
Heart failure	Adjusting for event-dependent observation	10,215	1.1	0.76	1.56	0.53	1.2	0.69	2.09
	Primary analysis	10,215	1.1	0.77	1.58	0.52	1.21	0.69	2.11
Hepatic failure	Adjusting for event-dependent observation	2,710	1	NA	NA	NA	NA	NA	NA
	Primary analysis	2,710	0.09	0	0.77	0.08	0.09	0.01	1.35
Stroke	Adjusting for event-dependent observation	0	NA	NA	NA	NA	NA	NA	NA
	Primary analysis	0	NA	NA	NA	NA	NA	NA	NA
Cardiovascular events	Adjusting for event-dependent observation	0	NA	NA	NA	NA	NA	NA	NA
	Primary analysis	0	NA	NA	NA	NA	NA	NA	NA
Transient ischemic attack	Adjusting for event-dependent observation	0	NA	NA	NA	NA	NA	NA	NA
	Primary analysis	0	NA	NA	NA	NA	NA	NA	NA
Venous thromboembolism	Adjusting for event-dependent observation	12,454	0.64	0.56	0.72	0.12	0.7	0.45	1.09
	Primary analysis	12,454	0.63	0.55	0.71	0.11	0.69	0.44	1.07

IRR= Incidence Rate Ratio **95%CI LB**= 95% Confidence Interval Lower Boundary **95%CI UB**= 95% Confidence Interval Upper Boundary

S10.4. JMDC

Outcome	Analysis	Cases	IRR	95% CI LB	95% CI UB	Calibrated P	Calibrated IRR	Calibrated 95% CI LB	Calibrated 95% CI UB
Myocardial infarction	Adjusting for event-dependent observation	5,788	0	NA	0	NA	NA	NA	NA
	Primary analysis	5,789	0	NA	1.19	NA	NA	NA	NA
Acute pancreatitis events	Adjusting for event-dependent observation	5,307	1.42	0.06	25.06	0.62	2.18	0.11	43.82
	Primary analysis	5,307	1.38	0.06	13.42	0.6	2.11	0.14	31.5
Acute renal failure	Adjusting for event-dependent observation	3,598	0.86	0.18	3.12	0.73	1.33	0.31	5.71
	Primary analysis	3,598	0.91	0.19	3.37	0.69	1.39	0.32	6.12
Gastrointestinal bleeding	Adjusting for event-dependent observation	10,009	0.16	0.01	0.82	0.25	0.25	0.03	2.44
	Primary analysis	10,009	0.16	0.01	0.79	0.24	0.24	0.03	2.38
Cardiac arrhythmia	Adjusting for event-dependent observation	10,034	0.4	0.11	1.2	0.5	0.62	0.18	2.15
	Primary analysis	10,034	0.38	0.1	1.11	0.43	0.58	0.17	1.98
Bradycardia	Adjusting for event-dependent observation	5,813	2.4	0.11	20.87	0.35	3.67	0.26	50.91
	Primary analysis	5,813	2.41	0.11	21.25	0.35	3.69	0.26	51.54
Chest pain or angina	Adjusting for event-dependent observation	10,082	0.6	0.32	1.09	0.74	0.92	0.45	1.85
	Primary analysis	10,082	0.6	0.31	1.08	0.74	0.91	0.45	1.84
End stage renal disease	Adjusting for event-dependent observation	2,439	NA	NA	NA	NA	NA	NA	NA
	Primary analysis	2,445	NA	NA	NA	NA	NA	NA	NA
Heart failure	Adjusting for event-dependent observation	10,071	0.67	0.34	1.23	0.77	1.02	0.5	2.1
	Primary analysis	10,071	0.66	0.34	1.22	0.76	1.02	0.49	2.08
Hepatic failure	Adjusting for event-dependent observation	3,014	1	0.04	15.31	0.79	1.54	0.08	30.15
	Primary analysis	3,014	0.97	0.04	18.75	0.81	1.48	0.07	33.23
Stroke	Adjusting for event-dependent observation	10,001	0.74	0.23	2.1	0.8	1.13	0.36	3.55
	Primary analysis	10,001	0.74	0.23	2.12	0.8	1.14	0.36	3.59
Cardiovascular events	Adjusting for event-dependent observation	10,035	0.33	0.13	0.72	0.21	0.51	0.21	1.25
	Primary analysis	10,035	0.33	0.13	0.72	0.2	0.5	0.2	1.25
Transient ischemic attack	Adjusting for event-dependent observation	2,208	0	NA	0	NA	NA	NA	NA
	Primary analysis	2,208	0	NA	38.67	NA	NA	NA	NA
Venous thromboembolism	Adjusting for event-dependent observation	10,029	0.98	0.41	2.14	0.45	1.51	0.62	3.67
	Primary analysis	10,029	0.98	0.41	2.14	0.45	1.51	0.62	3.67

IRR= Incidence Rate Ratio **95%CI LB**= 95% Confidence Interval Lower Boundary **95%CI UB**= 95% Confidence Interval Upper Boundary

S10.5. MDCD

Outcome	Analysis	Cases	IRR	95% CI LB	95% CI UB	Calibrated P	Calibrated IRR	Calibrated 95% CI LB	Calibrated 95% CI UB
Myocardial infarction	Adjusting for event-dependent observation	11,276	NA	NA	NA	NA	NA	NA	NA
	Primary analysis	11,276	0.97	0.83	1.13	0.39	0.89	0.7	1.15
Acute pancreatitis events	Adjusting for event-dependent observation	10,851	1.22	1.02	1.46	0.39	1.13	0.86	1.47
	Primary analysis	10,851	1.22	1.02	1.45	0.4	1.12	0.86	1.46
Acute renal failure	Adjusting for event-dependent observation	12,714	NA	NA	NA	NA	NA	NA	NA
	Primary analysis	12,714	1.09	0.98	1.21	0.92	1	0.8	1.25
Gastrointestinal bleeding	Adjusting for event-dependent observation	12,000	1.03	0.91	1.16	0.66	0.95	0.75	1.2
	Primary analysis	12,000	1.03	0.91	1.17	0.69	0.95	0.76	1.2
Cardiac arrhythmia	Adjusting for event-dependent observation	12,340	1.01	0.89	1.13	0.53	0.93	0.74	1.17
	Primary analysis	12,340	1.01	0.89	1.13	0.54	0.93	0.74	1.17
Bradycardia	Adjusting for event-dependent observation	10,541	NA	NA	NA	NA	NA	NA	NA
	Primary analysis	10,541	0.8	0.64	1	0.05	0.74	0.55	0.99
Chest pain or angina	Adjusting for event-dependent observation	11,642	1.17	0.98	1.34	0.59	1.07	0.84	1.38
	Primary analysis	11,642	1.16	1.01	1.34	0.58	1.07	0.84	1.36
End stage renal disease	Adjusting for event-dependent observation	10,562	NA	NA	NA	NA	NA	NA	NA
	Primary analysis	10,562	1.34	1.1	1.64	0.15	1.24	0.93	1.64
Heart failure	Adjusting for event-dependent observation	12,030	1.03	0.91	1.17	0.68	0.95	0.75	1.2
	Primary analysis	12,030	1.03	0.91	1.17	0.68	0.95	0.75	1.2
Hepatic failure	Adjusting for event-dependent observation	10,278	0.91	0.7	1.17	0.29	0.83	0.6	1.16
	Primary analysis	10,278	0.83	0.64	1.09	0.13	0.77	0.55	1.07
Stroke	Adjusting for event-dependent observation	11,662	0.98	0.86	1.12	0.4	0.9	0.71	1.14
	Primary analysis	11,662	0.92	0.81	1.06	0.2	0.85	0.67	1.08
Cardiovascular events	Adjusting for event-dependent observation	12,067	0.99	0.88	1.12	0.46	0.91	0.73	1.15
	Primary analysis	12,067	0.93	0.82	1.05	0.21	0.86	0.68	1.08
Transient ischemic attack	Adjusting for event-dependent observation	10,938	1.02	0.86	1.22	0.66	0.94	0.72	1.23
	Primary analysis	10,938	1	0.84	1.19	0.57	0.92	0.71	1.2
Venous thromboembolism	Adjusting for event-dependent observation	12,646	0.96	0.86	1.07	0.29	0.88	0.71	1.1
	Primary analysis	12,646	0.95	0.85	1.05	0.25	0.87	0.7	1.09

IRR= Incidence Rate Ratio **95%CI LB**= 95% Confidence Interval Lower Boundary **95%CI UB**= 95% Confidence Interval Upper Boundary

S10.6. MDCR

Outcome	Analysis	Cases	IRR	95% CI LB	95% CI UB	Calibrated P	Calibrated IRR	Calibrated 95% CI LB	Calibrated 95% CI UB
Myocardial infarction	Adjusting for event-dependent observation	14,554	1.04	0.96	1.13	0.47	1.09	0.86	1.39
	Primary analysis	14,554	0.94	0.86	1.03	0.91	0.99	0.78	1.26
Acute pancreatitis events	Adjusting for event-dependent observation	10,998	0.98	0.82	1.16	0.86	1.03	0.77	1.36
	Primary analysis	10,998	0.94	0.79	1.12	0.93	0.99	0.74	1.32
Acute renal failure	Adjusting for event-dependent observation	15,657	1.03	0.95	1.11	0.53	1.08	0.85	1.37
	Primary analysis	15,657	0.92	0.85	1	0.8	0.97	0.76	1.23
Gastrointestinal bleeding	Adjusting for event-dependent observation	15,255	0.97	0.9	1.05	0.85	1.02	0.81	1.3
	Primary analysis	15,255	0.92	0.84	0.99	0.75	0.96	0.76	1.22
Cardiac arrhythmia	Adjusting for event-dependent observation	13,552	0.81	0.73	0.9	0.22	0.85	0.67	1.09
	Primary analysis	13,552	0.82	0.74	0.9	0.24	0.86	0.67	1.1
Bradycardia	Adjusting for event-dependent observation	12,752	0.83	0.74	0.93	0.29	0.87	0.68	1.12
	Primary analysis	12,752	0.83	0.74	0.93	0.28	0.87	0.68	1.12
Chest pain or angina	Adjusting for event-dependent observation	14,060	0.91	0.82	0.99	0.69	0.95	0.75	1.21
	Primary analysis	14,060	0.9	0.82	0.99	0.65	0.94	0.74	1.2
End stage renal disease	Adjusting for event-dependent observation	10,839	0.84	0.69	1.01	0.41	0.88	0.66	1.18
	Primary analysis	10,839	0.84	0.7	1.02	0.42	0.88	0.66	1.19
Heart failure	Adjusting for event-dependent observation	14,531	1.06	0.98	1.16	0.37	1.12	0.88	1.42
	Primary analysis	14,531	1.04	0.96	1.13	0.48	1.09	0.86	1.39
Hepatic failure	Adjusting for event-dependent observation	10,325	0.78	0.6	1.03	0.28	0.82	0.58	1.17
	Primary analysis	10,325	0.77	0.59	1.01	0.25	0.81	0.57	1.15
Stroke	Adjusting for event-dependent observation	14,536	0.96	0.88	1.05	0.91	1.01	0.8	1.29
	Primary analysis	14,536	0.89	0.81	0.97	0.57	0.93	0.73	1.18
Cardiovascular events	Adjusting for event-dependent observation	14,573	1.05	0.97	1.14	0.43	1.1	0.87	1.4
	Primary analysis	14,573	0.97	0.89	1.05	0.89	1.02	0.8	1.29
Transient ischemic attack	Adjusting for event-dependent observation	13,727	0.95	0.87	1.05	0.93	1	0.79	1.28
	Primary analysis	13,727	0.93	0.84	1.02	0.82	0.97	0.76	1.24
Venous thromboembolism	Adjusting for event-dependent observation	16,067	0.72	0.67	0.78	0.02	0.76	0.6	0.96
	Primary analysis	16,067	0.68	0.63	0.73	0.01	0.71	0.56	0.91

IRR= Incidence Rate Ratio **95%CI LB**= 95% Confidence Interval Lower Boundary **95%CI UB**= 95% Confidence Interval Upper Boundary

S10.7. VA

Outcome	Analysis	Cases	IRR	95% CI LB	95% CI UB	Calibrated P	Calibrated IRR	Calibrated 95% CI LB	Calibrated 95% CI UB
Myocardial infarction	Adjusting for event-dependent observation	15,437	1.30	1.20	1.40	0.94	0.99	0.73	1.34
	Primary analysis	15,437	1.31	1.21	1.42	0.94	1.00	0.74	1.36
Acute pancreatitis events	Adjusting for event-dependent observation	11,824	1.27	1.11	1.45	0.87	0.97	0.71	1.34
	Primary analysis	11,825	1.28	1.12	1.46	0.91	0.98	0.71	1.35
Acute renal failure	Adjusting for event-dependent observation	17,735	NA	NA	NA	NA	NA	NA	NA
	Primary analysis	17,735	1.45	1.36	1.55	0.48	1.11	0.83	1.50
Gastrointestinal bleeding	Adjusting for event-dependent observation	15,613	NA	NA	NA	NA	NA	NA	NA
	Primary analysis	15,613	1.27	1.18	1.37	0.87	0.97	0.72	1.32
Cardiac arrhythmia	Adjusting for event-dependent observation	15,593	1.12	1.04	1.22	0.34	0.86	0.64	1.17
	Primary analysis	15,593	1.11	1.02	1.20	0.29	0.85	0.63	1.15
Bradycardia	Adjusting for event-dependent observation	13,234	1.15	1.04	1.27	0.42	0.88	0.65	1.20
	Primary analysis	13,234	1.21	1.10	1.34	0.64	0.93	0.68	1.26
Chest pain or angina	Adjusting for event-dependent observation	16,121	NA	NA	NA	NA	NA	NA	NA
	Primary analysis	16,121	1.29	1.19	1.39	0.92	0.98	0.73	1.33
End stage renal disease	Adjusting for event-dependent observation	11,687	1.36	1.19	1.56	0.79	1.04	0.76	1.44
	Primary analysis	11,687	1.33	1.16	1.52	0.92	1.02	0.74	1.40
Heart failure	Adjusting for event-dependent observation	15,772	1.34	1.25	1.45	0.85	1.03	0.76	1.39
	Primary analysis	15,772	1.36	1.26	1.46	0.80	1.04	0.77	1.40
Hepatic failure	Adjusting for event-dependent observation	11,066	NA	NA	NA	NA	NA	NA	NA
	Primary analysis	11,066	1.03	0.85	1.23	0.18	0.79	0.56	1.11
Stroke	Adjusting for event-dependent observation	13,627	1.26	1.14	1.39	0.82	0.96	0.71	1.31
	Primary analysis	13,627	1.29	1.17	1.42	0.92	0.98	0.72	1.34
Cardiovascular events	Adjusting for event-dependent observation	16,687	NA	NA	NA	NA	NA	NA	NA
	Primary analysis	16,687	1.41	1.31	1.51	0.63	1.08	0.80	1.46
Transient ischemic attack	Adjusting for event-dependent observation	12,315	NA	NA	NA	NA	NA	NA	NA
	Primary analysis	12,315	1.57	1.40	1.77	0.25	1.20	0.88	1.65
Venous thromboembolism	Adjusting for event-dependent observation	17,469	NA	NA	NA	NA	NA	NA	NA
	Primary analysis	17,469	1.12	1.05	1.20	0.33	0.86	0.64	1.16

IRR= Incidence Rate Ratio **95%CI LB**= 95% Confidence Interval Lower Boundary **95%CI UB**= 95% Confidence Interval Upper Boundary

S11. Literature review sources

S11.1. PubMed search strategy

(((((((("Antirheumatic Agents"[Mesh]) OR (((disease modifying antirheumatic drug*[Title/Abstract] OR disease modifying anti-rheumatic drug*[Title/Abstract] OR disease modifying antirheumatic agent*[Title/Abstract] OR disease modifying anti-rheumatic agent*[Title/Abstract]) OR (DMARD*[Title/Abstract])))) OR ((((((((("hydroxychloroquine"[MeSH Terms]) OR Hydroxychloroquine[Title/Abstract]) OR chloroquinol[Title/Abstract]) OR ercoquin[Title/Abstract]) OR hydrochloroquine[Title/Abstract]) OR hydrocloroquine[Title/Abstract]) OR oxychloroquine[Title/Abstract]) OR plaquenil[Title/Abstract]) OR quensyl[Title/Abstract]) OR "sn 8137"[Title/Abstract])) OR (((((((("tocilizumab"[MeSH Terms]) OR tocilizumab[Title/Abstract]) OR actemra*[Title/Abstract]) OR atlizumab[Title/Abstract]) OR lusinex[Title/Abstract]) OR "r 1569"[Title/Abstract]) OR r1569[Title/Abstract]) OR roactemra[Title/Abstract])) OR "baricitinib"[Supplementary Concept]) OR (((((((baricitinib[Title/Abstract]) OR "incb 028050"[Title/Abstract]) OR "incb 28050"[Title/Abstract]) OR "incb028050"[Title/Abstract]) OR "incb28050"[Title/Abstract]) OR "ly 3009104"[Title/Abstract]) OR "ly3009104"[Title/Abstract]) OR olumiant[Title/Abstract])) OR ((((((((((((((((((((((("methotrexate"[MeSH Terms]) OR methotrexate[Title/Abstract]) OR "a methopterine"[Title/Abstract]) OR abitrexate[Title/Abstract]) OR amethopterin*[Title/Abstract]) OR ametopterine[Title/Abstract]) OR antifolan[Title/Abstract]) OR biotrexate[Title/Abstract]) OR canceren[Title/Abstract]) OR "cl 14377"[Title/Abstract]) OR cl14377[Title/Abstract]) OR emtexate[Title/Abstract]) OR emthexat*[Title/Abstract]) OR enthexate[Title/Abstract]) OR emtrexate[Title/Abstract]) OR farmitrexat*[Title/Abstract]) OR farmotrex[Title/Abstract]) OR folex*[Title/Abstract]) OR ifamet[Title/Abstract]) OR imeth[Title/Abstract]) OR "intradose MTX"[Title/Abstract]) OR jylamvo[Title/Abstract]) OR lantarel[Title/Abstract]) OR ledertrexate[Title/Abstract])) OR ((((((((((((((((((((((((((((maxtrex[Title/Abstract]) OR metex[Title/Abstract]) OR methoblastin[Title/Abstract]) OR methohexate[Title/Abstract]) OR methotrate[Title/Abstract]) OR methotrexat*[Title/Abstract]) OR methoxtrexate[Title/Abstract]) OR methrotrexate[Title/Abstract]) OR methylaminopterin*[Title/Abstract]) OR meticil[Title/Abstract]) OR metoject[Title/Abstract]) OR metothrexate[Title/Abstract]) OR metotrex*[Title/Abstract]) OR metrex[Title/Abstract]) OR mexate*[Title/Abstract]) OR "mpi 5004"[Title/Abstract]) OR mpi5004[Title/Abstract]) OR MTX[Title/Abstract]) OR neotrexate[Title/Abstract]) OR nordimet[Title/Abstract]) OR novatrex[Title/Abstract]) OR "nsc 740"[Title/Abstract]) OR nsc740 AND "[Title/Abstract]) OR otrexup[Title/Abstract]) OR rasuvo[Title/Abstract]) OR reumatrex[Title/Abstract]) OR rheumatrex[Title/Abstract]) OR texate*[Title/Abstract]) OR texorate[Title/Abstract]) OR trexall[Title/Abstract]) OR xaken[Title/Abstract]) OR xatmep[Title/Abstract]) OR zexate[Title/Abstract])) OR ((((((((((((((((((((((((((((((sulfasalazine[MeSH Terms]) OR salazosulfapyridine[Title/Abstract]) OR " AND azlufidine en-tabs AND "[Title/Abstract]) OR azopyrin*[Title/Abstract]) OR azosulfidine[Title/Abstract]) OR azulfid*[Title/Abstract]) OR azulfin[Title/Abstract]) OR benzosulfa[Title/Abstract]) OR " AND colo pleon AND "[Title/Abstract]) OR " AND colo-pleon AND "[Title/Abstract]) OR colopleon[Title/Abstract]) OR disalazin[Title/Abstract]) OR gastropyrin[Title/Abstract]) OR " AND pleon ra AND "[Title/Abstract]) OR " AND pyralin en AND "[Title/Abstract]) OR rorasul[Title/Abstract]) OR rosulfant[Title/Abstract]) OR " AND s.a.s.-500 AND "[Title/Abstract]) OR salaz*[Title/Abstract]) OR " AND salicyl azo sulfapyridine AND "[Title/Abstract]) OR salicylazosulfapyridin*[Title/Abstract]) OR salisulf[Title/Abstract]) OR salopyr[Title/Abstract]) OR saridine[Title/Abstract]) OR " AND sas 500 Title/Abstract) OR sulcolon[Title/Abstract]) OR sulfasalazine[Title/Abstract]) OR sulfasalizine[Title/Abstract]) OR sulfosalazine[Title/Abstract]) OR sulphasalazine[Title/Abstract]) OR zopyrin[Title/Abstract]))) AND ((((((((((((((((((((((((((((("coronavirus infections"[MeSH Terms]) OR coronavirus*[Title/Abstract]) OR coronovirus*[Title/Abstract]) OR "2019-nCoV"[Title/Abstract]) OR "COVID-19"[Title/Abstract]) OR "CORVID-19"[Title/Abstract]) OR "WN-CoV"[Title/Abstract]) OR "HCoV-19"[Title/Abstract]) OR CoV[Title/Abstract]) OR ncov[Title/Abstract]) OR "SARS-CoV-2"[Title/Abstract]) OR SARSCov19[Title/Abstract]) OR ncov*wuhan[Title/Abstract]) OR "severe acute respiratory syndrome"[MeSH Terms]) OR "severe acute respiratory syndrome"[Title/Abstract]) OR "sudden acute respiratory syndrome"[Title/Abstract]) OR SARS[Title/Abstract]) OR MERS[Title/Abstract]) OR "Middle East Respiratory Syndrome"[Title/Abstract]) OR sars coronavirus[MeSH Terms]) OR viral infection[Title/Abstract]) OR viral disease[Title/Abstract]) OR virus infection[Title/Abstract]) OR virus disease[Title/Abstract]) OR viral pneumonia[MeSH Terms]) OR viral pneumonia[Title/Abstract]) OR influenza[MeSH Terms]) OR influenza[Title/Abstract]) OR flu[Title])

S11.2. Embase search strategy (1974 to present)

1	disease modifying antirheumatic drug/ (16237)
2	disease modifying antirheumatic drug*.ti,ab. (6045)
3	disease modifying anti-rheumatic drug*.ti,ab. (3750)
4	disease modifying antirheumatic agent*.ti,ab. (38)
5	disease modifying anti-rheumatic agent*.ti,ab. (37)
6	DMARD*.ti,ab. (14249)
7	hydroxychloroquine/ (23310)
8	Hydroxychloroquine.ti,ab. (7610)
9	chloroquinol.ti,ab. (2)
10	ercoquin.ti,ab. (0)
11	hydrochloroquine.ti,ab. (56)
12	hydrocloroquine.ti,ab. (0)
13	oxychloroquine.ti,ab. (6)
14	plaquenil.ti,ab. (305)
15	quensyl.ti,ab. (4)
16	"sn 8137".ti,ab. (1)
17	tocilizumab/ (10914)
18	tocilizumab.ti,ab. (6966)
19	actemra*.ti,ab. (71)
20	atlizumab.ti,ab. (21)
21	lusinex.ti,ab. (0)
22	"r 1569".ti,ab. (0)
23	r1569.ti,ab. (0)
24	roactemra.ti,ab. (43)
25	baricitinib/ (944)
26	Baricitinib.ti,ab. (600)
27	"incb 028050".ti,ab. (2)
28	"incb 28050".ti,ab. (1)
29	"incb028050".ti,ab. (18)
30	"incb28050".ti,ab. (0)
31	"ly 3009104".ti,ab. (2)
32	"ly3009104".ti,ab. (19)
33	olumiant.ti,ab. (8)
34	methotrexate/ (173846)
35	methotrexate.ti,ab. (65636)
36	"a methopterine".ti,ab. (0)
37	abitrexate.ti,ab. (0)
38	amethopterin*.ti,ab. (232)
39	ametopterine.ti,ab. (1)
40	antifolan.ti,ab. (0)
41	biotrexate.ti,ab. (3)
42	canceren.ti,ab. (1)
43	"cl 14377".ti,ab. (0)
44	cl14377.ti,ab. (0)
45	emtexate.ti,ab. (0)
46	emthexat*.ti,ab. (2)
47	emtrexate.ti,ab. (1)
48	enthexate.ti,ab. (0)
49	farmitrexat*.ti,ab. (0)
50	farmotrex.ti,ab. (0)
51	folex*.ti,ab. (3)
52	ifamet.ti,ab. (0)
53	imeth.ti,ab. (2)
54	"intradose MTX".ti,ab. (0)
55	jylamvo.ti,ab. (0)
56	lantarel.ti,ab. (3)
57	ledertrexate.ti,ab. (4)
58	maxtrex.ti,ab. (0)
59	metex.ti,ab. (12)
60	methoblastin.ti,ab. (0)
61	methohexate.ti,ab. (0)
62	methotrate.ti,ab. (4)

63	methotrexat*.ti,ab. (65844)
64	methoxtrexate.ti,ab. (3)
65	methrotrexate.ti,ab. (62)
66	methylaminopterin*.ti,ab. (6)
67	meticil.ti,ab. (0)
68	metoject.ti,ab. (13)
69	metothrexate.ti,ab. (108)
70	metotrex*.ti,ab. (257)
71	metrex.ti,ab. (18)
72	mexate*.ti,ab. (1)
73	"mpi 5004".ti,ab. (0)
74	mpi5004.ti,ab. (0)
75	MTX.ti,ab. (23376)
76	neotrexate.ti,ab. (0)
77	nordimet.ti,ab. (1)
78	novatrex.ti,ab. (0)
79	"nsc 740".ti,ab. (34)
80	"nsc740".ti,ab. (0)
81	otrexup.ti,ab. (2)
82	rasuvo.ti,ab. (1)
83	reumatrex.ti,ab. (0)
84	rheumatrex.ti,ab. (6)
85	texate*.ti,ab. (2)
86	texorate.ti,ab. (0)
87	trexall.ti,ab. (3)
88	xaken.ti,ab. (0)
89	xatmep.ti,ab. (0)
90	zexate.ti,ab. (0)
91	salazosulfapyridine/ (24991)
92	salazosulfapyridine.ti,ab. (403)
93	"azlufidine en-tabs".ti,ab. (0)
94	azopyrin*.ti,ab. (0)
95	azosulfidine.ti,ab. (1)
96	azulfid*.ti,ab. (81)
97	azulfin.ti,ab. (0)
98	benzosulfa.ti,ab. (0)
99	"colo pleon".ti,ab. (2)
100	"colo-pleon".ti,ab. (2)
101	colopleon.ti,ab. (0)
102	disalazin.ti,ab. (0)
103	gastropyrin.ti,ab. (0)
104	"pleon ra".ti,ab. (0)
105	"pyralin en".ti,ab. (0)
106	rorasul.ti,ab. (0)
107	rosulfant.ti,ab. (0)
108	"s.a.s.-500".ti,ab. (2)
109	salaz*.ti,ab. (1188)
110	"salicyl azo sulfapyridine".ti,ab. (2)
111	salicylazosulfapyridin*.ti,ab. (181)
112	salisulf.ti,ab. (0)
113	salopyr.ti,ab. (0)
114	saridine.ti,ab. (0)
115	"sas 500".ti,ab. (2)
116	sulcolon.ti,ab. (0)
117	sulfasalazine.ti,ab. (5325)
118	sulfasalizine.ti,ab. (4)
119	sulfosalazine.ti,ab. (5)
120	sulphasalazine.ti,ab. (1742)
121	zopyrin.ti,ab. (0)
122	1 or 2 or 3 or 4 or 5 or 6 or 7 or 8 or 9 or 10 or 11 or 12 or 13 or 14 or 15 or 16 or 17 or 18 or 19 or 20 or 21 or 22 or 23 or 24 or 25 or 26 or 27 or 28 or 29 or 30 or 31 or 32 or 33 or 34 or 35 or 36 or 37 or 38 or 39 or 40 or 41 or 42 or 43 or 44 or 45 or 46 or 47 or 48 or 49 or 50 or 51 or 52 or 53 or 54 or 55 or 56 or 57 or 58 or 59 or 60 or 61 or 62 or 63 or 64 or 65 or 66 or 67 or 68 or 69 or 70 or 71 or 72 or 73 or 74 or 75 or 76 or 77 or 78 or 79 or 80 or 81 or 82 or 83 or 84 or 85 or 86 or 87 or 88 or 89 or 90 or 91 or 92 or 93 or 94 or 95 or 96 or 97 or 98 or 99 or 100 or 101 or 102 or 103 or 104 or 105 or 106 or 107 or 108 or 109 or 110 or 111 or 112 or 113 or 114 or 115 or 116 or 117 or 118 or 119 or 120 or 121 (226851)
123	Coronavirus infection/ (1778)
124	coronavirus*.ti,ab. (11962)

125	coronovirus*.ti,ab. (19)
126	"2019-nCoV".ti,ab. (233)
127	"COVID-19".ti,ab. (255)
128	"CORVID-19".ti,ab. (0)
129	"WN-CoV".ti,ab. (0)
130	"HCoV-19".ti,ab. (0)
131	CoV.ti,ab. (6191)
132	ncov.ti,ab. (246)
133	"SARS-CoV-2".ti,ab. (102)
134	SARSCov19.ti,ab. (0)
135	ncov*wuhan.ti,ab. (0)
136	((outbreak* or "respiratory illness" or "respiratory disease" or respiratory symptom* or seafood market or food market or wildlife) and (Wuhan or China or Chinese)).ti,ab. (5699)
137	severe acute respiratory syndrome/ (8192)
138	"severe acute respiratory syndrome".ti,ab. (4884)
139	"sudden acute respiratory syndrome".ti,ab. (9)
140	SARS.ti,ab. (9457)
141	MERS.ti,ab. (4478)
142	"Middle East Respiratory Syndrome".ti,ab. (1889)
143	SARS coronavirus/ (4579)
144	virus infection/ (158726)
145	((virus* or viral) adj (infection* or disease*)).ti,ab. (154324)
146	virus pneumonia/ (6683)
147	((viral or virus) adj3 pneumonia).ti,ab. (2989)
148	exp influenza/ (83690)
149	influenza.ti,ab. (103586)
150	flu.ti,ab. (19565)
151	123 or 124 or 125 or 126 or 127 or 128 or 129 or 131 or 132 or 133 or 134 or 135 or 136 or 137 or 138 or 139 or 140 or 141 or 142 or 143 or 144 or 145 or 146 or 147 or 148 or 149 or 150 (408377)
152	122 and 151 (4890)
153	limit 152 to (chinese or english or italian or spanish) (4681)

S11.3. ClinicalTrials.gov search strategy

for: "disease modifying antirheumatic drug" OR "disease modifying anti-rheumatic drug" OR DMARD OR Hydroxychloroquine OR tocilizumab OR baricitinib OR methotrexate OR Sulfasalazine | Corona Virus Infection OR coronavirus OR coronovirus OR Covid-19 OR "2019-nCoV" OR "SARS-CoV-2" OR "SARSCov19" OR SARS OR "severe acute respiratory syndrome" OR MERS OR "Middle East Respiratory Syndrome" OR viral pneumonia OR influenza

S11.4. ICTRP strategy

CORONAVIRUS INFECTIONS, CORONAVIRUS SARS-ASSOCIATED, CORONAVIRUS SARS-ASSOCIATED (DIAGNOSIS), MERS, RESPIRATORY SYNDROME, ACUTE, SEVERE, RESPIRATORY SYNDROME, SEVERE ACUTE, SARS, SARS ASSOC CORONAVIRUS, SARS-ASSOCIATED CORONAVIRUS, severe acute respiratory syndrome - middle east respiratory syndrome - CORONAVIRUS INFECT, CORONAVIRUS INFECTION, CORONAVIRUS INFECTION (DIAGNOSIS), CORONAVIRUS INFECTION (DISORDER), CORONAVIRUS INFECTION, UNSPECIFIED, CORONAVIRUS INFECTIONS, CORONAVIRUS INFECTIONS [DISEASE/FINDING], INFECT CORONAVIRUS, INFECTION, CORONAVIRUS, INFECTION; VIRAL, CORONAVIRUS, INFECTIONS, CORONAVIRUS, corona virus infection - PNEUMONIA VIRAL, PNEUMONIA VIRAL NOS, PNEUMONIA, VIRAL, PNEUMONIA, VIRAL [DISEASE/FINDING], PNEUMONIA; VIRAL, PNEUMONIA; VIRUS, PNEUMONIA;VIRAL, PNEUMONIAS, VIRAL, VIRAL PNEUMONITIS, VIRAL; PNEUMONIA, VIRUS; PNEUMONIA, viral pneumonia - coronovirus - CORONA VIRUS INFECTION, CORONAVIRIDAE, FAMILY CORONAVIRIDAE, FAMILY CORONAVIRIDAE (ORGANISM), coronavirus - sars-cov-2 - sarscov19 - AIDS, FLU, FLU SYNDROME, FLU VACCINE, FLU VACCINES, FLU, HUMAN, GRIPPE, HUMAN FLU, SYNDROME FLU, VACCINES, FLU, influenza - 2019-ncov - covid-19 - CORONAVIRUS INFECTIONS, FLJ20450, MERS, MITOCHONDRIAL SERRS, MTSERRS, RESPIRATORY SYNDROME, ACUTE, SEVERE, RESPIRATORY SYNDROME, SEVERE ACUTE, SERINE TRNA LIGASE 1, CYTOPLASMIC, SERINE TRNA LIGASE 2, MITOCHONDRIAL, SERRS, SERRSMT, SERS, SERYL-TRNA SYNTHETASE, SERYL-TRNA SYNTHETASE 2, SERYL-TRNA SYNTHETASE 2, MITOCHONDRIAL, SERYL-TRNA SYNTHETASE, MITOCHONDRIAL, SEVERE ACUTE RESPIRATORY SYNDROME, SEVERE ACUTE RESPIRATORY SYNDROME (DISORDER), SEVERE ACUTE RESPIRATORY SYNDROME [DISEASE/FINDING], SEVERE ACUTE RESPIRATORY SYNDROME NOS, SEVERE ACUTE RESPIRATORY SYNDROME VIRUS, SEVERE ACUTE RESPIRATORY SYNDROME-RELATED CORONAVIRUS, SEVERE ACUTE RESPIRATORY SYNDROME-RELATED CORONAVIRUS (ORGANISM), SYS, sars - CORONA VIRUS INFECTION, CORONAVIRUS INFECT, CORONAVIRUS INFECTION, CORONAVIRUS INFECTION (DIAGNOSIS), CORONAVIRUS INFECTION (DISORDER), CORONAVIRUS INFECTION, UNSPECIFIED, CORONAVIRUS INFECTIONS, CORONAVIRUS INFECTIONS [DISEASE/FINDING], CORONAVIRUS SARS-ASSOCIATED, CORONAVIRUS SARS-ASSOCIATED (DIAGNOSIS), INFECT CORONAVIRUS, INFECTION, CORONAVIRUS, INFECTION; VIRAL, CORONAVIRUS, INFECTIONS, CORONAVIRUS, RESPIRATORY SYNDROME, ACUTE, SEVERE, RESPIRATORY SYNDROME, SEVERE ACUTE, SARS, SARS (SEVERE ACUTE RESPIRATORY SYNDROME), SARS ASSOC CORONAVIRUS, SARS-ASSOCIATED CORONAVIRUS, SEA, SEA (ENVIRONMENT), SEAS, SEVERE ACUTE

RESPIRATORY SYNDROME, SEVERE ACUTE RESPIRATORY SYNDROME (DISORDER), SEVERE ACUTE RESPIRATORY SYNDROME (SARS), SEVERE ACUTE RESPIRATORY SYNDROME (SARS) (DIAGNOSIS), SEVERE ACUTE RESPIRATORY SYNDROME [DISEASE/FINDING], SEVERE ACUTE RESPIRATORY SYNDROME NOS, mers - disease modifying anti-rheumatic drug - disease modifying antirheumatic drug - CHLOROQUINE, HCQ, HYDROXYCHLOROCHIN, OXYCHLOROCHIN, OXYCHLOROQUINE, hydroxychloroquine - SALAZOSULFAPYRIDINE, SALAZOSULPHAPYRIDINE, SALICYLAZOSULFAPYRIDINE, SALICYLAZOSULPHAPYRIDINE, SULFANILAMIDE, SULPHASALAZINE, sulfasalazine - AMETHOPTERIN, MTX, methotrexate - ATLIZUMAB, MONOCLONAL ANTIBODY, MRA, MRA, tocilizumab - baricitinib - [MS100] ANTIRHEUMATICS, AGENTS, ANTI-RHEUMATIC, AGENTS, ANTIRHEUMATIC, ANTI RHEUMATIC AGENTS, ANTI RHEUMATIC DRUGS, ANTI-RHEUMATIC AGENT, ANTI-RHEUMATIC AGENT (PRODUCT), ANTI-RHEUMATIC AGENT, NOS, ANTI-RHEUMATIC AGENTS, ANTI-RHEUMATIC DRUGS, ANTIRHEUMATIC AGENT, ANTIRHEUMATIC AGENT [TC], ANTIRHEUMATIC AGENTS, ANTIRHEUMATIC DISEASE MODIFYING SECOND LINE DRUGS, ANTIRHEUMATIC DISEASE-MODIFYING SECOND-LINE DRUGS, ANTIRHEUMATIC DRUGS, ANTIRHEUMATIC DRUGS DIS MODIFYING, ANTIRHEUMATIC DRUGS, DISEASE MODIFYING, ANTIRHEUMATIC DRUGS, DISEASE-MODIFYING, ANTIRHEUMATICS, DIS MODIFYING ANTIRHEUMATIC DRUGS, DISEASE MODIFYING ANTIRHEUMATIC DRUGS, DISEASE MODIFYING, ANTIRHEUMATIC SECOND LINE DRUGS, DISEASE-MODIFYING ANTIRHEUMATIC DRUG, DISEASE-MODIFYING ANTIRHEUMATIC DRUGS, DISEASE-MODIFYING, ANTIRHEUMATIC SECOND-LINE DRUGS, DRUGS, ANTI-RHEUMATIC, DRUGS, ANTIRHEUMATIC, DRUGS, DISEASE-MODIFYING ANTIRHEUMATIC, dmard

S12. Ethical Approval

All data partners received IRB approval or waiver in accordance to their institutional governance guidelines.

Database	Statement
AmbEMR	This is a retrospective database study on de-identified data and is deemed not human subject research. Approval is provided for OHDSI community studies.
CCAE	New England Institutional Review Board (IRB) and was determined to be exempt from broad IRB approval, as this research project did not involve human subject research.
CPRD	Approval for CPRD was provided by the Independent Scientific Advisory Committee (ISAC).<br>This study is based in part on data from the Clinical Practice Research Datalink obtained under licence from the UK Medicines and Healthcare products Regulatory Agency. The data is provided by patients and collected by the NHS as part of their care and support. The interpretation and conclusions contained in this study are those of the author/s alone. The protocol for this study (20_059R) was approved by the Independent Scientific Advisory Committee (ISAC).
DA Germany	This is a retrospective database study on de-identified data and is deemed not human subject research. Approval is provided for OHDSI community studies.
IMRD	The present study is filed and under review for Scientific Review Committee for institutional adjudication. Due to the public health imperative of information related to these data, approval is provided for this publication.
IPCI	The present study was approved by the Scientific and Ethical Advisory Board of the IPCI project (project number: 4/2020).
JMDC	New England Institutional Review Board (IRB) and was determined to be exempt from broad IRB approval, as this research project did not involve human subject research.
MDCD	New England Institutional Review Board (IRB) and was determined to be exempt from broad IRB approval, as this research project did not involve human subject research.
MDCD	New England Institutional Review Board (IRB) and was determined to be exempt from broad IRB approval, as this research project did not involve human subject research.

S12, Continued.

Database	Statement
Open Claims	This is a retrospective database study on de-identified data and is deemed not human subject research. Approval is provided for OHDSI community studies.
Clinformatics	New England Institutional Review Board (IRB) and was determined to be exempt from broad IRB approval, as this research project did not involve human subject research.
Optum EHR	New England Institutional Review Board (IRB) and was determined to be exempt from broad IRB approval, as this research project did not involve human subject research.
SIDIAP	The use of SIDIAP data base was approved by the SIDIAP Scientific Committee and the IDIAPJGol Clinical Research Ethics Committee.
VA	The use of VA data was reviewed by the Department of Veterans Affairs Central Institutional Review Board (IRB) and was determined to meet the criteria for exemption under Exemption Category 4(3) and approved the request for Waiver of HIPAA Authorization. The VA Privacy Office certified the release of aggregate analysis results for the meta-analysis.

S13. References

1. Suchard MA, Schuemie MJ, Krumholz HM, et al. Comprehensive comparative effectiveness and safety of first-line antihypertensive drug classes: a systematic, multinational, large-scale analysis. *Lancet* 2019; **394**(10211): 1816-26.

2. Voss EA, Boyce RD, Ryan PB, van der Lei J, Rijnbeek PR, Schuemie MJ. Accuracy of an automated knowledge base for identifying drug adverse reactions. *Journal of biomedical informatics* 2017; **66**: 72-81.

3. Singh S, Fouayzi H, Anzuoni K, Goldman L, Min JY, Griffin M *et al.* Diagnostic algorithms for cardiovascular death in administrative claims databases: A systematic review. *Drug Saf* 2018; **23**: 018–0754.

4. Ooba N, Setoguchi S, Ando T, Sato T, Yamaguchi T, Mochizuki M *et al.* Claims-based definition of death in japanese claims database: Validity and implications. *PLoS One* 2013; **8**: e66116. doi: 10.1371/journal.pone.0066116. Print 2013.

5. Robinson TE, Elley CR, Kenealy T, Drury PL. Development and validation of a predictive risk model for all-cause mortality in type 2 diabetes. *Diabetes Res Clin Pract* 2015; **108**: 482–8. doi: 10.1016/j.diabres.2015.02.015. Epub 2015 Mar 16.

6. Ammann EM, Schweizer ML, Robinson JG, Eschol JO, Kafa R, Girotra S *et al.* Chart validation of inpatient ICD-9-CM administrative diagnosis codes for acute myocardial infarction (AMI) among intravenous immune globulin (IGIV) users in the sentinel distributed database. *Pharmacoepidemiol Drug Saf* 2018; **27**: 398–404. doi: 10.1002/pds.4398. Epub 2018 Feb 15.

7. Floyd JS, Blondon M, Moore KP, Boyko EJ, Smith NL. Validation of methods for assessing cardiovascular disease using electronic health data in a cohort of veterans with diabetes. *Pharmacoepidemiol Drug Saf* 2016; **25**: 467–71. doi: 10.1002/pds.3921. Epub 2015 Nov 11.

8. Rubbo B, Fitzpatrick NK, Denaxas S, Daskalopoulou M, Yu N, Patel RS *et al.* Use of electronic health records to ascertain, validate and phenotype acute myocardial infarction: A systematic review and recommendations. *Int J Cardiol* 2015; **187:705-11.**: 10.1016/j.ijcard.2015.03.075. Epub 2015 Mar 5.

9. Wahl PM, Rodgers K, Schneeweiss S, Gage BF, Butler J, Wilmer C *et al.* Validation of claims-based diagnostic and procedure codes for cardiovascular and gastrointestinal serious adverse events in a commercially-insured population. *Pharmacoepidemiol Drug Saf* 2010; **19**: 596–603. doi: 10.1002/pds.1924.

10. Normand SL, Morris CN, Fung KS, McNeil BJ, Epstein AM. Development and validation of a claims based index for adjusting for risk of mortality: The case of acute myocardial infarction. *J Clin Epidemiol* 1995; **48**: 229–43.

11. Zaher C, Goldberg GA, Kadlubek P. Estimating angina prevalence in a managed care population. *Am J Manag Care* 2004; **10**: S339–46.

12. Floyd JS, Wellman R, Fuller S, Bansal N, Psaty BM, Boer IH de *et al.* Use of electronic health data to estimate heart failure events in a Population-Based cohort with CKD. *Clin J Am Soc Nephrol* 2016; **11**: 1954–1961. doi: 10.2215/CJN.03900416. Epub 2016 Aug 9.

13. Gini R, Schuemie MJ, Mazzaglia G, Lapi F, Francesconi P, Pasqua A *et al.* Automatic identification of type 2 diabetes, hypertension, ischaemic heart disease, heart failure and their levels of severity from italian general practitioners' electronic medical records: A validation study. *BMJ Open* 2016; **6**: e012413. doi: 10.1136/bmjopen–2016–012413.

14. Kaspar M, Fette G, Guder G, Seidlmayer L, Ertl M, Dietrich G *et al.* Underestimated prevalence of heart failure in hospital inpatients: A comparison of ICD codes and discharge letter information. *Clin Res Cardiol* 2018; **107**: 778–787. doi: 10.1007/s00392–018–1245–z.Epub 2018 Apr 17.

15. Li Q, Glynn RJ, Dreyer NA, Liu J, Mogun H, Setoguchi S. Validity of claims-based definitions of left ventricular systolic dysfunction in medicare patients. *Pharmacoepidemiol Drug Saf* 2011; **20**: 700–8. doi: 10.1002/pds.2146. Epub 2011 May 14.

16. Patel YR, Robbins JM, Kurgansky KE, Imran T, Orkaby AR, McLean RR *et al.* Development and validation of a heart failure with preserved ejection fraction cohort using electronic medical records. *BMC Cardiovasc Disord* 2018; **18**: 128. doi: 10.1186/s12872–018–0866–5.

17. Saczynski JS, Andrade SE, Harrold LR, Tjia J, Cutrona SL, Dodd KS *et al.* A systematic review of validated methods for identifying heart failure using administrative data. *Pharmacoepidemiol Drug Saf* 2012; **21**: 129–40. doi: 10.1002/pds.2313.

18. Schultz SE, Rothwell DM, Chen Z, Tu K. Identifying cases of congestive heart failure from administrative data: A validation study using primary care patient records. *Chronic Dis Inj Can* 2013; **33**: 160–6.

19. Feder SL, Redeker NS, Jeon S, Schulman-Green D, Womack JA, Tate JP *et al.* Validation of the ICD-9 diagnostic code for palliative care in patients hospitalized with heart failure within the veterans health administration. *Am J Hosp Palliat Care* 2018; **35**: 959–965. doi: 10.1177/1049909117747519. Epub 2017 Dec 18.

20. Rosenman M, He J, Martin J, Nutakki K, Eckert G, Lane K *et al.* Database queries for hospitalizations for acute congestive heart failure: Flexible methods and validation based on set theory. *J Am Med Inform Assoc* 2014; **21**: 345–52. doi: 10.1136/amiajnl–2013–001942.Epub 2013 Oct 10.

21. Quach S, Blais C, Quan H. Administrative data have high variation in validity for recording heart failure. *Can J Cardiol* 2010; **26**: 306–12.

22. Hennessy S, Leonard CE, Freeman CP, Deo R, Newcomb C, Kimmel SE *et al.* Validation of diagnostic codes for outpatient-originating sudden cardiac death and ventricular arrhythmia in medicaid and medicare claims data. *Pharmacoepidemiol Drug Saf* 2010; **19**: 555–62. doi: 10.1002/pds.1869.

23. Tamariz L, Harkins T, Nair V. A systematic review of validated methods for identifying ventricular arrhythmias using administrative and claims data. *Pharmacoepidemiol Drug Saf* 2012; **21**: 148–53. doi: 10.1002/pds.2340.

24. Gage BF, Waterman AD, Shannon W, Boechler M, Rich MW, Radford MJ. Validation of clinical classification schemes for predicting stroke: Results from the national registry of atrial fibrillation. *JAMA* 2001; **285**: 2864–70.

25. Jensen PN, Johnson K, Floyd J, Heckbert SR, Carnahan R, Dublin S. A systematic review of validated methods for identifying atrial fibrillation using administrative data. *Pharmacoepidemiol Drug Saf* 2012; **21**: 141–7. doi: 10.1002/pds.2317.

26. Karnik S, Tan SL, Berg B, Glurich I, Zhang J, Vidaillet HJ *et al.* Predicting atrial fibrillation and flutter using electronic health records. *Conf Proc IEEE Eng Med Biol Soc* 2012; **2012:5562-5.**: 10.1109/EMBC.2012.6347254.

27. Navar-Boggan AM, Rymer JA, Piccini JP, Shatila W, Ring L, Stafford JA *et al.* Accuracy and validation of an automated electronic algorithm to identify patients with atrial fibrillation at risk for stroke. *Am Heart J* 2015; **169**: 39–44.e2. doi: 10.1016/j.ahj.2014.09.014. Epub 2014 Oct 22.

28. Yahi A, Tatonetti NP. A knowledge-based, automated method for phenotyping in the EHR using only clinical pathology reports. *AMIA Jt Summits Transl Sci Proc* 2015; **2015**: 64–8. eCollection 2015.

29. Shin J, Gonzales M, Pletcher MJ. Risk of emergent bradycardia associated with initiation of immediate- or slow-release metoprolol. *Pharmacotherapy* 2013; **33**: 1353–61. doi: 10.1002/phar.1319. Epub 2013 Jun 27.

30. Turgeon RD, Fernandes KA, Juurlink D, Tu JV, Mamdani M. Ticagrelor and bradycardia: A nested case-control study. *Pharmacoepidemiol Drug Saf* 2015; **24**: 1281–5. doi: 10.1002/pds.3884. Epub 2015 Oct 7.

31. Andrade SE, Harrold LR, Tjia J, Cutrona SL, Saczynski JS, Dodd KS *et al.* A systematic review of validated methods for identifying cerebrovascular accident or transient ischemic attack using administrative data. *Pharmacoepidemiol Drug Saf* 2012; **21**: 100–28. doi: 10.1002/pds.2312.

32. Yuan Z, Voss EA, DeFalco FJ, Pan G, Ryan PB, Yannicelli D *et al.* Risk prediction for ischemic stroke and transient ischemic attack in patients without atrial fibrillation: A retrospective cohort study. *J Stroke Cerebrovasc Dis* 2017; **26**: 1721–1731. doi: 10.1016/j.jstrokecerebrovasdis.2017.03.036. Epub 2017 Apr 6.

33. Park TH, Choi JC. Validation of stroke and thrombolytic therapy in korean national health insurance claim data. *J Clin Neurol* 2016; **12**: 42–8. doi: 10.3988/jcn.2016.12.1.42. Epub 2015 Sep 11.

34. Gon Y, Kabata D, Yamamoto K, Shintani A, Todo K, Mochizuki H *et al.* Validation of an algorithm that determines stroke diagnostic code accuracy in a japanese hospital-based cancer registry using electronic medical records. *BMC Med Inform Decis Mak* 2017; **17**: 157. doi: 10.1186/s12911–017–0554–x.

35. Sung SF, Hsieh CY, Lin HJ, Chen YW, Yang YH, Li CY. Validation of algorithms to identify stroke risk factors in patients with acute ischemic stroke, transient ischemic attack, or intracerebral hemorrhage in an administrative claims database. *Int J Cardiol* 2016; **215:277-82.**: 10.1016/j.ijcard.2016.04.069. Epub 2016 Apr 14.

36. Tu K, Wang M, Young J, Green D, Ivers NM, Butt D *et al.* Validity of administrative data for identifying patients who have had a stroke or transient ischemic attack using EMRALD as a reference standard. *Can J Cardiol* 2013; **29**: 1388–94. doi: 10.1016/j.cjca.2013.07.676. Epub 2013 Sep 26.

37. Tamariz L, Harkins T, Nair V. A systematic review of validated methods for identifying venous thromboembolism using administrative and claims data. *Pharmacoepidemiol Drug Saf* 2012; **21**: 154–62. doi: 10.1002/pds.2341.

38. Burwen DR, Wu C, Cirillo D, Rossouw JE, Margolis KL, Limacher M *et al.* Venous thromboembolism incidence, recurrence, and mortality based on women's health initiative data and medicare claims. *Thromb Res* 2017; **150:78-85.**: 10.1016/j.thromres.2016.11.015. Epub 2016 Nov 15.

39. Coleman CI, Peacock WF, Fermann GJ, Crivera C, Weeda ER, Hull M *et al.* External validation of a multivariable claims-based rule for predicting in-hospital mortality and 30-day post-pulmonary embolism complications. *BMC Health Serv Res* 2016; **16**: 610. doi: 10.1186/s12913–016–1855–y.

40. Ammann EM, Cuker A, Carnahan RM, Perepu US, Winiecki SK, Schweizer ML *et al.* Chart validation of inpatient international classification of diseases, ninth revision, clinical modification (ICD-9-CM) administrative diagnosis codes for venous thromboembolism (VTE) among intravenous immune globulin (IGIV) users in the sentinel distributed database. *Medicine* 2018; **97**: e9960. doi: 10.1097/MD.0000000000009960.

41. Goldstein JL, Zhao SZ, Burke TA, Palmer R, Allmen H von, Henderson SC. Incidence of outpatient physician claims for upper gastrointestinal symptoms among new users of celecoxib, ibuprofen, and naproxen in an insured population in the united states. *Am J Gastroenterol* 2003; **98**: 2627–34. doi: 10.1111/j.1572–0241.2003.08722.x.

42. Curtis JR, Chen SY, Werther W, John A, Johnson DA. Validation of ICD-9-CM codes to identify gastrointestinal perforation events in administrative claims data among hospitalized rheumatoid arthritis patients. *Pharmacoepidemiol Drug Saf* 2011; **20**: 1150–8. doi: 10.1002/pds.2215. Epub 2011 Aug 27.

43. Lanza LL, Walker AM, Bortnichak EA, Dreyer NA. Peptic ulcer and gastrointestinal hemorrhage associated with nonsteroidal anti-inflammatory drug use in patients younger than 65 years. A large health maintenance organization cohort study. *Arch Intern Med* 1995; **155**: 1371–7.

44. Lin KJ, Garcia Rodriguez LA, Hernandez-Diaz S. Systematic review of peptic ulcer disease incidence rates: Do studies without validation provide reliable estimates? *Pharmacoepidemiol Drug Saf* 2011; **20**: 718–28. doi: 10.1002/pds.2153. Epub 2011 May 27.

45. Patorno E, Gagne JJ, Lu CY, Haynes K, Sterrett AT, Roy J *et al.* The role of hemoglobin laboratory test results for the detection of upper gastrointestinal bleeding outcomes resulting from the use of medications in observational studies. *Drug Saf* 2017; **40**: 91–100. doi: 10.1007/s40264–016–0472–3.

46. Valkhoff VE, Coloma PM, Masclee GM, Gini R, Innocenti F, Lapi F *et al.* Validation study in four health-care databases: Upper gastrointestinal bleeding misclassification affects precision but not magnitude of drug-related upper gastrointestinal bleeding risk. *J Clin Epidemiol* 2014; **67**: 921–31. doi: 10.1016/j.jclinepi.2014.02.020. Epub 2014 May 1.

47. Afzal Z, Schuemie MJ, Blijderveen JC van, Sen EF, Sturkenboom MC, Kors JA. Improving sensitivity of machine learning methods for automated case identification from free-text electronic medical records. *BMC Med Inform Decis Mak* 2013; **13:30.**: 10.1186/1472–6947–13–30.

48. Lenihan CR, Montez-Rath ME, Mora Mangano CT, Chertow GM, Winkelmayer WC. Trends in acute kidney injury, associated use of dialysis, and mortality after cardiac surgery, 1999 to 2008. *Ann Thorac Surg* 2013; **95**: 20–8. doi: 10.1016/j.athoracsur.2012.05.131. Epub 2012 Dec 25.

49. Winkelmayer WC, Schneeweiss S, Mogun H, Patrick AR, Avorn J, Solomon DH. Identification of individuals with CKD from medicare claims data: A validation study. *Am J Kidney Dis* 2005; **46**: 225–32. doi: 10.1053/j.ajkd.2005.04.029.

50. Grams ME, Waikar SS, MacMahon B, Whelton S, Ballew SH, Coresh J. Performance and limitations of administrative data in the identification of AKI. *Clin J Am Soc Nephrol* 2014; **9**: 682–9. doi: 10.2215/CJN.07650713. Epub 2014 Jan 23.

51. Arnold J, Ng KP, Sims D, Gill P, Cockwell P, Ferro C. Incidence and impact on outcomes of acute kidney injury after a stroke: A systematic review and meta-analysis. *BMC Nephrol* 2018; **19**: 283. doi: 10.1186/s12882–018–1085–0.

52. Sutherland SM, Byrnes JJ, Kothari M, Longhurst CA, Dutta S, Garcia P *et al.* AKI in hospitalized children: Comparing the pRIFLE, AKIN, and KDIGO definitions. *Clin J Am Soc Nephrol* 2015; **10**: 554–61. doi: 10.2215/CJN.01900214. Epub 2015 Feb 3.

53. Waikar SS, Wald R, Chertow GM, Curhan GC, Winkelmayer WC, Liangos O *et al.* Validity of international classification of diseases, ninth revision, clinical modification codes for acute renal failure. *J Am Soc Nephrol* 2006; **17**: 1688–94. doi: 10.1681/ASN.2006010073. Epub 2006 Apr 26.

54. Rhee C, Murphy MV, Li L, Platt R, Klompas M. Improving documentation and coding for acute organ dysfunction biases estimates of changing sepsis severity and burden: A retrospective study. *Crit Care* 2015; **19:338.**: 10.1186/s13054–015–1048–9.

55. Chen HJ, Wang JJ, Tsay WI, Her SH, Lin CH, Chien CC. Epidemiology and outcome of acute pancreatitis in end-stage renal disease dialysis patients: A 10-year national cohort study. *Nephrol Dial Transplant* 2017; **32**: 1731–1736. doi: 10.1093/ndt/gfw400.

56. Muntner P, Gutierrez OM, Zhao H, Fox CS, Wright NC, Curtis JR *et al.* Validation study of medicare claims to identify older US adults with CKD using the reasons for geographic and racial differences in stroke (REGARDS) study. *Am J Kidney Dis* 2015; **65**: 249–58. doi: 10.1053/j.ajkd.2014.07.012. Epub 2014 Sep 19.

57. Shen AK, Kelman JA, Warnock R, Zhang W, Brereton S, McKean S *et al.* Beneficiary characteristics and vaccinations in the end-stage renal disease medicare beneficiary population, an analysis of claims data 2006-2015. *Vaccine* 2017; **35**: 7302–7308. doi: 10.1016/j.vaccine.2017.10.105. Epub 2017 Nov 10.

58. Bui CL, Kaye JA, Castellsague J, Calingaert B, McQuay LJ, Riera-Guardia N *et al.* Validation of acute liver injury cases in a population-based cohort study of oral antimicrobial users. *Curr Drug Saf* 2014; **9**: 23–8.

59. Cheetham TC, Lee J, Hunt CM, Niu F, Reisinger S, Murray R *et al.* An automated causality assessment algorithm to detect drug-induced liver injury in electronic medical record data. *Pharmacoepidemiol Drug Saf* 2014; **23**: 601–8. doi: 10.1002/pds.3531. Epub 2013 Oct 21.

60. Jinjuvadia K, Kwan W, Fontana RJ. Searching for a needle in a haystack: Use of ICD-9-CM codes in drug-induced liver injury. *Am J Gastroenterol* 2007; **102**: 2437–43. doi: 10.1111/j.1572–0241.2007.01456.x. Epub 2007 Jul 27.

61. Lo Re V 3rd, Carbonari DM, Forde KA, Goldberg D, Lewis JD, Haynes K *et al.* Validity of diagnostic codes and laboratory tests of liver dysfunction to identify acute liver failure events. *Pharmacoepidemiol Drug Saf* 2015; **24**: 676–83. doi: 10.1002/pds.3774. Epub 2015 Apr 10.

62. Lo Re V 3rd, Haynes K, Goldberg D, Forde KA, Carbonari DM, Leidl KB *et al.* Validity of diagnostic codes to identify cases of severe acute liver injury in the US food and drug administration's Mini-Sentinel distributed database. *Pharmacoepidemiol Drug Saf* 2013; **22**: 861–72. doi: 10.1002/pds.3470. Epub 2013 Jun 25.

63. Overby CL, Pathak J, Gottesman O, Haerian K, Perotte A, Murphy S *et al.* A collaborative approach to developing an electronic health record phenotyping algorithm for drug-induced liver injury. *J Am Med Inform Assoc* 2013; **20**: e243–52. doi: 10.1136/amiajnl–2013–001930.Epub 2013 Jul 9.

64. Udo R, Maitland-van der Zee AH, Egberts TC, Breeijen JH den, Leufkens HG, Solinge WW van *et al.* Validity of diagnostic codes and laboratory measurements to identify patients with idiopathic acute liver injury in a hospital database. *Pharmacoepidemiol Drug Saf* 2016; **25**: 21–8. doi: 10.1002/pds.3824. Epub 2015 Jul 5.

65. Wing K, Bhaskaran K, Smeeth L, Staa TP van, Klungel OH, Reynolds RF *et al.* Optimising case detection within UK electronic health records: Use of multiple linked databases for detecting liver injury. *BMJ Open* 2016; **6**: e012102. doi: 10.1136/bmjopen–2016–012102.

66. Dore DD, Chaudhry S, Hoffman C, Seeger JD. Stratum-specific positive predictive values of claims for acute pancreatitis among commercial health insurance plan enrollees with diabetes mellitus. *Pharmacoepidemiol Drug Saf* 2011; **20**: 209–13. doi: 10.1002/pds.2077. Epub 2010 Dec 23.

67. Dore DD, Hussein M, Hoffman C, Pelletier EM, Smith DB, Seeger JD. A pooled analysis of exenatide use and risk of acute pancreatitis. *Curr Med Res Opin* 2013; **29**: 1577–86. doi: 10.1185/03007995.2013.838550. Epub 2013 Sep 13.

68. Yabe D, Kuwata H, Kaneko M, Ito C, Nishikino R, Murorani K *et al.* Use of the japanese health insurance claims database to assess the risk of acute pancreatitis in patients with diabetes: Comparison of DPP-4 inhibitors with other oral antidiabetic drugs. *Diabetes Obes Metab* 2015; **17**: 430–4. doi: 10.1111/dom.12381. Epub 2014 Sep 17.
